# Chalcogen‐Bonding Interactions in Telluroether Heterocycles [Te(CH_2_)_*m*_]_*n*_ (*n=*1–4; *m=*3–7)

**DOI:** 10.1002/chem.202002510

**Published:** 2020-10-16

**Authors:** Marko Rodewald, J. Mikko Rautiainen, Tobias Niksch, Helmar Görls, Raija Oilunkaniemi, Wolfgang Weigand, Risto S. Laitinen

**Affiliations:** ^1^ Institut für Anorganische und Analytische Chemie Friedrich-Schiller-Universität Jena Humboldt Strasse 8 07743 Jena Germany; ^2^ Department of Chemistry, Nanoscience Center University of Jyväskylä P.O. Box 35 40014 Jyväskylä Finland; ^3^ Klinik für Nuklearmedizin Universitätsklinikum Jena Am Klinikum 1 07747 Jena Germany; ^4^ Laboratory of Inorganic Chemistry, Environmental and Chemical Engineering University of Oulu P.O. Box 3000 90014 Oulu Finland

**Keywords:** density functional calculations, heterocycles, noncovalent interactions, solid-state structures, tellurium

## Abstract

The Te**⋅⋅⋅**Te secondary bonding interactions (SBIs) in solid cyclic telluroethers were explored by preparing and structurally characterizing a series of [Te(CH_2_)_*m*_]_*n*_ (*n=*1–4; *m=*3–7) species. The SBIs in 1,7‐Te_2_(CH_2_)_10_, 1,8‐Te_2_(CH_2_)_12_, 1,5,9‐Te_3_(CH_2_)_9_, 1,8,15‐Te_3_(CH_2_)_18_, 1,7,13,19‐Te_4_(CH_2_)_20_, 1,8,15,22‐Te_4_(CH_2_)_24_ and 1,9,17,25‐Te_4_(CH_2_)_28_ lead to tubular packing of the molecules, as has been observed previously for related thio‐ and selenoether rings. The nature of the intermolecular interactions was explored by solid‐state PBE0‐D3/pob‐TZVP calculations involving periodic boundary conditions. The molecular packing in 1,7,13,19‐Te_4_(CH_2_)_20_, 1,8,15,22‐Te_4_(CH_2_)_24_ and 1,9,17,25‐Te_4_(CH_2_)_28_ forms infinite shafts. The electron densities at bond critical points indicate a narrow range of Te**⋅⋅⋅**Te bond orders of 0.12–0.14. The formation of the shafts can be rationalized by frontier orbital overlap and charge transfer.

## Graphical abstract

**Tubular stacks**: The nature of the Te**⋅⋅⋅**Te chalcogen‐bonding interactions in solid telluroethers 1,7‐Te_2_(CH_2_)_10_, 1,8‐Te_2_(CH_2_)_12_, 1,5,9‐Te_3_(CH_2_)_9_, 1,8,15‐Te_3_(CH_2_)_18_, 1,7,13,19‐Te_4_(CH_2_)_20_, 1,8,15,22‐Te_4_(CH_2_)_24_, and 1,9,17,25‐Te_4_(CH_2_)_28_ was explored by single‐crystal XRD and solid‐state PBE0‐D3/pob‐TZVP calculations involving periodic boundary conditions. Tubular stacking of the larger macrocycles results in the formation of infinite tubular shafts.

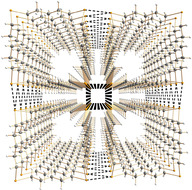

## Introduction

Chalcogen bonding, which has recently been formally defined by IUPAC,^[1]^ is a special class of secondary bonding interactions (SBIs), a term that was originally coined by Alcock.^[2]^ SBIs are interatomic interactions that are longer than covalent single bonds but shorter than the sums of the van der Waals radii. Chalcogen bonds are most prominent for the heavy chalcogen atoms tellurium and selenium and thus resemble the halogen,[^1a^, ^3^] pnictogen and tetrel bonds[^4^, ^5^] of the heavy p‐block elements. The chalcogen bonds can be understood as combinations of orbital interactions, as well as electrostatic and dispersion contributions.^[6]^ They are also called σ‐hole,[^3b^, ^3c^, ^7^] noncovalent, semibonding, nonbonding, weakly bonding, closed‐shell or soft–soft interactions. In the early literature, the SBIs involving chalcogen atoms have been referred to as chalcogen–chalcogen interactions.[^1^, ^6a^] This has now evolved to the more general term chalcogen bonding.

The covalent aspects of the secondary bonding interactions can be described as donor–acceptor interactions n^2^(D)→σ*(E−X) in which the lone pair orbital of the electron‐donor atom D overlaps with the antibonding σ* orbital of the E−X bond (E=heavy p‐block element; X=an electronegative atom).^[6]^ The strength of this 3c–4e arrangement varies from a very weak interaction to that of a hypervalent single bond. Note that in the IUPAC definition of chalcogen bond, the chalcogen‐bond donor is the chalcogen atom that acts as the electron acceptor.^[1]^ The heaviest p‐block elements show the strongest SBIs, because the energy difference between the σ(E−X) and σ*(E−X) orbitals is diminished on going down the periodic table.^[6]^ At the same time, the orbital overlap decreases, since the orbitals become more diffuse. The dispersion effects become more significant with increasing period number and play the major role in the interactions between the heaviest p‐block elements.

This contribution is concerned with chalcogen bonds involving tellurium atoms. The relative strengths of tellurium chalcogen bonds are dependent on the identity of the acceptor atom. The strongest interactions are observed when the acceptor atom is oxygen or nitrogen.^[6d]^ However, tellurium–tellurium interactions are also known, as exemplified by the hexagonal allotrope of tellurium.^[8]^ The trigonal Te_*n*_ chains show close Te**⋅⋅⋅**Te contacts between the adjacent chains, which expand the formal coordination sphere of the chalcogen atom to an elongated octahedron.

Trends in chalcogen‐bond strength have been studied, for example, in a series of trichalcogenaferrocenophanes [Fe(C_5_H_4_E)_2_E′] (E, E′=S, Se, Te).^[9]^ The complexes containing only sulfur or selenium show weak interactions between the chalcogen atoms, whereas in tellurium‐containing complexes the chalcogen bonds between chalcogen atoms are significantly stronger. All these tellurium complexes form supramolecular networks with continuous quasi‐2D layer structures.

Macrocyclic unsaturated chalcogenoethers are another class of compounds that can further our understanding of chalcogen bonds. These types of compounds have been extensively studied both computationally and experimentally by Gleiter and co‐workers.[^6a^, ^6e^, ^6f^, ^6g^, ^6h^, ^10^] Due to the chalcogen‐bonding interactions, the ring molecules are often packed in a columnar fashion to form tubular lattices. This packing of cyclic species is also mimicked by some related acyclic dimethyl polyalkynyl diseleno‐ and ditelluroethers.^[11]^

Herein, we explore the packing of macrocyclic aliphatic telluroethers. A number of related thioethers,^[12]^ selenoethers,^[13]^ hybrid thioselenoethers[^12l^, ^13b^, ^14^] and thiotelluroethers^[12l]^ have been characterized, but structural information on macrocyclic telluroethers is much sparser. Besides the few hybrid thiotelluroether macrocycles,^[12l]^ only the preparation of Te(CH_2_)_4_,^[15]^ Te(CH_2_)_5_ ^[16]^ and 1,5,9‐Te_3_(CH_2_)_9_ ^[17]^ has been previously reported. Although no crystal structures are known for these species, there is NMR spectroscopic evidence for their existence.[^17^, ^18^]

**Scheme 1:**
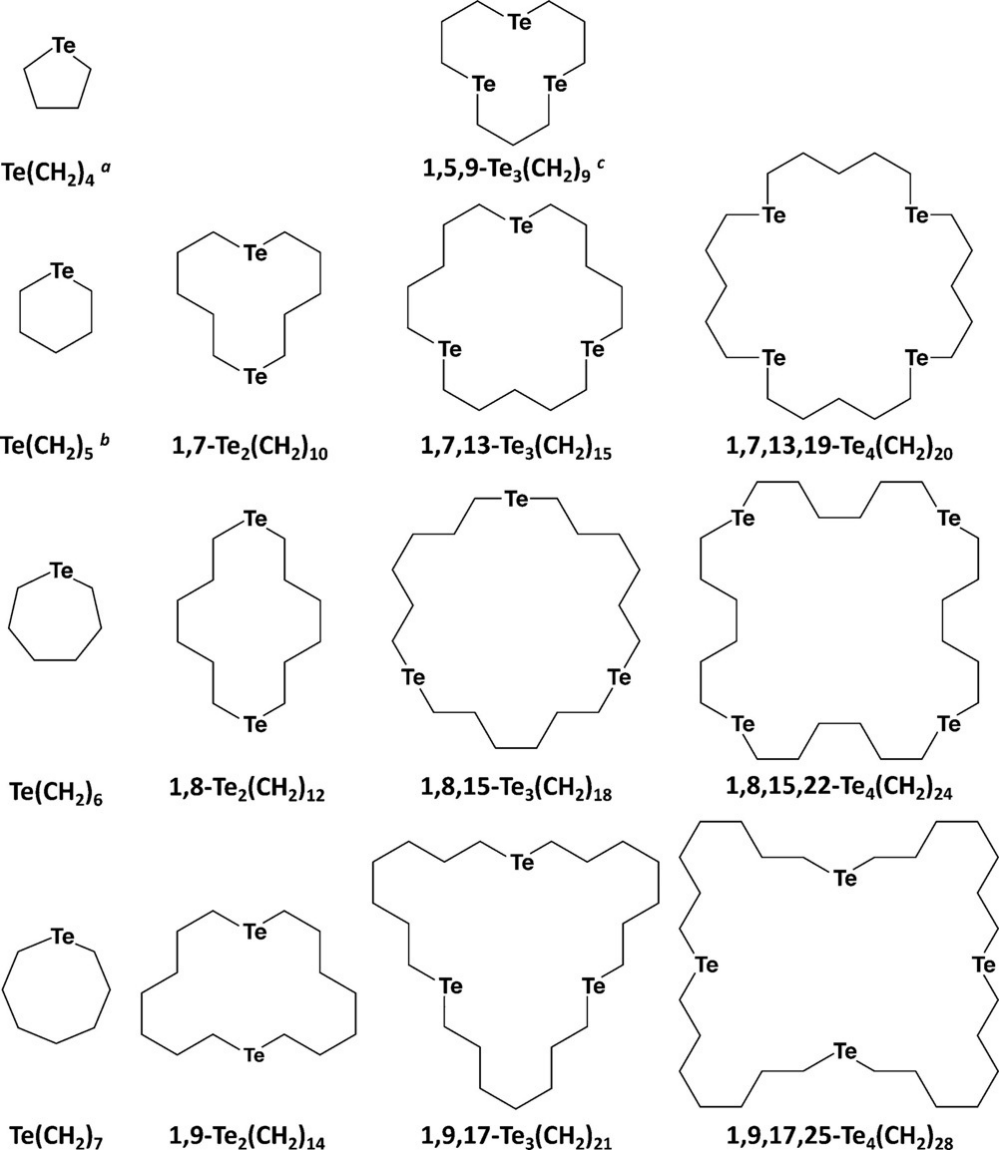
Telluroether heterocycles [Te(CH_2_)_*m*_]_*n*_ (*n=*1–4; *m=*3–7) considered in this work. [a] ref. [15]; [b] ref. [16]; [c] ref. [17].

We report on the ^1^H, ^13^C and ^125^Te NMR spectroscopic identification of the molecular species as well as the molecular structures of 1,7‐Te_2_(CH_2_)_10_, 1,8‐Te_2_(CH_2_)_12_, 1,5,9‐Te_3_(CH_2_)_9_, 1,8,15‐Te_3_(CH_2_)_18_, 1,7,13,19‐Te_4_(CH_2_)_20_, 1,8,15,22‐Te_4_(CH_2_)_24_ and 1,9,17,25‐Te_4_(CH_2_)_28_. The species studied in this work were prepared by reaction of sodium telluride with α,ω‐bromoalkanes Br(CH_2_)_*n*_Br with *n=*3–7 (Scheme 1). The nature of the molecular packing in the lattices was explored by solid‐state DFT (PBE0‐D3/pob‐TZVP) calculations involving periodic boundary conditions.

## Results and Discussion

### Formation and identification of the macrocyclic telluroethers

The reactions of Na_2_Te with α,ω‐dibromoalkanes Br(CH_2_)_*m*_Br (*m=*3–7) afforded mixtures of heterocyclic telluroethers with low to moderate yields of isolated compounds (see Table S1 in Supporting Information). The preparations were modelled after those of Morgan and co‐workers, who have reported the synthesis of Te(CH_2_)_4_ from elemental tellurium and I(CH_2_)_4_I,^[15]^ and of Te(CH_2_)_5_ from Al_3_Te_2_ and Br(CH_2_)_5_Br.^[16]^ Takaguchi et al.^[17]^ have reported the preparation of 1,5,9‐Te_3_(CH_2_)_9_ from Na_2_Te and Br(CH_3_)_3_Br.

**Figure 1:**
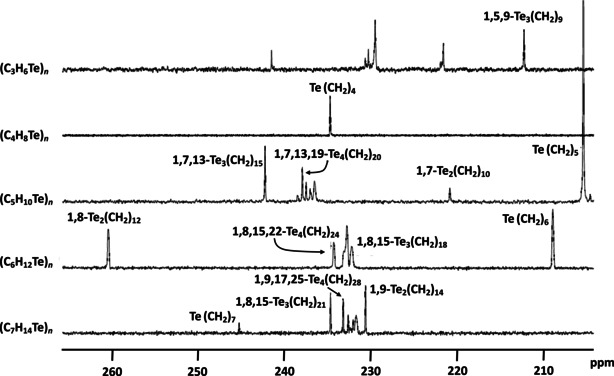
^125^Te{^1^H} NMR spectra of the product mixtures of the reactions of Na_2_Te and Br(CH_2_)_*m*_Br (*m=*3–7). The peak of Te(CH_2_)_5_ was cut off at 43 % of its height for better visibility of other reaction products. The partial assignment of the ^125^Te resonances is based on the spectroscopic information of the isolated species (see Supporting Information).

In most of our reaction mixtures, other products were present in addition to the isolated ones. They most likely include even larger rings, as indicated by very similar chemical shifts in the NMR spectra of the reaction mixtures (see Figure 1) and by TLC analysis (see Figure S1 in Supporting Information). Similarly, the formation of polymeric species seems likely as, during the column chromatographic workup, thick residues could be observed [except for the reactions involving Br(CH_2_)_4_Br] that only spread through the first column layers without being eluted through the column. Both identified products and unidentified side products were sensitive to light when dissolved, and neither side products nor decomposition products could be unambiguously identified. Particularly problematic in this regard were the reaction products of Na_2_Te and Br(CH_2_)_3_Br, for which multiple peaks were observed in the ^125^Te NMR spectra of the reaction mixtures, and the multiple spots in the TLC analyses indicated the presence of several products, of which only 1,5,9‐Te_3_(CH_2_)_9_ could be obtained after workup. 1,5,9‐Te_3_(CH_2_)_9_ was the only heterocyclic telluroether that showed notable sensitivity to light even as pure solid substance. Repeated attempts at this synthesis, however, yielded 1,5,9‐Te_3_(CH_2_)_9_ reliably. Te(CH_2_)_7_ was observed only as a trace species in the reaction mixture, as shown by the ^125^Te NMR spectrum (see Figure 1), and was only obtained together with 1,9‐Te_2_(CH_2_)_14_.

Unambiguous identification of the isolated substances was achieved by a combination of EIMS (see Figures S2–S9 in Supporting Information), NMR spectroscopy (see Figures S10–S99 in Supporting Information) and XRD. The purity of each sample after the column chromatographic separation was initially confirmed by ^125^Te{^1^H} NMR spectroscopy, whereby the pure compounds showed only a single peak. Single crystals of the seven substances whose molecular structures are reported herein were grown from these NMR samples and the very same crystals that were used for crystal structure determination were also subjected to EIMS. Thus, it could be inferred that, under the given ionization conditions, the heaviest ion detected was indeed the molecular ion for all compounds with the only exception being the largest ring system, 1,7,13,19‐Te_4_(CH_2_)_20_, which fragmented too strongly. The observed isotopic distribution patterns matched the calculated ones in all cases (see Figures S6–S9 in Supporting Information). This confirmed that EIMS could be used to identify the largest structure in each sample, the purity of which was confirmed by means of NMR spectroscopy beforehand. It led to a fully consistent assignment to the order in which the products were eluted from the column.

The integrated intensities of the chemical shifts of [Te(CH_2_)_*m*_]_*n*_ (*n=*1–4, *m=*5–7) in the ^125^Te NMR spectra shown in Figure 1 can be used to estimate the molar ratios of the different telluroether heterocycles formed in the reactions, as shown in Table 1.

**Table 1:** Normalized molar ratios [%] of [Te(CH_2_)_*m*_]_*n*_ (*n=*1–4, *m=*5–7) in the reaction mixtures, calculated from the integrated intensities of the chemical shifts in the ^125^Te{^1^H} NMR spectra.

Compound	*n=*1	*n=*2	*n=*3	*n=*4
[Te(CH_2_)_5_]_*n*_	93.2	1.7	3.5	1.6
[Te(CH_2_)_6_]_*n*_	60.6	20.6	12.1	6.7
[Te(CH_2_)_7_]_*n*_	17.6	44.2	22.7	15.5

**Scheme 2:**
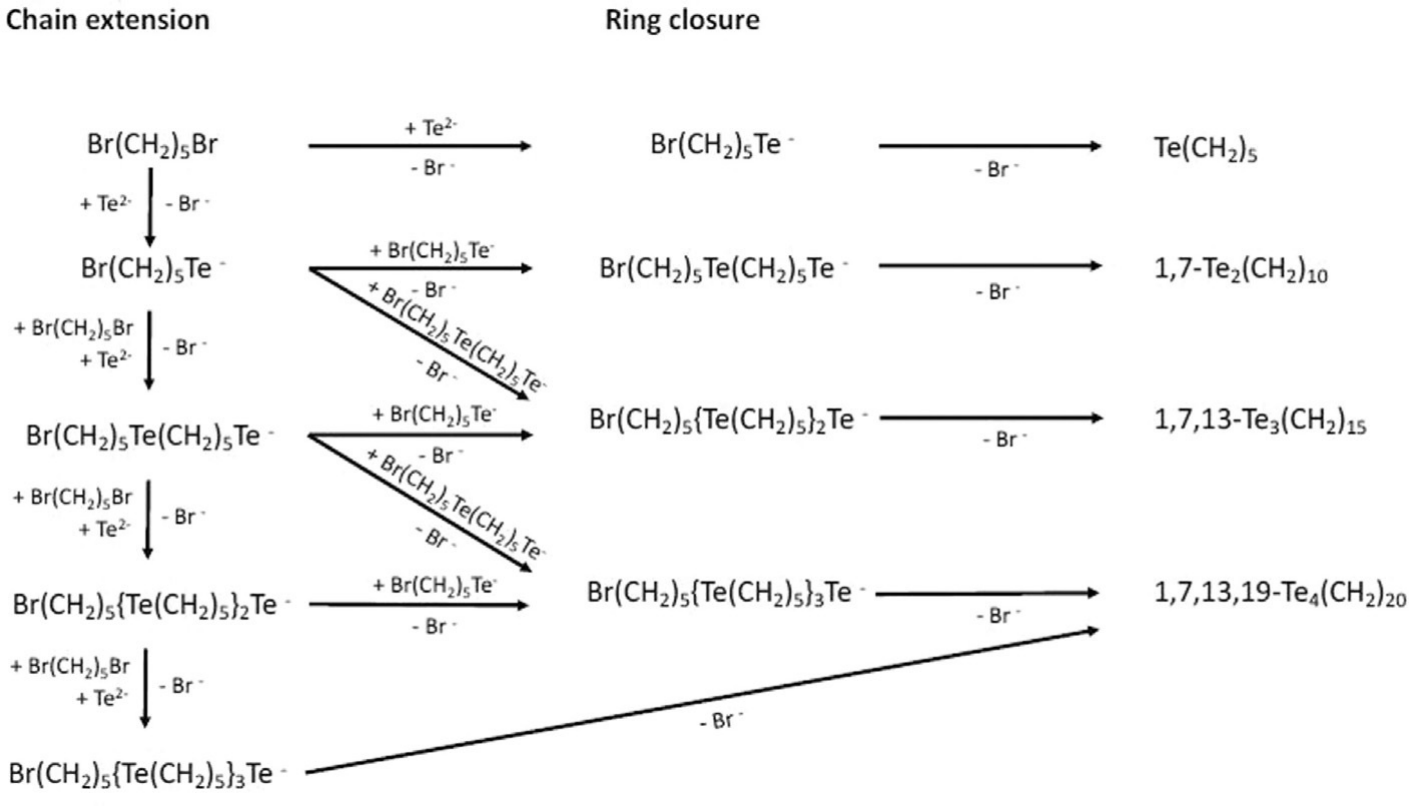
Possible pathways for the formation of [Te(CH_2_)_5_]_*n*_.

Following the observations of Morgan and Burgress^[15]^ and Morgan and Burstall,^[16]^ the formation of the [Te(CH_2_)_5_]_*n*_ is proposed to proceed through the pathways presented in Scheme 2. The process can be divided into two main stages: chain extension and ring closure. The [Te(CH_2_)_6_]_*n*_ and [Te(CH_2_)_7_]_*n*_ macrocycles are expected to show similar behaviour.

The formation of the mixtures of [Te(CH_2_)_5_]_*n*_, [Te(CH_2_)_6_]_*n*_ and [Te(CH_2_)_7_]_*n*_ (*n=*1–4) was also explored by dispersion‐corrected PBE0‐D3/def2‐TZVPP calculations with lattice‐energy contributions for the starting material Na_2_Te(c) and the side product NaBr(c) taken from PBE0‐D3/pob‐TZVP solid‐state calculations. The Gibbs energies for the formation of telluroether heterocycles are exemplified for the case of [Te(CH_2_)_5_]_*n*_ (*n=*1–4) heterocycles in Table 2.

**Table 2:** PBE0‐D3/def2‐TZVPP Gibbs energies of formation of [Te(CH_2_)_5_]_*n*_ (*n=*1−4) from equimolar reactions of Na_2_Te and Br(CH_2_)_5_Br. (solv=EtOH). The energy change refers to the molar ratios given in the reaction formulae.

Reagents	Products	Δ*G*_f_ [kJ]^[a]^
Na_2_Te(c)+ Br(CH_2_)_5_Br(solv)	Te(CH_2_)_5_(solv)+2 NaBr(c)	−241.6
	1/2 1,7‐Te_2_(CH_2_)_10_(solv)+2 NaBr(c)	−217.6
	1/3 1,7,13‐Te_3_(CH_2_)_15_(solv)+2 NaBr(c)	−214.3
	1/4 1,7,13,19‐Te_4_(CH_2_)_20_(solv)+2 NaBr(c)	−212.5

[a] Gibbs energy change refers to the molar ratios given in the reaction formulae.

All four equimolar reactions of Na_2_Te and Br(CH_2_)_5_Br shown in Table 2 are exergonic with approximately equal Gibbs energy changes. Whereas all four alternative reactions are therefore thermodynamically equally probable at 298 K, the activation energies of these reactions determine the relative rates of formation of different species. Ring closure is kinetically favoured in case of unstrained rings. Due to fast ring closure, Te(CH_2_)_4_ is the only observed species in the reaction mixture. It can also be seen in the molar ratios shown in Table 1 that in the case of [Te(CH_2_)_5_]_*n*_ the ring closure is still the fastest process but not as dominant, and therefore [Te(CH_2_)_5_]_*n*_ is not the only observed product. With [Te(CH_2_)_6_]_*n*_, the faster ring closure still results in Te(CH_2_)_6_ being the main product, but the relative amounts of the larger rings become more significant. With [Te(CH_2_)_7_]_*n*_ the ring closure is finally not the faster process anymore, and 1,9‐Te_2_(CH_2_)_14_ is observed to be more abundant.

### Molecular structures

**Figure 2:**
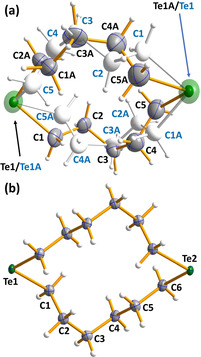
Molecular structures of a) 1,7‐Te_2_(CH_2_)_10_. and b) 1,8‐Te_2_(CH_2_)_12_. The anisotropic displacement parameters are shown at 50 % probability. Selected bond lengths and angles are listed in Table S3 in the Supporting Information. Te is depicted in green and C in grey.

**Figure 3:**
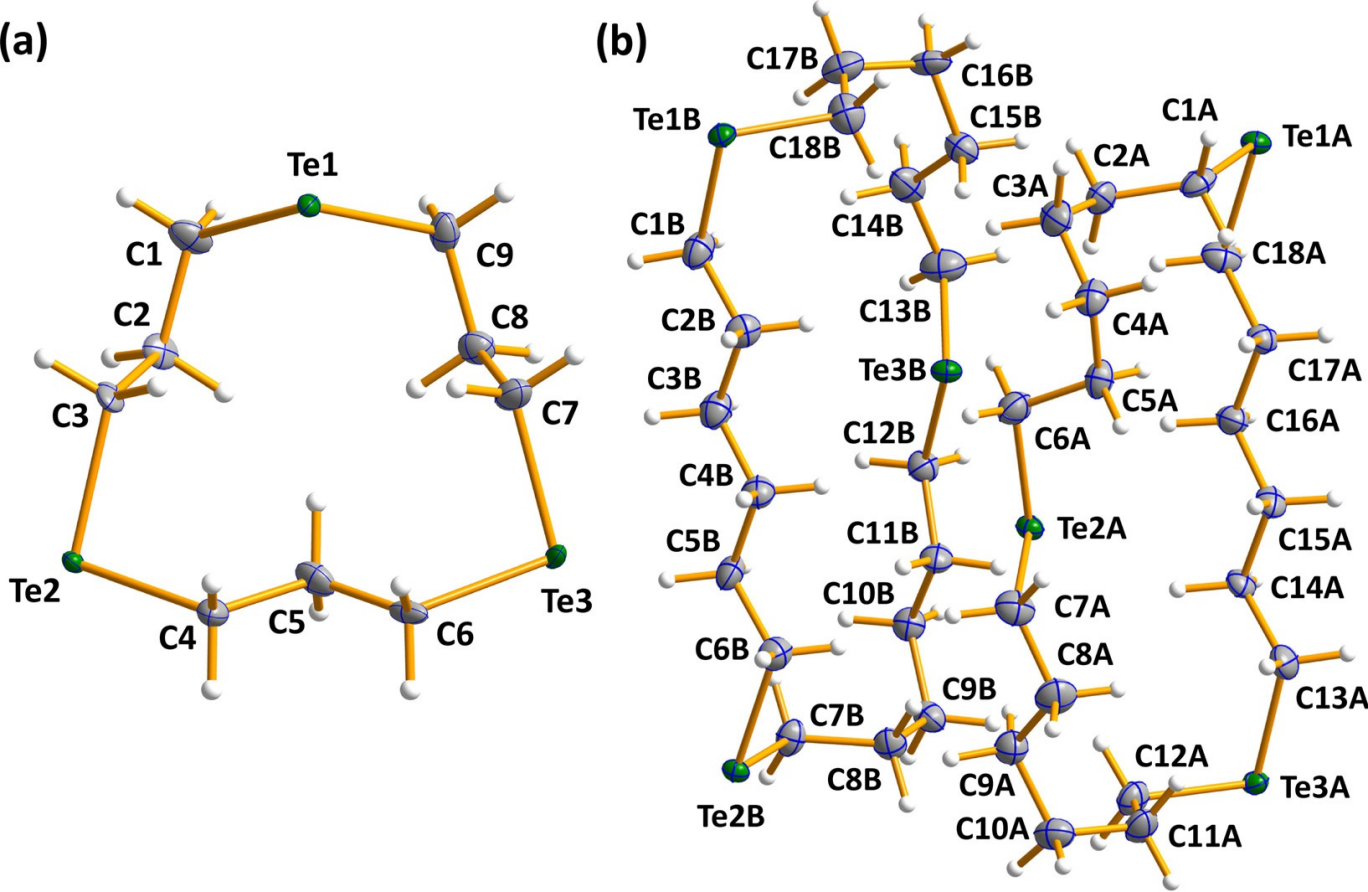
Molecular structures of a) 1,5,9‐Te_3_(CH_2_)_9_ and b) 1,8,15‐Te_3_(CH_2_)_18_. The anisotropic displacement parameters are shown at 50 % probability. Selected bond lengths and angles are listed in Table S3 in the Supporting Information. Te is depicted in green and C in grey.

**Figure 4:**
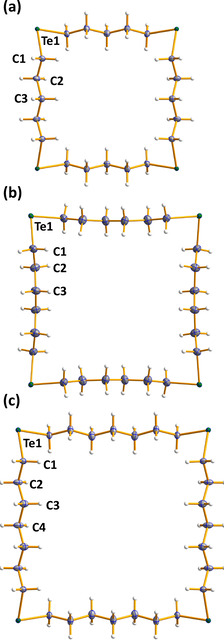
Molecular structures of a) 1,7,13,19‐Te_4_(CH_2_)_20_, b) 1,8,15,22‐Te_4_(CH_2_)_24_, and c) 1,9,17,25‐Te_4_(CH_2_)_28_. The anisotropic displacement parameters are shown at 50 % probability. Selected bond lengths and angles are listed in Table S3 in the Supporting Information. Te is depicted in green and C in grey.

Single crystals suitable for XRD were grown from dichloromethane/hexane in the case of 1,7‐Te_2_(CH_2_)_10_, 1,7,13,19‐Te_4_(CH_2_)_20_, 1,8,15,22‐Te_4_(CH_2_)_24_ and 1,9,17,25‐Te_4_(CH_2_)_28_, from chloroform/hexane in the case of 1,8‐Te_2_(CH_2_)_12_ and 1,5,9‐Te_3_(CH_2_)_9_, and from pentane in the case of 1,8,15‐Te_3_(CH_2_)_18_. Their molecular structures are shown in Figures 2, 3, 4.

All bond parameters are normal for C−Te and C−C single bonds [av 2.158(11) and 1.525(19) Å, respectively]. The structure of 1,7‐Te_2_(CH_2_)_10_ is disordered with two molecules randomly assuming two different orientations (see Figure 2 a). The ring molecules are formed from individual atoms, as shown below: 
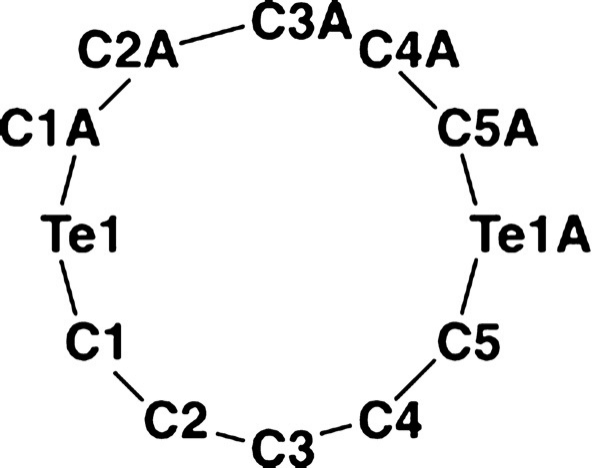


The second interspliced disordered pair is formed by symmetry (operation 1−*x*, 1−*y*, 1−*z*; see Figure 2 a). The site occupation factors of both disordered molecules are therefore 0.5.

### Intermolecular interactions

Each tellurium macrocycle is packed into supramolecular networks through Te**⋅⋅⋅**Te chalcogen bonds. The nature of these close contacts was explored by solid‐state PBE0‐D3/pob‐TZVP computations with periodic boundary conditions. The calculations were performed on 1,7‐Te_2_(CH_2_)_10_, 1,8‐Te_2_(CH_2_)_12_, 1,5,9‐Te_3_(CH_2_)_9_, 1,8,15‐Te_3_(CH_2_)_18_, 1,7,13,19‐Te_4_(CH_2_)_20_, 1,8,15,22‐Te_4_(CH_2_)_24_ and 1,9,17,25‐Te_4_(CH_2_)_28_, and in each case they yielded bond parameters and intermolecular contacts that were in good agreement with those observed in the crystal structures (see Table 3).

**Table 3:** Intramolecular Te−C bond lengths and intermolecular Te**⋅⋅⋅**Te contacts [Å] in optimized[Te(CH_2_)_*m*_]_*n*_ structures together with BCP densities *ρ*_BCP_ for Te**⋅⋅⋅**Te contacts, their calculated bond orders (BO), and estimated intermolecular interaction energies Δ*E*.

Compound	*d*(Te−C)	*d*(Te**⋅⋅⋅**Te)	No. of Te**⋅⋅⋅**Te contacts^[a]^	*ρ*_BCP_	BO^[b]^	Δ*E* [kJ mol^−1^]^[c]^
1,7‐Te_2_(CH_2_)_10_	2.151/2.152/2.152/2.156	3.686^[d]^	2	0.012	0.17	−164
	2.147/2.149/2.152/2.155	3.907^[d]^	2	0.008	0.11	−149
1,8‐Te_2_(CH_2_)_12_	2.148/2.155	3.876	2	0.010	0.14	−174
1,5,9‐Te_3_(CH_2_)_9_	Te1 2.152/2.160	–				−164
	Te2 2.152/2.159	3.794/3.743	5	0.011/0.010	0.14–0.16	
	Te3 2.151/2.152	3.743		0.010	0.14	
1,8,15‐Te_3_(CH_2_)_18_	Te1 2.154/2.155	3.933	5	0.009	0.13	−235
	Te2 2.147/2.154	3.842		0.010	0.14	
	Te3 2.158/2.163	3.922		0.009	0.13	
1,7,13,19‐Te_4_(CH_2_)_20_	2.156	3.847	16	0.010	0.14	−309
1,8,15,22‐Te_4_(CH_2_)_24_	2.155	3.882	16	0.010	0.14	−314
1,9,17,25‐Te_4_(CH_2_)_28_	2.156	3.859	16	0.010	0.14	−368

**Figure 5:**
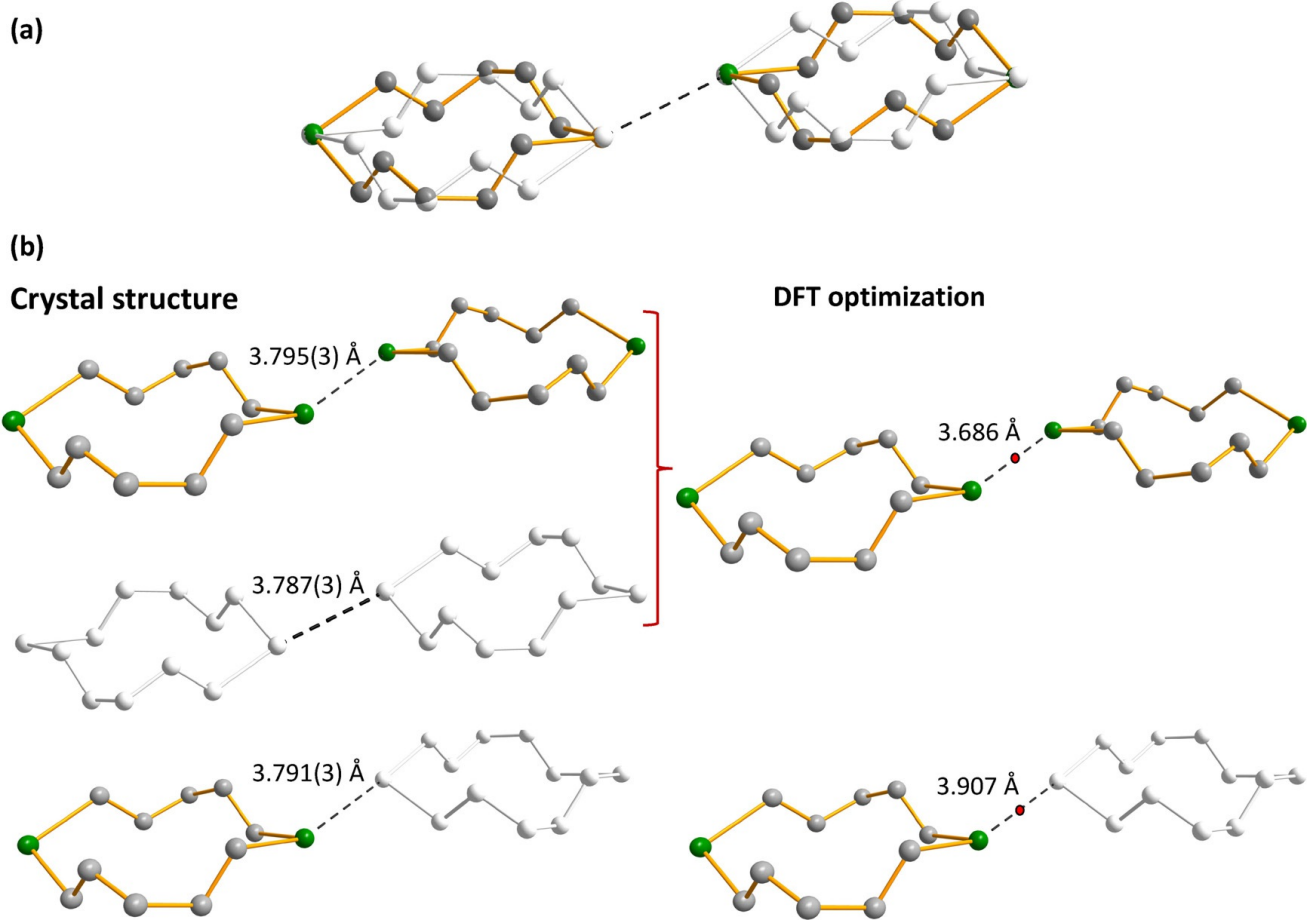
Comparison of the close contacts in 1,7‐Te_2_(CH_2_)_10_ molecules between the experimental crystal structures and the solid‐state PBE0‐D3/pob‐TZVP calculations involving periodic boundary conditions. The hydrogen atoms have been omitted for clarity. a) Disordered crystal structure. b) The interactions between the alternative orientations of the neighbouring molecules with intermolecular distances between the tellurium atoms. The red dots in the PBE0‐D3/pob‐TZVP optimized structures indicate the locations of the BCPs. The electron densities at the BPCs are listed in Table 3.

[a] Number of Te**⋅⋅⋅**Te close contacts from one molecule. [b] The PBE0‐D3/pob‐TZVP bond orders were calculated by comparison of the electron densities at BCPs to the corresponding values in single bonds.^[9]^ [c] The stabilization of the solid lattice was estimated by calculation of the difference of the energies in the lattice per molecule and the discrete molecules in vacuum. No thermal corrections to energies were made. [d] For the relative orientations of the disordered molecules, see Figure 5.

Te**⋅⋅⋅**Te interactions in the disordered structure of 1,7‐Te_2_(CH_2_)_10_ can lead to four different alternatives depending on the mutual orientations of the disordered molecules (Figure 5). The Te**⋅⋅⋅**Te interactions link the individual molecules into infinite chains.

**Figure 6:**
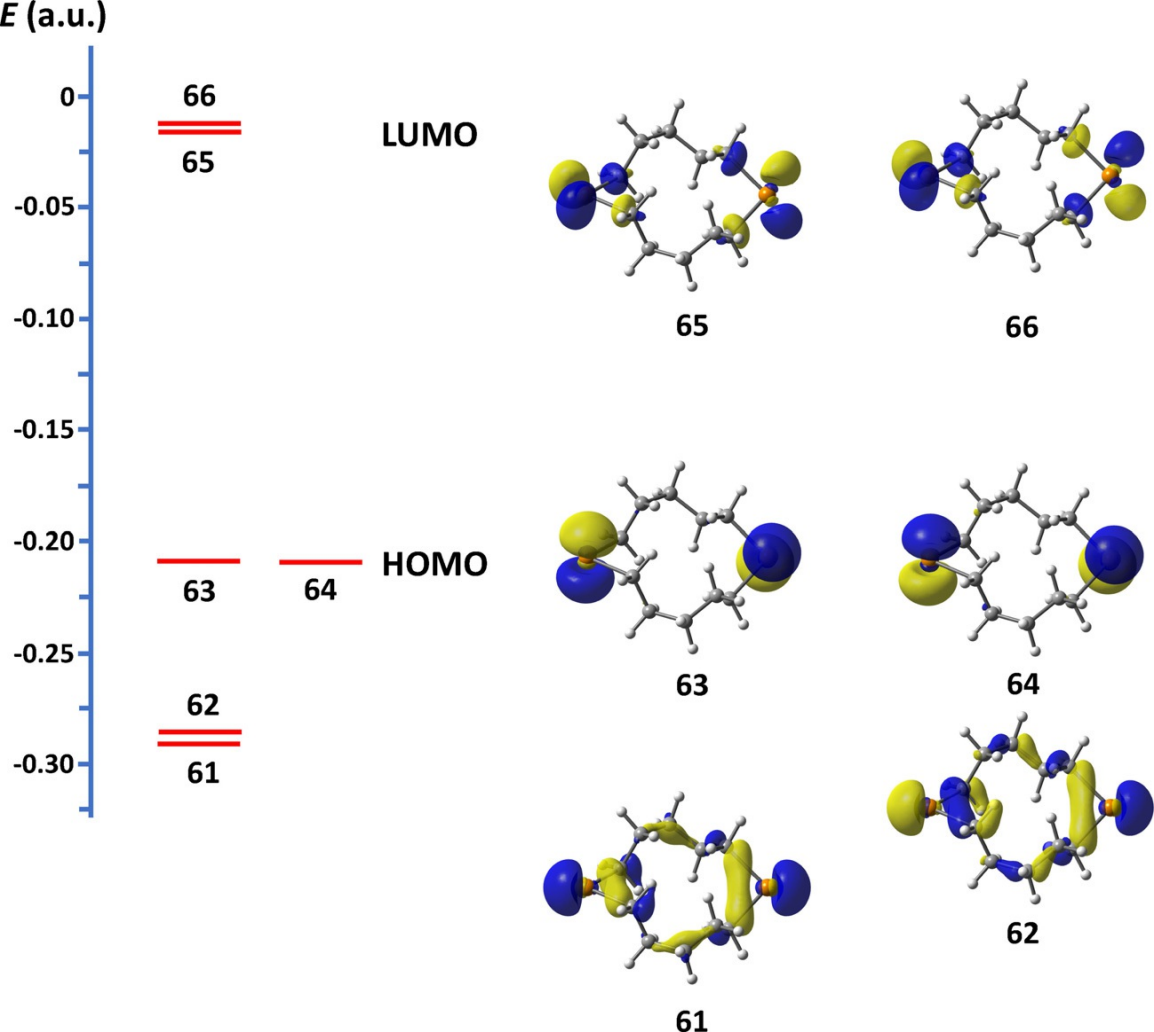
The frontier orbitals of 1,7‐Te_2_(CH_2_)_10_.

The frontier orbitals of 1,7‐Te_2_(CH_2_)_10_ together with orbital energies are depicted in Figure 6. The covalent interactions in the short Te**⋅⋅⋅**Te contacts can be qualitatively explained by HOMO–LUMO overlap and consequent charge transfer, as indicated by the PBE0‐D3/def2‐TZVPP calculations. The overlap is maximized when the mutual orientation of the molecules corresponds to that observed in the crystalline lattice.

The C_2_Te planes of the disordered neighbouring molecules show an angle of 0 or 47° (see Figure 5). The latter mutual orientation provides for favourable frontier orbital overlap. The orientations of the molecules also coincide with the direction of σ‐holes on tellurium atoms, and bonding could also be explained by the σ‐hole interaction/mutual polarization model.^[7]^ Following symmetry‐adapted perturbation theory results by Gleiter et al.^[6e]^ on R_1_R_2_Te**⋅⋅⋅**TeR_1_R_2_ (R_1_, R_2_=Me, CN) model systems, the largest contributions to Te**⋅⋅⋅**Te chalcogen bonds in these macrocycles are expected to come from dispersion and induction energy terms with smaller contributions from electrostatic interactions.

**Figure 7:**
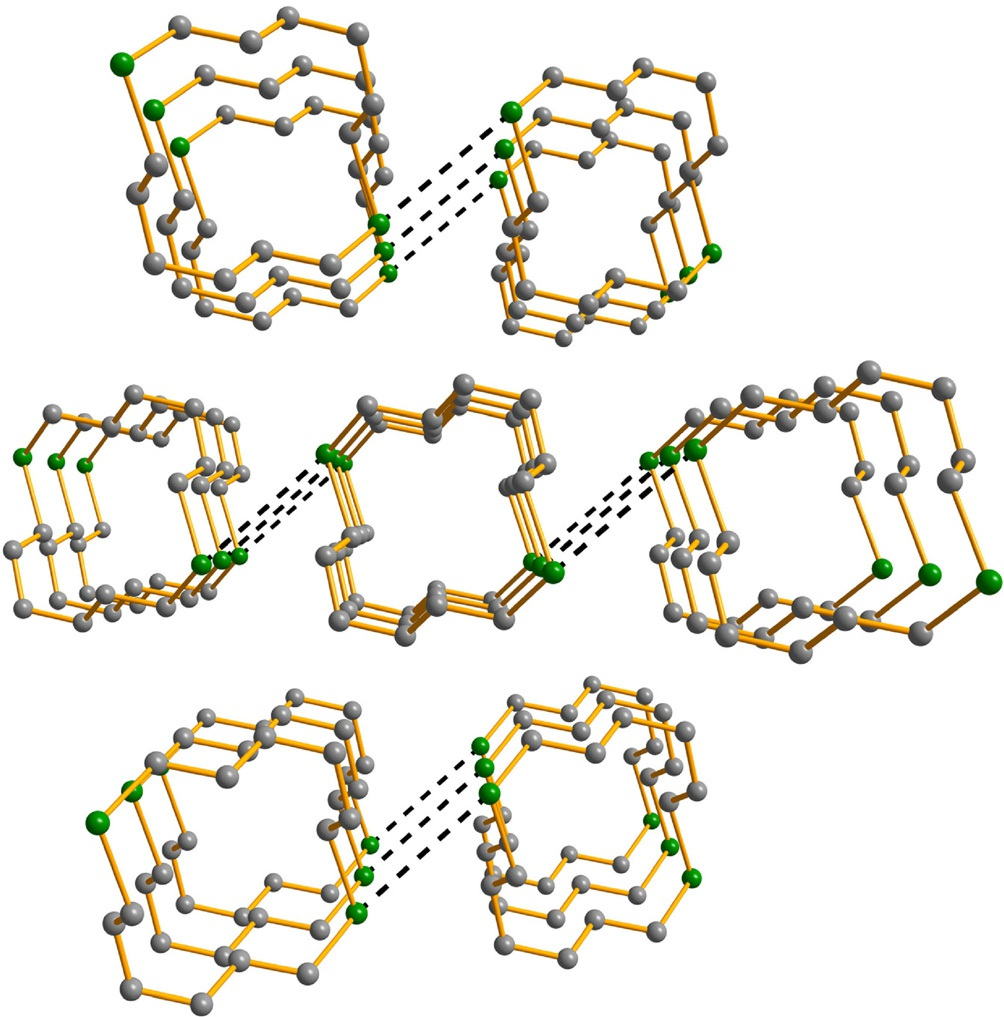
Tubular stacking of the 1,8‐Te_2_(CH_2_)_12_ molecules in the crystal lattice. The hydrogen atoms have been omitted for clarity. Te is depicted in green and C in grey.

1,8‐Te_2_(CH_2_)_12_ shows quite similar Te**⋅⋅⋅**Te interactions, and the molecules are also stacked into infinite chains with Te**⋅⋅⋅**Te distance of 4.0390(3) Å. The chains are linked together only by weak van der Waals interactions. Interestingly, as a consequence of the Te**⋅⋅⋅**Te interactions, the molecules in both 1,7‐Te_2_(CH_2_)_10_ and 1,8‐Te_2_(CH_2_)_12_ form infinite tubular columns, as exemplified for 1,8‐Te_2_(CH_2_)_12_ in Figure 7. Tubular stacking has been inferred earlier to also be a general feature of macrocyclic thio‐ and selenoethers.^[6]^

**Figure 8:**
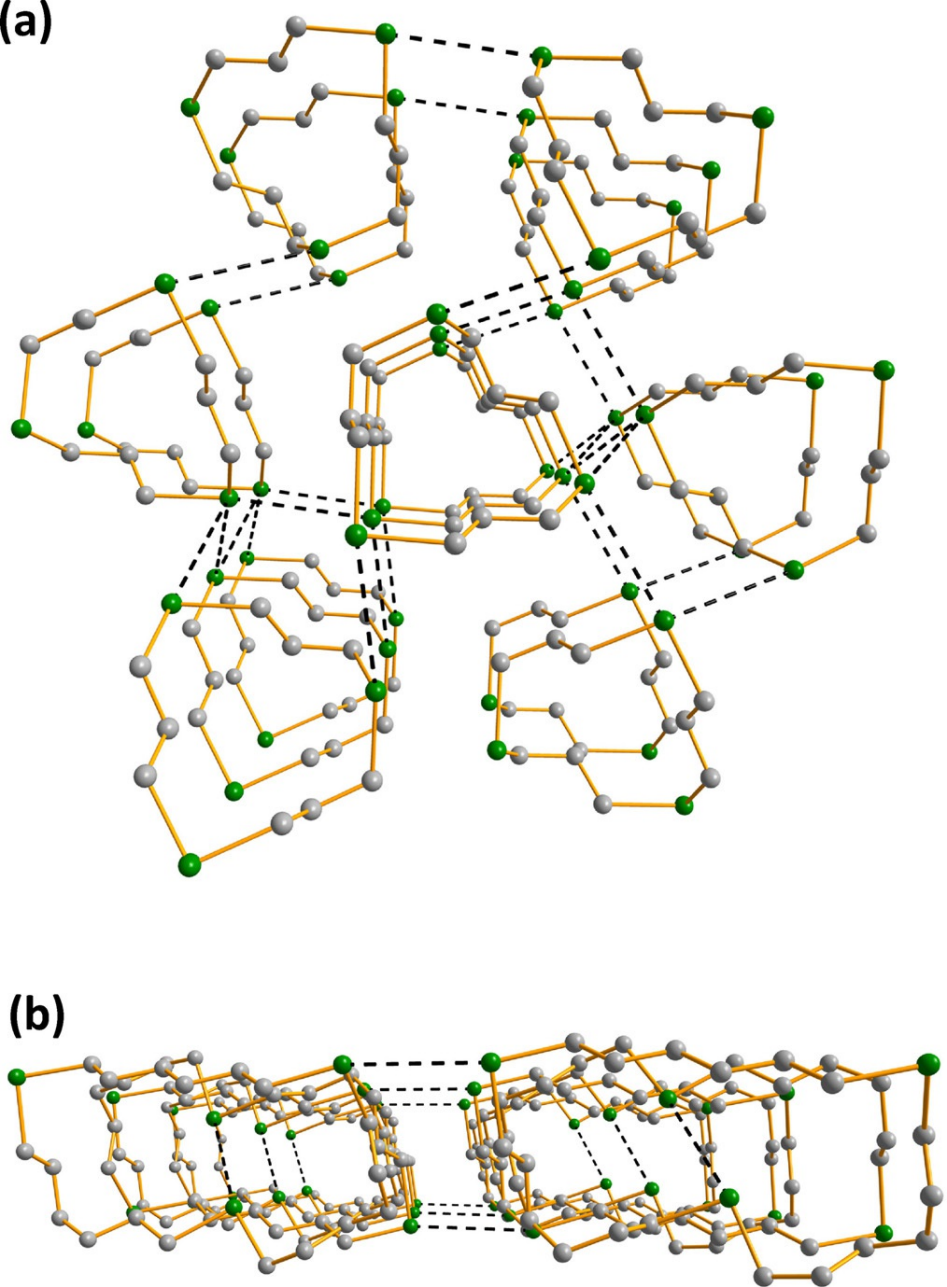
The tubular stacking of molecules of a) 1,5,9‐Te_3_(CH_2_)_9_ and b) 1,8,15‐Te_3_(CH_2_)_18_ in the crystalline lattice. The hydrogen atoms have been omitted for clarity. Te is depicted in green and C in grey.

As the number of tellurium atoms in the macrocycles and the number of Te**⋅⋅⋅**Te contacts increases, the supramolecular lattices become more complex. This is illustrated in Figure 8 by the solid‐state lattices of 1,5,9‐Te_3_(CH_2_)_9_ and 1,8,15‐Te_3_(CH_2_)_18_. The molecules are again stacked in a tubular fashion with Te**⋅⋅⋅**Te close contacts spanning the range of 3.8044(2)–4.0557(2) Å.

**Figure 9:**
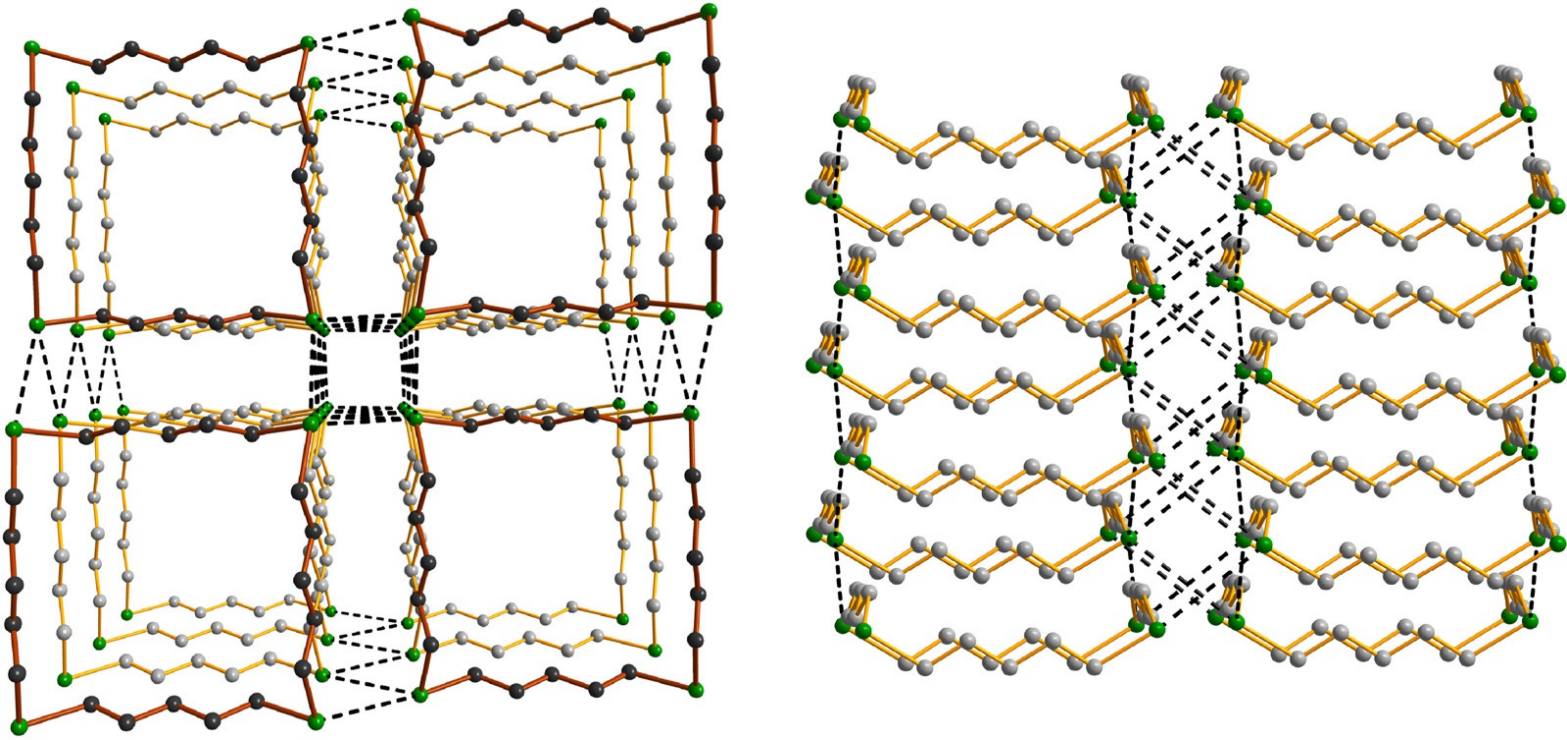
The Te**⋅⋅⋅**Te close contacts form infinite rectangular shafts, as illustrated for 1,7,13,19‐Te_4_(CH_2_)_20_ in two perpendicular views. The hydrogen atoms have been omitted for clarity. Te is depicted in green and C in grey.

Perhaps the most interesting supramolecular lattices are formed by 1,7,13,19‐Te_4_(CH_2_)_20_, 1,8,15,22‐Te_4_(CH_2_)_24_ and 1,9,17,25‐Te_4_(CH_2_)_28_, which are also aesthetically pleasing. The tellurium atoms in each molecule are involved in a rectangular packing arrangement and form infinite shafts, as shown in Figure 9 for 1,7,13,19‐Te_4_(CH_2_)_20_. Visually similar arrangements involving (>Te**⋅⋅⋅**Te<)_4_ interactions are found also in the solid‐state lattice of acyclic MeTe{C≡C}_*n*_TeMe (*n=*2–4).^[11b]^

**Figure 10:**
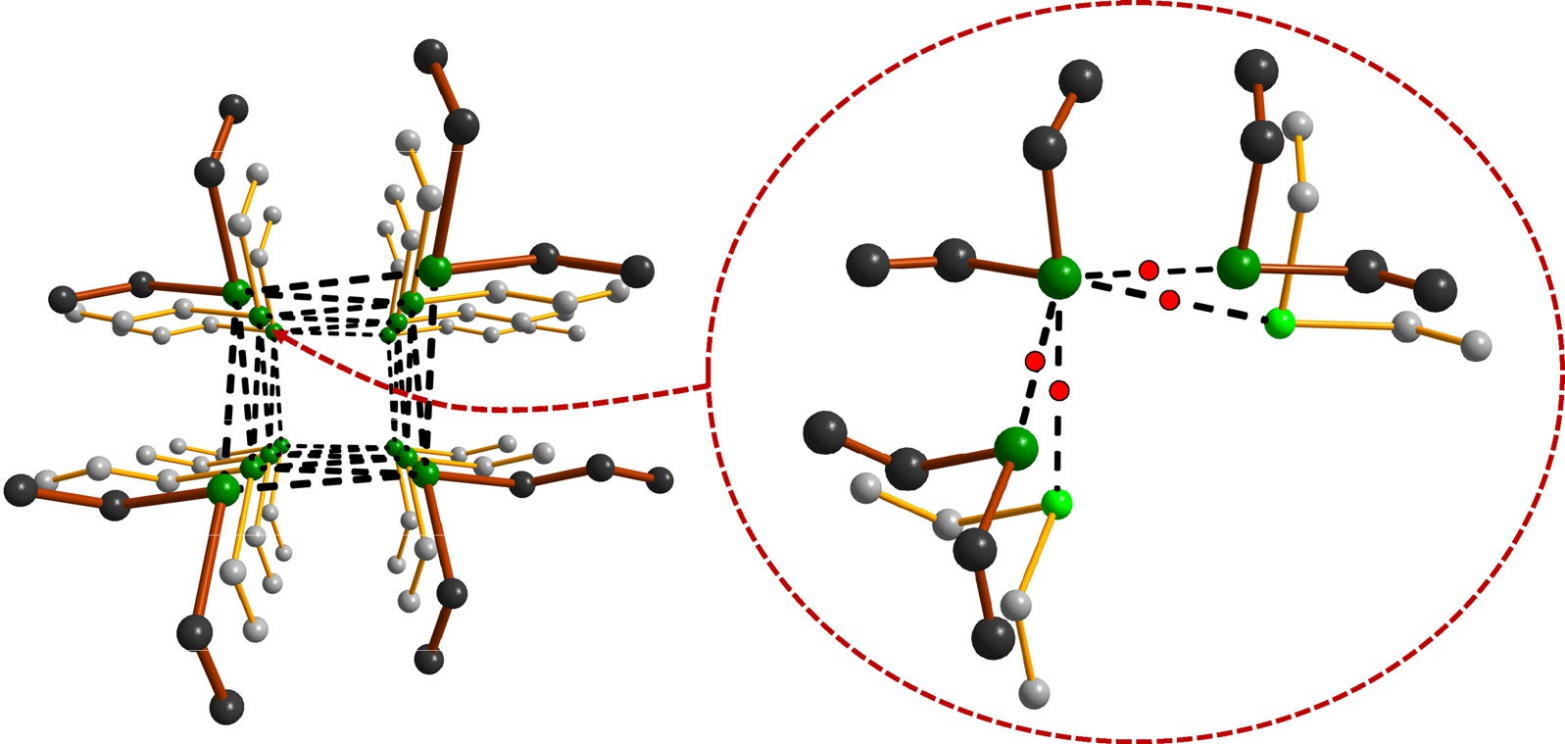
Details of the molecular packing in 1,7,13,19‐Te_4_(CH_2_)_20_ showing the expansion of the coordination environment around tellurium from two to six by four chalcogen bonds. The red dots represent the locations of BCPs at the PBE0‐D3/pob‐TZVP level of theory from solid‐state calculations involving periodic boundary conditions (see Table 3). The hydrogen atoms have been omitted for clarity. Te is depicted in green and C in grey.

Closer inspection of the Te**⋅⋅⋅**Te interactions in 1,7,13,19‐Te_4_(CH_2_)_20_, 1,8,15,22‐Te_4_(CH_2_)_24_ and 1,9,17,25‐Te_4_(CH_2_)_28_ shows that the coordination of each tellurium atom is expanded from two to six by four SBIs to give a quasi‐octahedral coordination sphere (see Figure 10). The Te**⋅⋅⋅**Te close contacts in the three species span 4.047(2)–4.066(2) Å for 1,7,13,19‐Te_4_(CH_2_)_20_, 1,8,15,22‐Te_4_(CH_2_)_24_ and 1,9,17,25‐Te_4_(CH_2_)_28_.

The nature and strength of the Te**⋅⋅⋅**Te interactions can be explored by means of electron densities at bond critical points (BCPs) from solid‐state PBE0‐D3/pob‐TZVP calculations. Bond orders of the Te**⋅⋅⋅**Te chalcogen bonds estimated from the BCP electron densities span a narrow range of 0.11–0.17 (see Table 3). The bond orders are well in line with those computed earlier for Te**⋅⋅⋅**Te−C chalcogen bonds in [Fe(C_5_H_4_E)_2_E′] (E, E′=S, Se, Te).^[9]^ The infinite shafts in 1,7,13,19‐Te_4_(CH_2_)_20_, 1,8,15,22‐Te_4_(CH_2_)_24_ and 1,9,17,25‐Te_4_(CH_2_)_28_ result in a larger number of SBIs compared to other [Te(CH_2_)_*m*_]_*n*_ molecules. This is reflected by the higher intermolecular interaction energies calculated for 1,7,13,19‐Te_4_(CH_2_)_20_, 1,8,15,22‐Te_4_(CH_2_)_24_, and 1,9,17,25‐Te_4_(CH_2_)_28_ (see Table 3), which in turn suggest greater stabilization of lattices for species containing four tellurium atoms.

**Figure 11:**
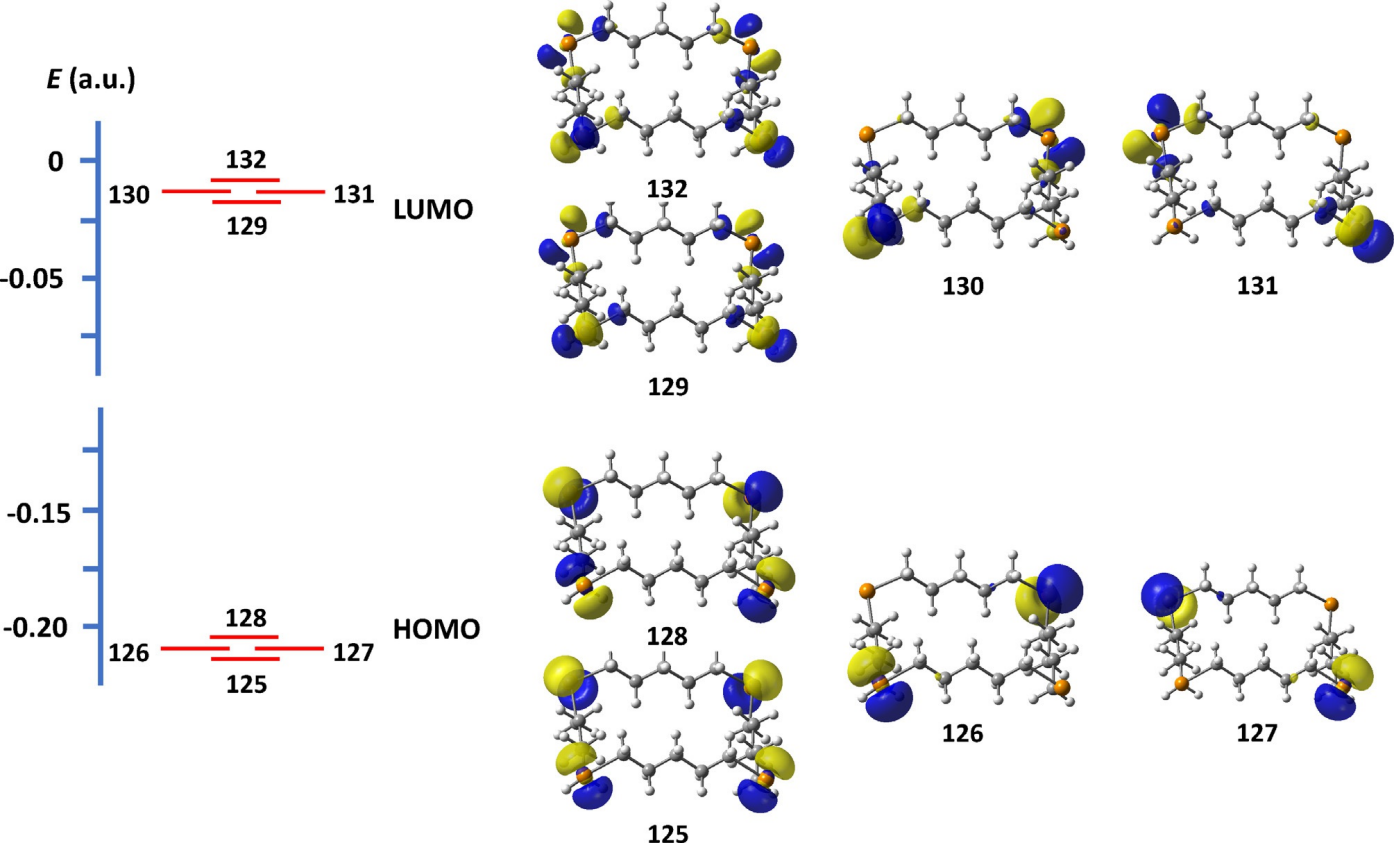
Frontier orbitals of 1,7,13,19‐Te_4_(CH_2_)_20_ computed at the PBE0‐D3/def2‐TZVPP level of theory.

The intermolecular close contacts and the formation of the shafts can be rationalized by frontier orbital overlap and charge transfer. The frontier orbitals of 1,7,13,19‐Te_4_(CH_2_)_20_ are shown in Figure 11 and serve as examples for all ring molecules containing four tellurium atoms.

**Figure 12:**
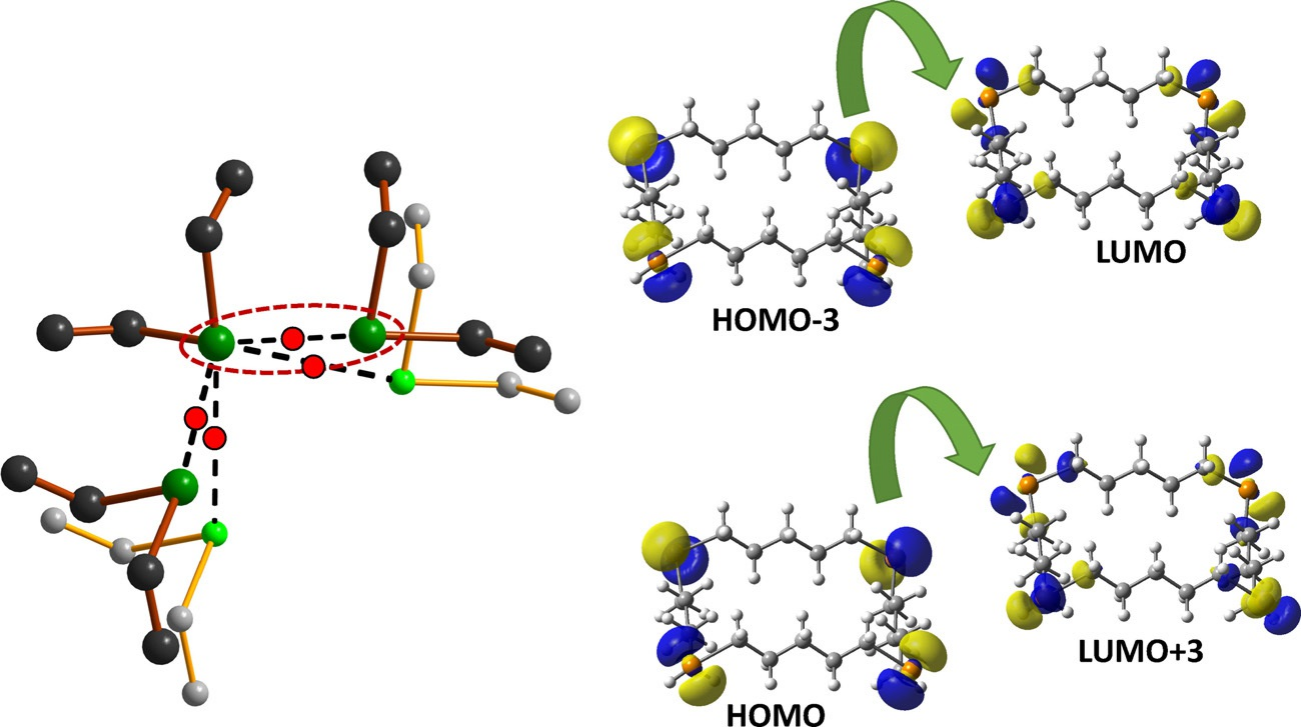
The PBE0‐D3/def2‐TZVPP HOMO‐3→LUMO and HOMO→LUMO+3 overlap and charge transfer in the intermolecular interaction indicated by the red slashed oval. The three other interactions are created by symmetry. The PBE0‐D3/pob‐TZVP electron density at the BCP calculated for the solid lattice (indicated in red) is 0.010 e Å^−3^ corresponding to the bond order of 0.14.

The orientation of the molecules in the crystalline lattice is consistent with the overlap and charge transfer between both HOMO−3→LUMO and HOMO→LUMO+3, as shown in Figure 12. These frontier orbitals are mostly non‐bonding with respect to any intramolecular bonds, and therefore their interactions do not have significant effects on the Te−C or C−C bond lengths.

**Figure 13:**
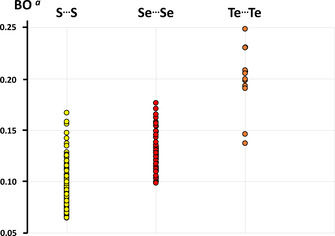
Pauling bond orders^[19]^ of intermolecular chalcogen–chalcogen interactions in cyclic unsaturated chalcogenoethers.[^10^, ^11^] [a] The relationship between the interatomic contact and the corresponding bond order (BO) is defined as follows: $BO=10^{-\left(r-r_{0}\right)/0.71}$ , where *r*=the interatomic distance and *r*_0_=single‐bond length of the atoms in question.

A comparison of the S**⋅⋅⋅**Se, Se**⋅⋅⋅**Se, and Te**⋅⋅⋅**Te contacts in related thio‐, seleno‐ and telluroether macrocycles and some related acyclic chalcogenoethers is given in Table S4 of the Supporting Information. A comparison of Pauling bond orders^[19]^ based on these distances is shown in Figure 13. The PBE0‐D3/pob‐TZVP bond orders listed in Table 3 for the species considered in this contribution are in good agreement with bond orders estimated by the Pauling method.

Figure 13 shows that, as expected, the chalcogen bonds become stronger, when moving down the periodic group, as has been concluded previously.[^6^, ^9^] This trend is well in accord with that inferred earlier for the trichalcogenaferrocenophanes.^[9]^

### Porosity

Because of the tubular packing of 1,7,13,19‐Te_4_(CH_2_)_20_, 1,8,15,22‐Te_4_(CH_2_)_24_ and 1,9,17,25‐Te_4_(CH_2_)_28_, the crystal structure determination indicated a significant void fraction in the crystal lattices (17, 20, and 27 %, respectively). Gleiter and co‐workers,[^10c^, ^10j^, ^11b^] have reported several related chalcogenoethers, which contain small molecules inside the tubes. 1,7,13,19‐Te_4_(CH_2_)_20_, 1,8,15,22‐Te_4_(CH_2_)_24_ and 1,9,17,25‐Te_4_(CH_2_)_28_ were crystallized from hexane/dichloromethane, and some electron density due to solvent molecules was also indicated in the tubular channels. Gleiter et al. have also observed that the solvent molecules in the solvent‐accessible void are disordered on crystallization from hexane/dichloromethane.^[6a]^ In case of aromatic solvents, the molecules tend to be fully ordered in the crystal lattice.

**Figure 14:**
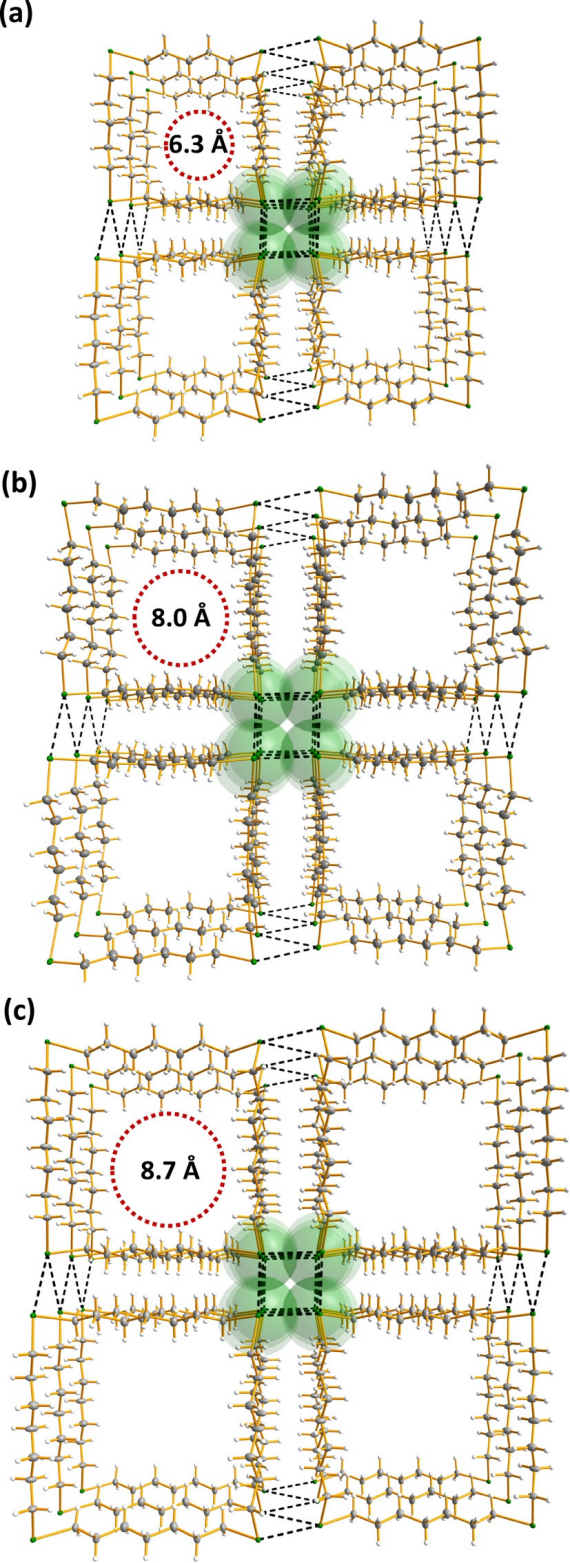
The pore openings in a) 1,7,13,19‐Te_4_(CH_2_)_20_, b) 1,8,15,22‐Te_4_(CH_2_)_24_ and c) 1,9,17,25‐Te_4_(CH_2_)_28_. Te is depicted in green and C in grey.

To further gauge the porosity of these materials the voids of 1,8,15,22‐Te_4_(CH_2_)_24_ were explored by BET analysis^[20]^ with N_2_ as the adsorptive gas, which indicated a surface area of 952 m^2^ g^−1^. A pore size distribution analysis using the slit pore, non‐local density functional theory (NLDFT) equilibrium model gave a sharp value for the pore diameter of 7.2 Å. This compares well with the values of 8.0 Å (edge) and 10.2 Å (diagonal) calculated for the square pore opening on the basis of the van der Waals radii (see Figure 14). The size of the pore opening is well controlled by changing the length of the aliphatic group.

## Conclusion

A series of heterocyclic telluroethers [Te(CH_2_)_*m*_]_*n*_ (*n=*1–4; *m=*3–7) has been prepared by the reaction of Na_2_Te with α,ω‐bromoalkanes Br(CH_2_)_*m*_Br. In most cases mixtures of telluroethers were obtained. They were separated by preparative column chromatography. All species formed in the reactions were characterized by EIMS and ^1^H, ^13^C and ^125^Te NMR spectroscopy, and in some cases by elemental analysis. The crystal structures of 1,7‐Te_2_(CH_2_)_10_, 1,8‐Te_2_(CH_2_)_12_, 1,5,9‐Te_3_(CH_2_)_9_, 1,8,15‐Te_3_(CH_2_)_18_, 1,7,13,19‐Te_4_(CH_2_)_20_, 1,8,15,22‐Te_4_(CH_2_)_24_ and 1,9,17,25‐Te_4_(CH_2_)_28_ indicated that Te**⋅⋅⋅**Te chalcogen bonds play an important role in the self‐assembly of the molecules in the crystals. In most structures, the rings pack to form infinite tubes. The most interesting supramolecular lattices are formed by 1,7,13,19‐Te_4_(CH_2_)_20_, 1,8,15,22‐Te_4_(CH_2_)_24_ and 1,9,17,25‐Te_4_(CH_2_)_28_. In all these compounds, the Te**⋅⋅⋅**Te close contacts form infinite square shafts in which the bonding environment of each tellurium atom is expanded from two to six forming quasi‐octahedral coordination spheres. QTAIM calculations showed that the Te**⋅⋅⋅**Te chalcogen bond orders in these shafts are approximately 0.14. The relative orientations of the molecules in the solid lattices are attributable to HOMO–LUMO interactions and charge transfer between the molecules.

## Experimental Section

### General procedures

All manipulations involving air‐ and moisture‐sensitive materials were conducted under a nitrogen atmosphere by using Schlenk techniques. Chloroform and dichloromethane were distilled over CaH_2_ and hexane over Na/benzophenone under a nitrogen atmosphere prior to use. Ethanol was degassed by bubbling nitrogen through the solvent for at least 15 min. Semiconductor‐grade tellurium was freshly ground. All other reagents were used as purchased without further purification.

### NMR spectroscopy

^1^H, ^13^C and ^125^Te NMR spectra were recorded in CDCl_3_ with a Bruker Avance III 400 spectrometer operating at 400.13, 100.61 and 126.24 MHz, respectively. The respective pulse widths were 13.0, 9.70 and 6.0 μs, and the corresponding relaxation delay was 2.0 s for each nucleus. The deuterated solvent was used as the ^2^H lock. All resonances were referenced by indirect referencing using the deuterium signal of the solvents for a lock to the frequency that relates to the resonance frequency of the TMS protons of exactly 400.130 000 MHz. The tellurium resonance *v*_0_(Te) was calculated by using the ratio *Ξ*=*v*_0_(Te)*/v*_0_(TMS)=31.549 769 % as recommended by IUPAC.^[21]^ The ^1^H and ^13^C chemical shifts are reported relative to TMS [*δ*_H_=*δ*_TMS_(H)−7.26; *δ*_C_=*δ*_TMS_(C)−77.16],^[22]^ and the ^125^Te chemical shifts relative to Me_2_Te.

### Mass spectrometry

EI mass spectra were recorded with Finnigan MAT SSQ 710 and Finnigan MAZ95XL spectrometers. The energy of the electrons was 70 eV.

### X‐ray crystallography

The crystals of 1,7‐Te_2_(CH_2_)_10_, 1,8‐Te_2_(CH_2_)_12_, 1,5,9‐Te_3_(CH_2_)_9_, 1,8,15‐Te_3_(CH_2_)_18_, 1,7,13,19‐Te_4_(CH_2_)_20_, 1,8,15,22‐Te_4_(CH_2_)_24_, and 1,9,17,25‐Te_4_(CH_2_)_28_ were coated with Paratone oil and mounted in a nylon CryoLoop, and the intensity data were collected with a Bruker Nonius Kappa CCD diffractometer at 133 K by using graphite‐monochromated Mo_Kα_ radiation (*λ*=0.71073 Å; 55 kV, 25 mA).^[23]^ Crystal data and the details of structure determinations are given in Table S2 in the Supporting Information. The data were corrected for Lorentzian and polarization effects, after which semi‐empirical absorption correction was applied to net intensities by using SADDABS.^[24]^

The structures were solved by direct methods with SHELXS‐2016 and refined with SHELXL‐2016.^[25]^ After the full‐matrix least‐squares refinement of the non‐hydrogen atoms with anisotropic thermal parameters, the hydrogen atoms were placed in calculated positions in the CH_2_ groups (C−H=0.99 Å). In the final refinement the hydrogen atoms were riding with the carbon atoms to which they are bonded. The isotropic thermal parameters of the hydrogen atoms were fixed at 1.5 times that of the corresponding carbon atoms. The scattering factors for the neutral atoms were those incorporated with the program.

The crystals of 1,7‐Te_2_(CH_2_)_10_ were found to be disordered and the molecule assumed two orientations around the symmetry element. Because of the symmetry constraints, the site occupation factors of the two ring molecules were exactly 0.5.

1,7,13,19‐Te_4_(CH_2_)_20_, 1,8,15,22‐Te_4_(CH_2_)_24_ and 1,9,17,25‐Te_4_(CH_2_)_28_ were crystallized from hexane/dichloromethane (see Table S2 in Supporting Information). Their structures all contain large voids, filled with disordered solvent molecules. The sizes of the voids are 125, 172 and 280 Å^3^ per unit cell, respectively. Their contribution to the structure factors was secured by back‐Fourier transformation by using the SQUEEZE routine of the program PLATON,^[26]^ resulting in 26, 18 and 35 electrons per unit cell, respectively.

Deposition Number(s) 198247, 198248, 198249, 198250, 19998251, and 198252 contain the supplementary crystallographic data for this paper. These data are provided free of charge by the joint Cambridge Crystallographic Data Centre and Fachinformationszentrum Karlsruhe Access Structures service www.ccdc.cam.ac.uk/structures.

### Surface area measurements

Surface area measurements were conducted with a Quantachrome Autosorb iQ system 16 with nitrogen as adsorptive. The Autosorb‐1 software was used in the calculations.

### Reactions of α,ω‐Br_2_(CH_2_)_*m*_ (*m=*3–7) with Na_2_Te

An excess of powdered sodium borohydride (1–3 g, 29–86 mmol) was slowly added over several hours in small portions of up to 200 mg to a suspension of finely powdered elemental tellurium (1 g, 7.84 mmol) in degassed ethanol (40 mL) under a nitrogen atmosphere and stirred at 90 °C. The reaction mixture turned from colourless to slightly purple to deeply purple and finally to deep red due to the build‐up of larger polytelluride ions. The addition of sodium borohydride was continued until all elemental tellurium had reacted and the solution remained colourless. Cooling to room temperature yielded colourless slurries, to which 7.84 mmol of the appropriate α,ω‐dibromoalkane dissolved in 10–25 mL of ethanol, was added over 12–30 min under exclusion of light. The mixtures were stirred for at least 15 h. Product separation was achieved by preparative column chromatography (silica) in all cases. Combined yields of 11–44 % were achieved, depending on the α,ω‐dibromoalkane. The actual amounts of reagents, the workup procedures used in the different reactions and the yields of isolated compounds are given in the Supporting Information.

### Computational details

Solid‐state structures of telluroether molecules were optimized by periodic‐boundary DFT calculations in the Crystal17 program package^[27]^ by employing the PBE0 hybrid functional^[28]^ and applying triple‐zeta valence basis set pob‐TZVP^[29]^ designed for solid‐state calculations for carbon and hydrogen while using a modified basis set given in ref. [30] for tellurium. Dispersion interactions in the solid state were treated with the Becke–Johnson damped version of Grimme's D3 model (PBE0‐D3/pob‐TZVP),^[31]^ as implemented in Crystal17. k‐Points within the Brillouin zone of the reciprocal space were sampled according to the Monkhorst–Pack method by employing a shrinking factor of 8 for reciprocal lattice vectors to generate a basis for diagonalizing the Hamiltonian matrix. Coulomb and exchange integrals were evaluated by using default tolerance factors of 7,7,7,7, and 14. Default SCF convergence and optimization thresholds were used for optimizations. The intermolecular interactions were analysed by topological analysis of the electron density^[32]^ carried out with the TOPOND module^[33]^ of the Crystal17 package. For TOPOND calculations the basis sets were modified by removing the f functions from tellurium. Lattice enthalpy and energy terms for Na_2_Te(c) and NaBr(c) were derived from frequency calculations of their optimized solid‐state structures (their experimental crystal structures are reported in ref. [34]).

For molecular orbital analyses and reaction energy calculations, molecular structures were optimized by DFT level with the ORCA program package,^[35]^ PBE0 functional,^[28]^ def2‐TZVPP basis set,^[36]^ and def2/JK auxiliary basis sets^[37]^ to speed up the calculation of the HF exchange term. Dispersion forces were treated by using the Becke–Johnson D3 model available in ORCA (PBE0‐D3/def2‐TZVPP).^[31]^ The effect of solvent on molecules in ethanol solutions were modelled with the conductor‐like polarizable continuum model (CPCM).^[38]^ Calculated energies are given in Tables S6 and S7 in the Supporting Information.

## Conflict of interest

The authors declare no conflict of interest.

## Supporting information

As a service to our authors and readers, this journal provides supporting information supplied by the authors. Such materials are peer reviewed and may be re‐organized for online delivery, but are not copy‐edited or typeset. Technical support issues arising from supporting information (other than missing files) should be addressed to the authors.

Supplementary
