# Supplementary material for: Chalcogen‐Bonding Interactions in Telluroether Heterocycles [Te(CH2)m]n (n=1–4; m=3–7)
Source: Chemistry. 2020 Oct 16;26(61):13806–18. doi: 10.1002/chem.202002510 (PMC7702089; doi:10.1002/chem.202002510)
Supplement: Supplementary file 1 — Supplementary [file CHEM-26-13806-s001.pdf]

Chemistry–A European Journal

Supporting Information

Chalcogen-Bonding Interactions in Telluroether Heterocycles $[Te(CH_2)_m]_n$ ($n = 1–4$; $m = 3–7$)

Marko Rodewald,[a] J. Mikko Rautiainen,[b] Tobias Niksch,[c] Helmar Görls,[a] Raija Oilunkaniemi,[d] Wolfgang Weigand,*[a] and Risto S. Laitinen*[d]

Table of Contents

Isolation of Reaction Products

Table S1. Preparative details of the $[Te(CH_2)_m]_n$ (n = 1-4; m = 3-7) ring molecules.
Figure S1. Thin layer chromatogram of the reaction of Na_2Te and $Br(CH_2)_6Br$.

Crystal Structure Determination of 1,7-$Te_2(CH_2)_{10}$, 1,8-$Te_2(CH_2)_{12}$, 1,5,9-$Te_3(CH_2)_9$, 1,8,15-$Te_3(CH_2)_{18}$, 1,7,13,19-$Te_4(CH_2)_{20}$, 1,8,15,22-$Te_4(CH_2)_{24}$, and 1,9,17,25-$Te_4(CH_2)_{28}$.

Table S2. Crystal data and details of structure determination
Table S3. Selected bond lengths (Å) and angles (°)
Table S4. The intermolecular chalcogen-chalcogen contacts (in Å) in some cyclic unsaturated chalcogenoethers and related open-chain species.

Mass spectra of $[Te(CH_2)_m]_n$ (n = 1-4; m = 3-7)

Figure S2. The mass spectra of the products of the reaction of (a) Na_2Te and $Br(CH_2)_3Br$ and (b) Na_2Te and $Br(CH_2)_4Br$.
Figure S3. The mass spectra of the products of the reaction of Na_2Te and $Br(CH_2)_5Br$.
Figure S4. The mass spectra of the products of the reaction of Na_2Te and $Br(CH_2)_6Br$.
Figure S5. The mass spectra of the products of the reaction of Na_2Te and $Br(CH_2)_7Br$.
Figure S6. The experimental and calculated isotopic distributions of the isolated products in the reaction of (a) Na_2Te and $Br(CH_2)_3Br$, and (b) Na_2Te and $Br(CH_2)_4Br$.
Figure S7. The experimental and calculated isotopic distributions of the isolated products in the reaction of Na_2Te and $Br(CH_2)_5Br$.
Figure S8. The experimental and calculated isotopic distributions of the isolated products in the reaction of Na_2Te and $Br(CH_2)_6Br$.
Figure S9. The experimental and calculated isotopic distributions of the isolated products in the reaction of Na_2Te and $Br(CH_2)_7Br$.

NMR Spectroscopic Information of $[Te(CH_2)_m]_n$ (n = 1-4; m = 3-7)

Table S5. Summary of the ^{1}H, ^{13}C, and ^{125}Te NMR spectroscopic information for the different $[Te(CH_2)_m]_n$ species. The data are recorded in $CDCl_3$ at 24 °C. (see Figures S10-S99 for individual NMR spectra).

Figure S10. ^{1}H-NMR spectrum of 1,5,9-$Te_3(CH_2)_9$.
Figure S11. $^{13}C\{1H\}$-NMR spectrum of 1,5,9-$Te_3(CH_2)_9$.
Figure S12. $^{125}Te\{^{1}H\}$-NMR spectrum of 1,5,9-$Te_3(CH_2)_9$.
Figure S13. ^{125}Te-NMR spectrum of 1,5,9-$Te_3(CH_2)_9$.
Figure S14. $^{1}H,^{13}C$-HSQC-NMR spectrum of 1,5,9-$Te_3(CH_2)_9$.
Figure S15. $^{1}H,^{125}Te$-HMBC-NMR spectrum of 1,5,9-$Te_3(CH_2)_9$.
Figure S16. ^{1}H-NMR spectrum of $Te(CH_2)_4$.
Figure S17. $^{13}C\{^{1}H\}$-NMR spectrum of $Te(CH_2)_4$.
Figure S18. $^{125}Te\{^{1}H\}$-NMR spectrum of $Te(CH_2)_4$.
Figure S19. ^{125}Te-NMR spectrum of $Te(CH_2)_4$.
Figure S20. $^{1}H,^{13}C$-HSQC-NMR spectrum of $Te(CH_2)_4$.
Figure S21. $^{1}H,^{125}Te$-HMBC-NMR spectrum of $Te(CH_2)_4$.
Figure S22. ^{1}H-NMR spectrum of $Te(CH_2)_5$.

Figure S23. $^{13}C\{^{1}H\}$-NMR spectrum of $Te(CH_2)_5$.
Figure S24. $^{125}Te\{^{1}H\}$-NMR spectrum of $Te(CH_2)_5$.
Figure S25. ^{125}Te-NMR spectrum of $Te(CH_2)_5$.
Figure S26. $^{1}H,^{1}H$-COSY-NMR spectrum of $Te(CH_2)_5$.
Figure S27. $^{1}H,^{13}C$-HSQC-NMR spectrum of $Te(CH_2)_5$.
Figure S28. $^{1}H,^{13}C$-HMBC-NMR spectrum of $Te(CH_2)_5$.
Figure S29. ^{1}H-NMR spectrum of 1,7-$Te_2(CH_2)_{10}$.
Figure S30. $^{13}C\{^{1}H\}$-NMR spectrum of 1,7-$Te_2(CH_2)_{10}$.
Figure S31. $^{125}Te\{^{1}H\}$-NMR spectrum of 1,7-$Te_2(CH_2)_{10}$.
Figure S32. ^{125}Te-NMR spectrum of 1,7-$Te_2(CH_2)_{10}$.
Figure S33. ^{1}H-NMR spectrum of 1,7,13-$Te_3(CH_2)_{15}$.
Figure S34. $^{13}C\{^{1}H\}$-NMR spectrum of 1,7,13-$Te_3(CH_2)_{15}$.
Figure S35. $^{125}Te\{^{1}H\}$-NMR spectrum of 1,7,13-$Te_3(CH_2)_{15}$.
Figure S36. ^{125}Te-NMR spectrum of 1,7,13-$Te_3(CH_2)_{15}$.
Figure S37. $^{1}H,^{1}H$-COSY-NMR spectrum of 1,7,13-$Te_3(CH_2)_{15}$.
Figure S38. $^{1}H,^{13}C$-HSQC-NMR spectrum of 1,7,13-$Te_3(CH_2)_{15}$.
Figure S39. $^{1}H,^{13}C$-HMBC-NMR spectrum of 1,7,13-$Te_3(CH_2)_{15}$.
Figure S40. ^{1}H-NMR spectrum of 1,7,13,18-$Te_4(CH_2)_{20}$.
Figure S41. $^{13}C\{^{1}H\}$-NMR spectrum of 1,7,13,18-$Te_4(CH_2)_{20}$.
Figure S42. $^{125}Te\{^{1}H\}$-NMR spectrum of 1,7,13,18-$Te_4(CH_2)_{20}$.
Figure S43. ^{1}H-NMR spectrum of $Te(CH_2)_6$.
Figure S44. $^{13}C\{^{1}H\}$-NMR spectrum of $Te(CH_2)_6$.
Figure S45. $^{125}Te\{^{1}H\}$-NMR spectrum of $Te(CH_2)_6$.
Figure S46. ^{125}Te-NMR spectrum of $Te(CH_2)_6$.
Figure S47. $^{1}H,^{1}H$-COSY-NMR spectrum of $Te(CH_2)_6$.
Figure S48. $^{1}H,^{13}C$-HSQC-NMR spectrum of $Te(CH_2)_6$.
Figure S49. $^{1}H,^{13}C$-HMBC-NMR spectrum of $Te(CH_2)_6$.
Figure S50. $^{1}H,^{125}Te$-HMBC-NMR spectrum of $Te(CH_2)_6$.
Figure S51. ^{1}H-NMR spectrum of 1,8-$Te_2(CH_2)_{12}$.
Figure S52. $^{13}C\{^{1}H\}$-NMR spectrum of 1,8-$Te_2(CH_2)_{12}$.
Figure S53. $^{125}Te\{^{1}H\}$-NMR spectrum of 1,8-$Te_2(CH_2)_{12}$.
Figure S54. ^{125}Te-NMR spectrum of 1,8-$Te_2(CH_2)_{12}$.
Figure S55. $^{1}H,^{1}H$-COSY-NMR spectrum of 1,8-$Te_2(CH_2)_{12}$.
Figure S56. $^{1}H,^{13}C$-HSQC-NMR spectrum of 1,8-$Te_2(CH_2)_{12}$.
Figure S57. $^{1}H,^{13}C$-HMBC-NMR spectrum of 1,8-$Te_2(CH_2)_{12}$.
Figure S58. $^{1}H,^{125}Te$-HMBC-NMR spectrum of 1,8-$Te_2(CH_2)_{12}$.
Figure S59. ^{1}H-NMR spectrum of 1,8,15-$Te_3(CH_2)_{18}$.
Figure S60. $^{13}C\{^{1}H\}$-NMR spectrum of 1,8,15-$Te_3(CH_2)_{18}$.
Figure S61. $^{125}Te\{^{1}H\}$-NMR spectrum of 1,8,15-$Te_3(CH_2)_{18}$.
Figure S62. ^{125}Te-NMR spectrum of 1,8,15-$Te_3(CH_2)_{18}$.
Figure S63. $^{1}H,^{1}H$-COSY-NMR spectrum of 1,8,15-$Te_3(CH_2)_{18}$.
Figure S64. $^{1}H,^{13}C$-HSQC-NMR spectrum of 1,8,15-$Te_3(CH_2)_{18}$.
Figure S65. $^{1}H,^{13}C$-HMBC-NMR spectrum of 1,8,15-$Te_3(CH_2)_{18}$.
Figure S66. $^{1}H,^{125}Te$-HMBC-NMR spectrum of 1,8,15-$Te_3(CH_2)_{18}$.
Figure S67. ^{1}H-NMR spectrum of 1,8,15,22-$Te_4(CH_2)_{24}$.
Figure S68. $^{13}C\{^{1}H\}$-NMR spectrum of 1,8,15,22-$Te_4(CH_2)_{24}$.
Figure S69. $^{125}Te\{^{1}H\}$-NMR spectrum of 1,8,15,22-$Te_4(CH_2)_{24}$.
Figure S70. ^{125}Te-NMR spectrum of 1,8,15,22-$Te_4(CH_2)_{24}$.
Figure S71. $^{1}H,^{1}H$-COSY-NMR spectrum of 1,8,15,22-$Te_4(CH_2)_{24}$.
Figure S72. $^{1}H,^{13}C$-HSQC-NMR spectrum of 1,8,15,22-$Te_4(CH_2)_{24}$.

Figure S73. $^{1}H,^{13}C$-HMBC-NMR spectrum of 1,8,15,22-$Te_4(CH_2)_{24}$.
Figure S74. $^{1}H,^{125}Te$-HMBC-NMR spectrum of 1,8,15,22-$Te_4(CH_2)_{24}$.
Figure S75. ^{125}Te-NMR spectrum of $Te(CH_2)_7$.
Figure S76. ^{1}H-NMR spectrum of 1,9-$Te_2(CH_2)_{14}$.
Figure S77. $^{13}C\{^{1}H\}$-NMR spectrum of 1,9-$Te_2(CH_2)_{14}$.
Figure S78. $^{125}Te\{^{1}H\}$-NMR spectrum of 1,9-$Te_2(CH_2)_{14}$.
Figure S79. ^{125}Te-NMR spectrum of 1,9-$Te_2(CH_2)_{14}$.
Figure S80. $^{1}H,^{1}H$-COSY-NMR spectrum of 1,9-$Te_2(CH_2)_{14}$.
Figure S81. $^{1}H,^{13}C$-HSQC-NMR spectrum of 1,9-$Te_2(CH_2)_{14}$.
Figure S82. $^{1}H,^{13}C$-HMBC-NMR spectrum of 1,9-$Te_2(CH_2)_{14}$.
Figure S83. $^{1}H,^{125}Te$-HMBC-NMR spectrum of 1,9-$Te_2(CH_2)_{14}$.
Figure S84. ^{1}H-NMR spectrum of 1,9,17-$Te_3(CH_2)_{21}$.
Figure S85. $^{13}C\{^{1}H\}$-NMR spectrum of 1,9,17-$Te_3(CH_2)_{21}$.
Figure S86. $^{125}Te\{^{1}H\}$-NMR spectrum of 1,9,17-$Te_3(CH_2)_{21}$.
Figure S87. ^{125}Te-NMR spectrum of 1,9,17-$Te_3(CH_2)_{21}$.
Figure S88. $^{1}H,^{1}H$-COSY-NMR spectrum of 1,9,17-$Te_3(CH_2)_{21}$.
Figure S89. $^{1}H,^{13}C$-HSQC-NMR spectrum of 1,9,17-$Te_3(CH_2)_{21}$.
Figure S90. $^{1}H,^{13}C$-HMBC-NMR spectrum of 1,9,17-$Te_3(CH_2)_{21}$.
Figure S91. $^{1}H,^{125}Te$-HMBC-NMR spectrum of 1,9,17-$Te_3(CH_2)_{21}$.
Figure S92. ^{1}H-NMR spectrum of 1,9,17,25-$Te_4(CH_2)_{28}$.
Figure S93. $^{13}C\{^{1}H\}$-NMR spectrum of 1,9,17,25-$Te_4(CH_2)_{28}$.
Figure S94. $^{125}Te\{^{1}H\}$-NMR spectrum of 1,9,17,25-$Te_4(CH_2)_{28}$.
Figure S95. ^{125}Te-NMR spectrum of 1,9,17,25-$Te_4(CH_2)_{28}$.
Figure S96. $^{1}H,^{1}H$-COSY-NMR spectrum of 1,9,17,25-$Te_4(CH_2)_{28}$.
Figure S97. $^{1}H,^{13}C$-HSQC-NMR spectrum of 1,9,17,25-$Te_4(CH_2)_{28}$.
Figure S98. $^{1}H,^{13}C$-HMBC-NMR spectrum of 1,9,17,25-$Te_4(CH_2)_{28}$.
Figure S99. $^{1}H,^{125}Te$-HMBC-NMR spectrum of 1,9,17,25-$Te_4(CH_2)_{28}$.

Computational Results

Table S6. Absolute energies, enthalpies, and Gibbs energies from ORCA PBE0/def2-TZVPP calculations.

Table S7. Absolute energies (E), enthalpies (H), and Gibbs (G) energies (Hartree) from Crystal17 PBE0/pob-TZVP calculations.

Isolation of Reaction Products

1,5,9-$Te_3(CH_2)_9$. The reaction mixture consisted of a brownish black solution and some orange oil beside large amounts of white solid material. The solution was separated by decantation and the solvent was removed by evaporation under reduced pressure. The resulting residue as well as the leftover material after decantation were extracted with chloroform. The combined extracts were filtered, and the solvent was removed by evaporation under reduced pressure. The raw product was dissolved in the hexane/chloroform mixture (2:1) for column chromatography, which was performed in dark. The first few fractions were not well separated and consisted of compounds that decomposed quickly, although the solutions were kept in dark. The decomposition was accompanied by the precipitation of white solid material. Thin layer chromatography showed three species: *Rf* = 0.81 (orange spot, almost immediate decomposition when placed under the UV lamp), *Rf* = 0.72 (light blue fluorescence), *Rf* = 0.61 (yellow spot, intense greenish fluorescence). 1,5,9-$Te_3(CH_2)_9$ (Rf = 0.55) was obtained in the form of slightly yellow crystals (see Table S1). The EI-MS spectrum of pure isolated 1,5,9-$Te_3(CH_2)_9$ is shown in Figures S2(a) and S6(a), and the NMR spectroscopic data are shown in Figures S10-S15.

$Te(CH_2)_4$. The reaction solution was decanted, and the solvent was removed by evaporation under reduced pressure leaving some solid residue behind. $Te(CH_2)_4$ is slightly volatile and therefore some material was removed together upon evaporation of ethanol. After the removal of ethanol, the solid residue was extracted with DCM. The extract was washed with water (three times), dried over sodium sulfate and filtered. The solvent was removed under reduced pressure yielding almost pure $Te(CH_2)_4$ (see Table S1), as indicated by MS and NMR spectroscopic information [see Figures S2(b), S6(b), S16-S21]. No larger ring compounds could be detected (see Figure 1 in the main text).

$[Te(CH_2)_5]_n$ (*n* = 1-4). The reaction mixture was stirred under exclusion of light for 15 h. The solvent was evaporated under reduced pressure and 75 mL of chloroform was added to the residue. After ultrasonication the mixture was filtered, and the black residue was extracted three times with 50 mL of chloroform. The combined filtrates were passed through a frit equipped with silica gel and sand to remove all insoluble

residues. The solvent was evaporated under reduced pressure yielding 0.674 g of a yellow to orange oil. The reaction products were separated by column chromatography using a mixture of hexane and chloroform (3:1) as an eluant. The following four compounds were isolated (for the isolated yields, see Table S1) $Te(CH_2)_5$ (R_f = 0.88, yellow liquid with unpleasant odor), 1,7-$Te_2(CH_2)_{10}$ (R_f = 0.72, colorless crystals), 1,7,13-$Te_3(CH_2)_{15}$ (R_f = 0.53, slightly yellow oil), and 1,7,13,19-$Te_4(CH_2)_{20}$ (R_f = 0.41, colorless crystals). The purity and the spectroscopic characterization of the products are shown in Figures S3, S7, S22-S42.

$[Te(CH_2)_6]_n$ (*n* = 1-4). The reaction mixture was stirred under exclusion of light for 22 h. The solvent was evaporated under reduced pressure and the residue was dissolved in 75 mL chloroform. The mixture was passed through a frit equipped with silica gel and sand to remove insoluble residues. The solvent was evaporated under reduced pressure yielding a viscous yellow oil. The reaction products were separated by column chromatography using hexane chloroform mixture (2:1) as an eluant. The following four products were isolated (For the isolated yields, see Table S1): $Te(CH_2)_6$ (R_f = 0.90, yellow liquid with unpleasant odor), 1,8-$Te_2(CH_2)_{12}$ (R_f = 0.72, colorless crystals), 1,8,15-$Te_3(CH_2)_{18}$ (R_f = 0.61, slightly yellow oil), and 1,8,15,22-$Te_4(CH_2)_{24}$ (R_f = 0.49, colorless crystals). The MS and NMR spectroscopic data are shown in Figures S4, S8, and S43-S74, respectively.

$[Te(CH_2)_7]_n$ (*n* = 1-4). The reaction mixture was stirred under exclusion of light for 67 h. The solvent was evaporated under reduced pressure and the residue was dissolved in 75 mL chloroform. The mixture was passed through a frit equipped with silica gel and sand to remove insoluble residues. The solvent was evaporated under reduced pressure yielding a viscous yellow oil. The reaction products were separated by column chromatography using hexane chloroform mixture (3:1) as an eluant. The following four products were isolated: $Te(CH_2)_7$ (R_f = 0.73; yellow liquid with unpleasant odor; not a complete isolation), 1,9-$Te_2(CH_2)_{14}$ (R_f = 0.57, slightly yellow oil), 1,9,17-$Te_3(CH_2)_{21}$ (R_f = 0.44, slightly yellow oil) 1,9,17,25-$Te_3(CH_2)_{28}$ (R_f = 0.34, colorless crystals). ES and NMR data are summarized in Figures S5, S9, and S75-S99.

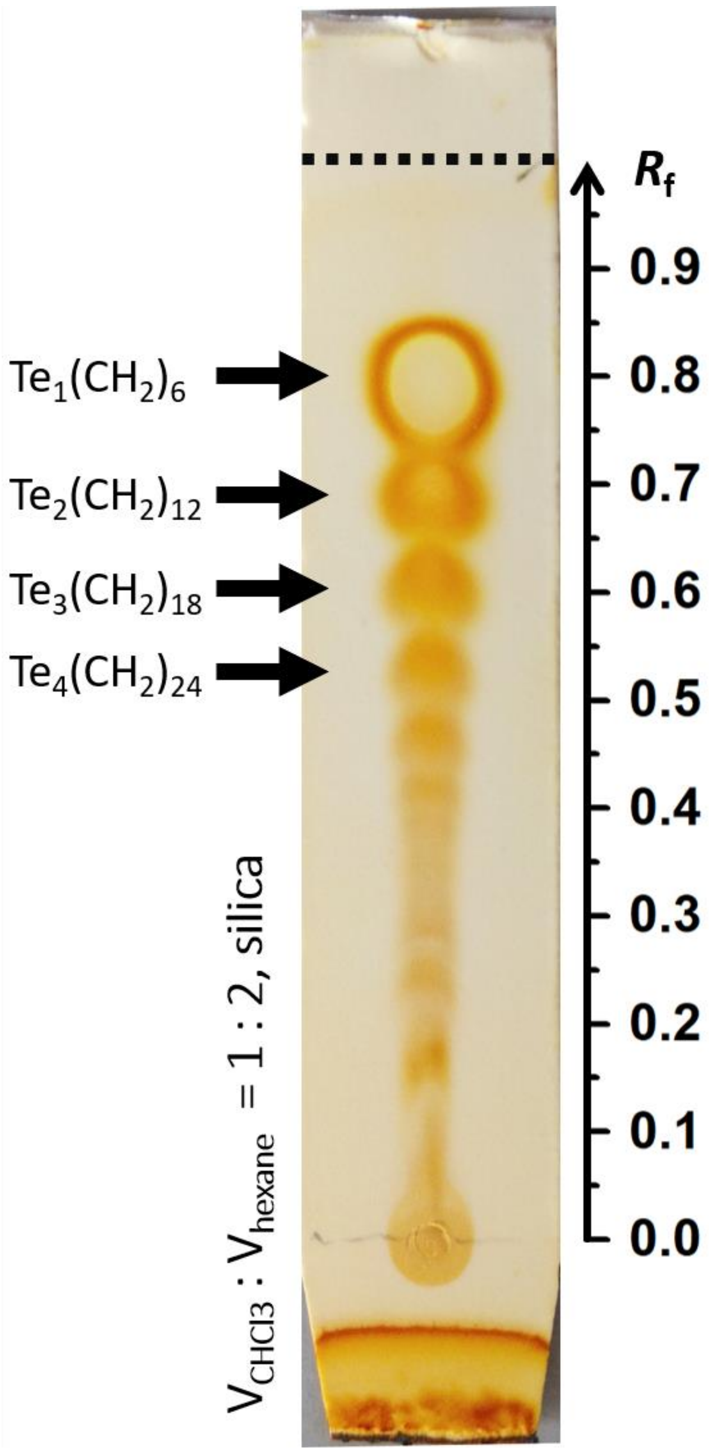

Figure S1. Thin layer chromatogram of the reaction of Na_2Te and $Br(CH_2)_6Br$. The chromatogram was stained in an iodine vapor chamber.

Table S1. Preparative details of the $[Te(CH_2)_m]_n$ (n = 1-4; m = 3-7) ring molecules.

m	Reaction [a]	$Te(CH_2)_m$ [b]	$[Te(CH_2)m]_2$ [b]	$[Te(CH_2)_m]_3$ [b]	$[Te(CH_2)_m]_4$ [b]
3	Na_2Te + $Br(CH_2)_3Br$<br>1.00 g 1.54 g<br>7.84 mmol 7.62 mmol			0.102 g (0.20 mmol; 8 %)	
4	Na_2Te + $Br(CH_2)_4Br$<br>1.00 g 1.69 g<br>7.84 mmol 7.85 mmol	0.309 g (1.68 mmol; 21 %)			
5	Na_2Te + $Br(CH_2)_5Br$<br>1.00 g 1.80 g<br>7.84 mmol 7.84 mmol	0.111 g (0.56 mmol; 7 %)	0.022 g (0.06 mmol; 1 %)	0.037 g (0.06 mmol; 2 %)	0.017 g (0.02 mmol; 1 %)
6	Na_2Te + $Br(CH_2)_6Br$<br>1.00 g 1.91 g<br>7.84 mmol 7.84 mmol	0.333 g (1,57 mol; 20 %)	0.159 g (0.38 mmol; 10 %)<br>$C_{12}H_{24}Te_2$:<br>calc. C 34.03 %, H 5.67 %<br>obs. C 34,53 %, H 5.64 %	0.140 g (0.22 mmol; 8 %)<br>$C_{18}H_{36}Te_3$:<br>calc. C 34.03 %, H 5.67 %<br>obs. C 34.46 %, H 5.67 %	0.098 g (0.12 mmol; 6 %)
7	Na_2Te + $Br(CH_2)_7Br$<br>1.00 g 2.09 g<br>7.84 mmol 8.11 mmol	n.a.	0.217 g (0.48 mmol; 12 %)<br>$C_{14}H_{28}Te_2$:<br>calc. C 37.23 %, H 6.21 %<br>obs. C 38.47 %, H 6.36 %	0.189 g (0.28 mmol; 11 %)<br>$C_{21}H_{42}Te_3$:<br>calc. C 37.23 %, H 6.21 %<br>obs. C 40.46 %, H 6.69 %	0.015 g (0.02 mmol; 1 %)

[a] The mass spectroscopic and NMR spectroscopic characterization of the isolated products is presented in Figures S1-S20. [b] Isolated yield.

Crystal Structure Determination of 1,7-$Te_2(CH_2)_{10}$, 1,8-$Te_2(CH_2)_{12}$, 1,5,9-$Te_3(CH_2)_9$, 1,8,15-$Te_3(CH_2)_{18}$, 1,7,13,19-$Te_4(CH_2)_{20}$, 1,8,15,22-$Te_4(CH_2)_{24}$, and 1,9,17,25-$Te_4(CH_2)_{28}$.

Table S2. Crystal Data and Details of Structure Determination.

Compound	1,7-$Te_2(CH_2)_{10}$	1,8-$Te_2(CH_2)_{12}$	1,5,9-$Te_3(CH_2)_9$	1,8,15-$Te_3(CH_2)_{18}$	1,7,13,19-$Te_4(CH_2)_{20}$	1,8,15,22-$Te_4(CH_2)_{24}$	1,9,17,25-$Te_4(CH_2)_{28}$
empirical formula	$C_{10}H_{20}Te_2$	$C_{12}H_{24}Te_2$	$C_9H_{18}Te_3$	$C_{18}H_{36}Te_3$	$C_{20}H_{40}Te_4$	$C_{24}H_{48}Te_4$	$C_{28}H_{56}Te_4$
fw	395.46	423.51	509.03	635.27	790.92	847.02	903.12
crystallization solvent	hexane/ chloroform	pentane	hexane/ chloroform	pentane	hexane/ dichloromethane	hexane/ dichloromethane	hexane/ dichloromethane
crystal system	triclinicic	monoclinic	orthorhombic	triclinic	tetragonal	tetragonal	tetragonal
space group	*P*-1	$P2_1/n$	$Pna2_1$	*P*-1	*I*-42*m*	*P*4/*nnc*	*I*-42*m*
a (Å)	6.2239(6)	8.1347(2)	15.9227(7)	10.6138(3)	16.8651(5)	18.9285(6)	20.5235(6)
b (Å)	7.0966(6)	5.7422(4)	14.4772(6)	12,5112(5)	16.8651(5)	18.9285(6)	20.5235(6)
c (Å)	7.4227(6)	15.5150(7)	5.8438(2)	18.0694(4)	5.0081(1)	4.7709(2)	4.9249(2)
α (deg)	74.553(3)			100.073(2)			
β (deg	89.272(6)	91.498(3)		92.150(2)			
γ (deg)	78.819(7)			109.534(2)			
V (Å^3)	309.75(5)	724.47(6)	1347.09(9)	2214.55(12)	1424.46(7)	1709.36(10)	2074.44(15)
Z	1	2	4	4	2	2	2
F(000)	184	400	912	1200	736	800	864
ρ (g cm^{-3})	2.120	1.941	2.510	1.905	1.844	1.646	1.446
μ (mm^{-1})	4.664	3.995	6.412	3.921	4.057	3.387	2.795
Crystal size (mm)	0.044x0.042x0.022	0.102x0.034x0.034	0.122x0.108x0.066	0.040x0.040x0.040	0.044x0.034x0.024	0.112x0.088x0.064	0.122x0.048x0.042
independent/ observed reflections ^{a}	2986/1360	4254/1664	8937/2951	14032/9935	7210/862	17078/989	14000/1242
Parameters/	80/0	112/0	109/1	379/0	30/0	33/0	39/0

restraints							
R_1 [$I > 2\sigma(I)$] [b]	0.0568	0.0207	0.0383	0.0636	0.0205	0.0357	0.0175
$wR2$ (all data) [c]	0.1646	0.0470	0.0798	0.1744	0.0694	0.0847	0.0344
GOP	1.078	1.193	1.068	1.145	1.265	1.361	1.120
Residual electon density (e Å^{-3})	2.087/-0.754	0.592/-0.548	1.132/-0.790	1.705/-1.266	1.719/-0.411	0.913/-0.336	0.306/-0.257

[a] $I > 2\sigma(I)$. [b] $R_1 = \Sigma||F_o| - |F_c||/\Sigma|F_o|$ [c] $wR_2 = [\Sigma w(F_o^2 - F_c^2)^2/\Sigma wF_o^4]^{½}$.

Table S3. Selected bond lengths (Å) and angles (°)

**1,7-$Te_2(CH_2)_{10}$**		**1,5,9-$Te_3(CH_2)_9$**		**1,8,15-$Te_3(CH_2)_{18}$**		**1,7,13,19-$Te_4(CH_2)_{20}$**	
Te1-C1	2.179(12)	Te1-C1	2.167(9)	Te1A-C1A	2.149(12)	Te1-C1	2.171(5)
Te1-C1A	2.147(13)	Te1-C9	2.179(9)	Te1A-C18A	2.156(11)		
Te1A-C5	2.124(13)	Te2-C3	2.159(8)	Te2A-C6A	2.164(12)	C1-Te1-C1[a]	91.0(3)
Te1A-C5A	2.159(13)	Te2-C4	2.159(8)	Te2A-C7A	2.164(12)		
		Te3-C6	2.165(7)	Te3A-C12A	2.157(12)	**1,8,15,22-$Te_4(CH_2)_{24}$**	
C1A-Te1-C1	93.6(5)	Te3-C7	2.154(9)	Te3A-C13A	2.160(12)	Te1-C1	2.150(5)
C5A-Te1A-C5	94.1(5)			Te1B-C1B	2.146(12)		
		C1-Te1-C9	96.9(4)	Te1B-C18B	2.152(12)	C1-Te1-C1[b]	92.8(3)
**1,8-$Te_2(CH_2)_{12}$**		C3-Te2-C4	94.6(4)	Te2B-C6B	2.155(12)		
Te1-C1	2.153(3)	C6-Te3-C7	93.8(3)	Te2B-C7B	2.143(11)	**1,9,17,25-$Te_4(CH_2)_{28}$**	
Te1-C6	2.159(3)			Te3B-C12B	2.152(10)	Te1-C1	2.154(5)
				Te3B-C13B	2.146(14)		
C1-Te1-C6	95.87(11)					C1-Te1-C1[c]	92.8(3)
				C1A-Te1A-C18A	95.2(4)		
				C6A-Te2A-C7A	94.6(5)		
				C12A-Te3A-C13A	94.1(5)		
				C1B-Te1B-C18B	93.5(5)		
				C6B-Te2B-C7B	95.3(5)		
				C12B-Te3B-C13B	93.6(5)		

[a] symmetry operation: *y*, 1-*x*, 1-*z*. [b] symmetry operation: ½-*x*, *y*, ½-*z*. [c] symmetry operation: *x*, 1-*y*, 1-*z*

Table S4. The intermolecular chalcogen-chalcogen contacts (in Å) in some cyclic unsaturated chalcogenoethers and related open-chain species.

Thioethers [a]	S···S	Selenoethers [a]	Se···Se	Telluroethers [a]	Te···Te
$\{C{\equiv}C\}_2(CH_2)_mS$ (m = 7, 8)	3.584-3.699 [b]				
$\{C{\equiv}C\}_2(CH_2)_mS_2$ (m = 7-10,12)	3.653-3.918 [c]	$\{C{\equiv}C\}_2(CH_2)_7Se_2$	3.641[c]	$\{C{\equiv}C\}_2(CH_2)_8Te_2$	3.814 [c]
$\{C{\equiv}C\}_n(CH_2)_mS_4$ (n = 2,4; m = 4-12)	3.506-3.995 [d, e]	$\{C{\equiv}C\}_n(CH_2)_mSe_4$ (n = 2,4; m = 4,6-8,10)	3,582-3.984 [f, d]		
$\{C{\equiv}C\}_n(CH_2)_mS_6$ (n = 3,6; m = 4-12,15)	3.368-3.968 [d, g]	$\{C{\equiv}C\}_6(CH_2)_{15}Se_6$	3.769-3.983 [d]		
$\{C{\equiv}C\}_n(CH_2)_mS_8$	3.722-3.913 [h]				
$\{C{\equiv}C\}_2\{HC{=}CH\}(CH_2)_{10}S_4$	3.746 [i]				
$\{HC{=}CH\}_2(CH_2)_mS_4$ (m = 6,8-10)	3.473-3.975 [i]				
		MeSe$\{C{\equiv}C\}_4$SeMe	3.666-3.731 [j]	MeTe$\{C{\equiv}C\}_n$TeMe (n = 2-4)	3.688-4.066 [k]

[a] The species considered herein contain only divalent chalcogen atoms, methylene groups CH_2, and -C≡C- or -C(H)=C(H)- fragments. [b] R. Gleiter, J. Classen, B. J. Rausch, T. Oeser F. Rominger, *J. Organomet. Chem.* **2002**, *641*, 3-8. [c] J. H. Schulte, D. B. Werz, F. Rominger, R. Gleiter, *Org. Biolmol. Chem.* **2003**, 2788-2794. [d] D. B. Werz, R. Gleiter, F. Rominger, *J. Org. Chem.* **2004** , *69*, 2945-2952. [e] C. Benisch, S. Bethke, R. Gleiter, T. Oeser, H. Pritzkow, F. Rominger, *Eur. J. Org. Chem.* **2000**, 2479-2488. [f] D. B. Werz, R. Gleiter, F. Rominger, *J. Org. Chem.* **2002**, *67*, 4290-4297. [g] D. B. Werz, R. Gleiter, F. Rominger, *J. Org. Chem.* **2004** , *69*, 2945-2952. [h] B. J. Rausch, D. B. Werz, S. Rittinger, R. Gleiter, T. Oeser, F. Rominger, *J. Chem. Soc., Perkin Trans. 2* **2002**,72-76. [i] T. H. Staeb, R. Gleiter, F. Rominger, *Eur. J. Org. Chem.* **2002**, 2815-2822. [j] D. B. Werz, R. Gleiter, *J. Org. Chem.* **2003**, *68*, 9400-9405. [k] D. B. Werz, R. Gleiter, F. Rominger, *Organometallics* **2003**, *22*, 843-849.

Mass spectra of $[Te(CH_2)_m]_n$ (*n* = 1-4; *m* = 3-7)

(a) **1,5,9-$Te_3(CH_2)_9$**

(b) **$Te(CH_2)_4$**

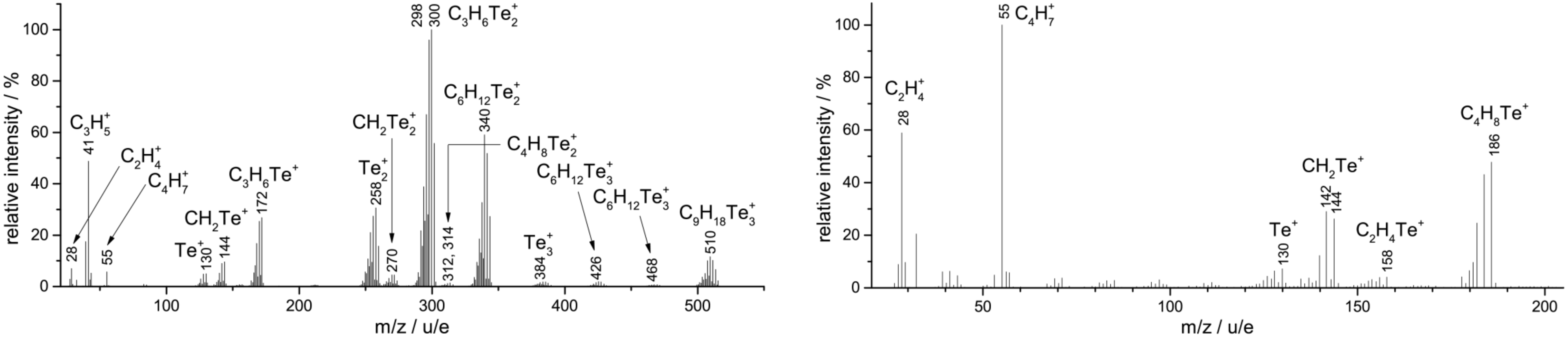

Figure S2. The mass spectra of the isolated products of the reaction of (a) Na_2Te and $Br(CH_2)_3Br$ and (b) Na_2Te and $Br(CH_2)_4Br$.

Te(CH₂)₅

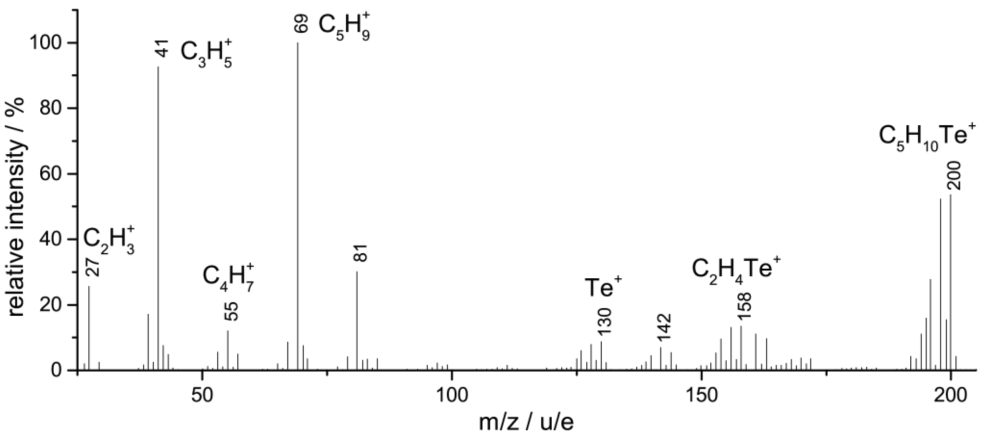

1,7,13-Te₃(CH₂)₁₅

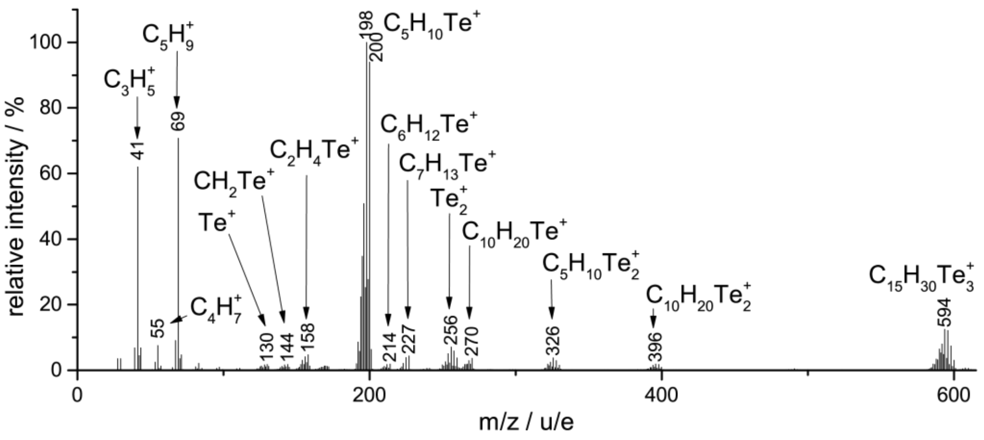

1,7-Te₂(CH₂)₁₀

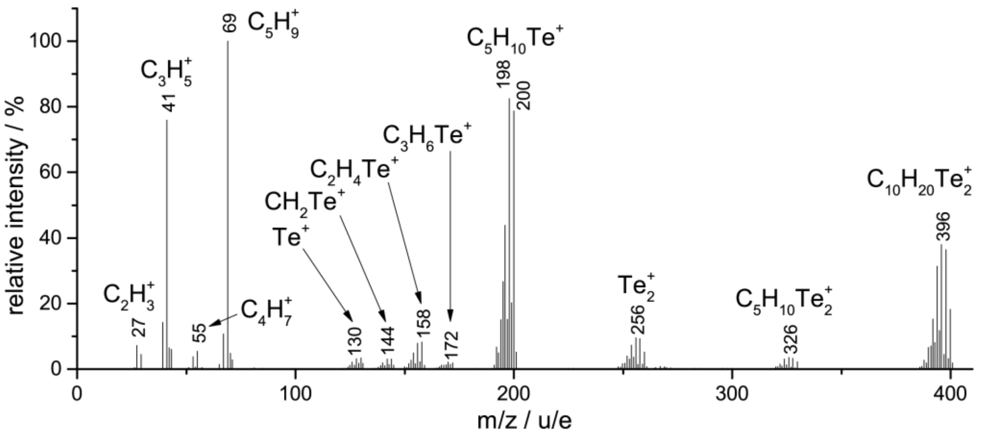

1,7,13,19-Te₄(CH₂)₂₀

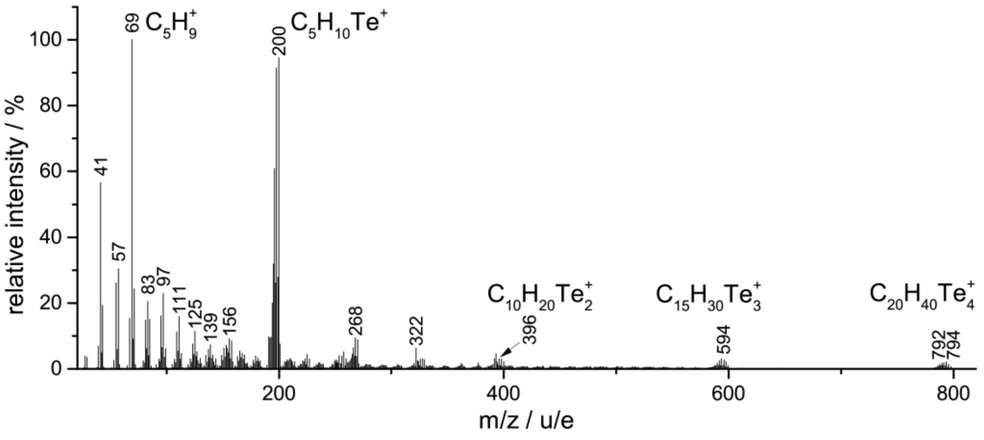

Figure S3. The mass spectra of the isolated products of the reaction of Na_2Te and $Br(CH_2)_5Br$.

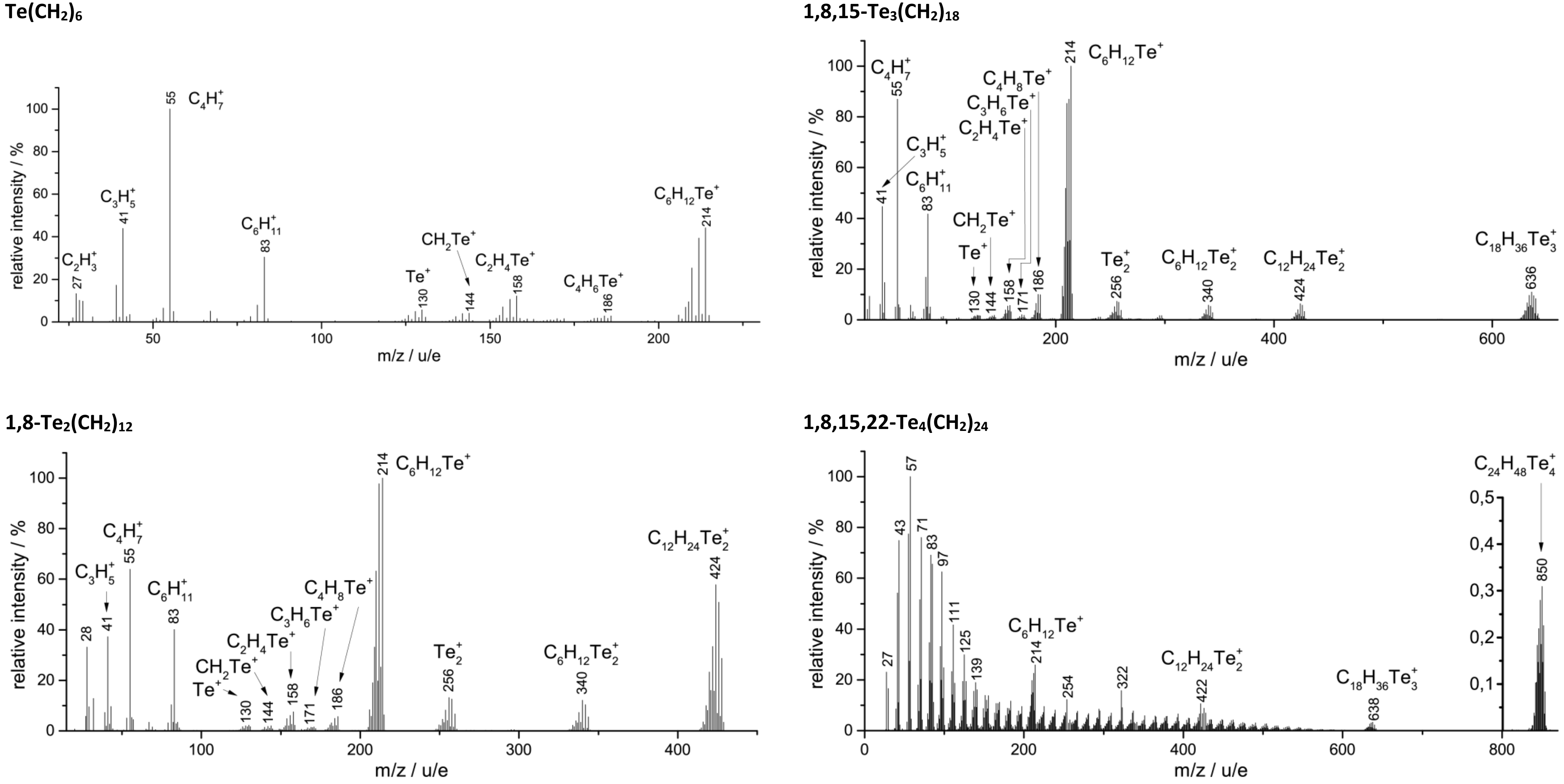

Figure S4. The mass spectra of the isolated products of the reaction of Na_2Te and $Br(CH_2)_6Br$.

1,9-$Te_2(CH_2)_{14}$

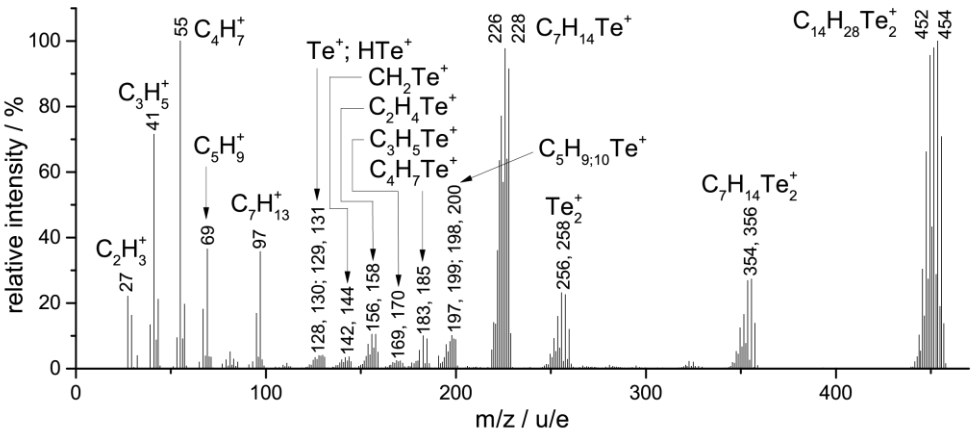

1,9,17-$Te_3(CH_2)_{21}$

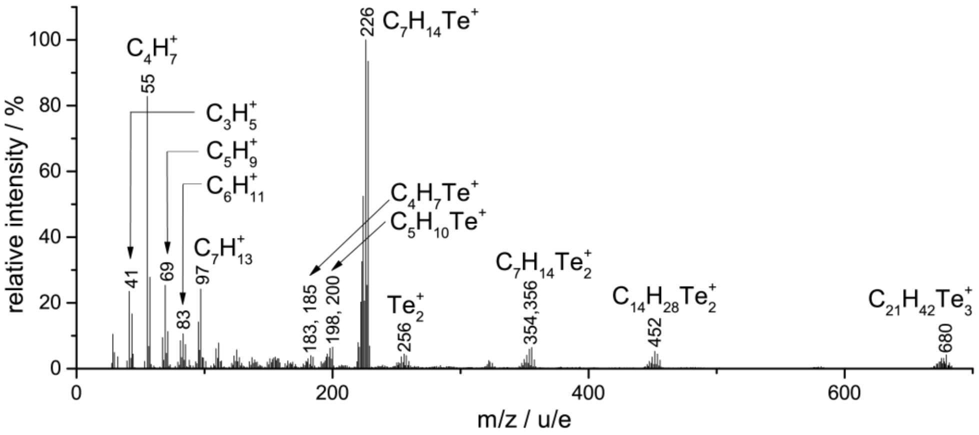

1,9,17,25-$Te_4(CH_2)_{28}$

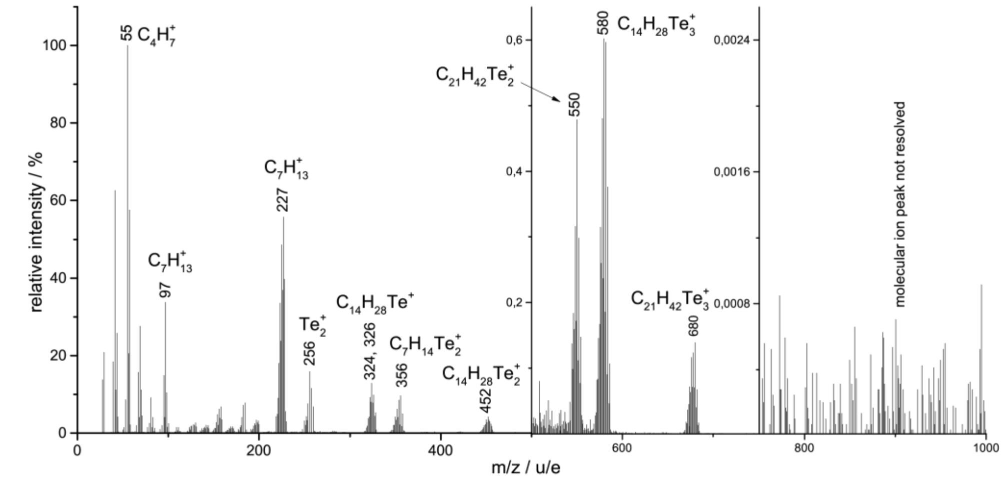

Figure S5. The mass spectra of the isolated products of the reaction of Na_2Te and $Br(CH_2)_7Br$.

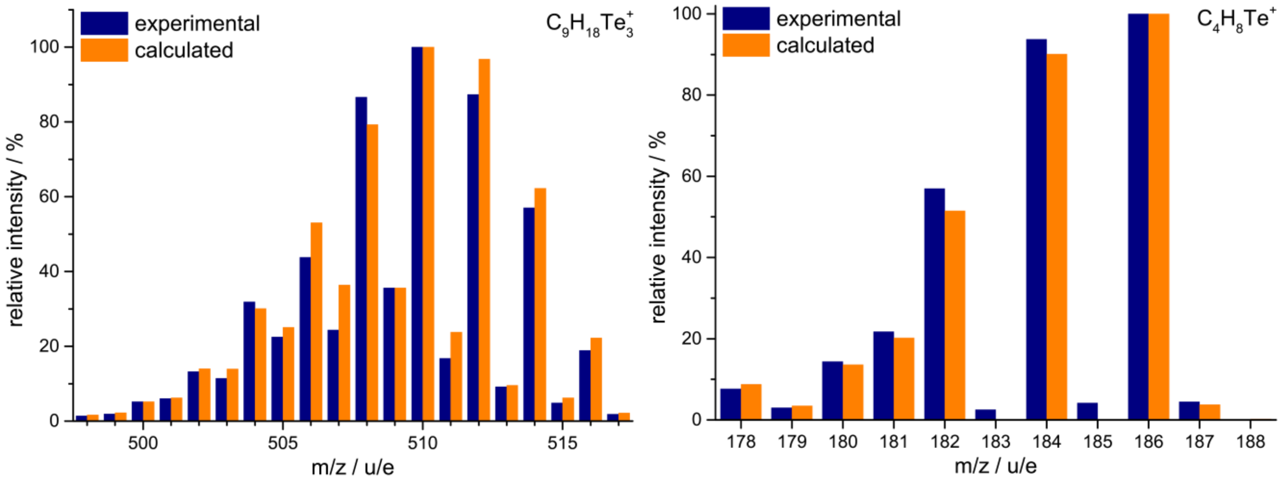

Figure S6. The experimental and calculated isotopic distributions of the molecular ion of the isolated products in the reaction of (a) Na_2Te and $Br(CH_2)_3Br$, and (b) Na_2Te and $Br(CH_2)_4Br$.

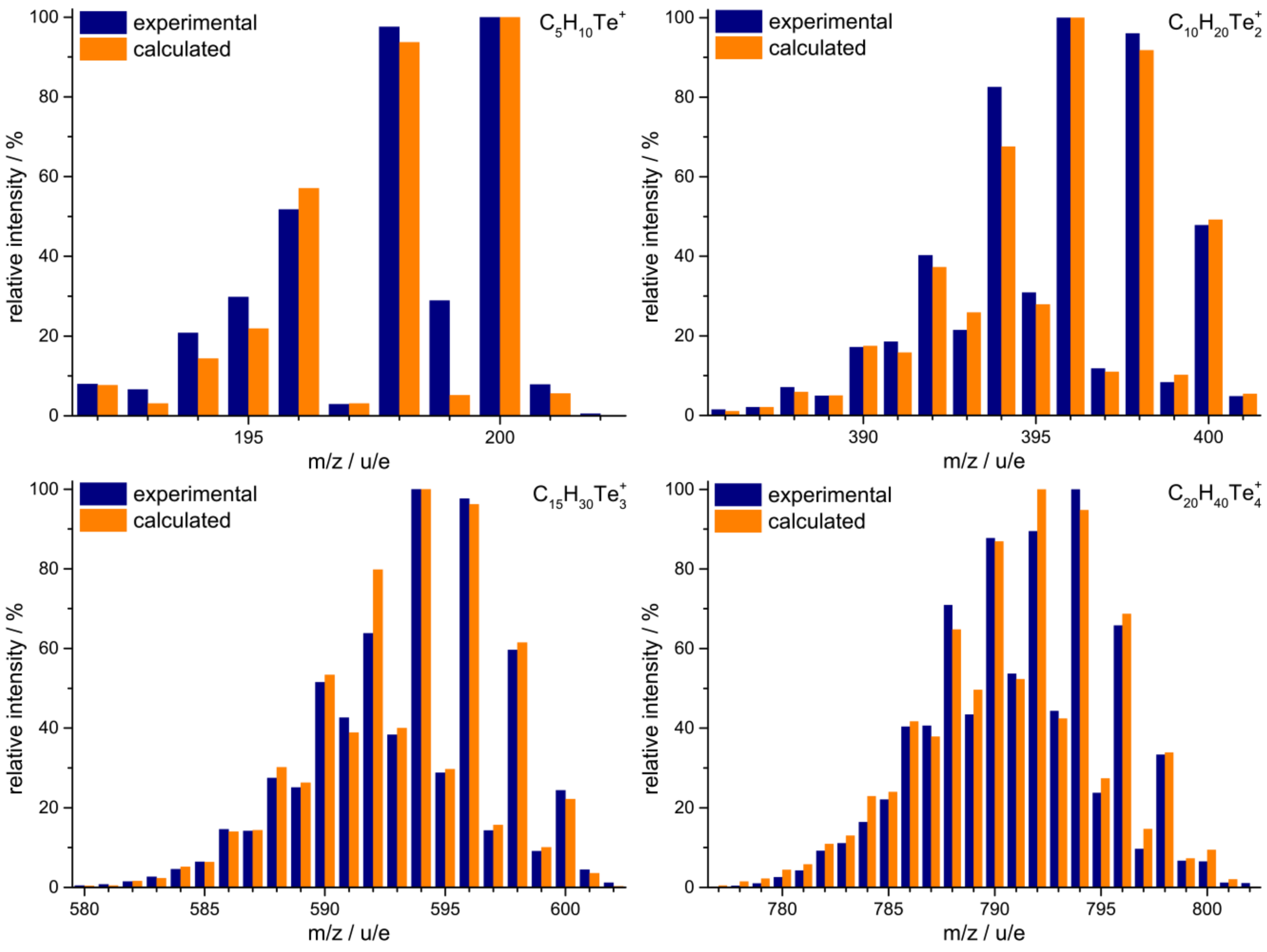

Figure S7. The experimental and calculated isotopic distributions of the isolated products in the reaction of Na_2Te and $Br(CH_2)_5Br$.

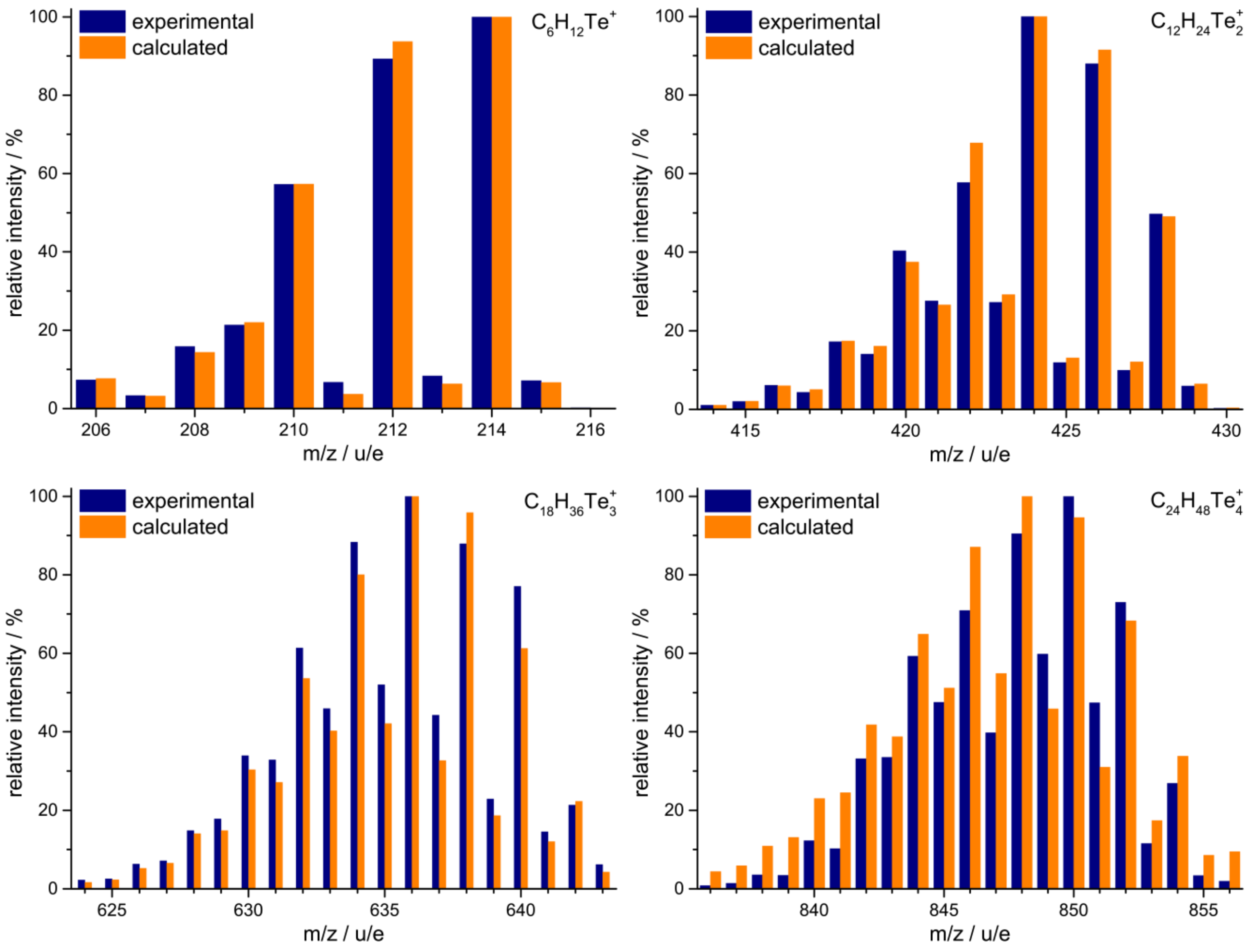

Figure S8. The experimental and calculated isotopic distributions of the isolated products in the reaction of Na_2Te and $Br(CH_2)_6Br$.

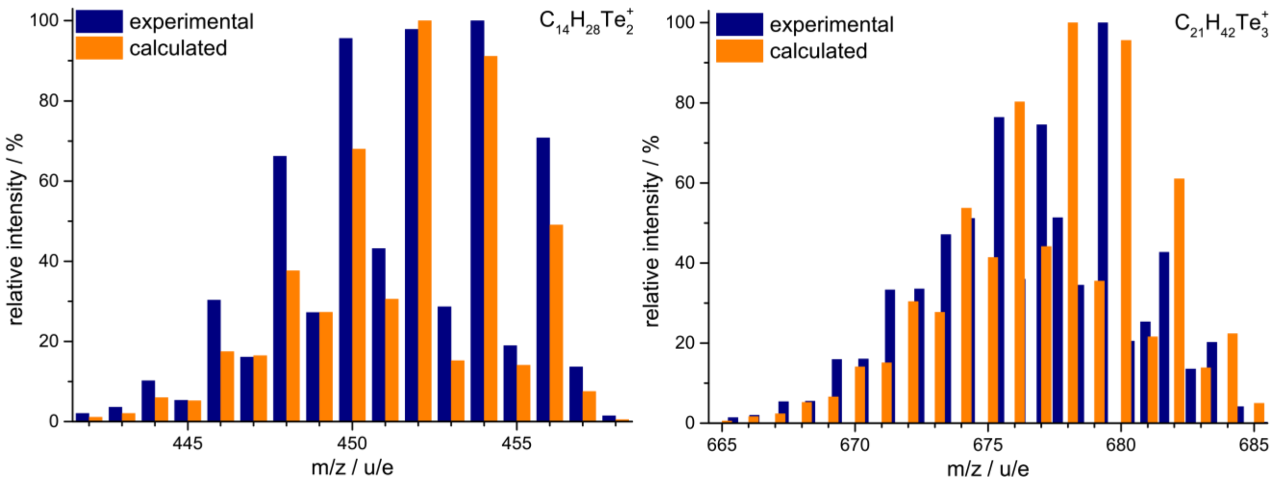

Figure S9. The experimental and calculated isotopic distributions of the isolated products in the reaction of Na_2Te and $Br(CH_2)_7Br$.

NMR Spectroscopic Information of $[Te(CH_2)_m]_n$ (*n* = 1-4; *m* = 3-7)

Table S5. Summary of the ^{1}H, ^{13}C, and ^{125}Te NMR spectroscopic information for the different $[Te(CH_2)_m]_n$ species. The data are recorded in $CDCl_3$ at 24 °C. (see Figures S10-S99 for individual NMR spectra).

(a) Chemical shifts (ppm) [a]

Compound	δ(Te)	$\delta(H_a)$	$\delta(H_b)$	$\delta(H_c)$	$\delta(H_d)$	$\delta(C_a)$	$\delta(C_b)$	$\delta(C_c)$	$\delta(C_d)$
1,5,9-$Te_3(CH_2)_9$	213	2.73	2.12	-	-	5.03	34.20	-	-
$Te(CH_2)_4$	235	3.15	2.04	-	-	6.33[c]	36.12[c]	-	-
$Te(CH_2)_5$	205	2.72	2.02	1.57	-	-3.53	28.72	29.65	-
1,7-$Te_2(CH_2)_{10}$	221	3.64	1.77	1.64	-	1.50	29.38	31.67	-
1,7,13-$Te_3(CH_2)_{15}$	242	2.63	1.78	1.50	-	2.38	31.69	34.14	-
1,7,13,19-$Te_4(CH_2)_{20}$	238	2,65	1.79	1.48	-	2.49	31.57	34.17	-
$Te(CH_2)_6$	209	2.80	2.13	1.65	-	1.63	31.81	28.24	-
1,8-$Te_2(CH_2)_{12}$	260	2.60	1.79	1.48	-	1.56	31.31	29.68	-
1,8,15-$Te_2(CH_2)_{18}$	232	2.63	1.76	1.43	-	2.76	31.98	30.87	-
1,8,15,22-$Te_4(CH_2)_{24}$	234	2.63	1.75	1.41	-	2.79	32.34	31.32	-
$Te(CH_2)_7$[b]	245	2.81	2.04	1.58	-	2.68	29.49	27.10	23.73
1,9-$Te_2(CH_2)_{14}$	230	2.63	1.74	1.48-1.32		2.53	31.70	30.78	27.44
1,9,17-$Te_3(CH_2)_{21}$	235	2.62	1.75	1.45-1.30		2.70	32.22	31.60	28.02
1,9,17,25-$Te_4(CH_2)_{28}$	233	2.63	1.74	1.45-1.31		2.82	32.30	31.86	28.27

(b) Coupling constants (Hz) [a]

Compound	$^{1}J(C_a\text{-}H)$	$^{1}J(C_b\text{-}H)$	$^{1}J(C_c\text{-}H)$	$^{1}J(C_d\text{-}H)$	$^{1}J(Te\text{-}C)$	$^{2}J(Te\text{-}H)$
1,5,9-$Te_3(CH_2)_9$	140	129	-	-	158	31
$Te(CH_2)_4$	144	d	-	-	124	26
$Te(CH_2)_5$	141	125	125[e]	-	129	30
1,7-$Te_2(CH_2)_{10}$	139	125	126	-	158	30
1,7,13-$Te_3(CH_2)_{15}$	139	125	125[e]	-	154	26
1,7,13,19-$Te_4(CH_2)_{20}$	140	126	-	-	-	24
$Te(CH_2)_6$	139	127[e]	-	-	145	33
1,8-$Te_2(CH_2)_{12}$	139	125	128	-	155	30
1,8,15-$Te_2(CH_2)_{18}$	141	125	125[e]	-	153	27
1,8,15,22-$Te_4(CH_2)_{24}$	139	127[e]	126[e]	-	152	24
$Te(CH_2)_7$[b]	137					35
1,9-$Te_2(CH_2)_{14}$	139	125	124[e]	123[e]	154	28
1,9,17-$Te_3(CH_2)_{21}$	139	125	124[e]	127[e]	152	25
1,9,17,25-$Te_4(CH_2)_{28}$	139	124			152	24

[a] The definition of the relative positions of the atoms H_a-H_d and C_a-C_d with respect to the tellurium atoms in different molecular species have been exemplified below for the monotelluroethers $Te(CH_2)_m$ (*m* = 4-7). [b] Data obtained from a mixed fraction $Te(CH_2)_7$: 1,9-$Te_2(CH_2)_{14}$ with molar ratio 1 : 3.5. These spectra are not included in the Supporting Information. [c] Data recorded at 27°C. [d] Satellite peaks clearly resolved but unclear peak centers because of higher order coupling and broken symmetry in spin system due to ^{13}C. [e] Data obtained from HMBC spectra. All other C-H coupling constants are taken from the ^{13}C satellites in the 1H-NMR spectra.

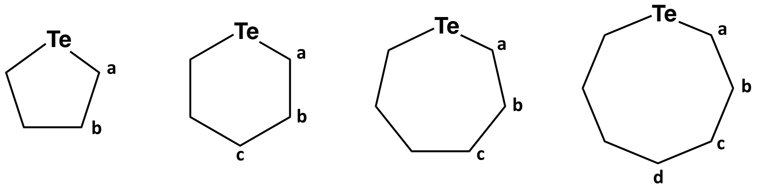

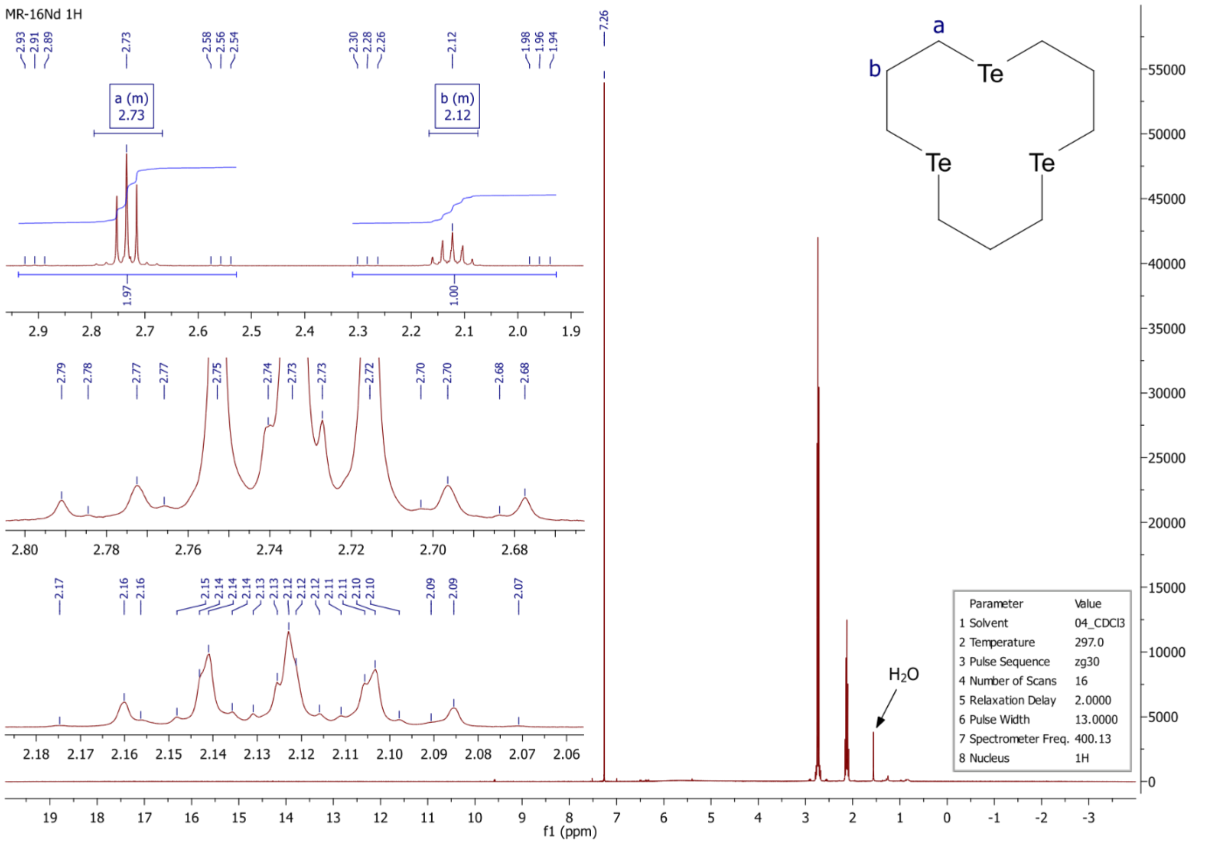

Figure S10. ^{1}H-NMR spectrum of 1,5,9-$Te_3(CH_2)_9$.

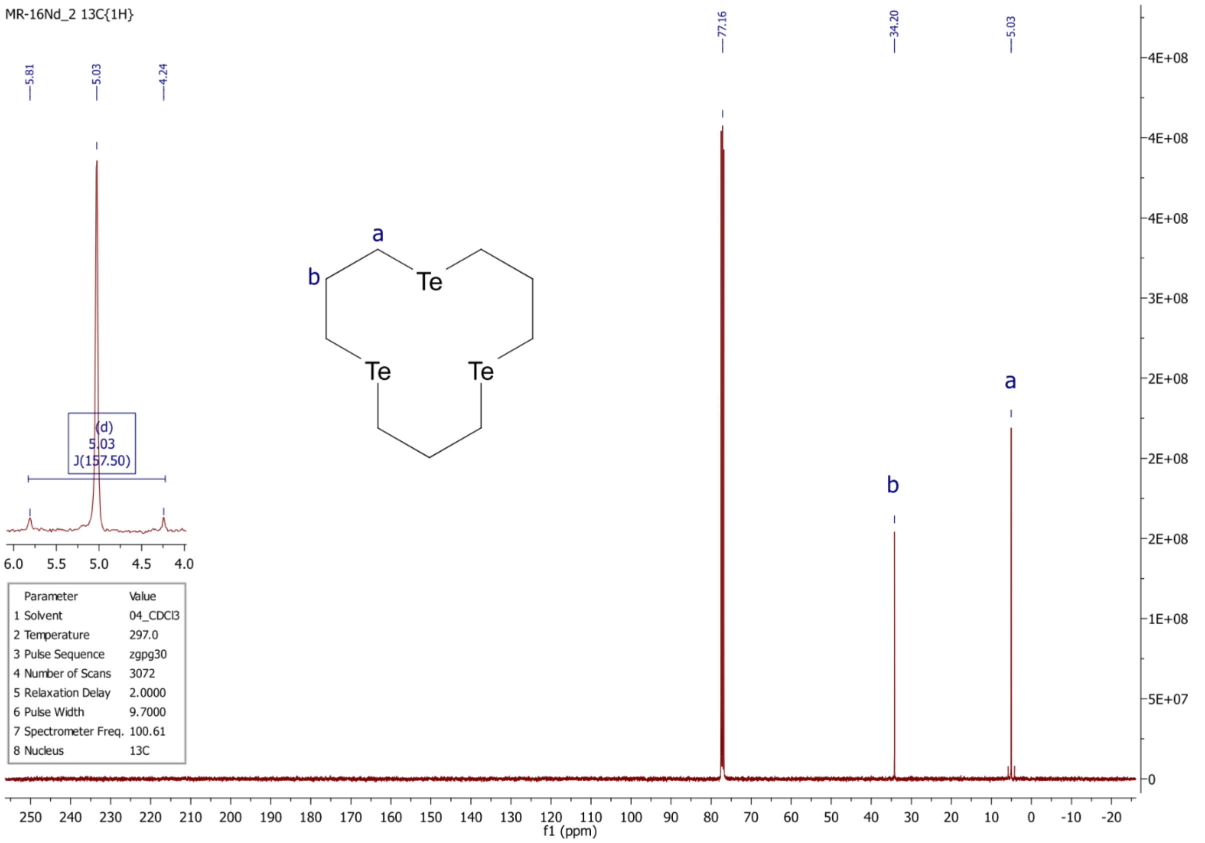

Figure S11. ^{13}C{^{1}H}-NMR spectrum of 1,5,9-$Te_3(CH_2)_9$.

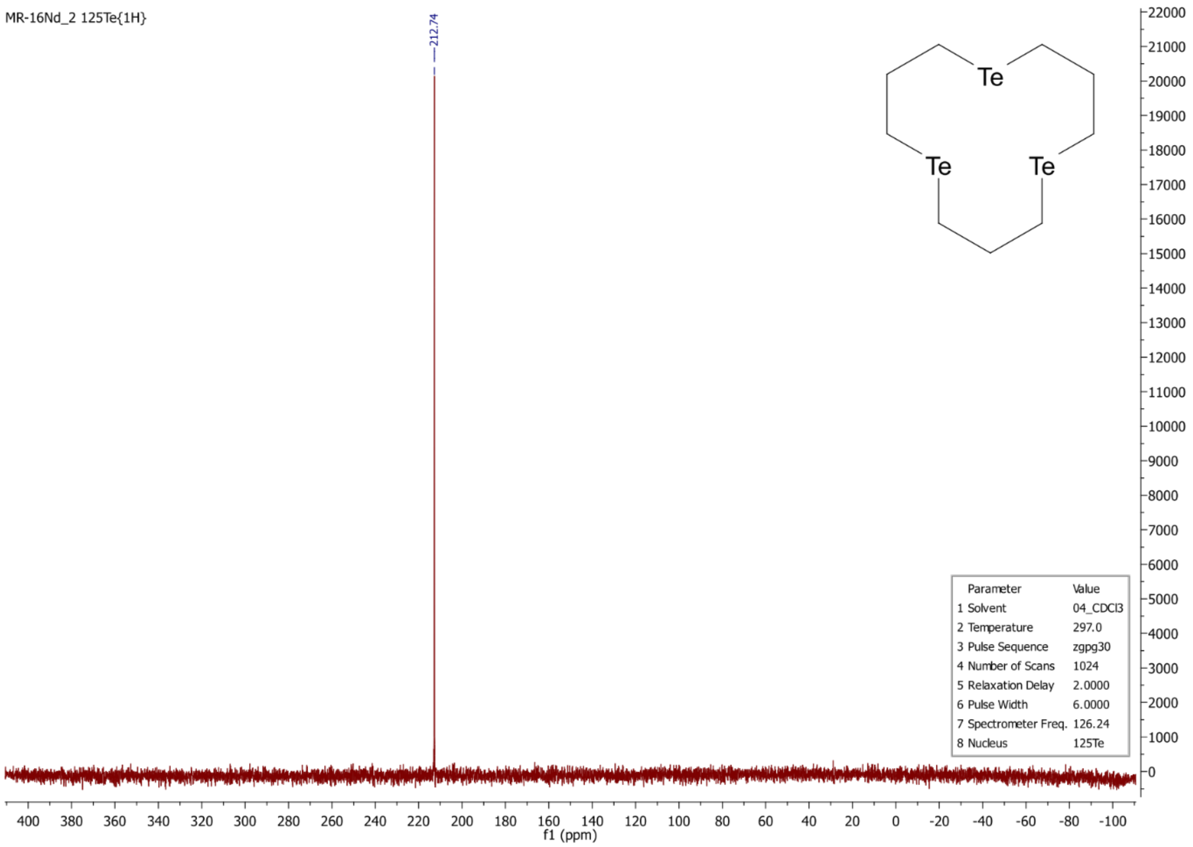

Figure S12. $^{125}Te\{^1H\}$-NMR spectrum of 1,5,9-$Te_3(CH_2)_9$.

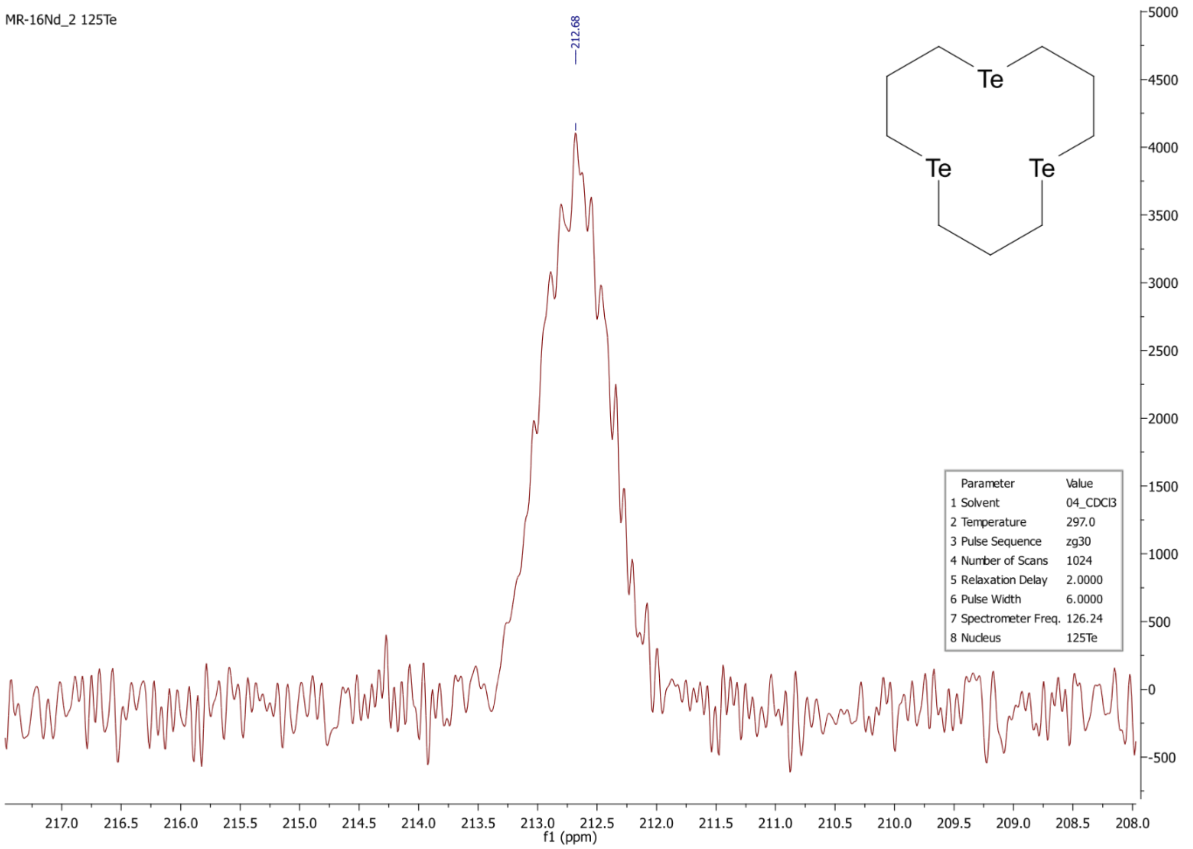

Figure S13. ^{125}Te-NMR spectrum of 1,5,9-$Te_3(CH_2)_9$.

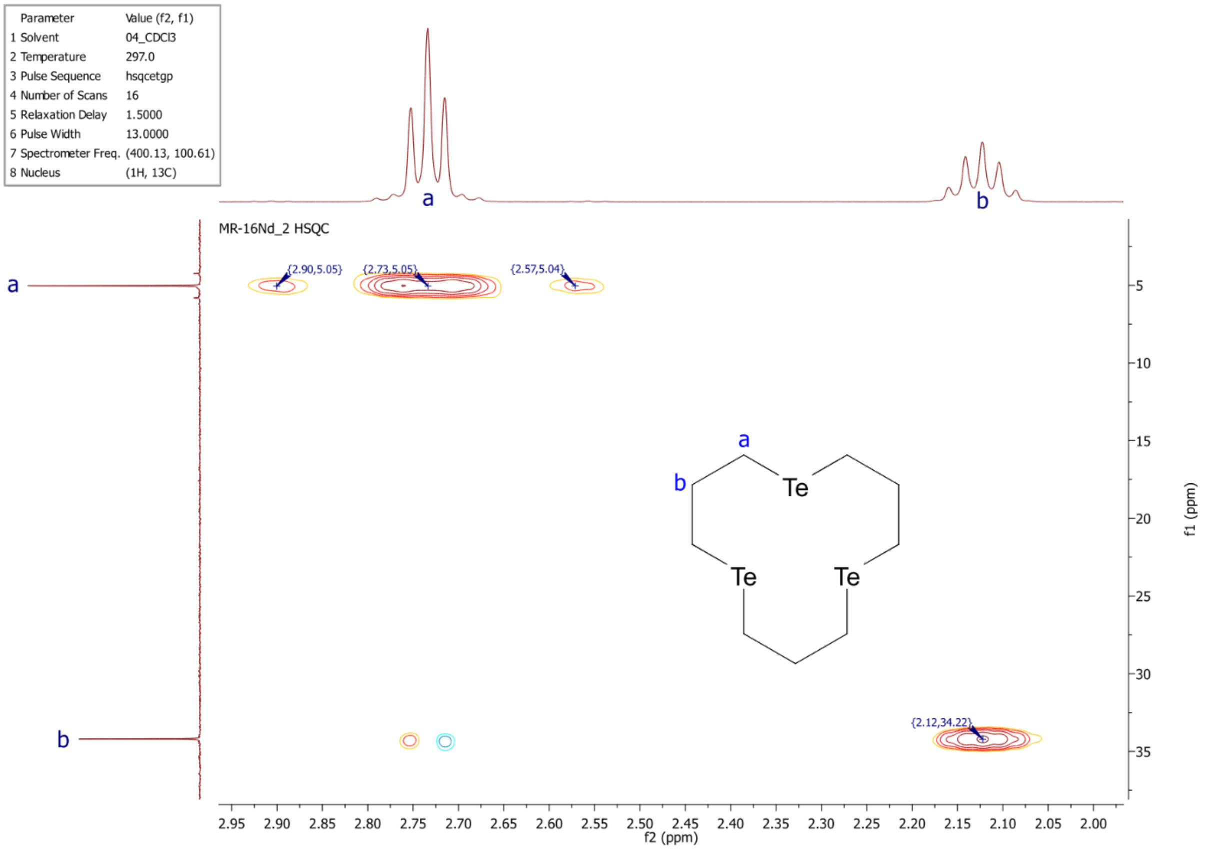

Figure S14. $^{1}H,^{13}C$-HSQC-NMR spectrum of 1,5,9-$Te_3(CH_2)_9$.

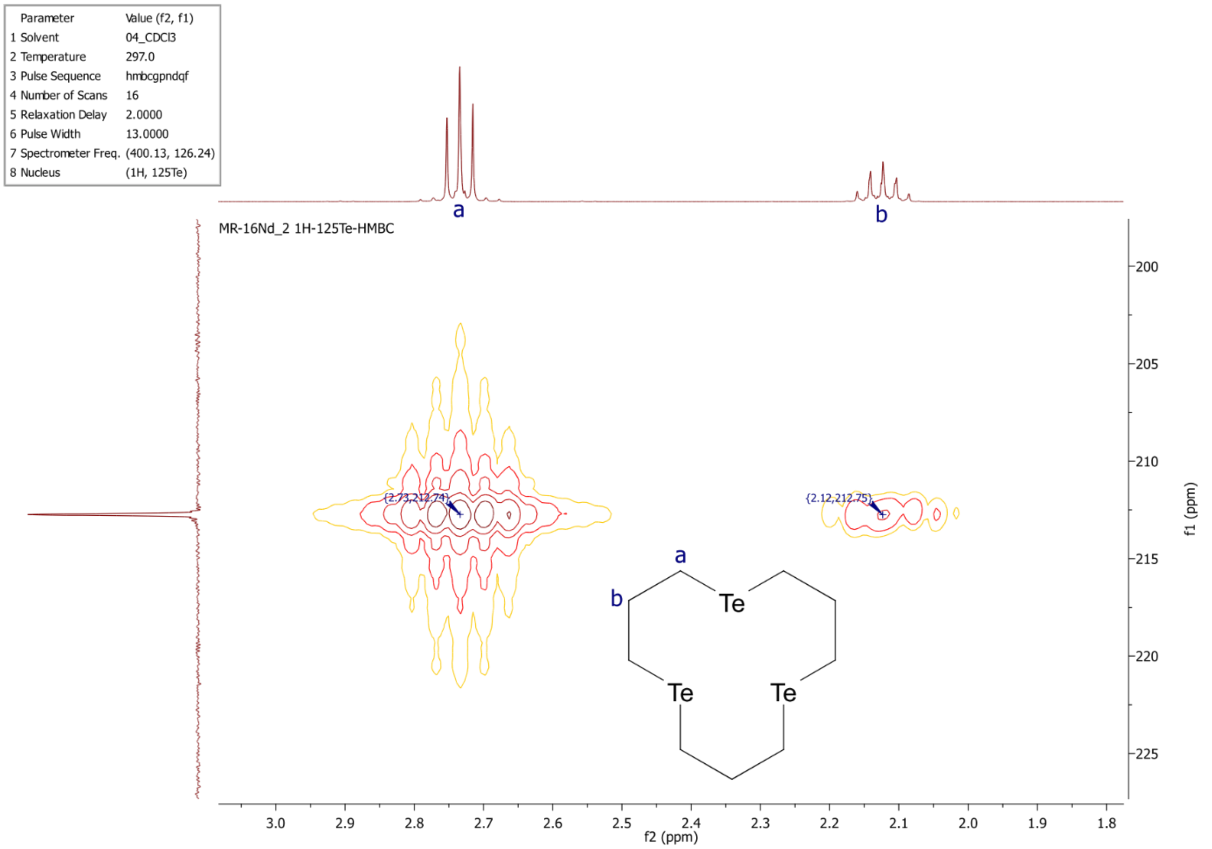

Figure S15. $^{1}H,^{125}Te$-HMBC-NMR spectrum of 1,5,9-$Te_3(CH_2)_9$.

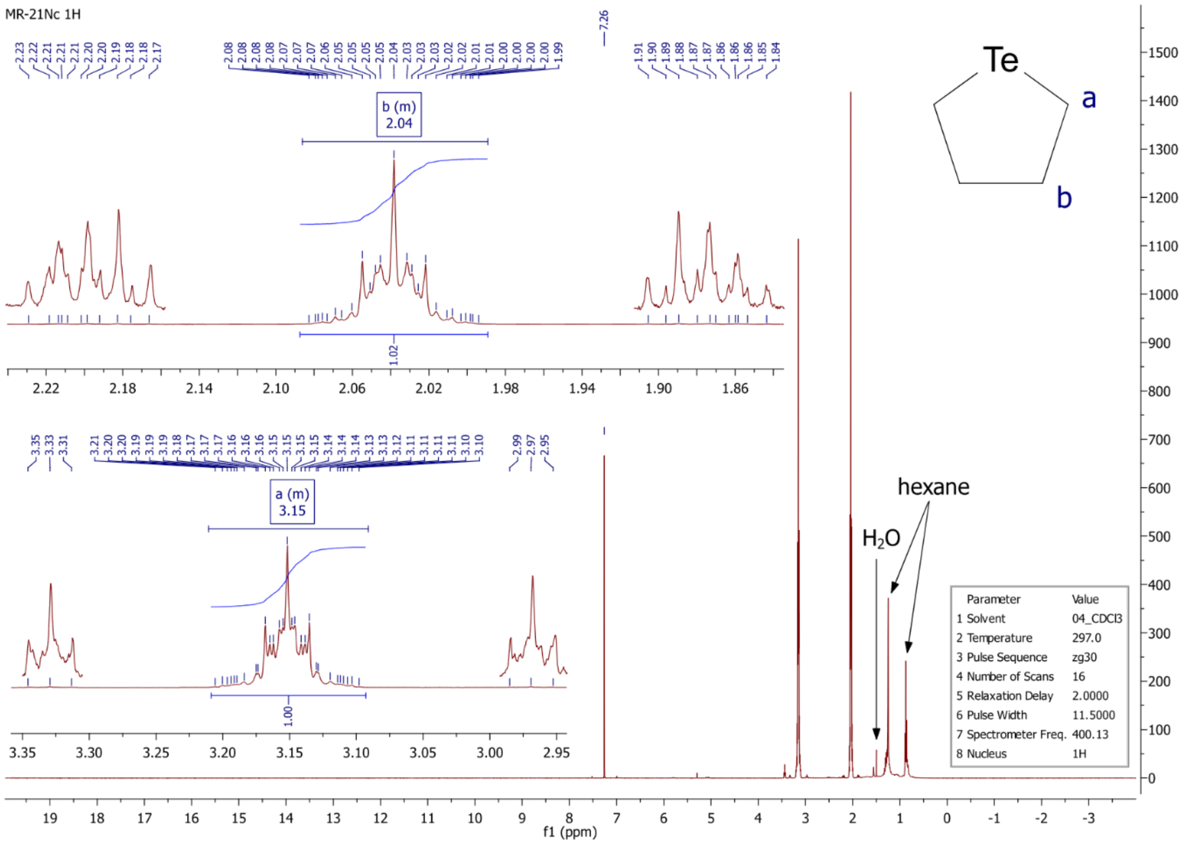

Figure S16. ^{1}H-NMR spectrum of $Te(CH_2)_4$.

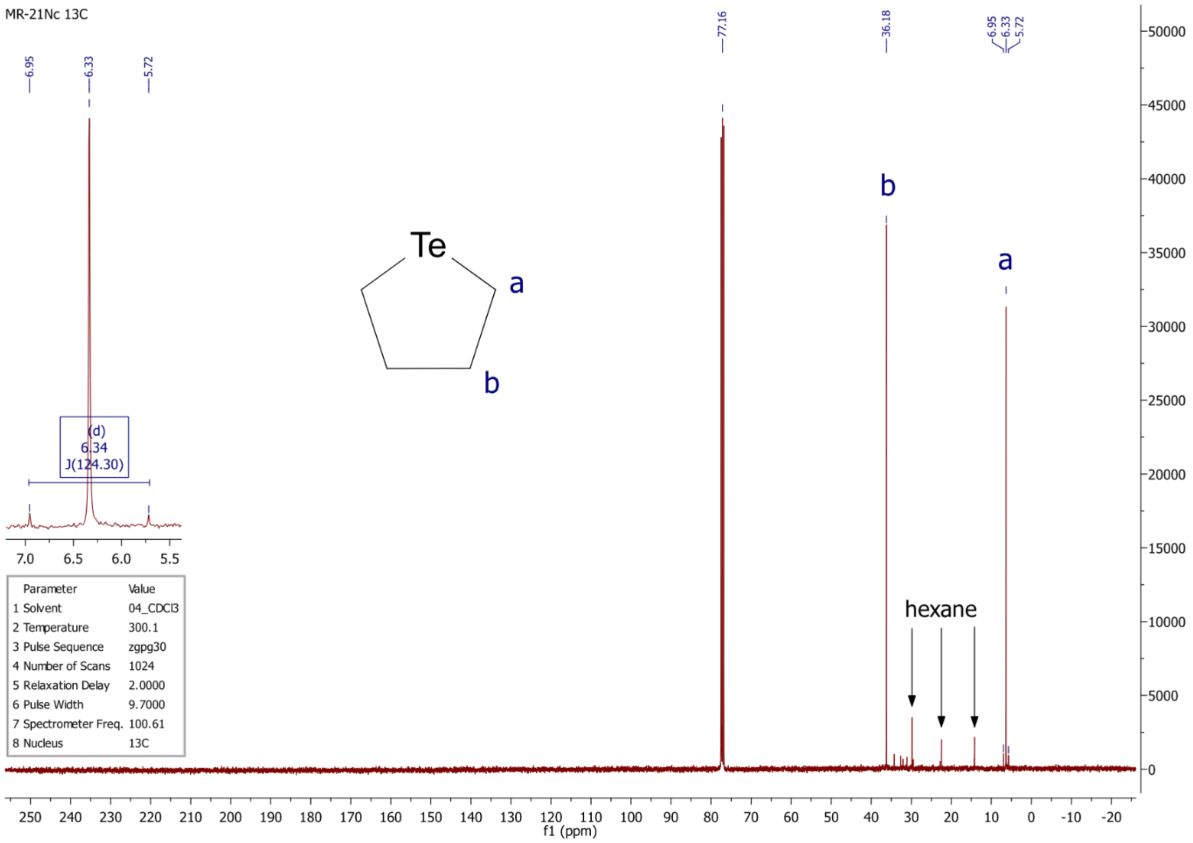

Figure S17. $^{13}C\{^{1}H\}$-NMR spectrum of $Te(CH_2)_4$.

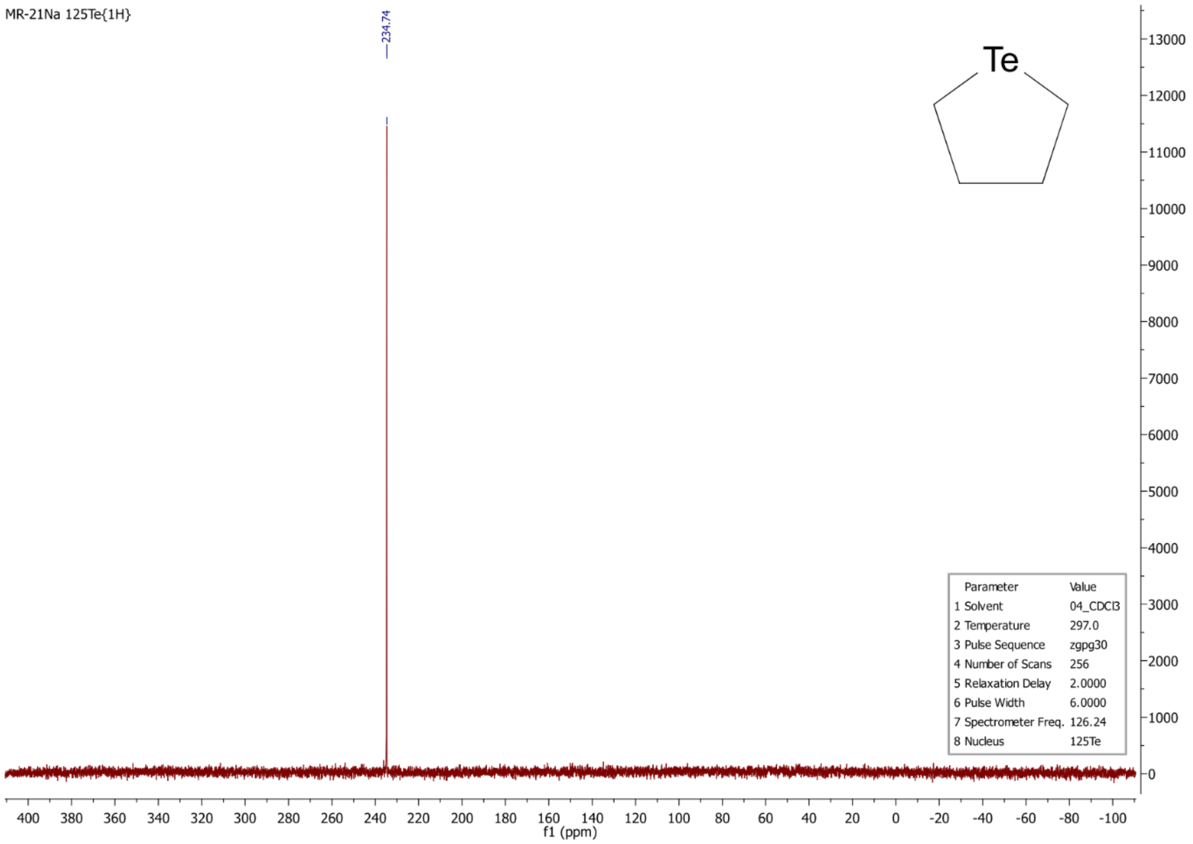

Figure S18. ^{125}Te{^{1}H}-NMR spectrum of $Te(CH_2)_4$.

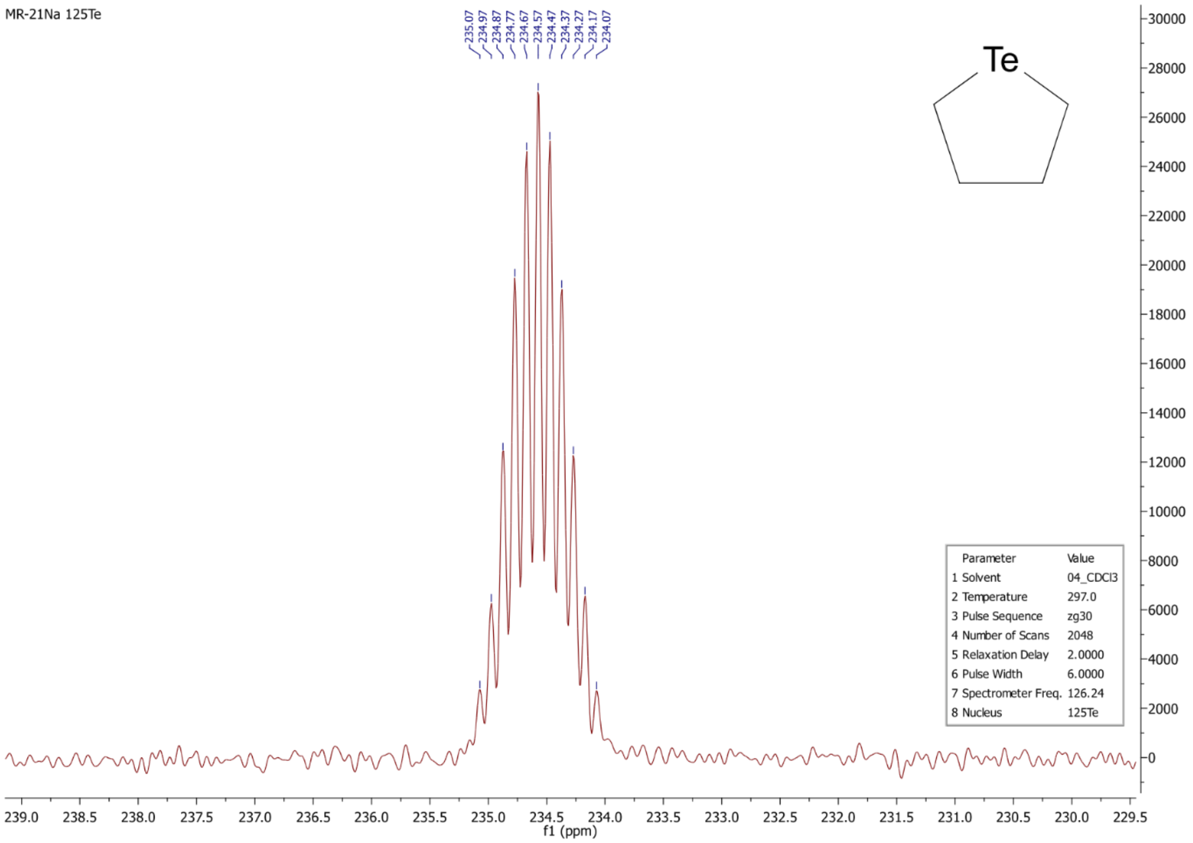

Figure S19. ^{125}Te-NMR spectrum of $Te(CH_2)_4$.

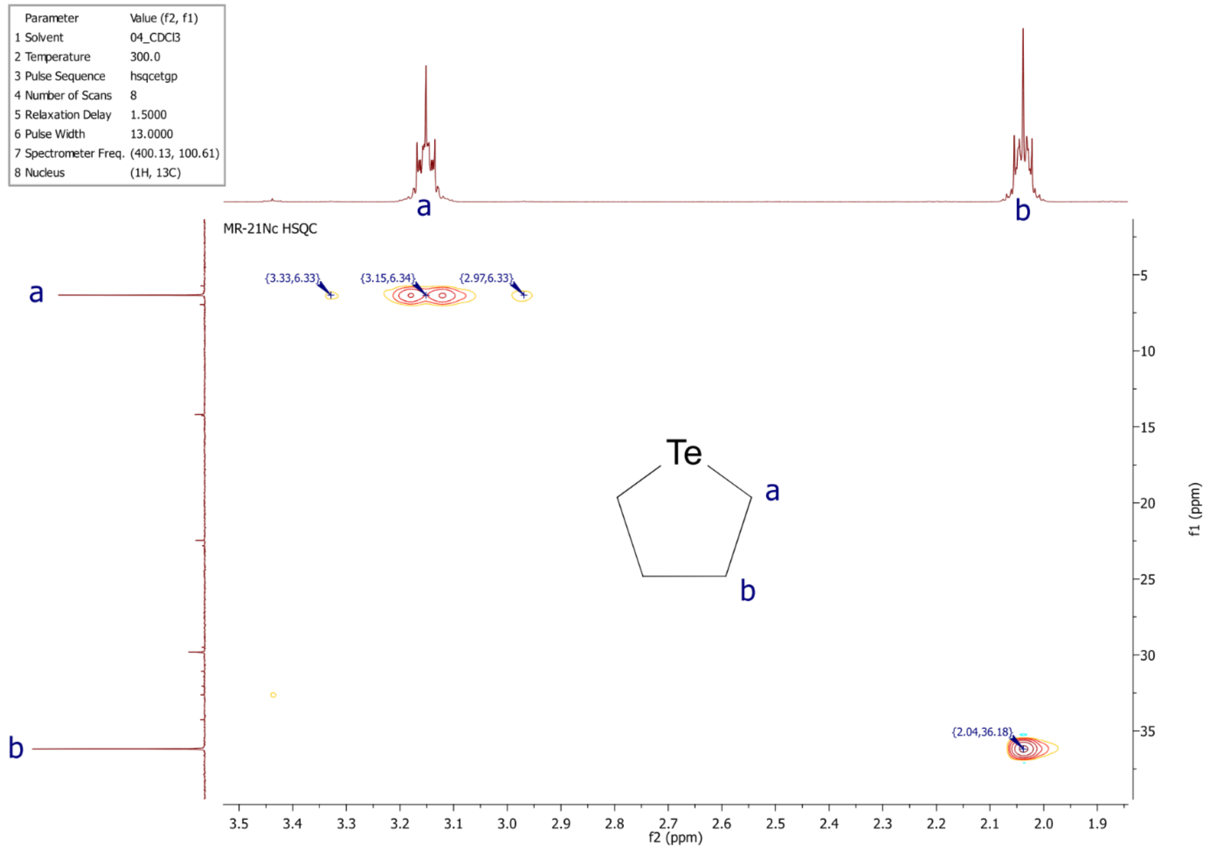

Figure S20. $^1H,^{13}C$-HSQC-NMR spectrum of $Te(CH_2)_4$.

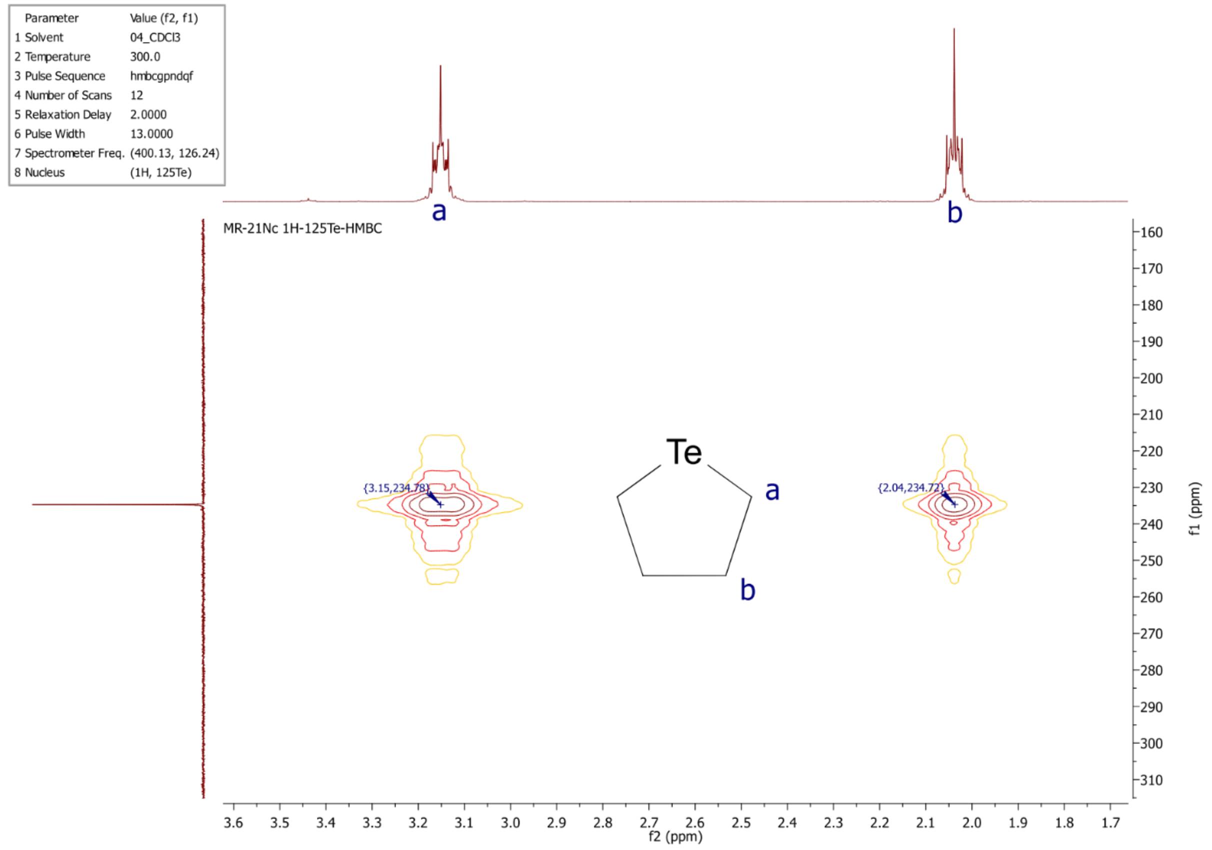

Figure S21. $^1H,^{125}Te$-HMBC-NMR spectrum of $Te(CH_2)_4$.

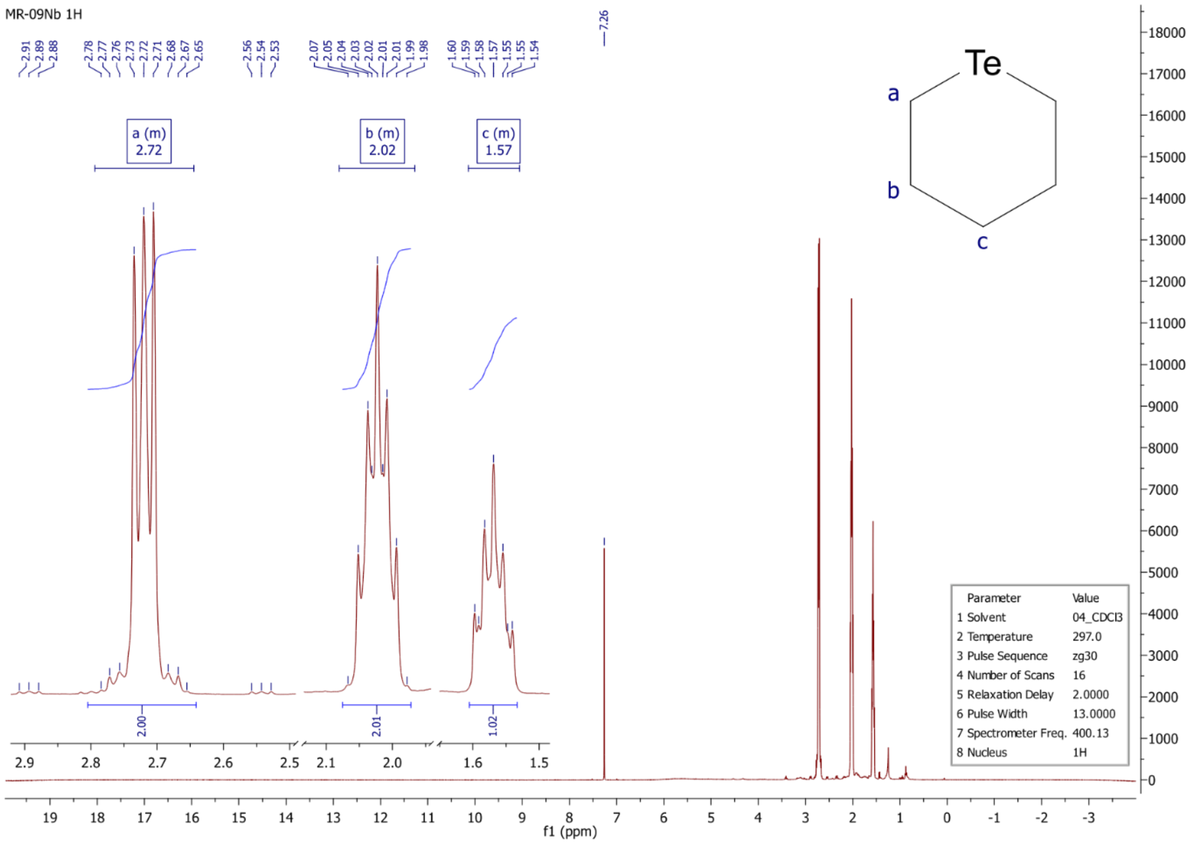

Figure S22. ^{1}H-NMR spectrum of $Te(CH_2)_5$.

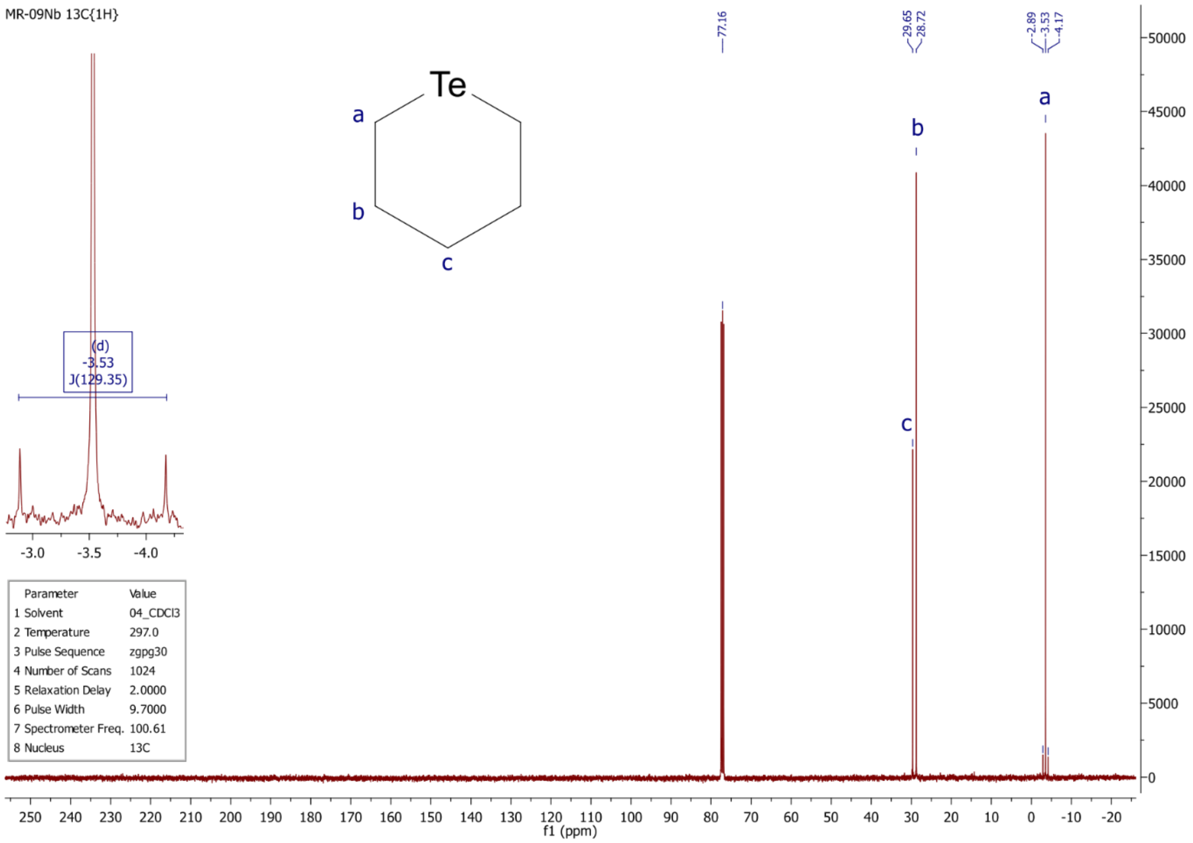

Figure S23. $^{13}C\{^{1}H\}$-NMR spectrum of $Te(CH_2)_5$.

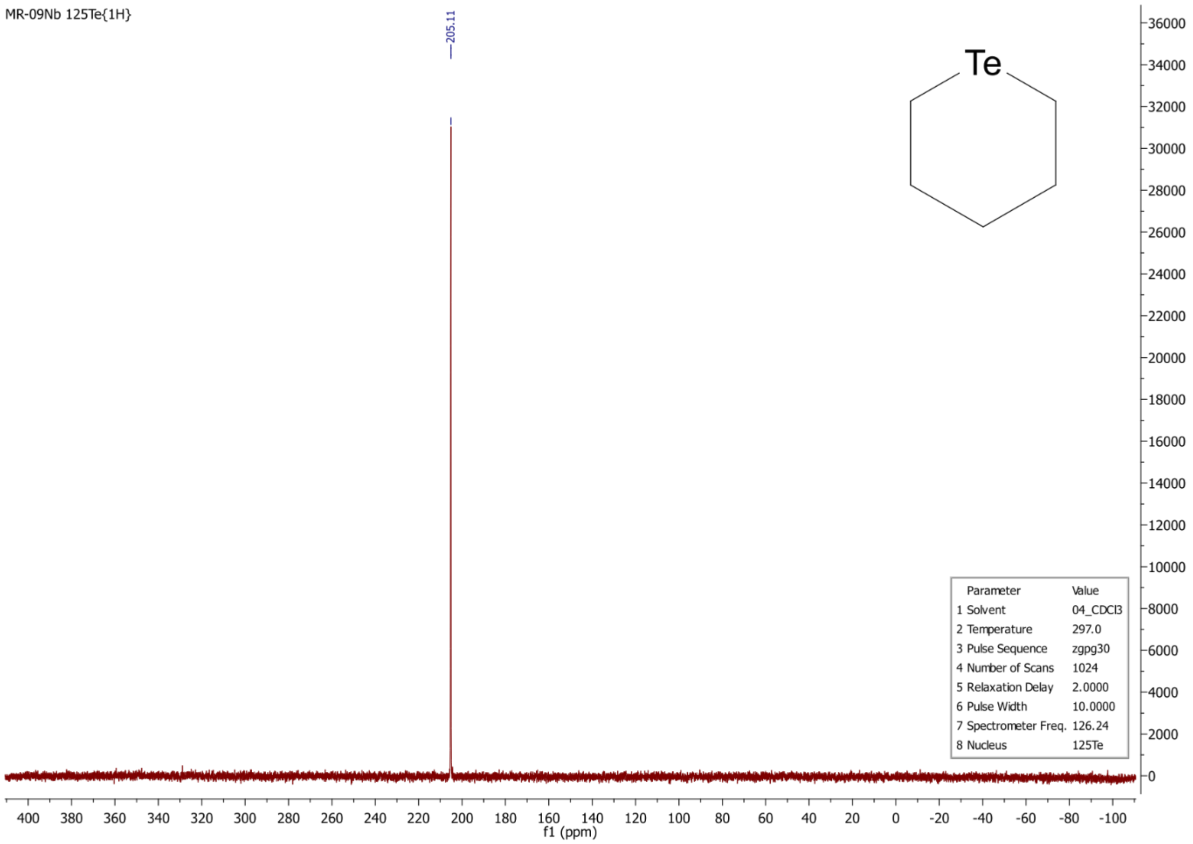

Figure S24. $^{125}Te\{^{1}H\}$-NMR spectrum of $Te(CH_2)_5$.

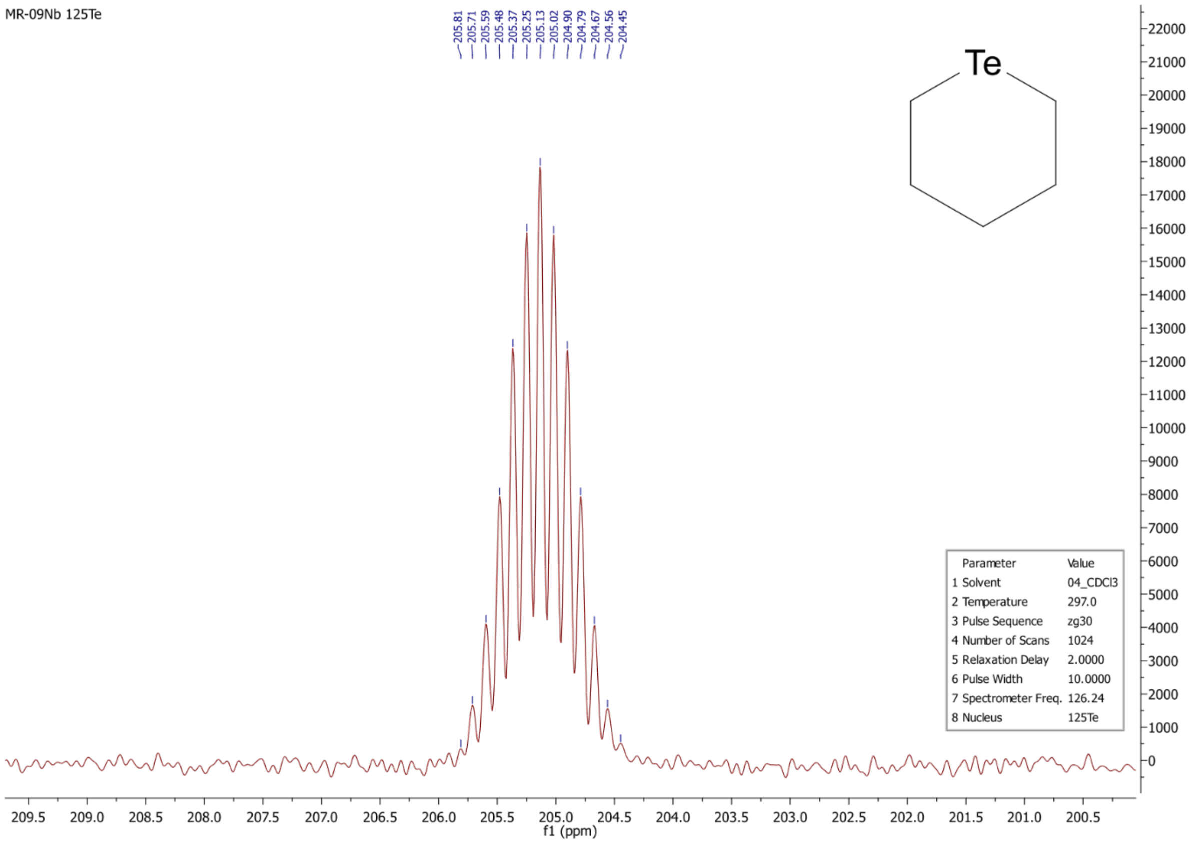

Figure S25. ^{125}Te-NMR spectrum of $Te(CH_2)_5$.

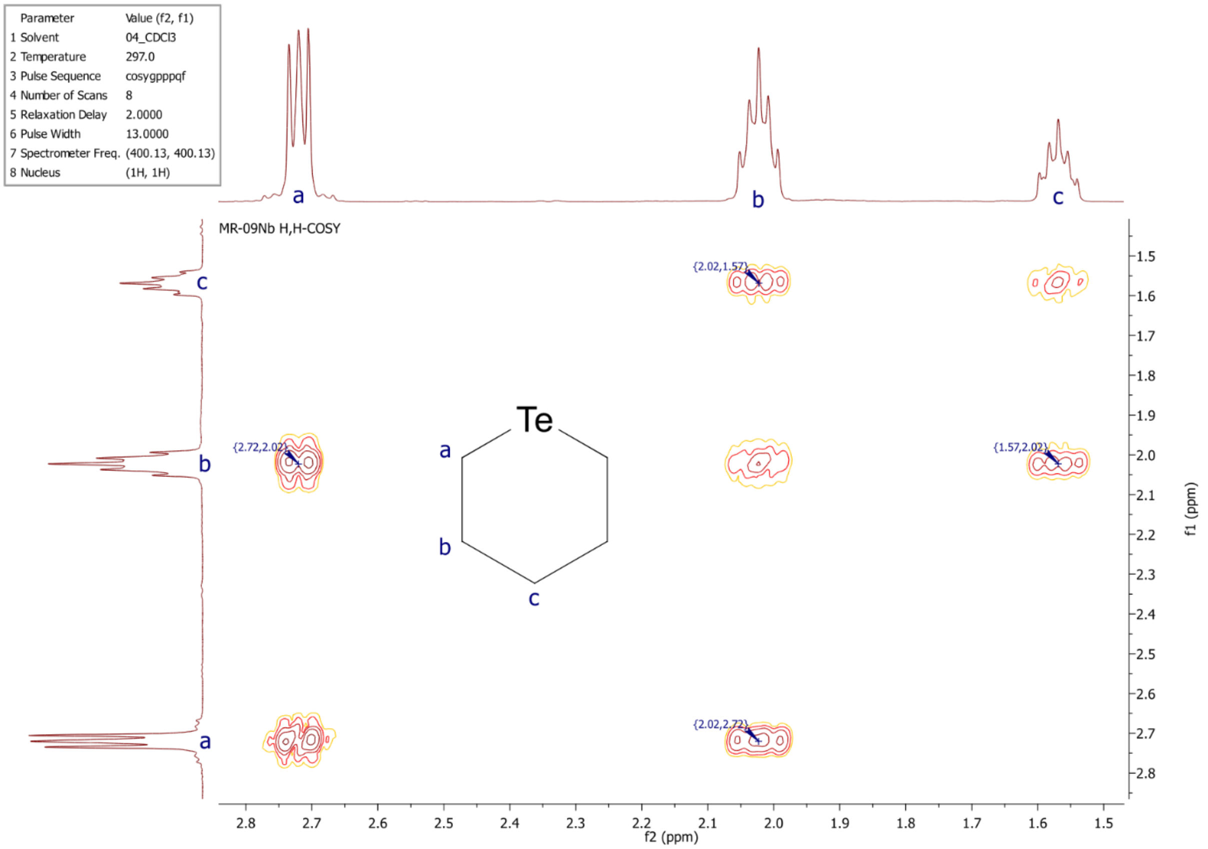

Figure S26. ^{1}H,^{1}H-COSY-NMR spectrum of $Te(CH_2)_5$.

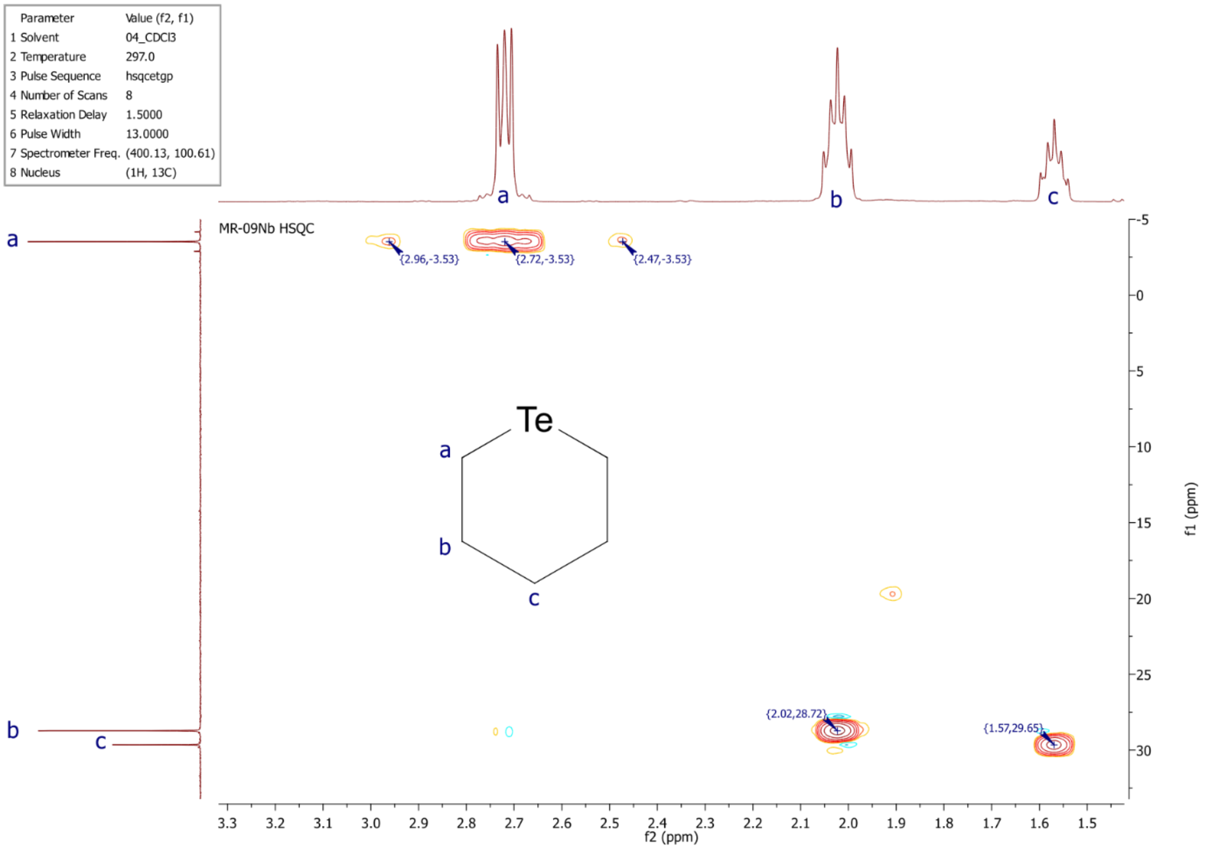

Figure S27. ^{1}H,^{13}C-HSQC-NMR spectrum of $Te(CH_2)_5$.

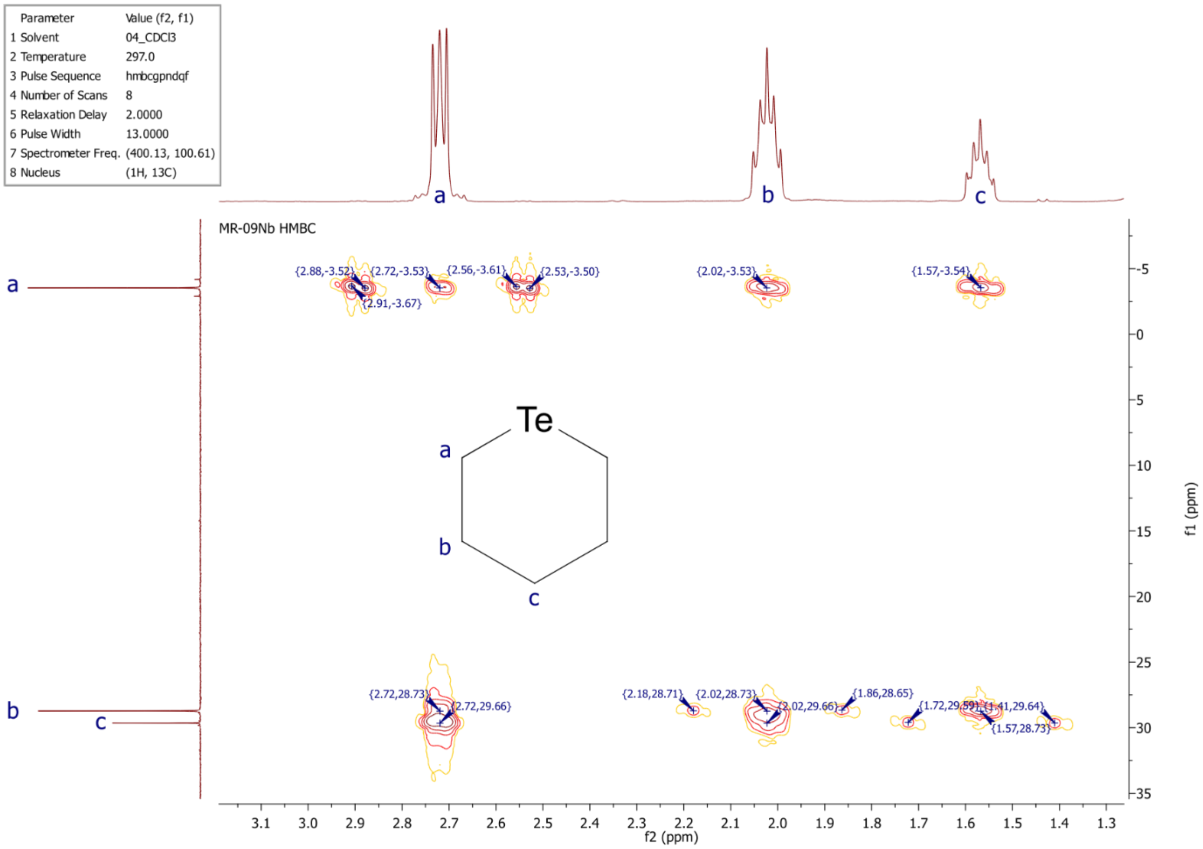

Figure S28. $^{1}H,^{13}C$-HMBC-NMR spectrum of $Te(CH_2)_5$.

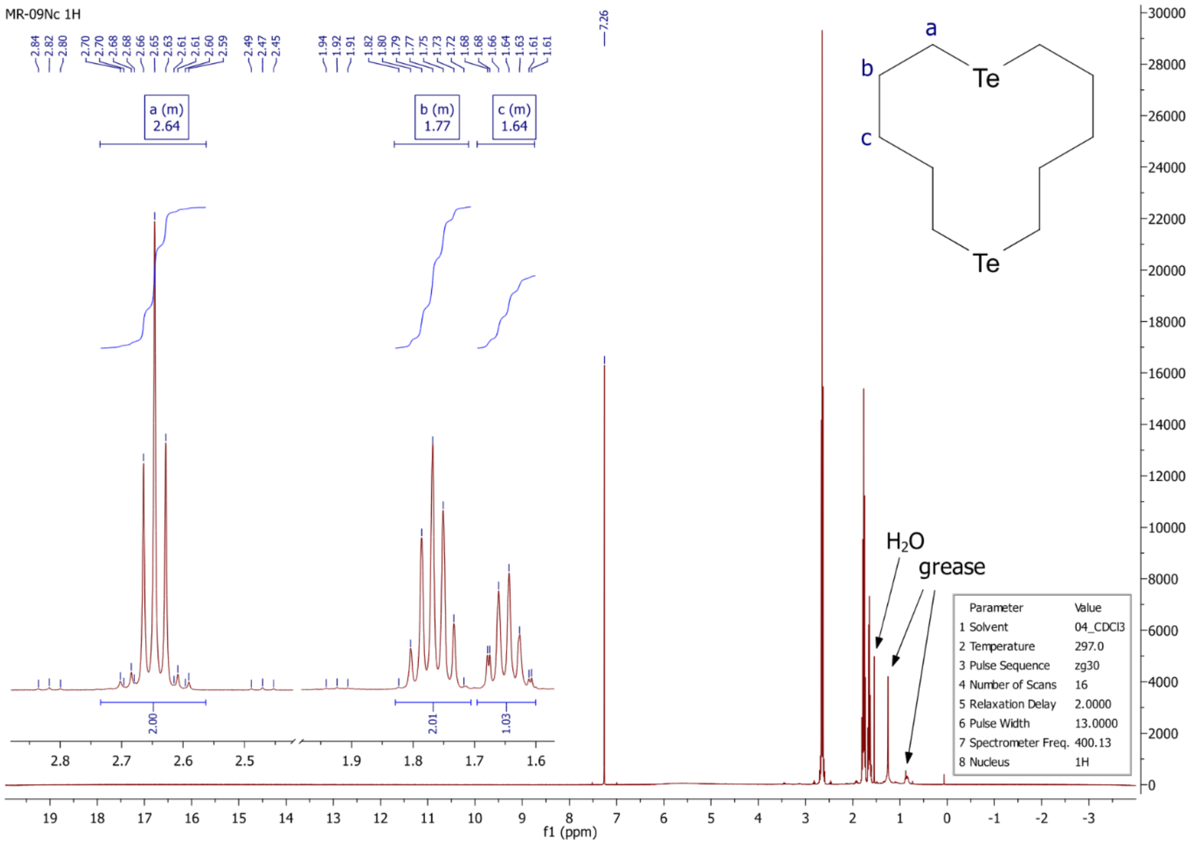

Figure S29. ^{1}H-NMR spectrum of 1,7-$Te_2(CH_2)_{10}$.

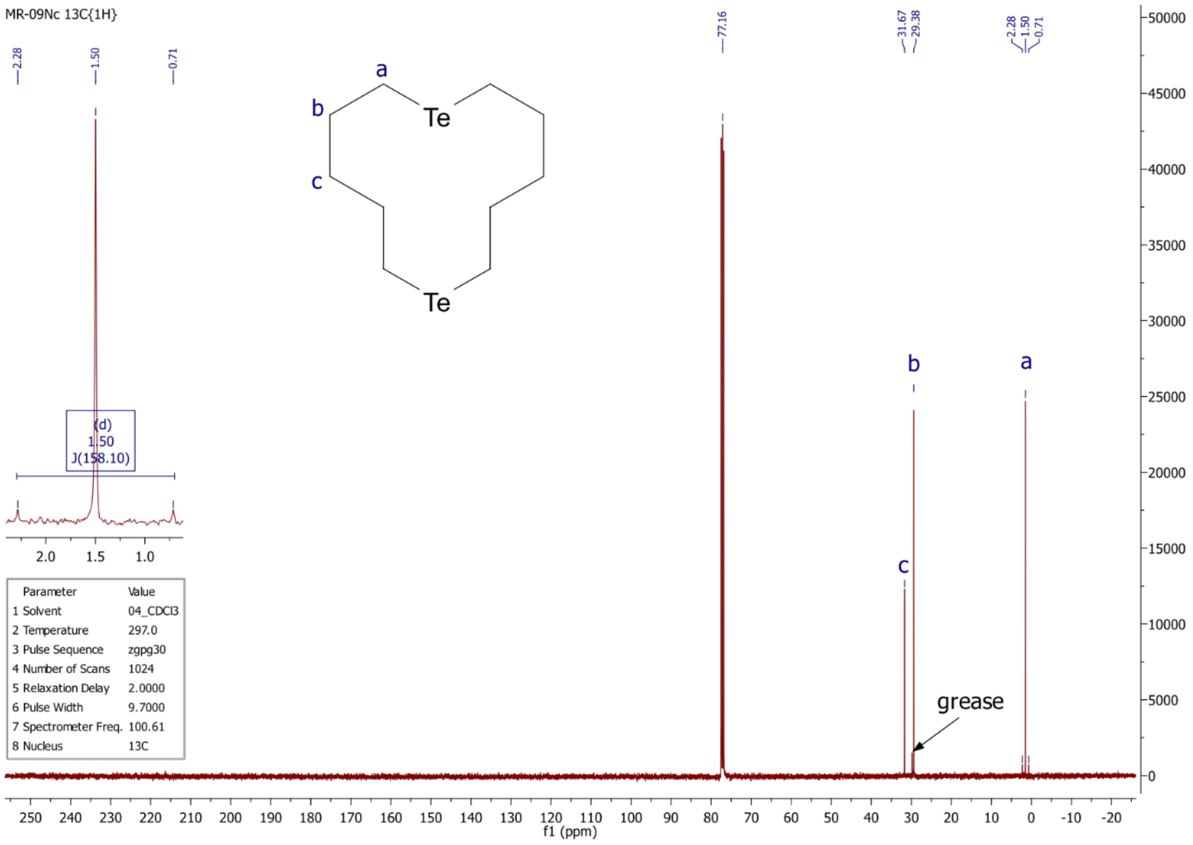

Figure S30. $^{13}C\{^{1}H\}$-NMR spectrum of 1,7-$Te_2(CH_2)_{10}$.

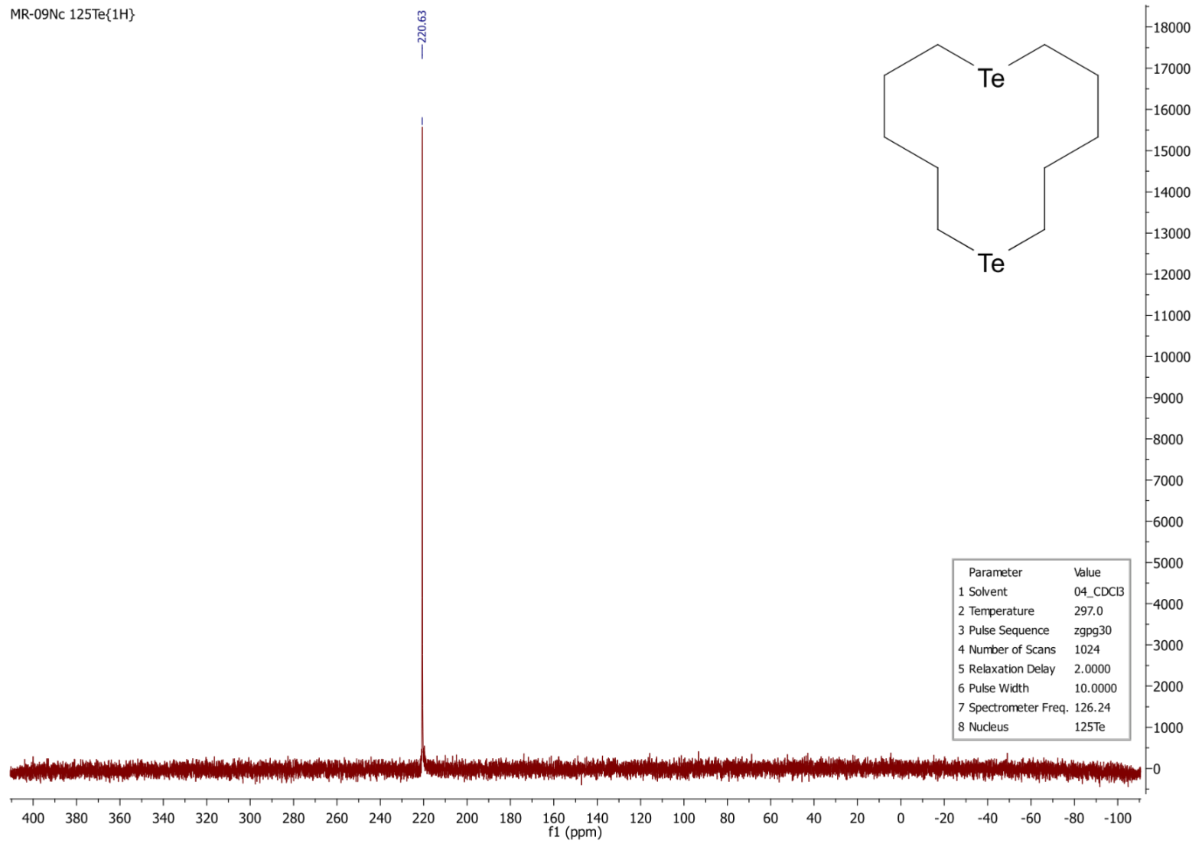

Figure S31. ^{125}Te{^{1}H}-NMR spectrum of 1,7-$Te_2(CH_2)_{10}$.

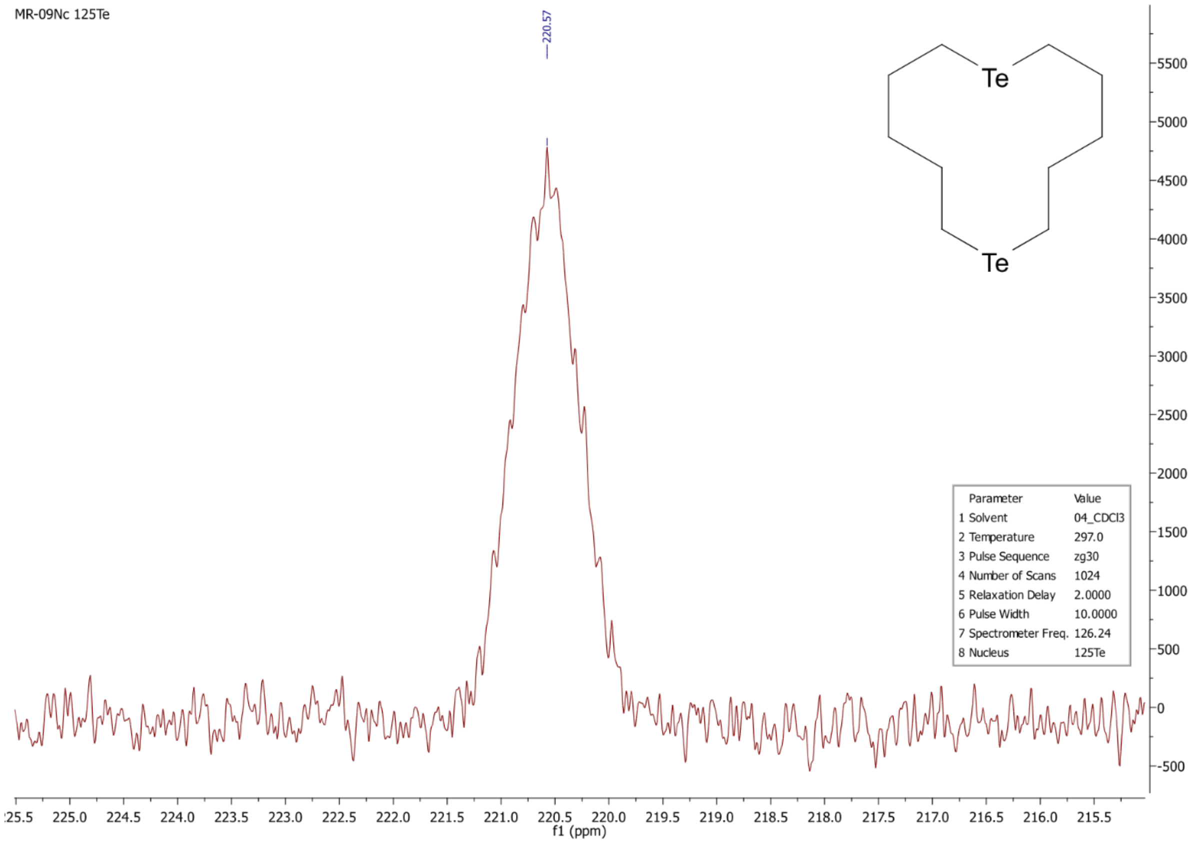

Figure S32. ^{125}Te-NMR spectrum of 1,7-$Te_2(CH_2)_{10}$.

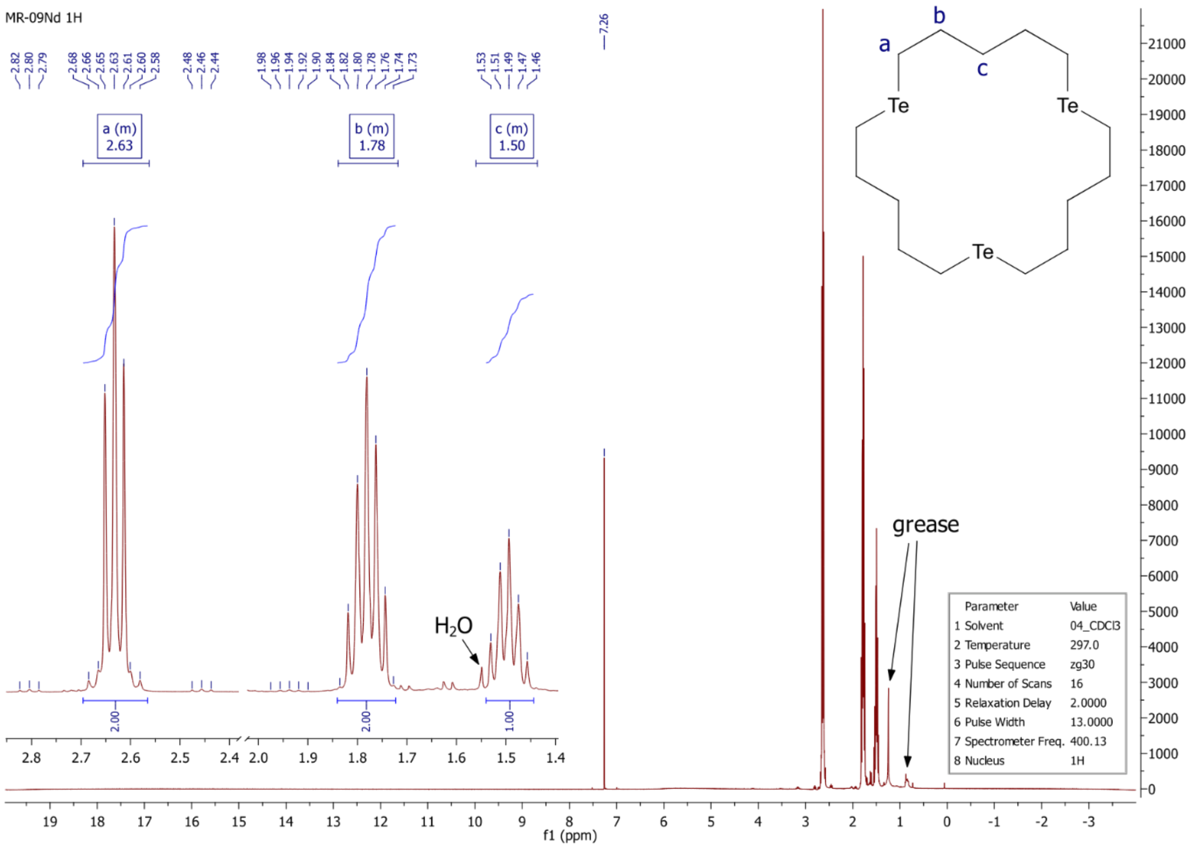

Figure S33. ^{1}H-NMR spectrum of 1,7,13-$Te_3(CH_2)_{15}$.

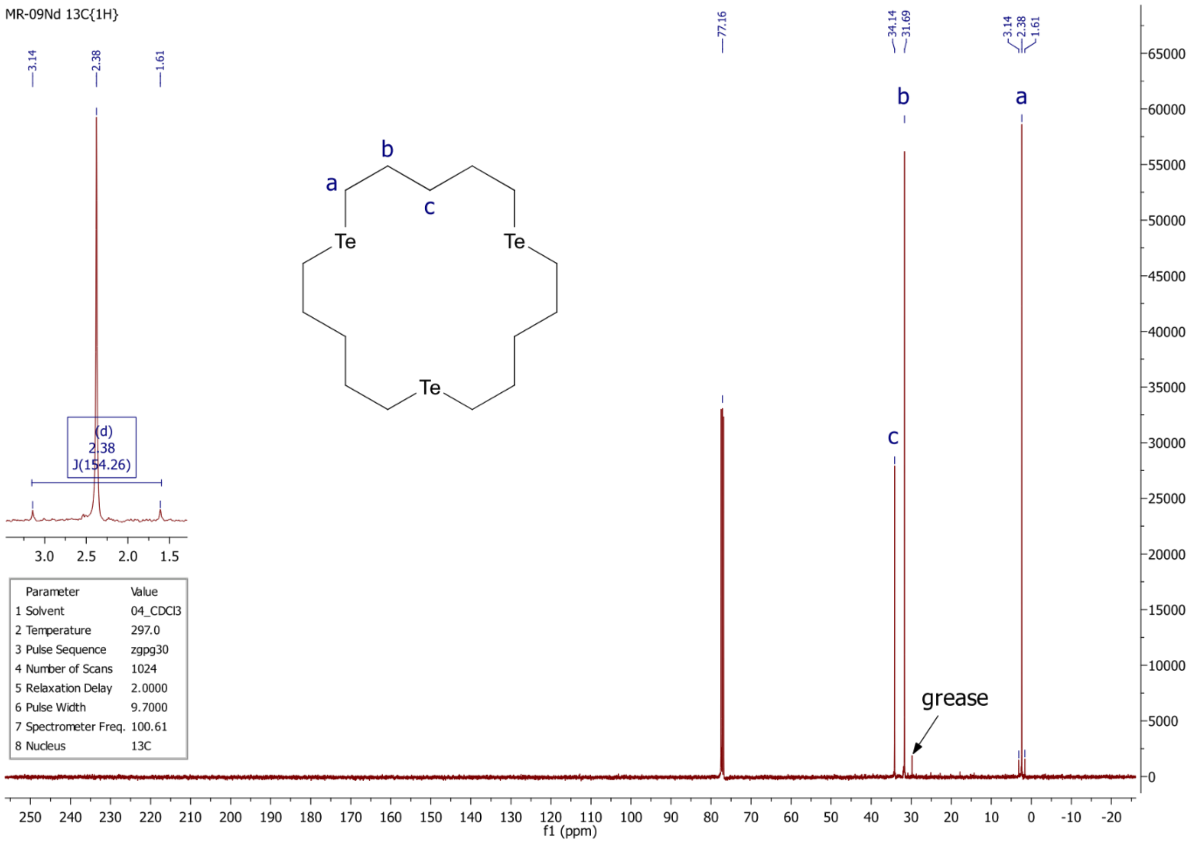

Figure S34. $^{13}C\{^{1}H\}$-NMR spectrum of 1,7,13-$Te_3(CH_2)_{15}$.

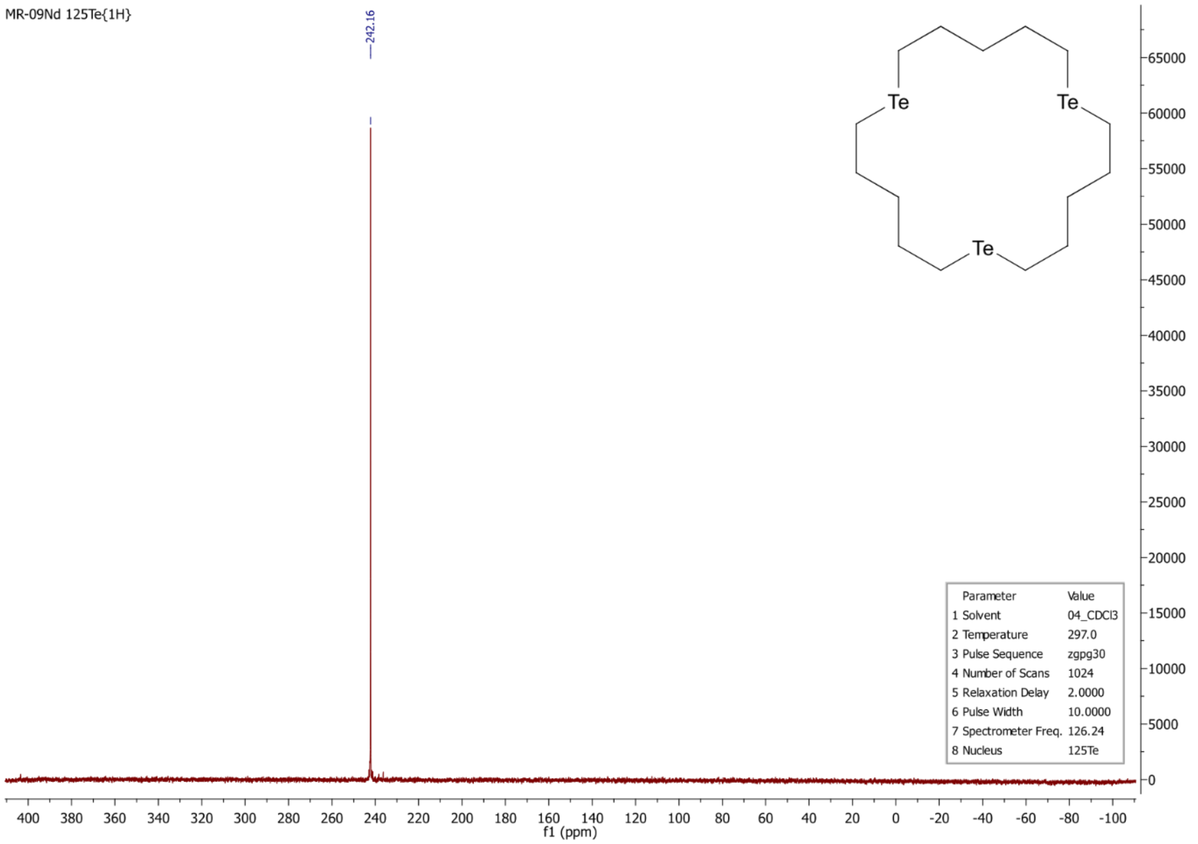

Figure S35. $^{125}Te\{^{1}H\}$-NMR spectrum of 1,7,13-$Te_3(CH_2)_{15}$.

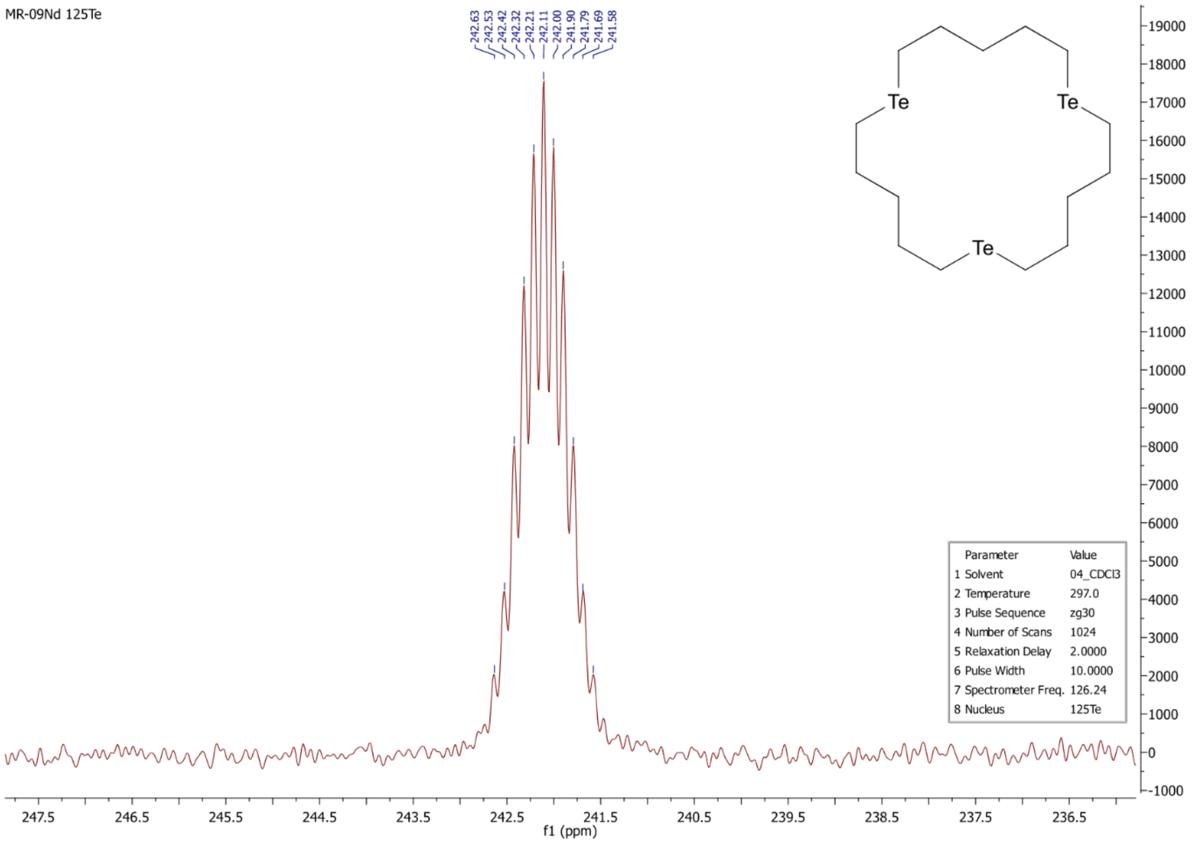

Figure S36. ^{125}Te-NMR spectrum of 1,7,13-$Te_3(CH_2)_{15}$.

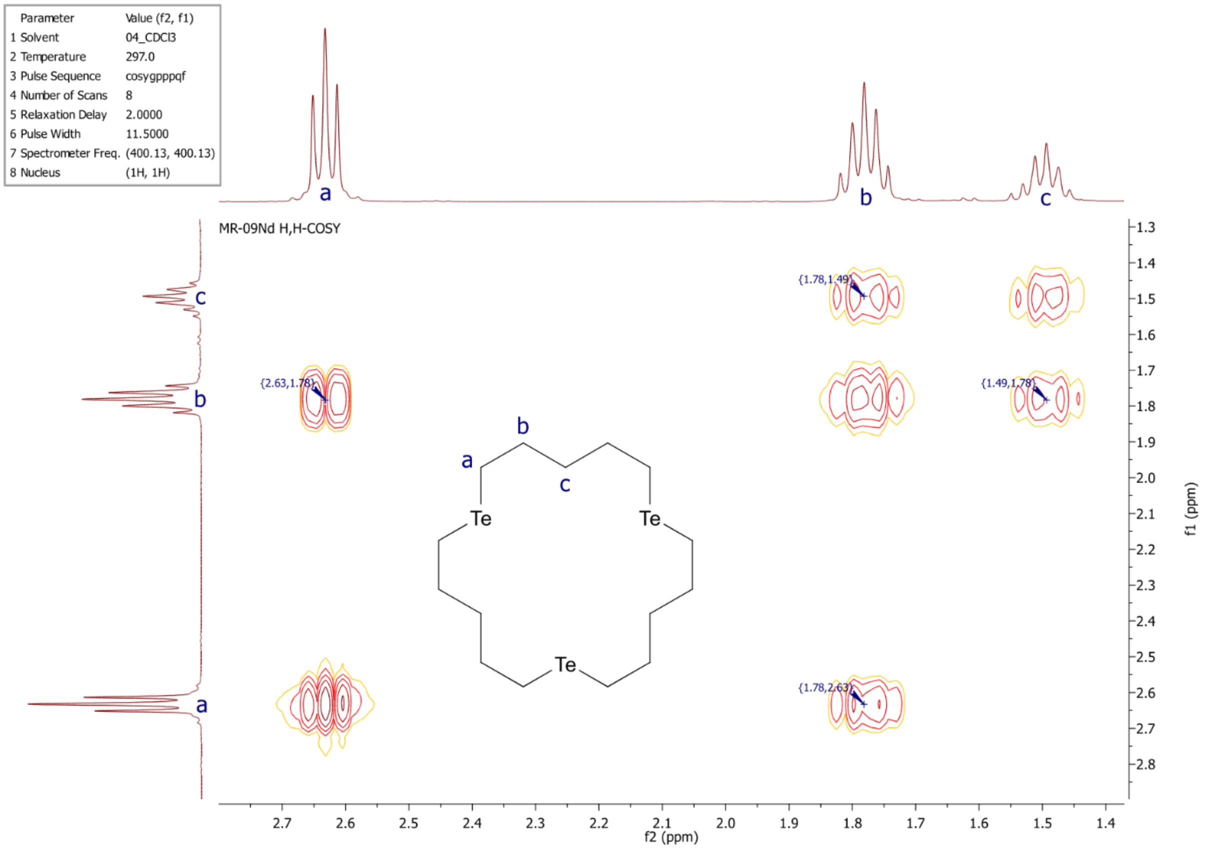

Figure S37. $^{1}H,^{1}H$-COSY-NMR spectrum of 1,7,13-$Te_3(CH_2)_{15}$.

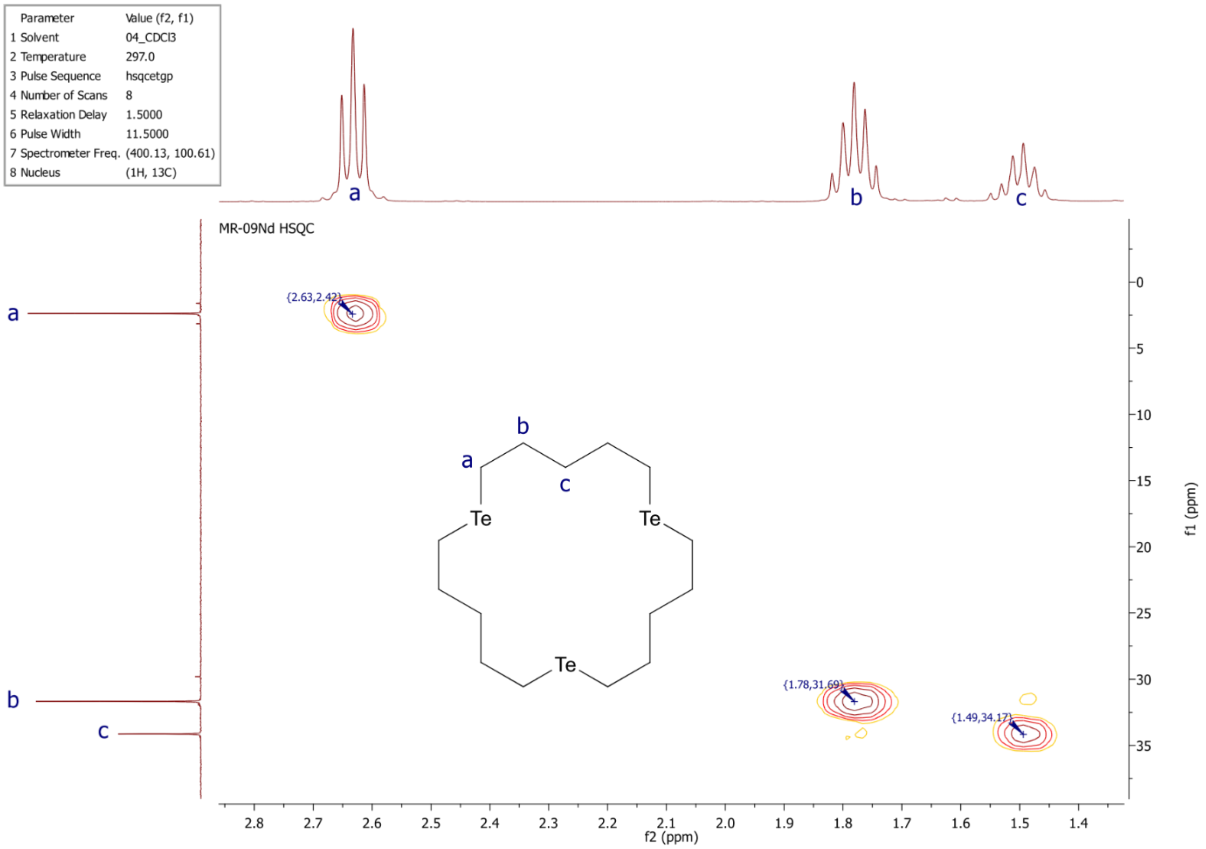

Figure S38. $^{1}H,^{13}C$-HSQC-NMR spectrum of 1,7,13-$Te_3(CH_2)_{15}$.

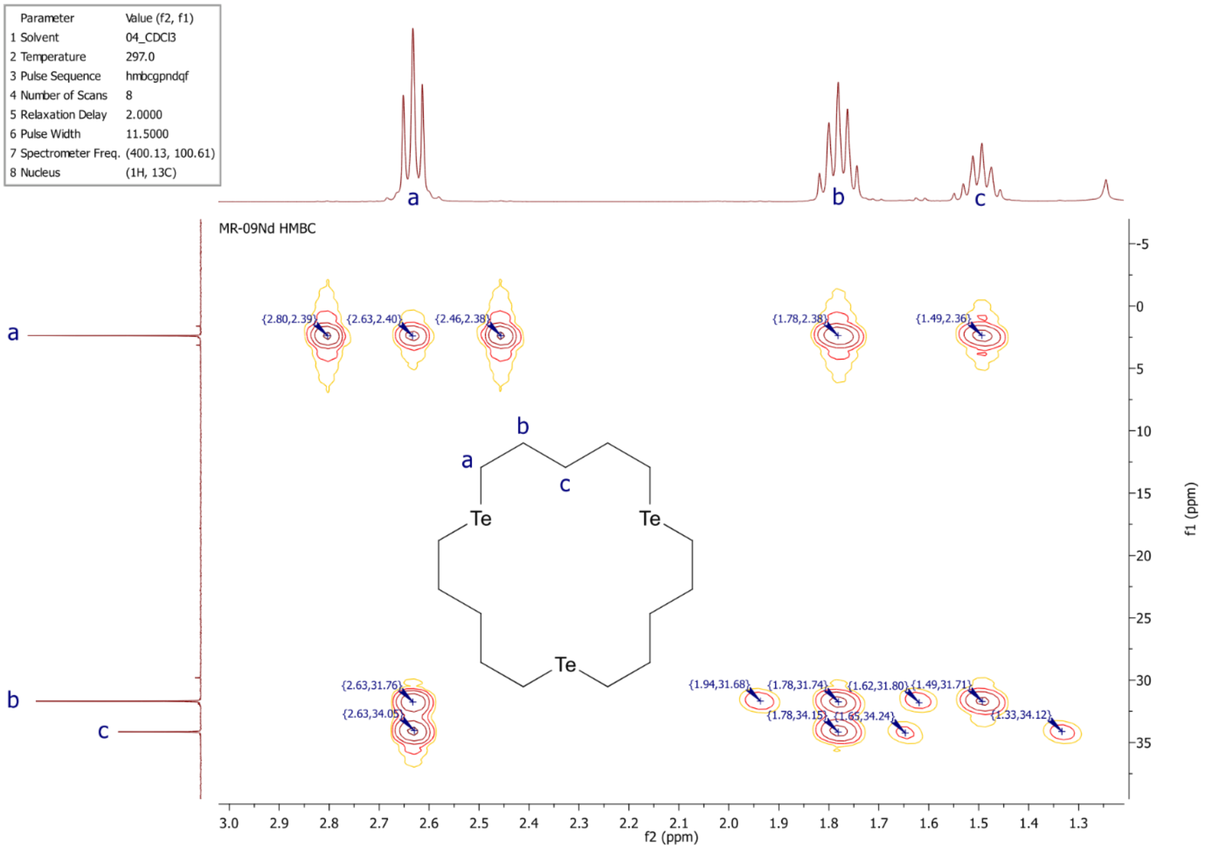

Figure S39. ^{1}H,^{13}C-HMBC-NMR spectrum of 1,7,13-$Te_3(CH_2)_{15}$.

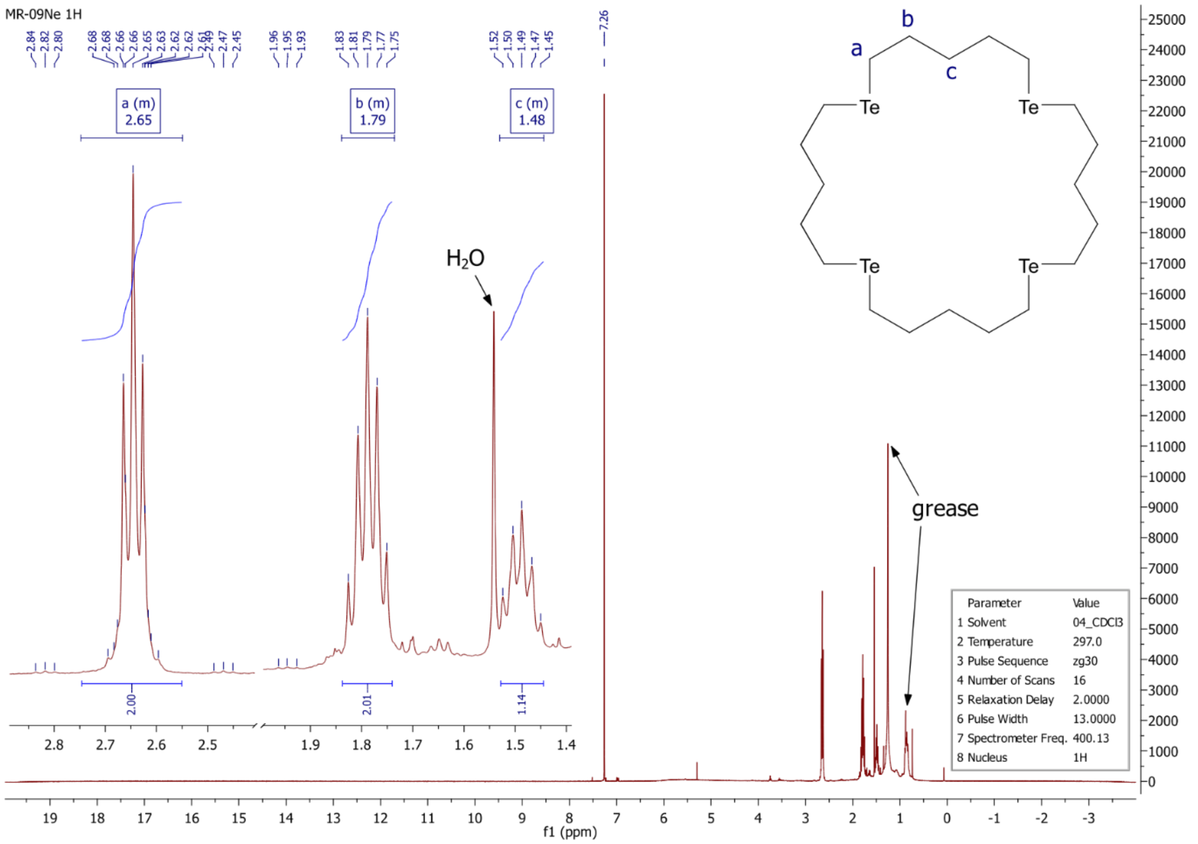

Figure S40. ^{1}H-NMR spectrum of 1,7,13,18-$Te_4(CH_2)_{20}$.

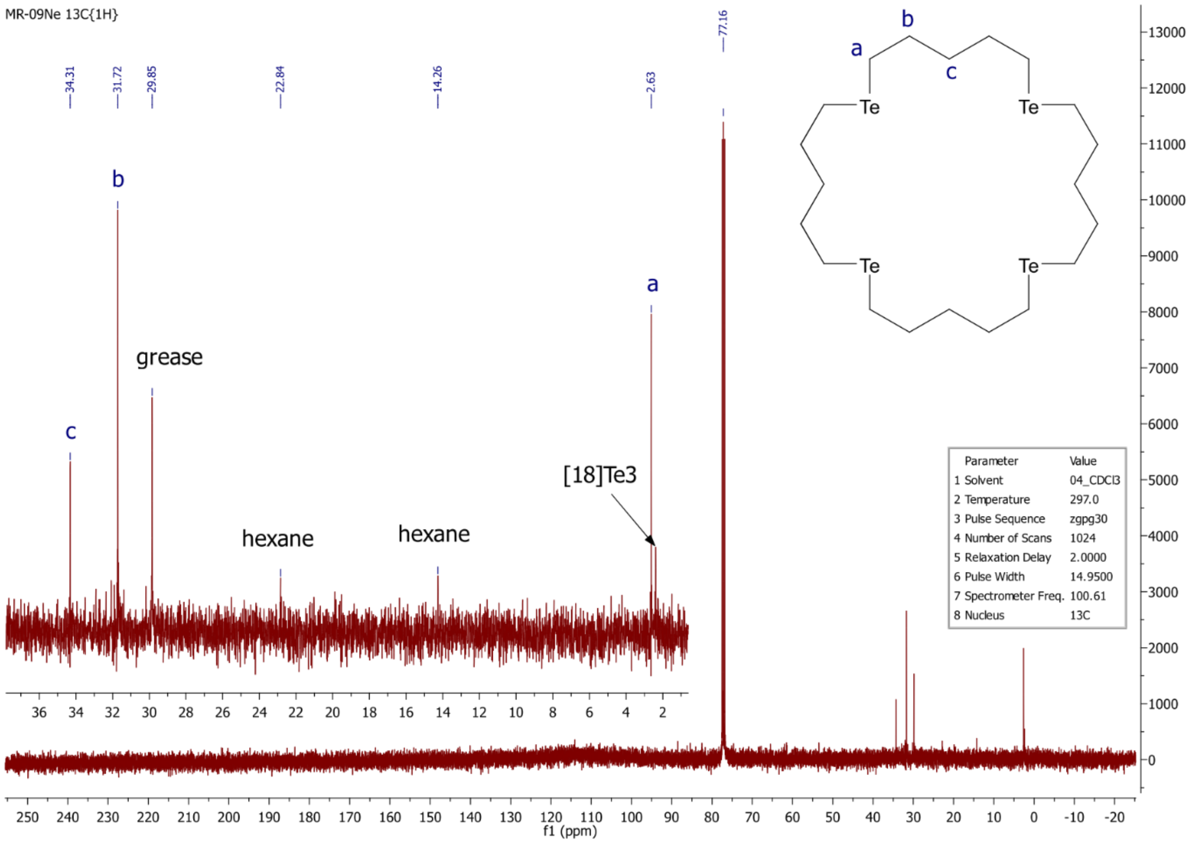

Figure S41. $^{13}C\{^{1}H\}$-NMR spectrum of 1,7,13,18-$Te_4(CH_2)_{20}$.

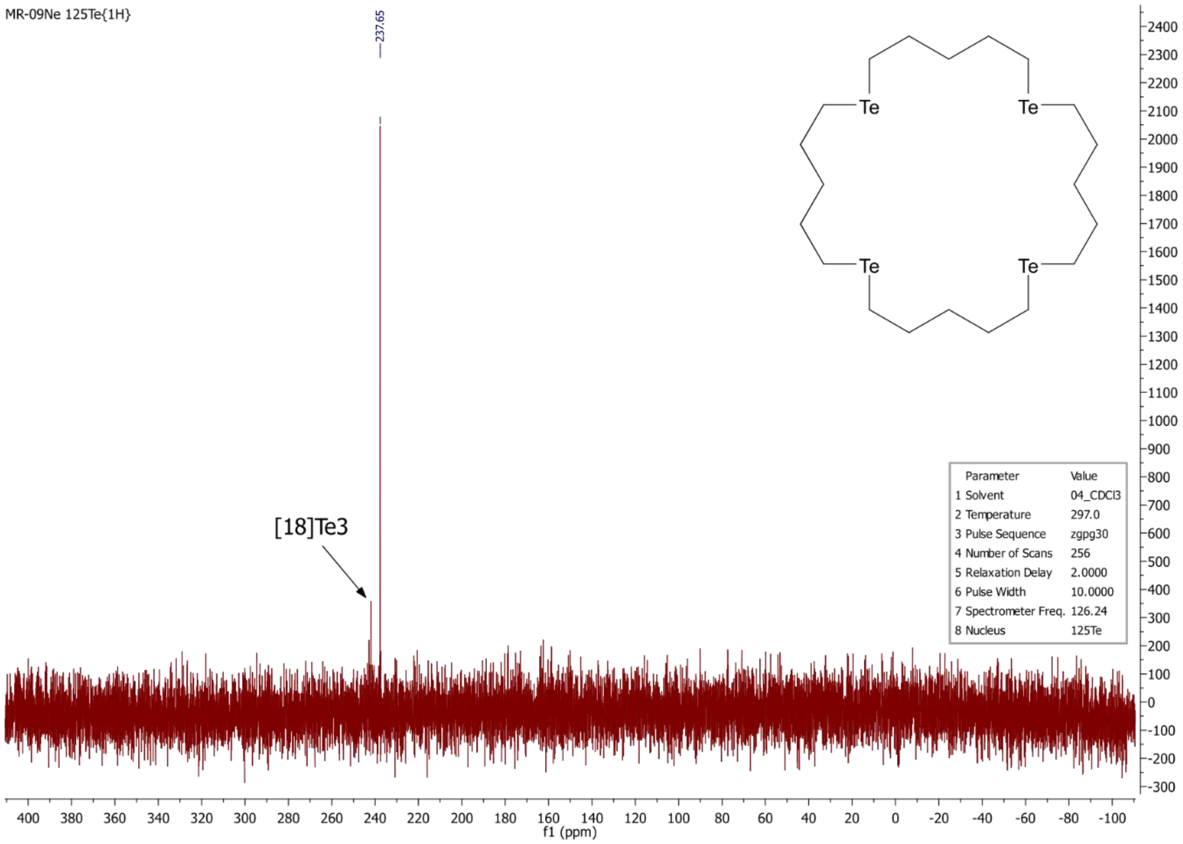

Figure S42. $^{125}Te\{^1H\}$-NMR spectrum of 1,7,13,18-$Te_4(CH_2)_{20}$.

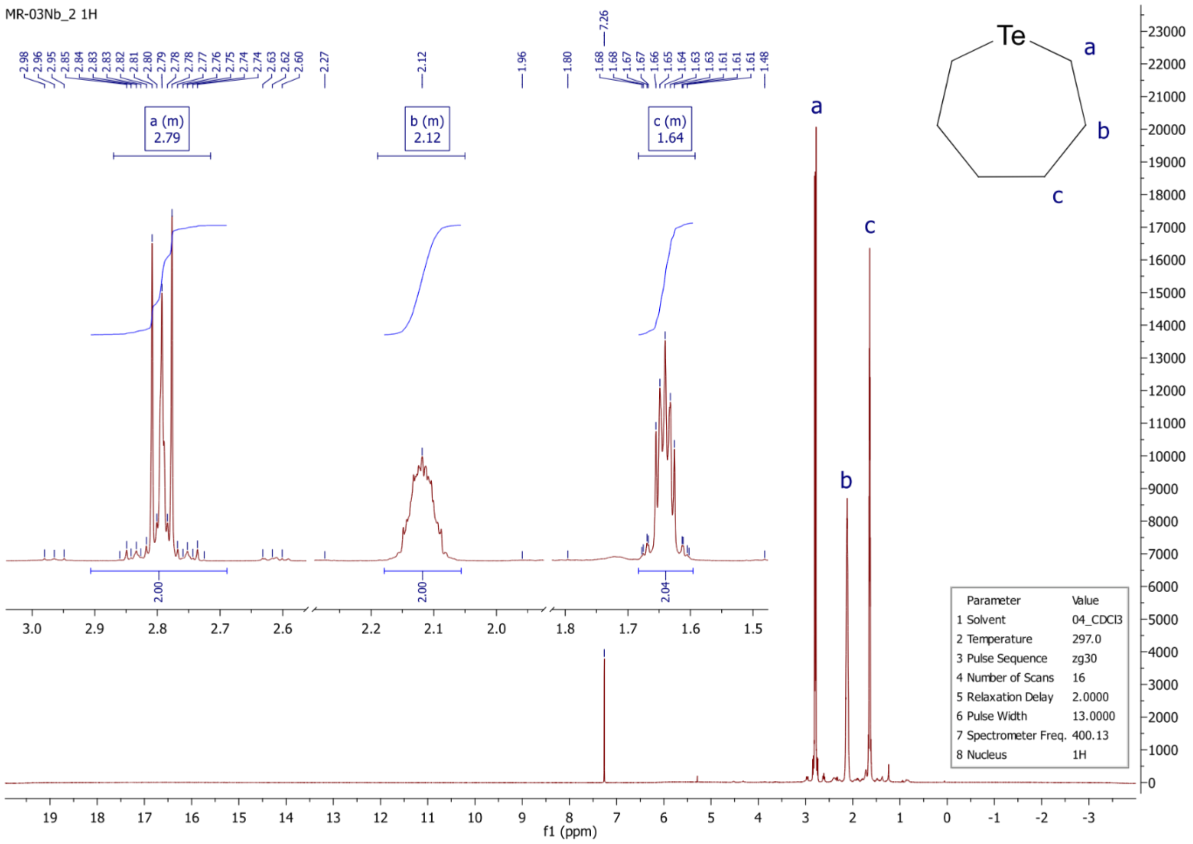

Figure S43. ^{1}H-NMR spectrum of $Te(CH_2)_6$.

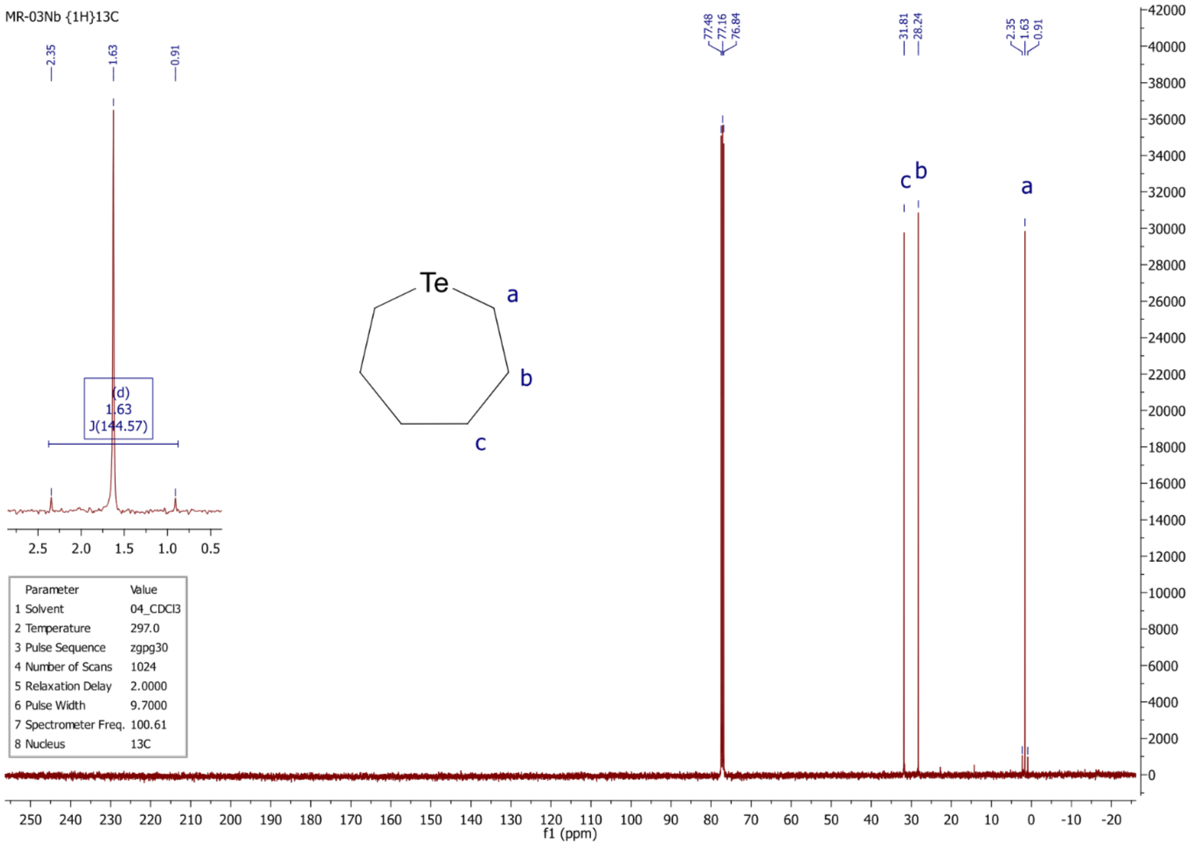

Figure S44. $^{13}C\{^{1}H\}$-NMR spectrum of $Te(CH_2)_6$.

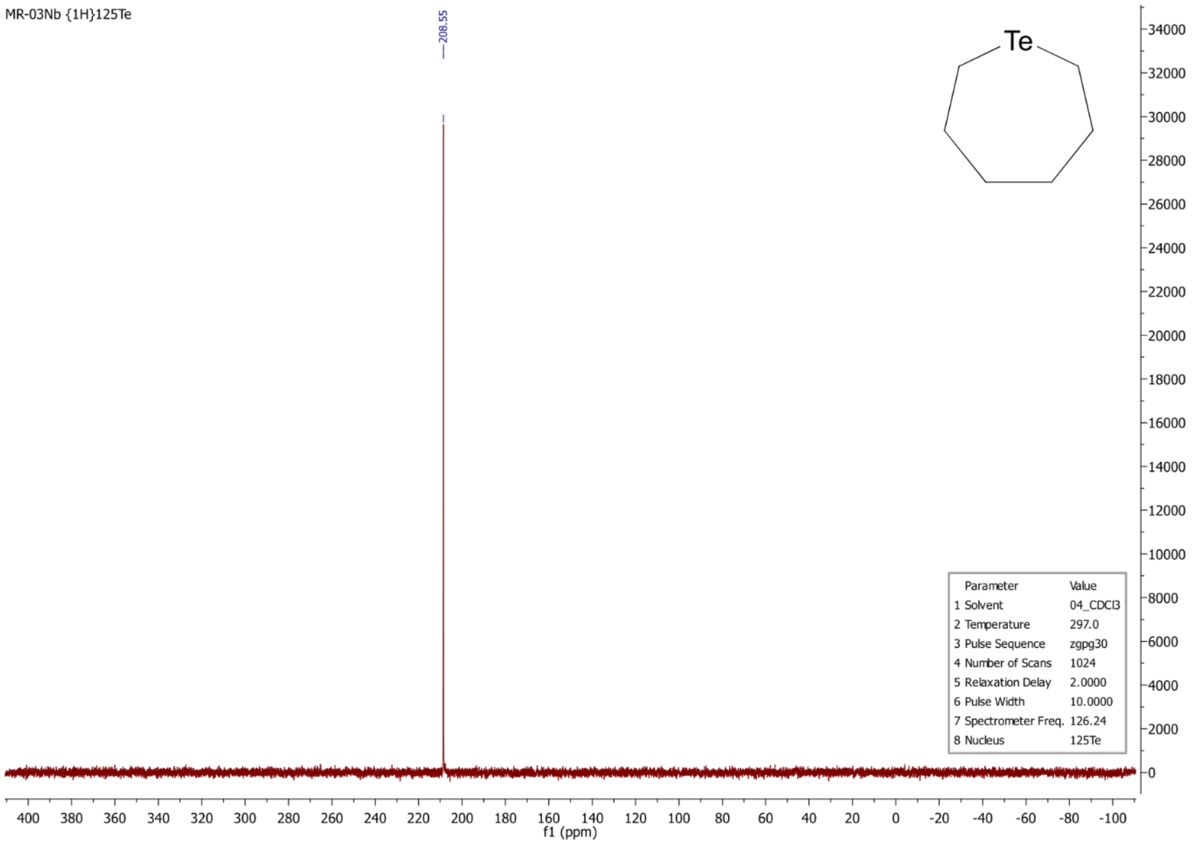

Figure S45. ^{125}Te{^{1}H}-NMR spectrum of $Te(CH_2)_6$.

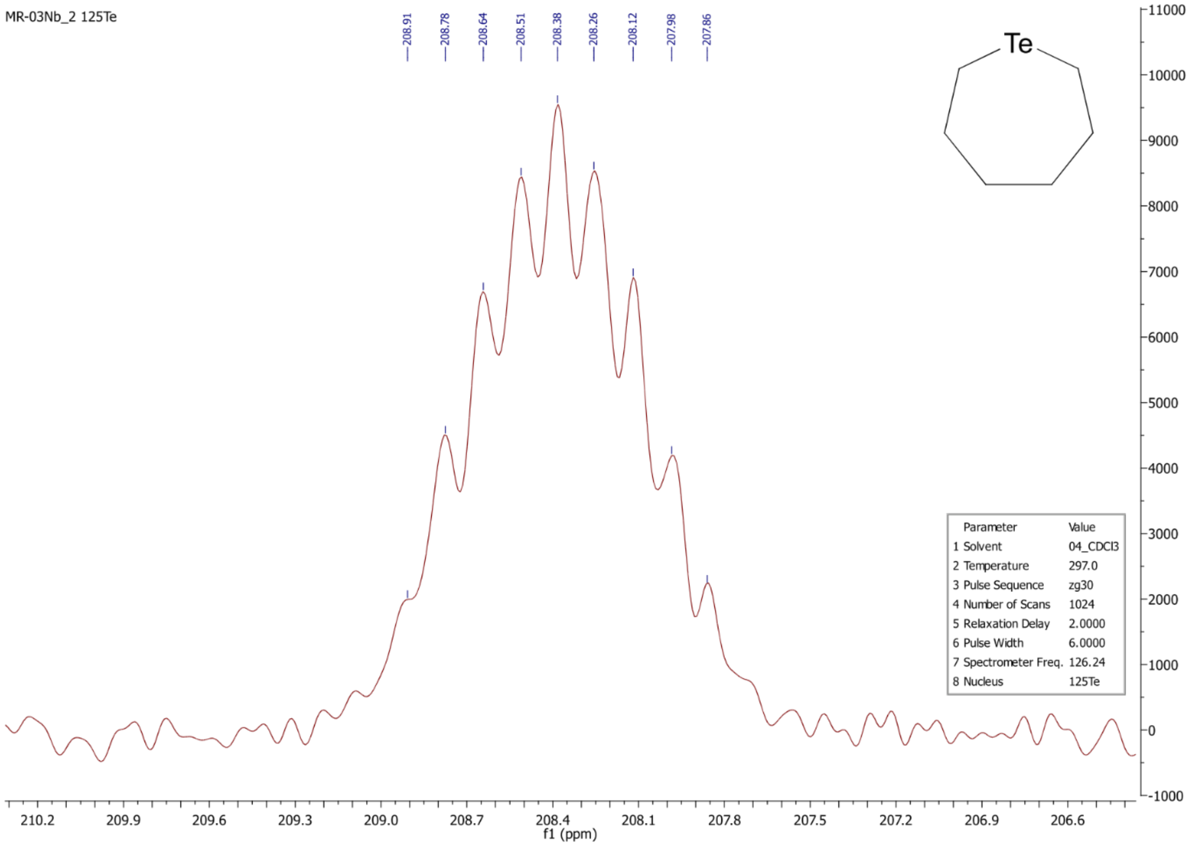

Figure S46. ^{125}Te-NMR spectrum of $Te(CH_2)_6$.

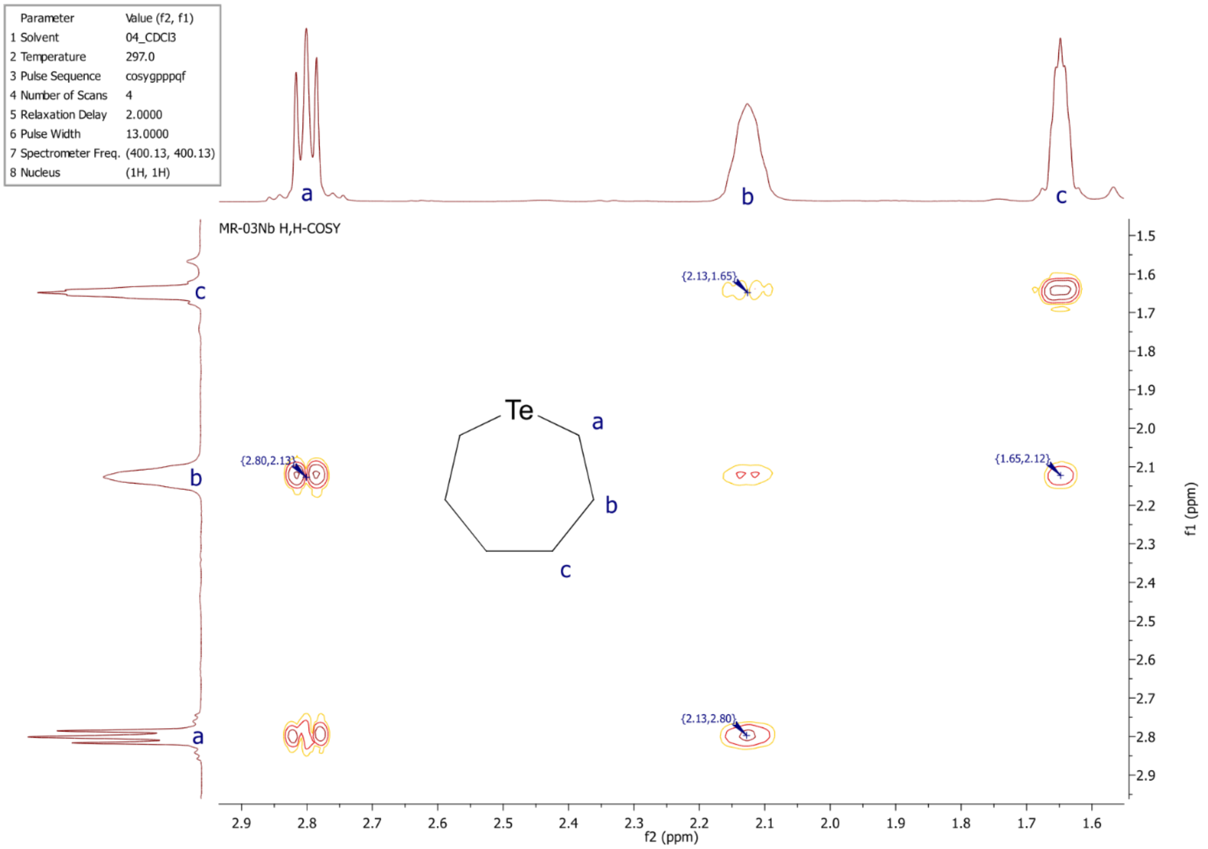

Figure S47. ^{1}H,^{1}H-COSY-NMR spectrum of $Te(CH_2)_6$.

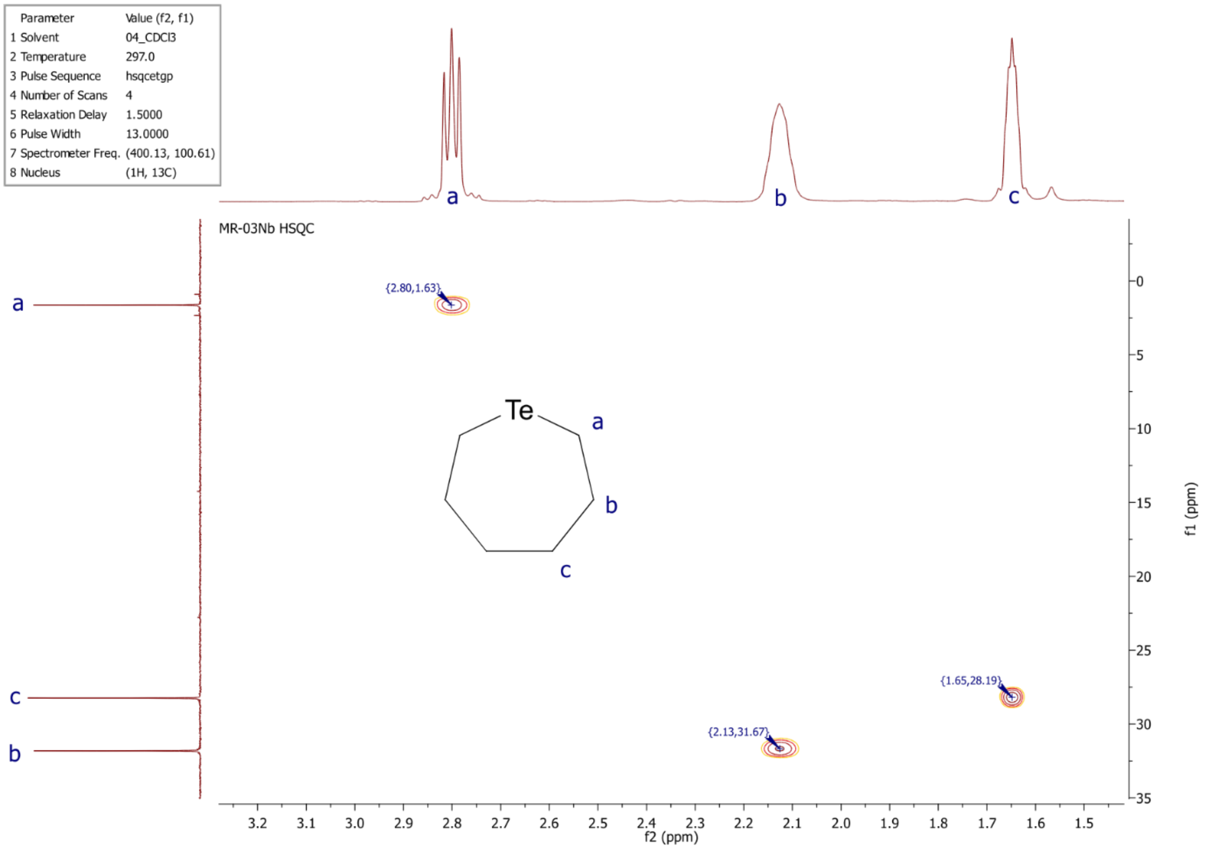

Figure S48. ^{1}H,^{13}C-HSQC-NMR spectrum of $Te(CH_2)_6$.

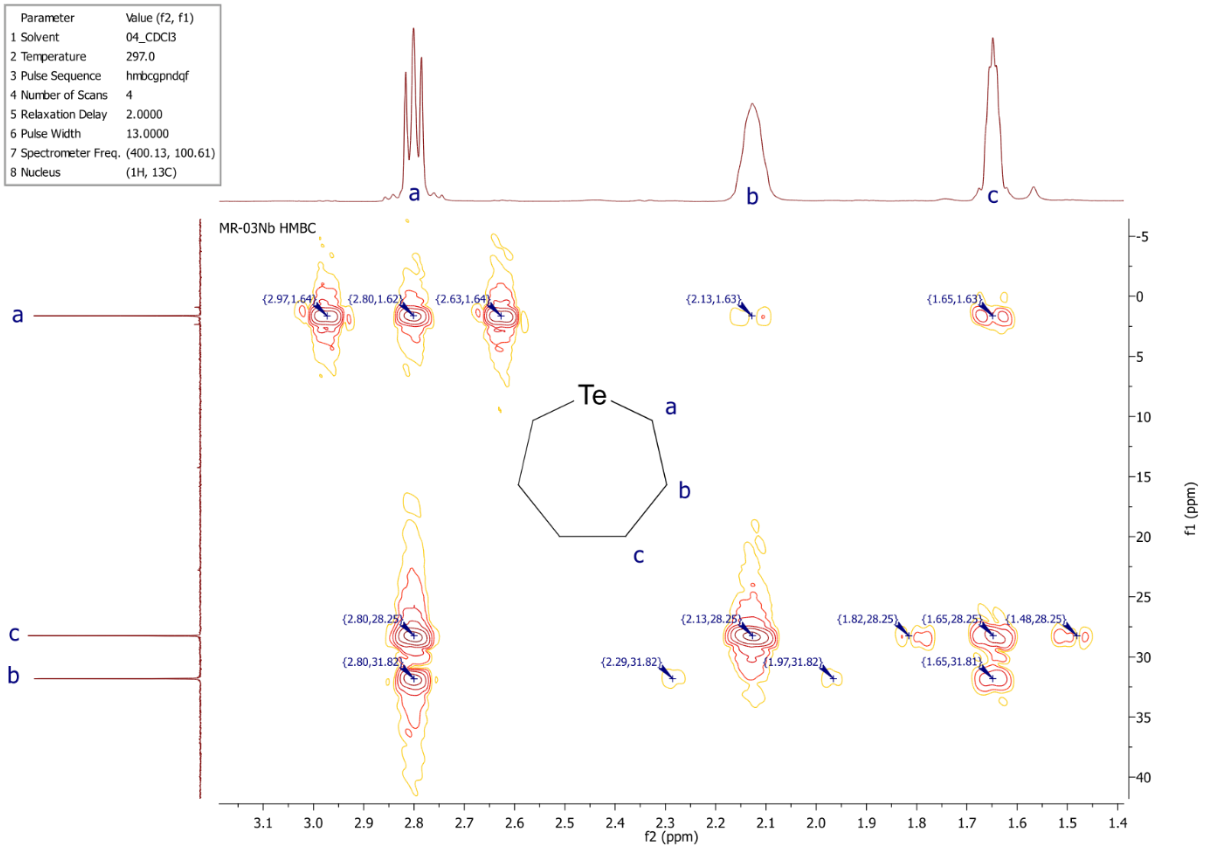

Figure S49. $^{1}H,^{13}C$-HMBC-NMR spectrum of $Te(CH_2)_6$.

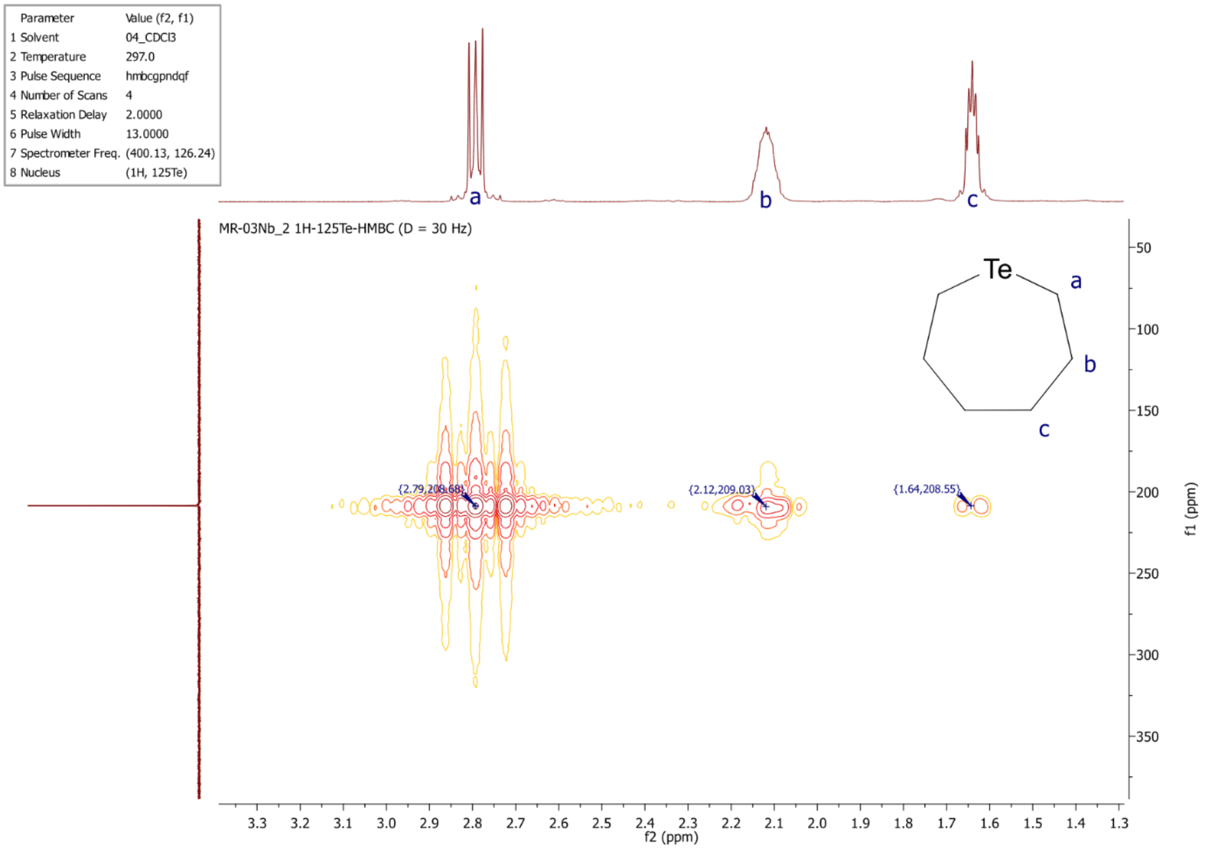

Figure S50. $^{1}H,^{125}Te$-HMBC-NMR spectrum of $Te(CH_2)_6$.

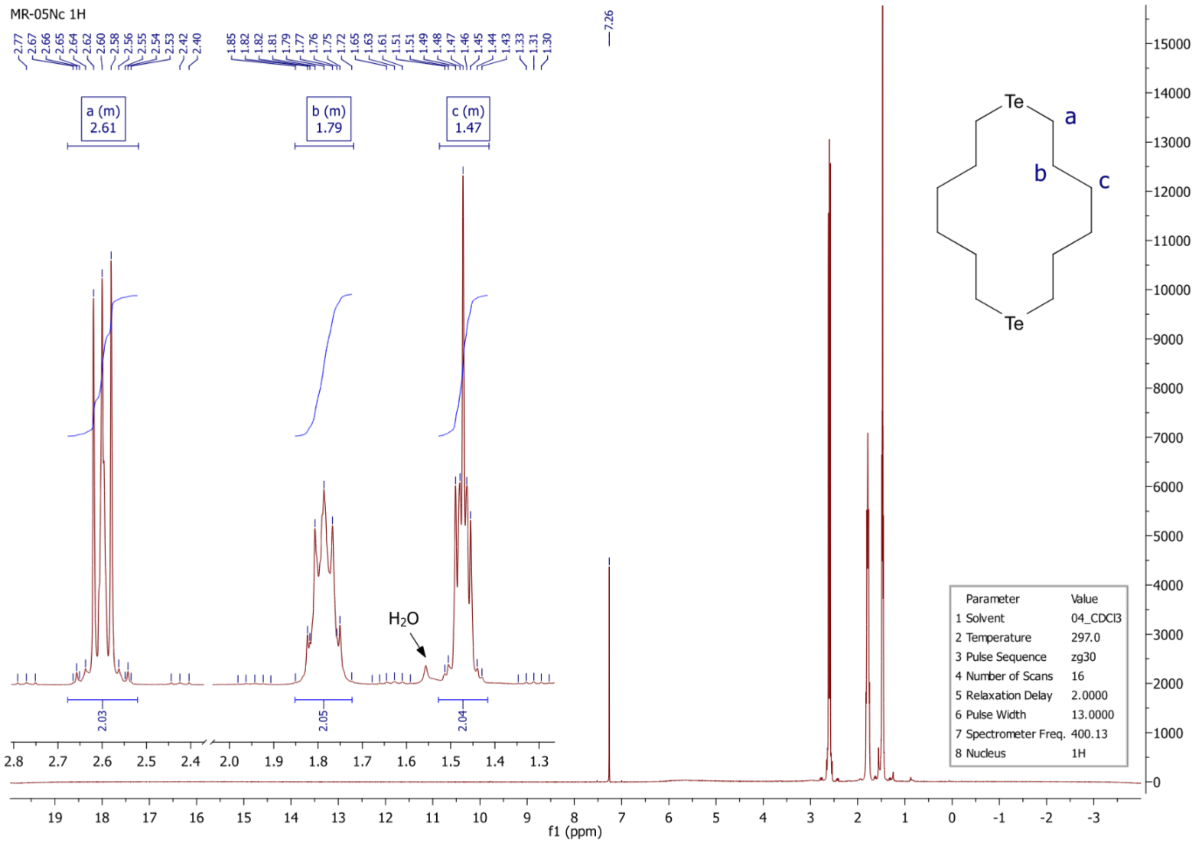

Figure S51. ^{1}H-NMR spectrum of 1,8-$Te_2(CH_2)_{12}$.

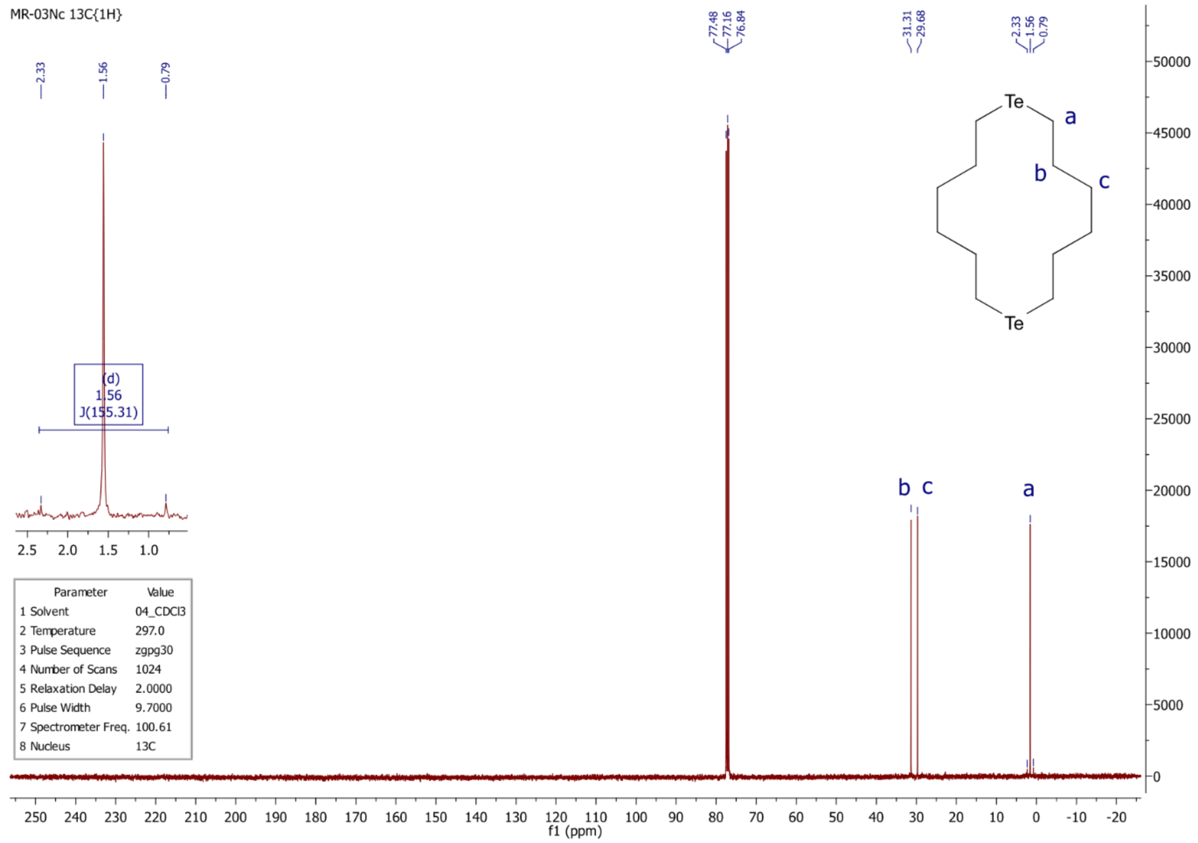

Figure S52. $^{13}C\{^{1}H\}$-NMR spectrum of 1,8-$Te_2(CH_2)_{12}$.

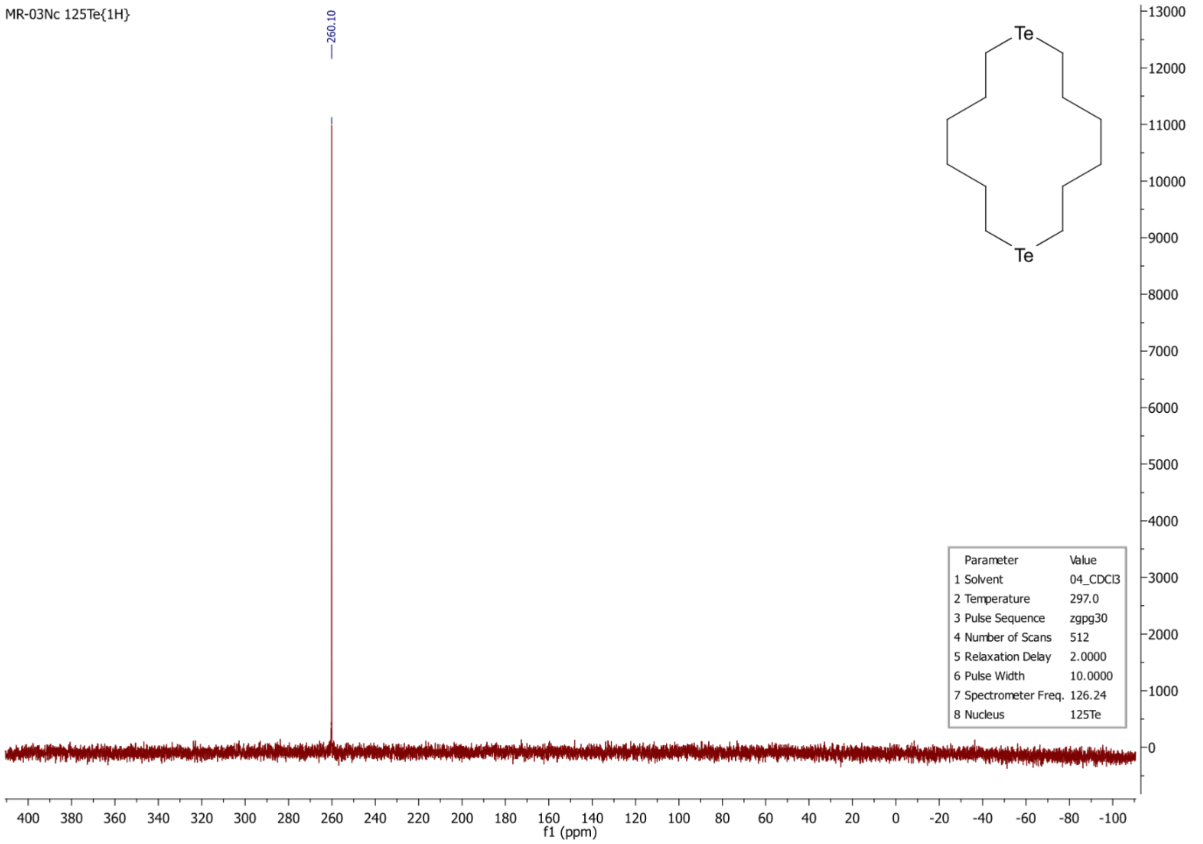

Figure S53. $^{125}Te\{^{1}H\}$-NMR spectrum of 1,8-$Te_2(CH_2)_{12}$.

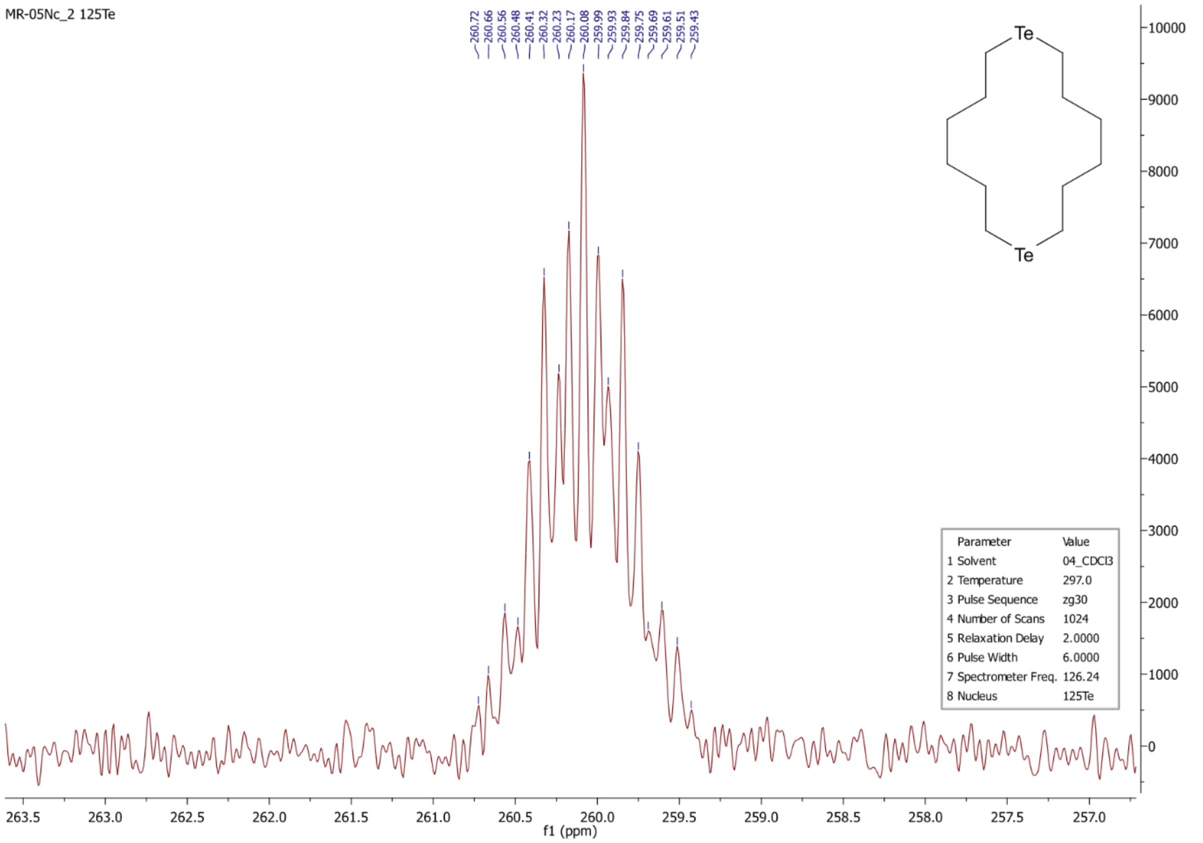

Figure S54. ^{125}Te-NMR spectrum of 1,8-$Te_2(CH_2)_{12}$.

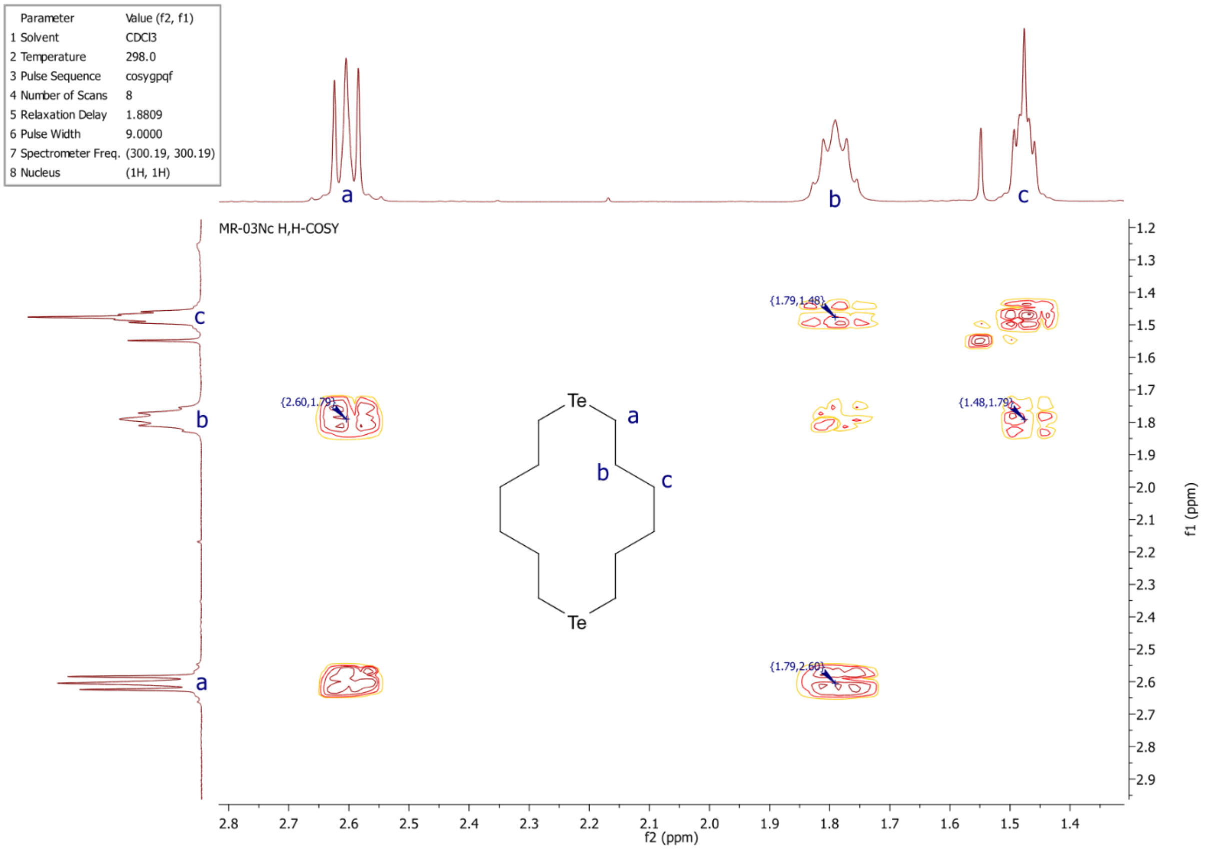

Figure S55. ^{1}H,^{1}H-COSY-NMR spectrum of 1,8-$Te_2(CH_2)_{12}$.

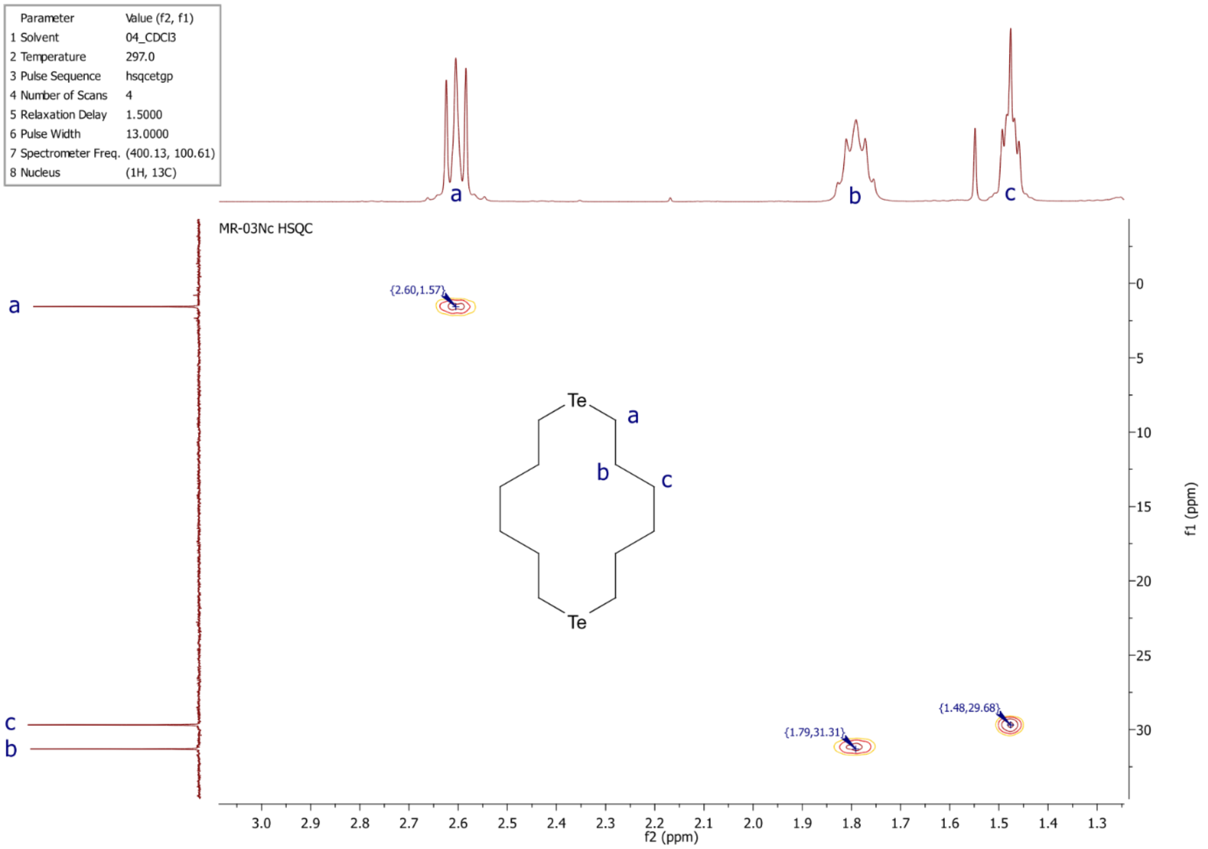

Figure S56. ^{1}H,^{13}C-HSQC-NMR spectrum of 1,8-$Te_2(CH_2)_{12}$.

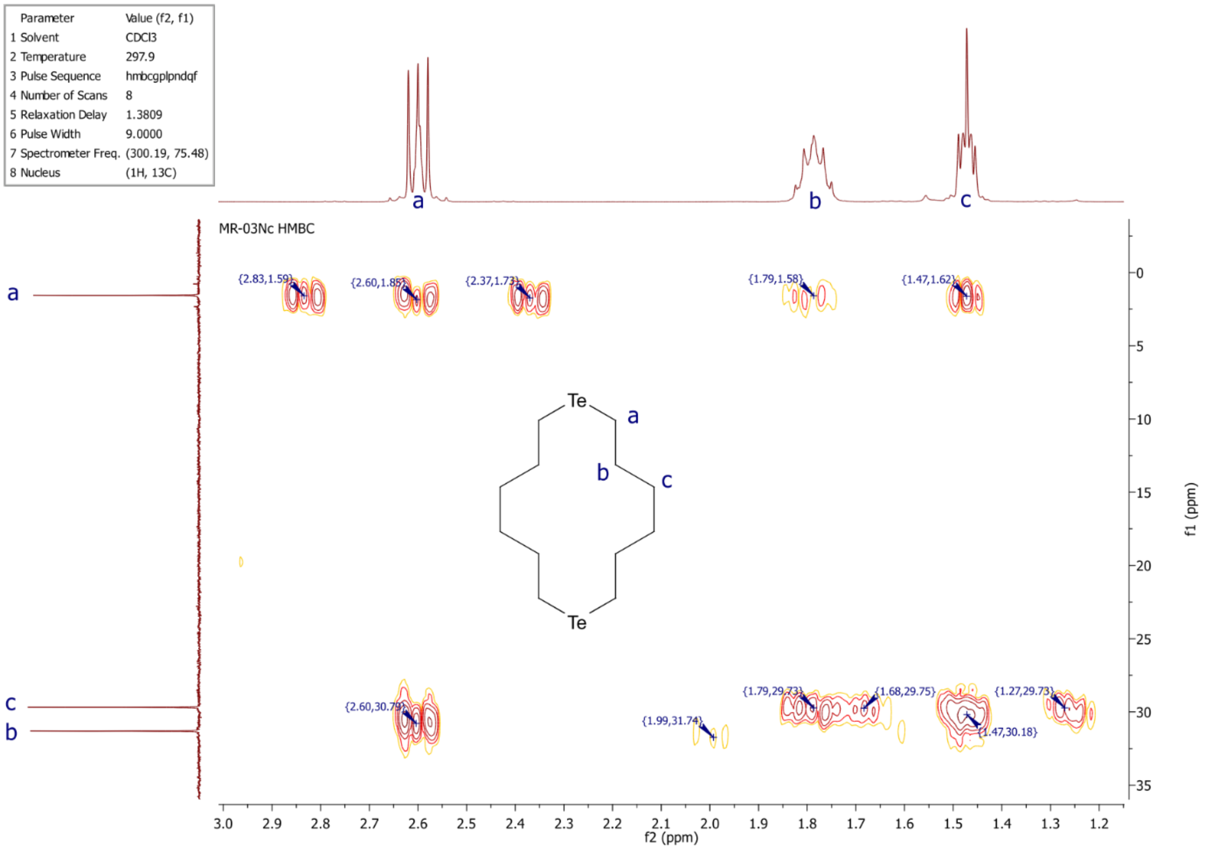

Figure S57. ^{1}H,^{13}C-HMBC-NMR spectrum of 1,8-$Te_2(CH_2)_{12}$.

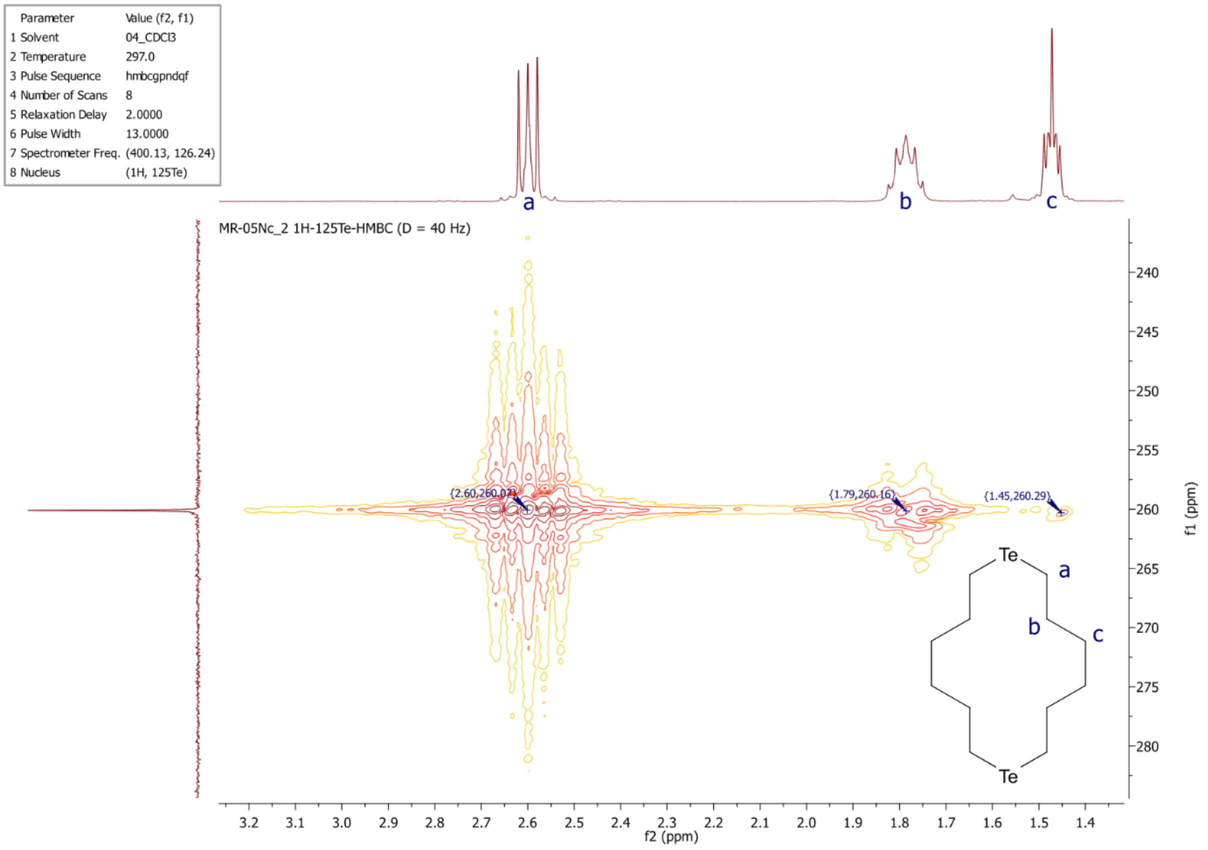

Figure S58. ^{1}H,^{125}Te-HMBC-NMR spectrum of 1,8-$Te_2(CH_2)_{12}$.

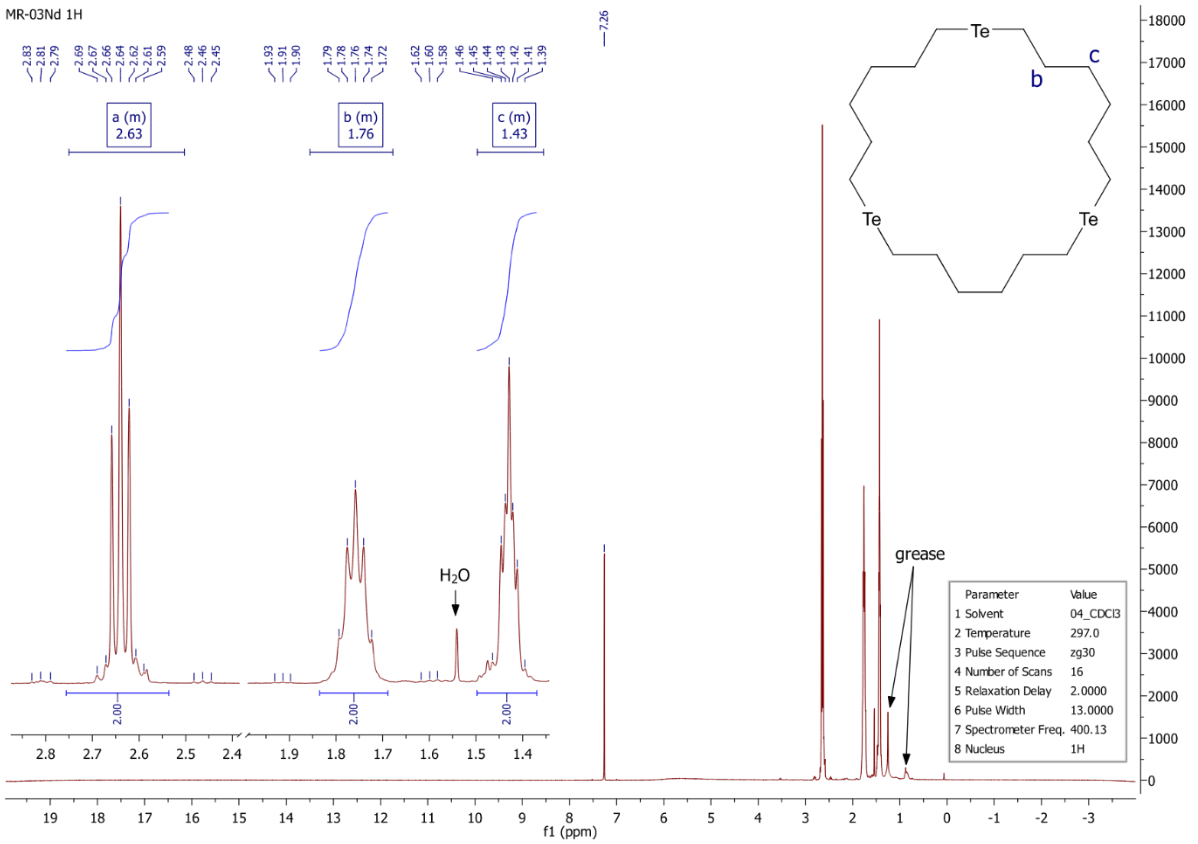

Figure S59. ^{1}H-NMR spectrum of 1,8,15-$Te_3(CH_2)_{18}$.

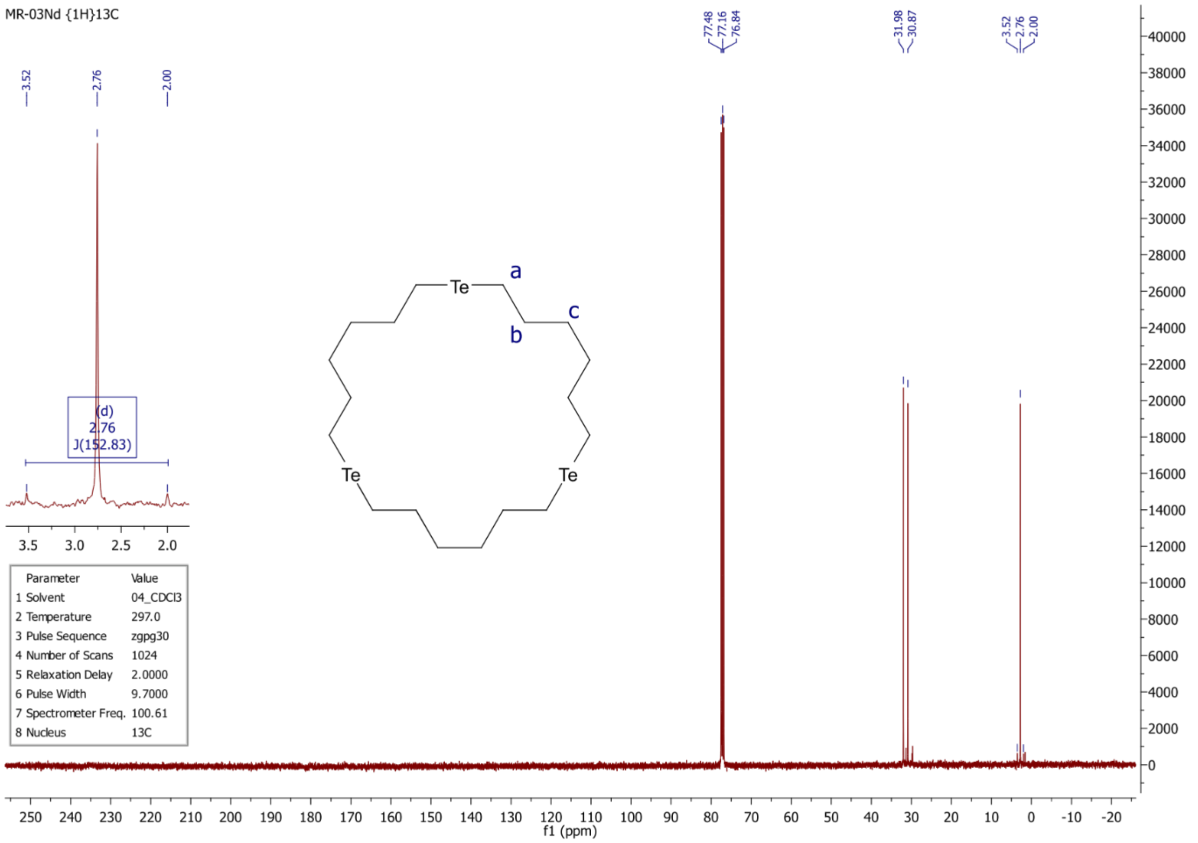

Figure S60. $^{13}C\{^{1}H\}$-NMR spectrum of 1,8,15-$Te_3(CH_2)_{18}$.

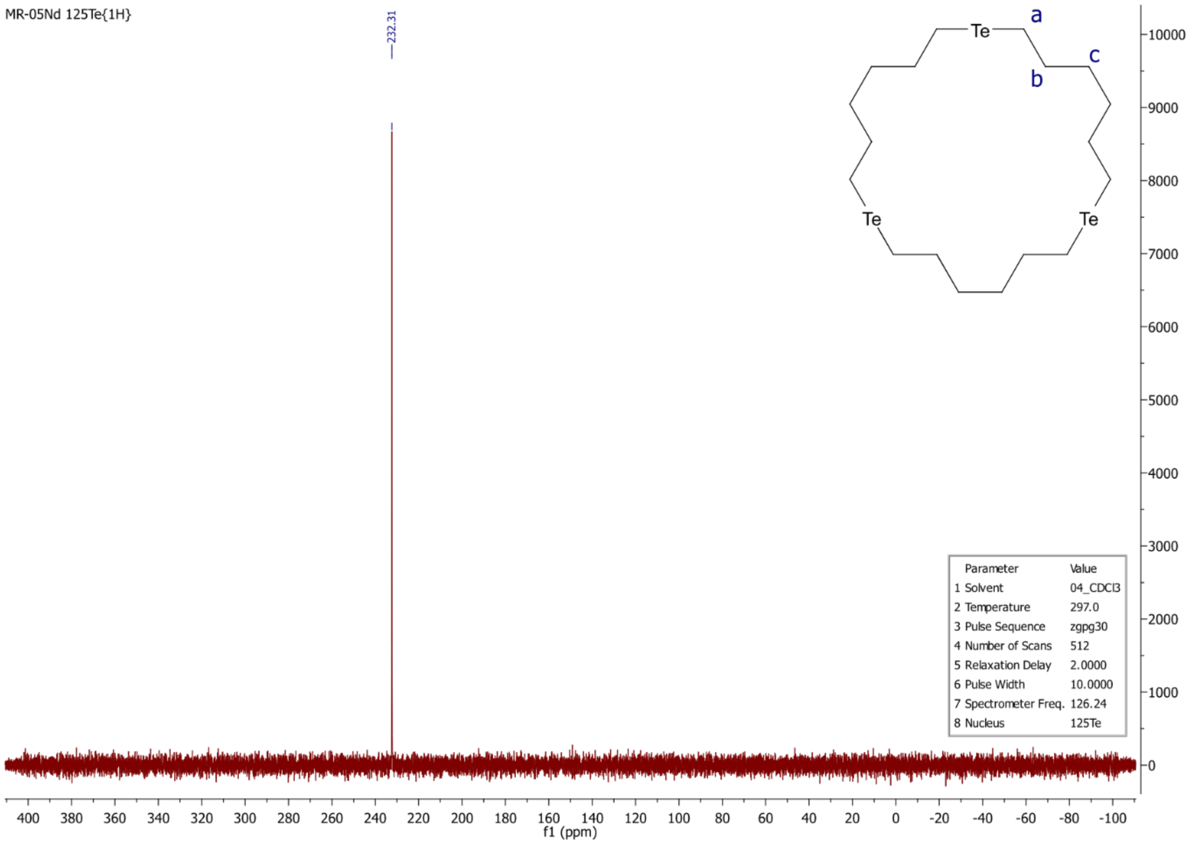

Figure S61. ^{125}Te{^{1}H}-NMR spectrum of 1,8,15-$Te_3(CH_2)_{18}$.

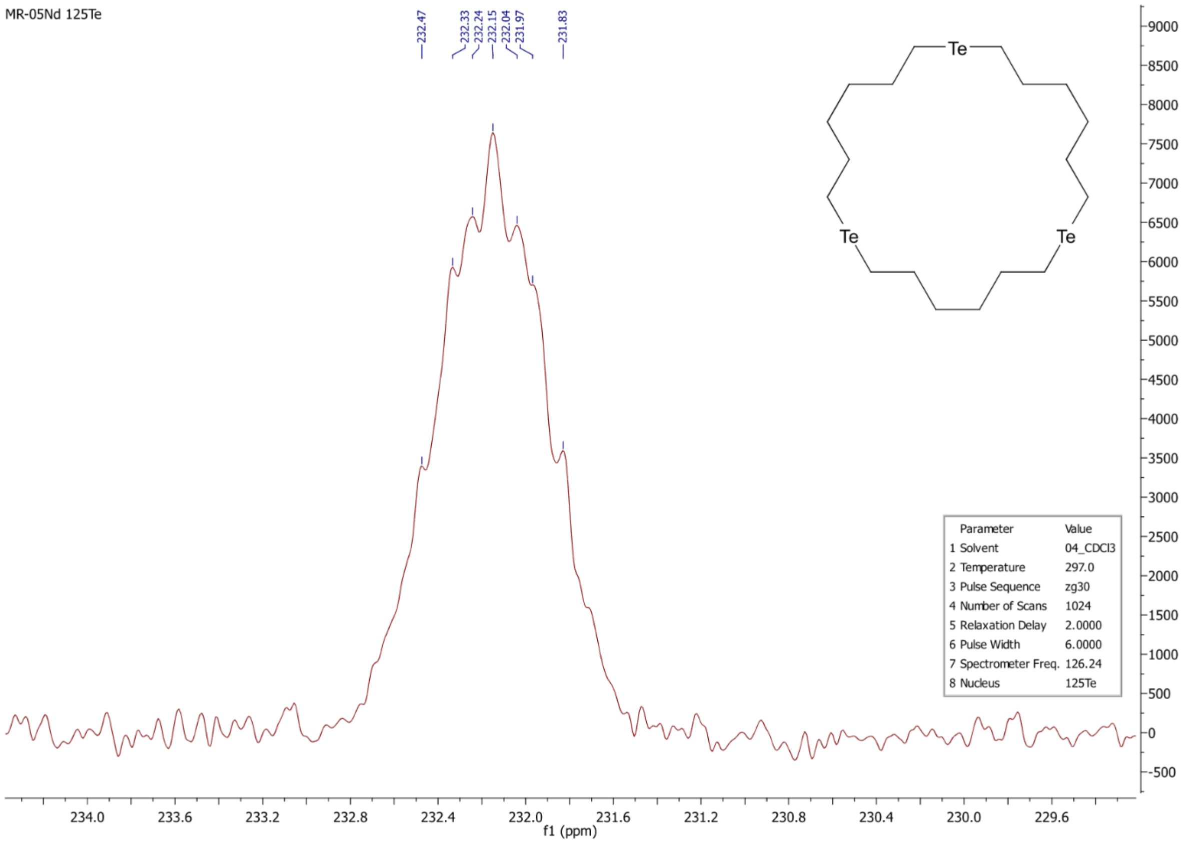

Figure S62. ^{125}Te-NMR spectrum of 1,8,15-$Te_3(CH_2)_{18}$.

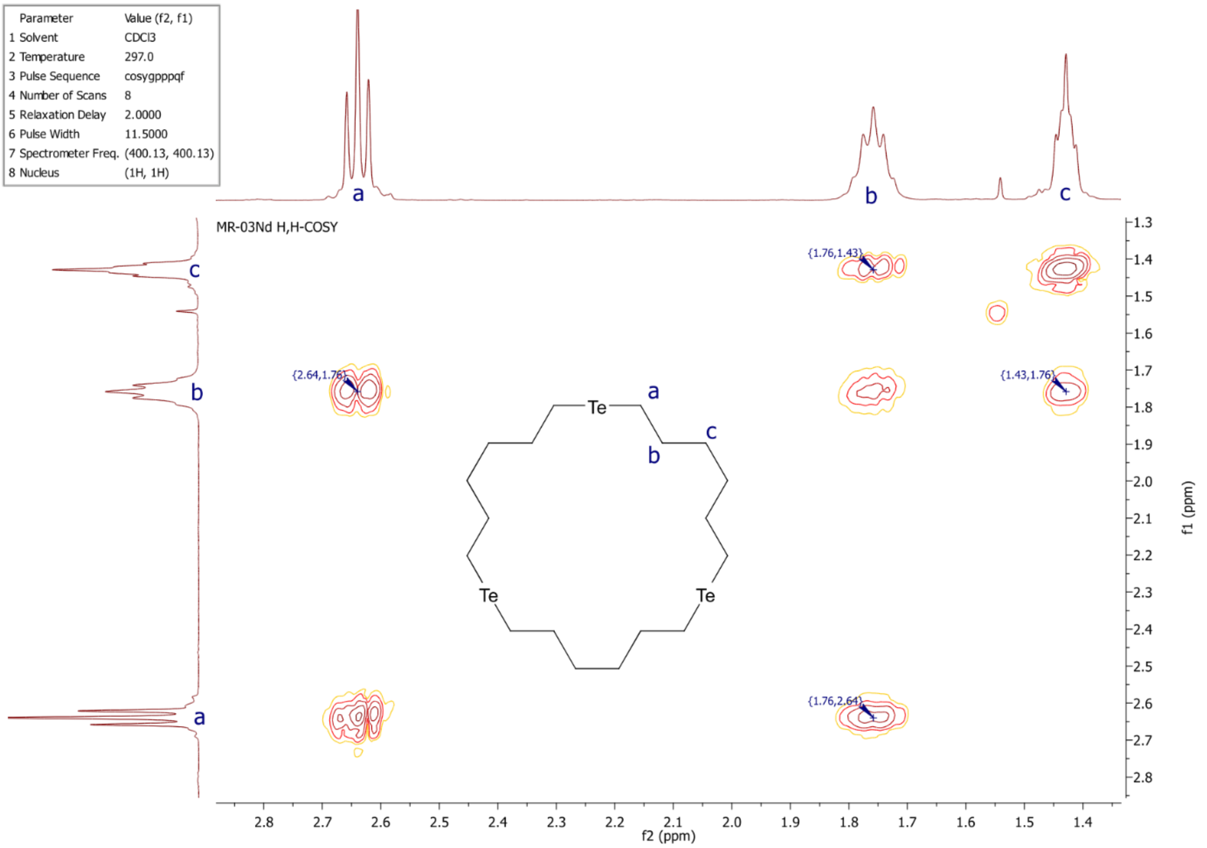

Figure S63. $^{1}H,^{1}H$-COSY-NMR spectrum of 1,8,15-$Te_3(CH_2)_{18}$.

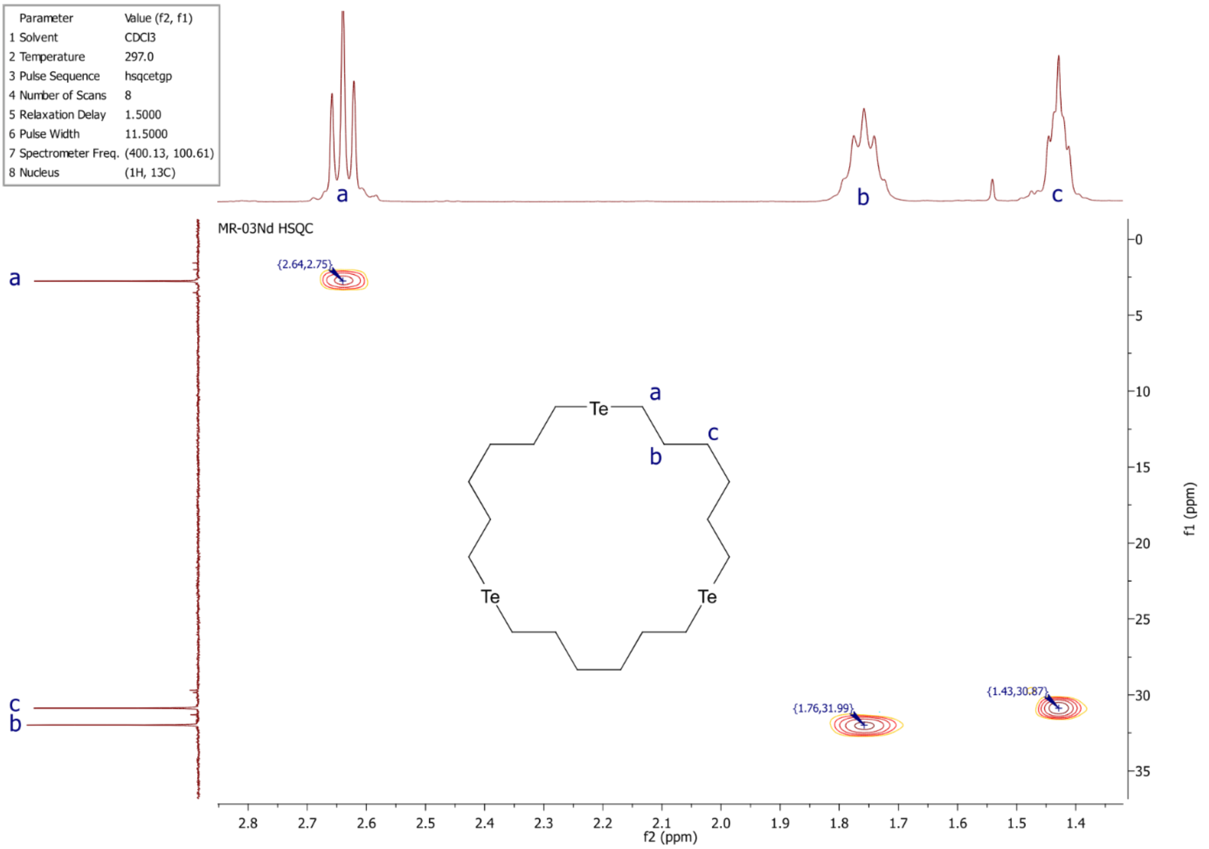

Figure S64. $^{1}H,^{13}C$-HSQC-NMR spectrum of 1,8,15-$Te_3(CH_2)_{18}$.

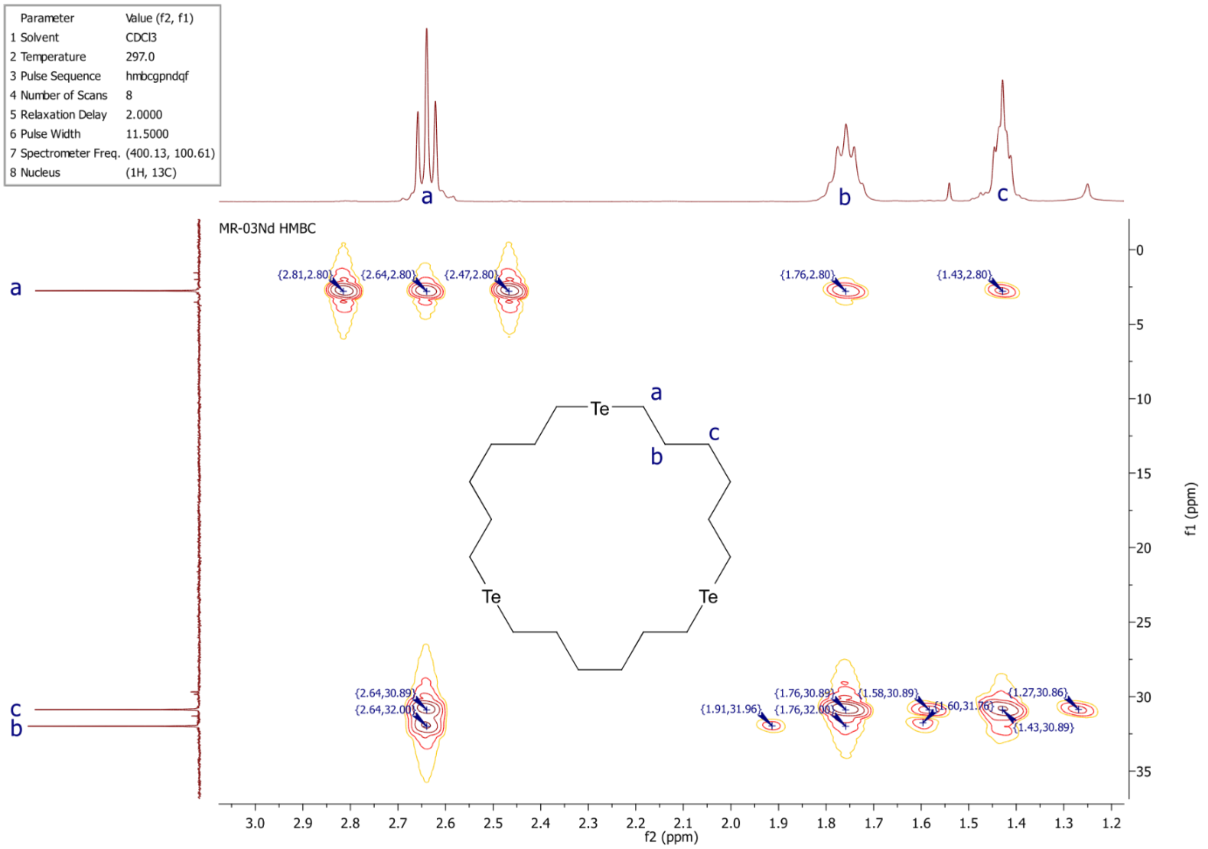

Figure S65. $^{1}H,^{13}C$-HMBC-NMR spectrum of 1,8,15-$Te_3(CH_2)_{18}$.

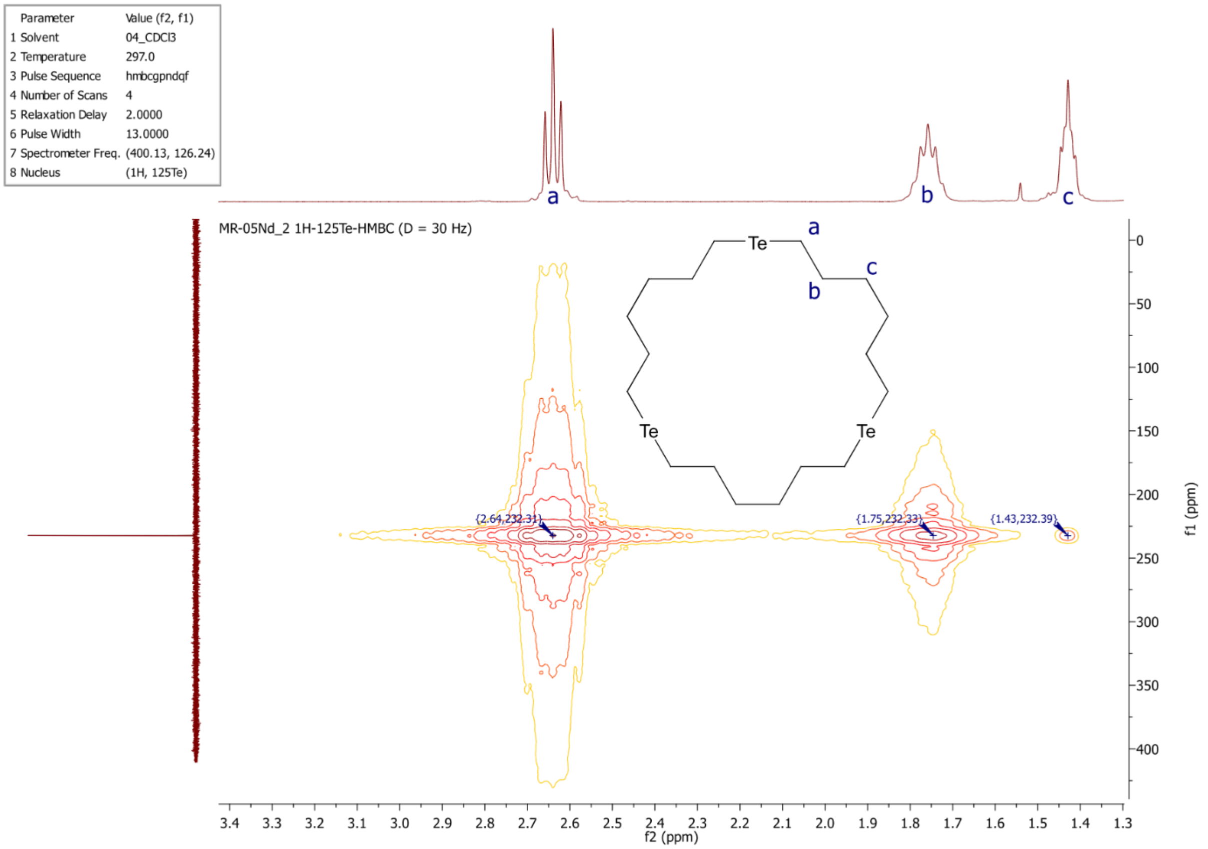

Figure S66. $^{1}H,^{125}Te$-HMBC-NMR spectrum of 1,8,15-$Te_3(CH_2)_{18}$.

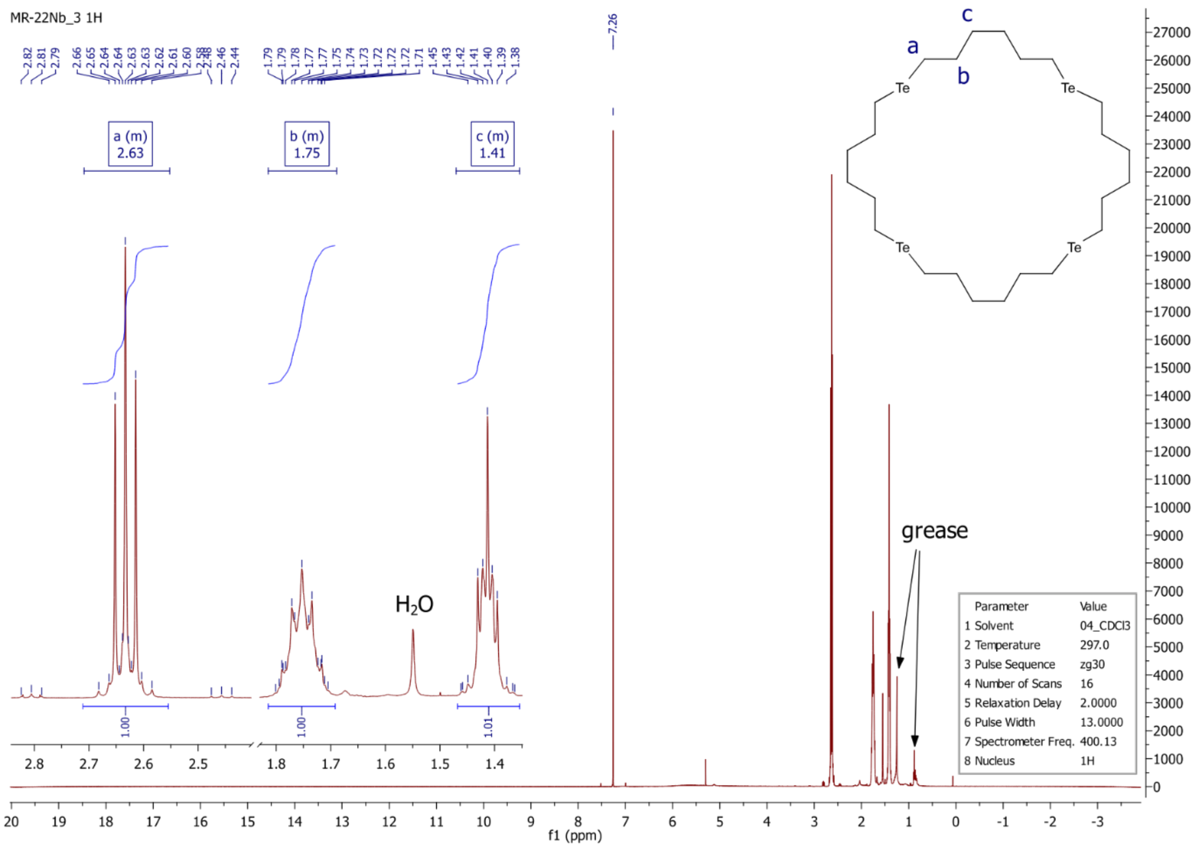

Figure S67. ^{1}H-NMR spectrum of 1,8,15,22-$Te_4(CH_2)_{24}$.

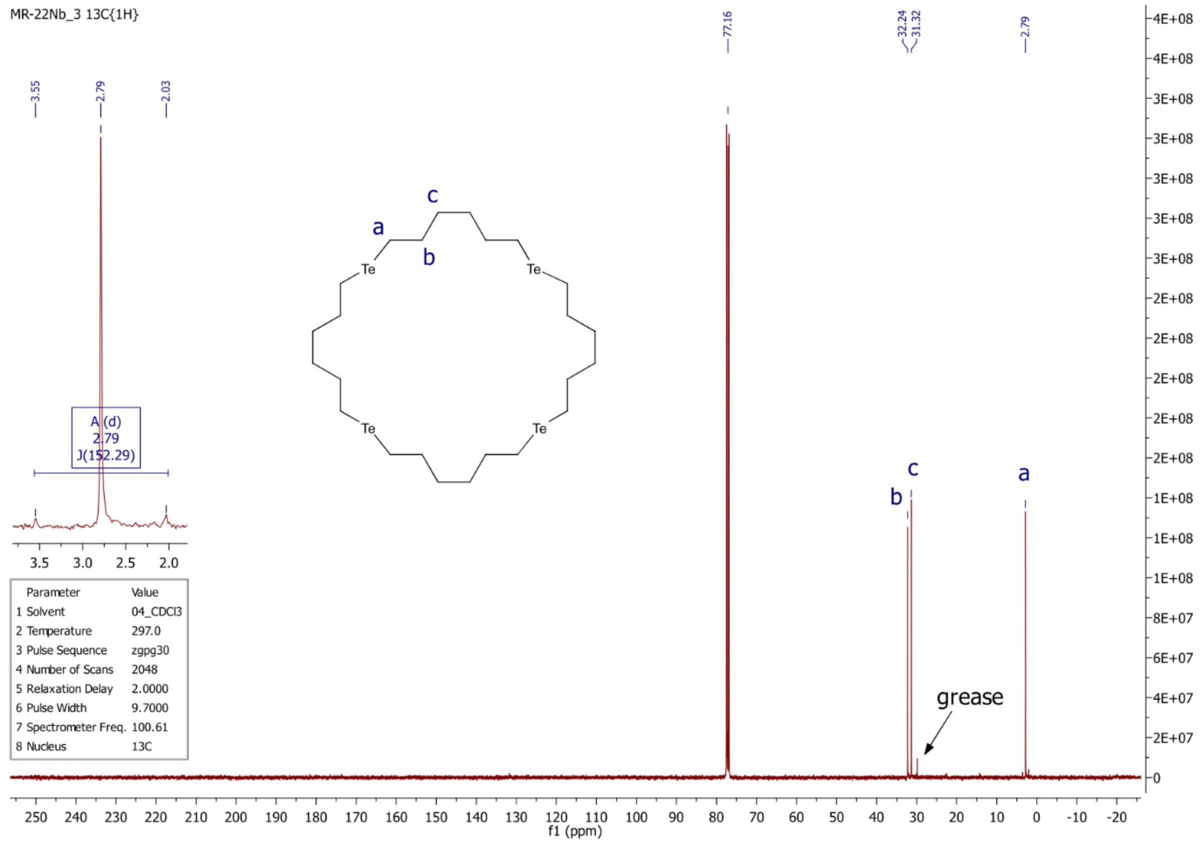

Figure S68. $^{13}C\{^{1}H\}$-NMR spectrum of 1,8,15,22-$Te_4(CH_2)_{24}$.

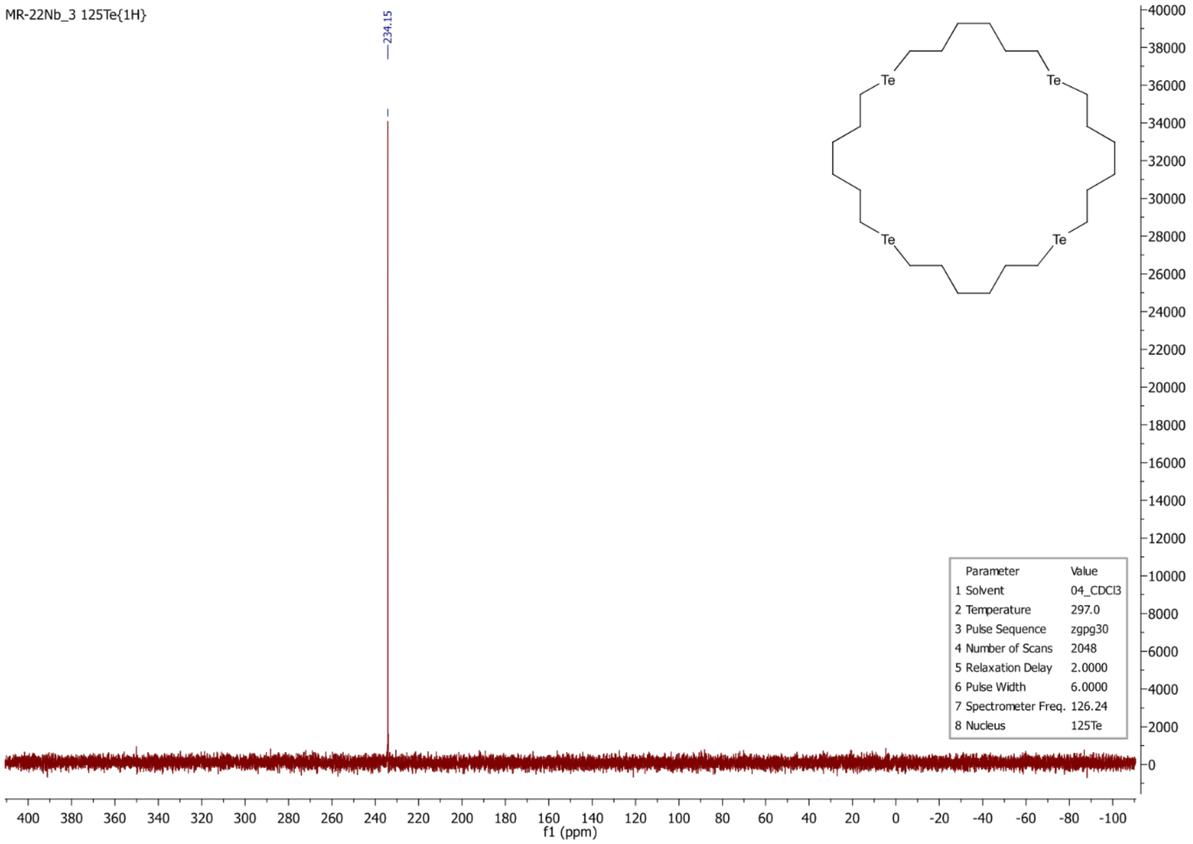

Figure S69. $^{125}Te\{^{1}H\}$-NMR spectrum of 1,8,15,22-$Te_4(CH_2)_{24}$.

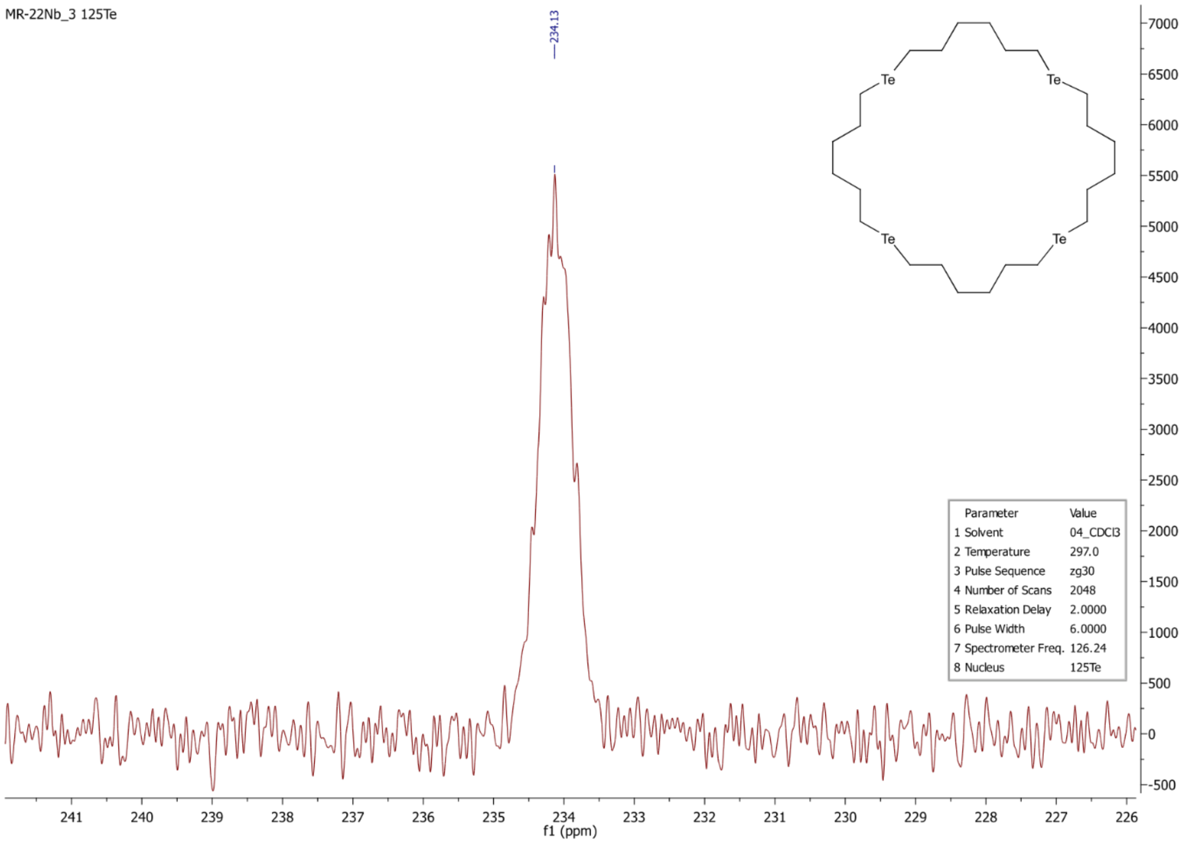

Figure S70. ^{125}Te-NMR spectrum of 1,8,15,22-$Te_4(CH_2)_{24}$.

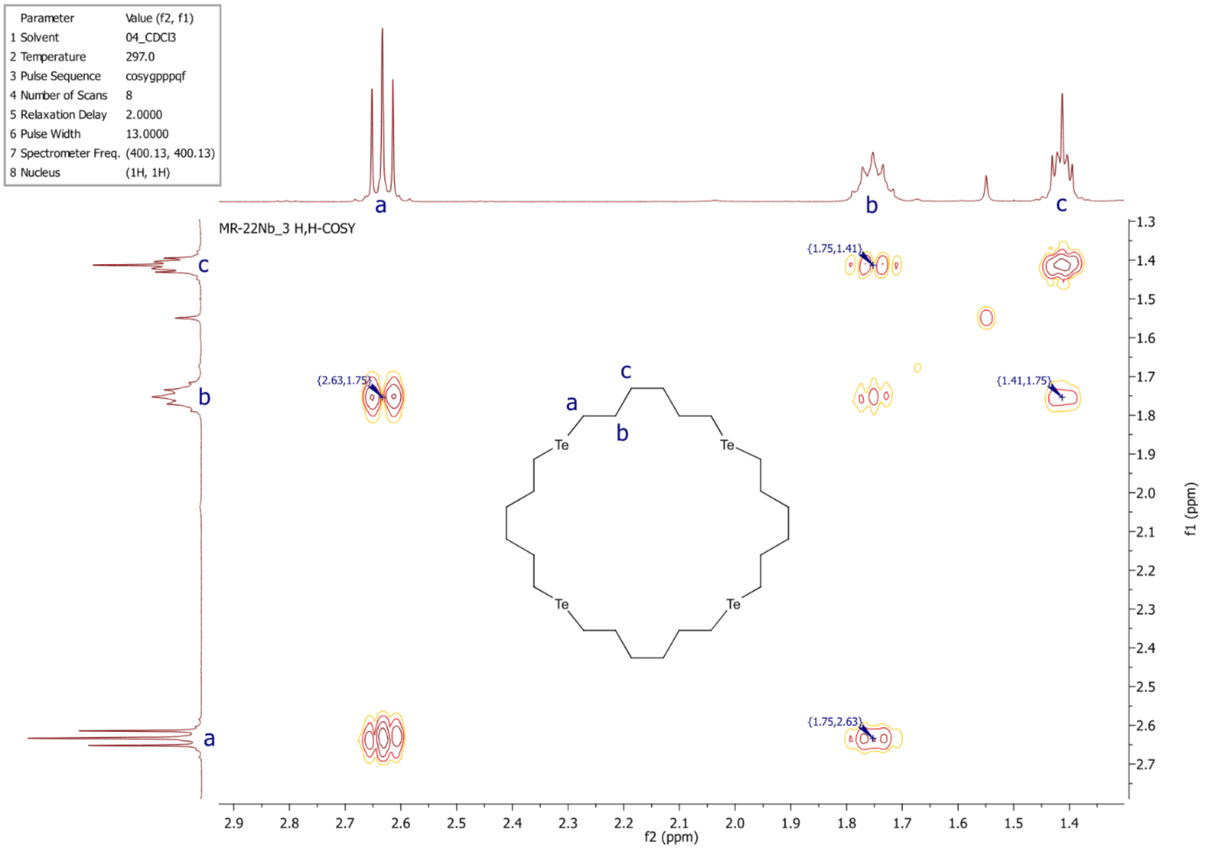

Figure S71. $^{1}H,^{1}H$-COSY-NMR spectrum of 1,8,15,22-$Te_4(CH_2)_{24}$.

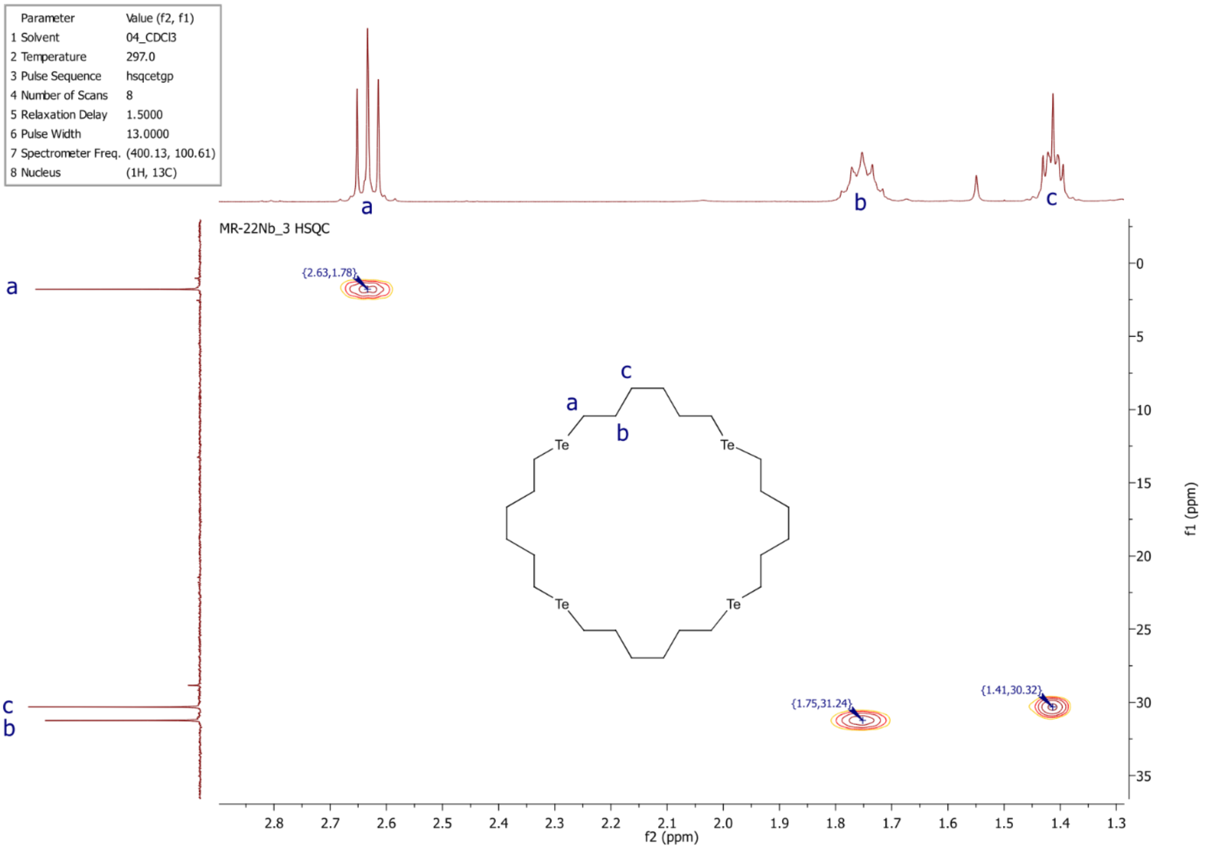

Figure S72. $^{1}H,^{13}C$-HSQC-NMR spectrum of 1,8,15,22-$Te_4(CH_2)_{24}$.

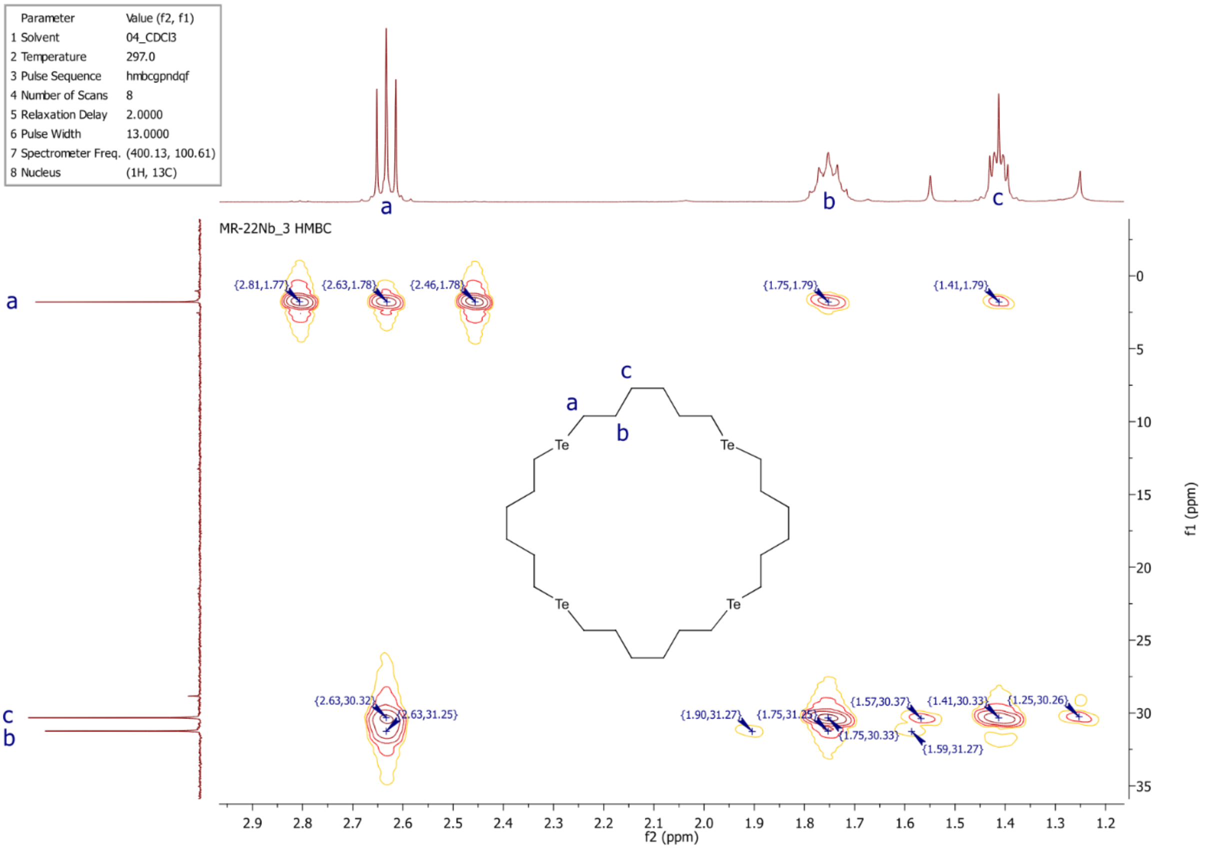

Figure S73. $^{1}H,^{13}C$-HMBC-NMR spectrum of 1,8,15,22-$Te_4(CH_2)_{24}$.

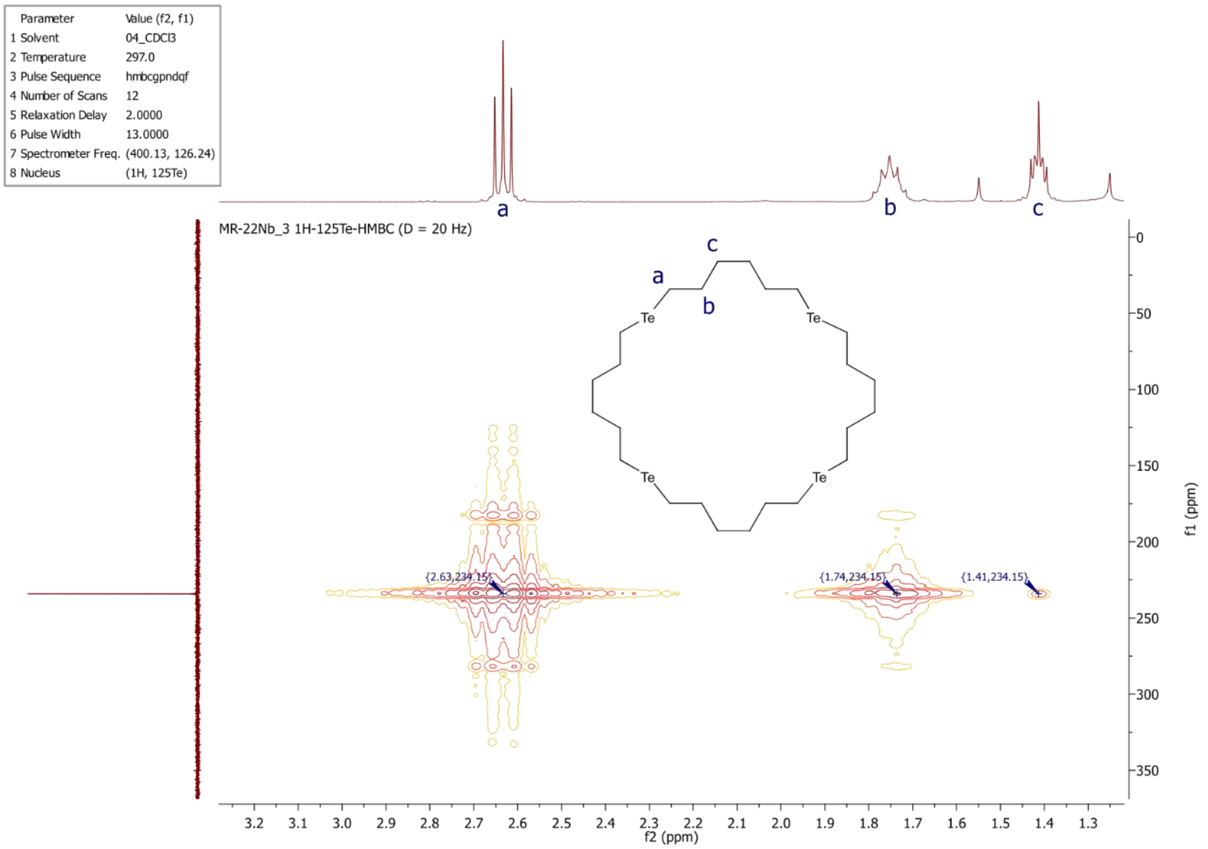

Figure S74. $^{1}H,^{125}Te$-HMBC-NMR spectrum of 1,8,15,22-$Te_4(CH_2)_{24}$.

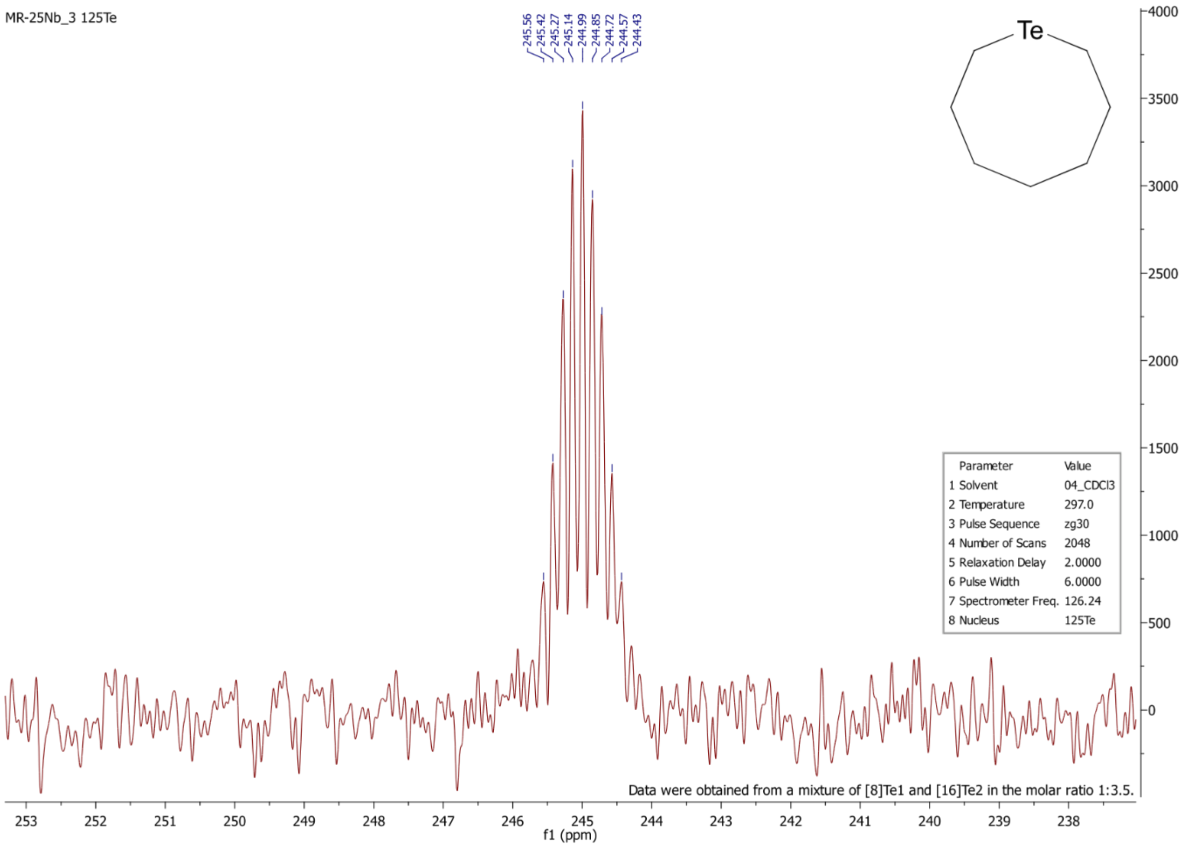

Figure S75. ^{125}Te-NMR spectrum of Te(CH_2)$_7$.

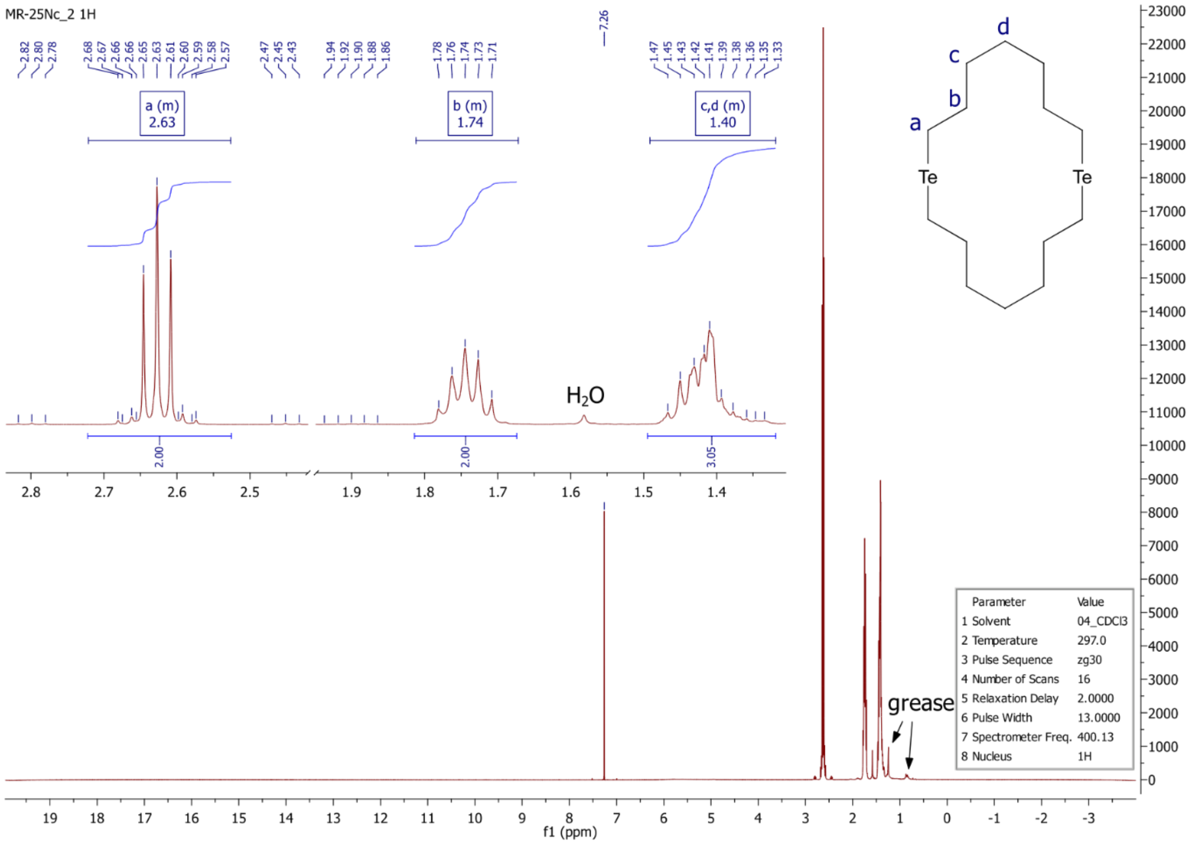

Figure S76. ^{1}H-NMR spectrum of 1,9-$Te_2(CH_2)_{14}$.

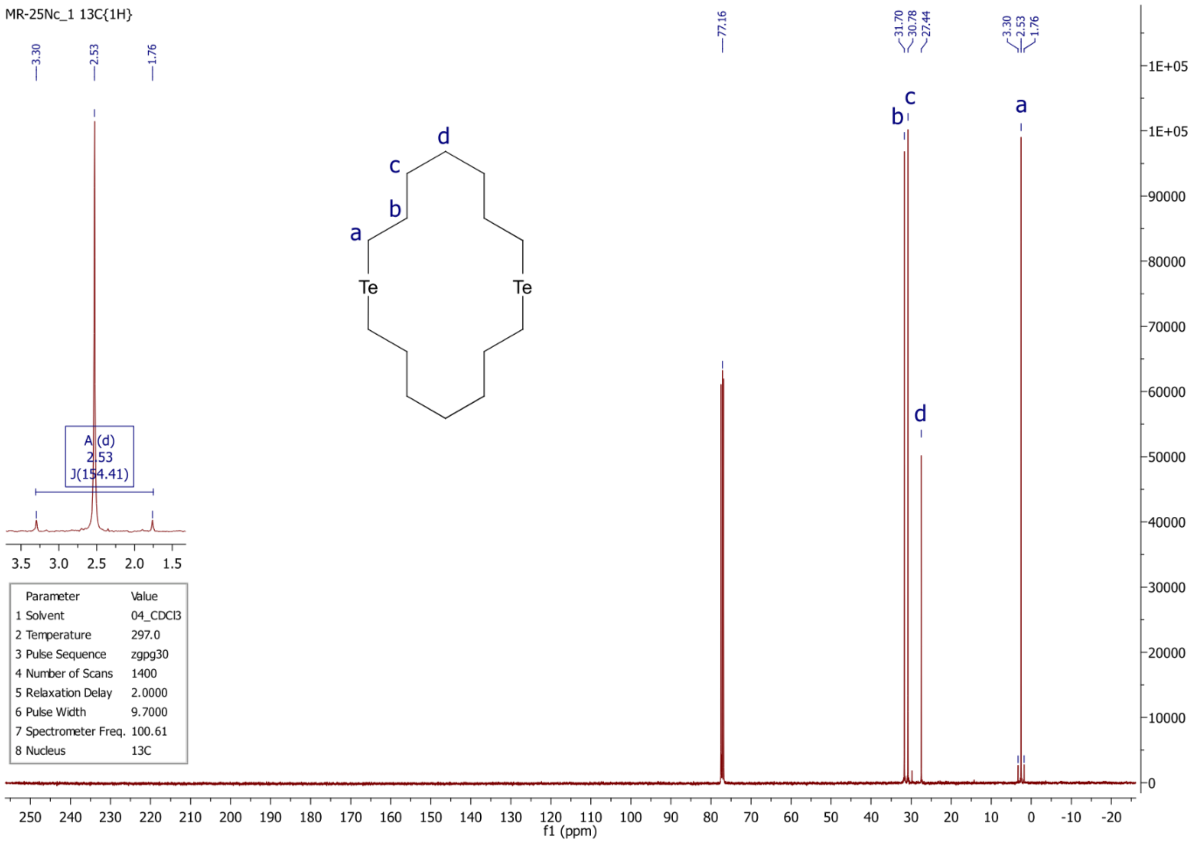

Figure S77. $^{13}C\{^{1}H\}$-NMR spectrum of 1,9-$Te_2(CH_2)_{14}$.

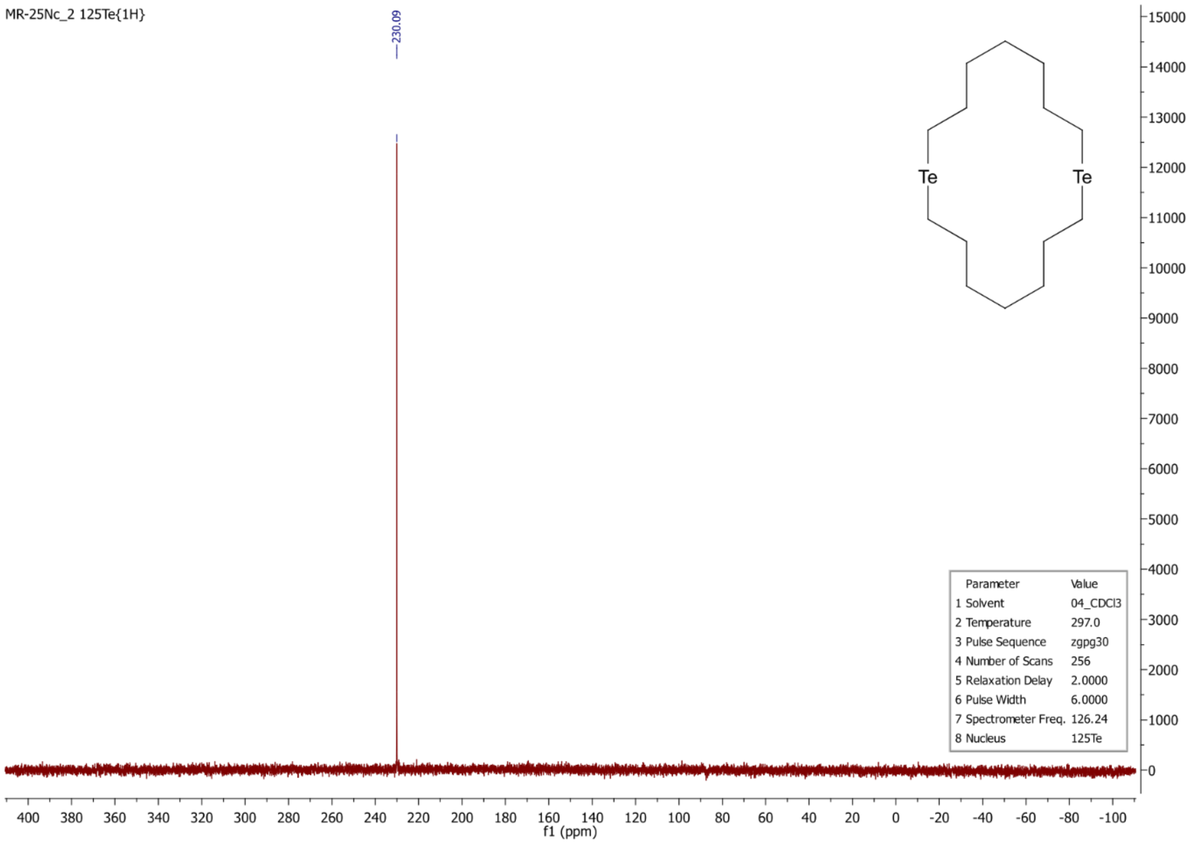

Figure S78. $^{125}Te\{^{1}H\}$-NMR spectrum of 1,9-$Te_2(CH_2)_{14}$.

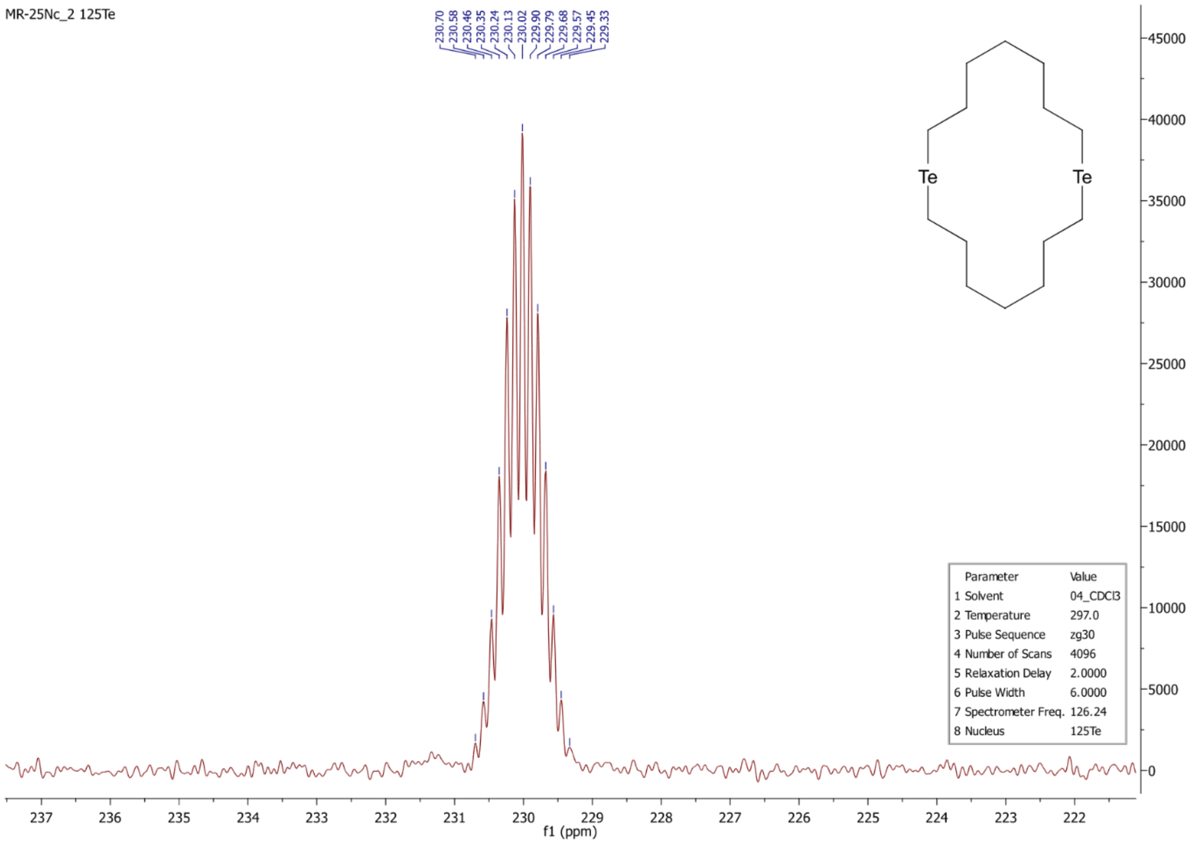

Figure S79. ^{125}Te-NMR spectrum of 1,9-$Te_2(CH_2)_{14}$.

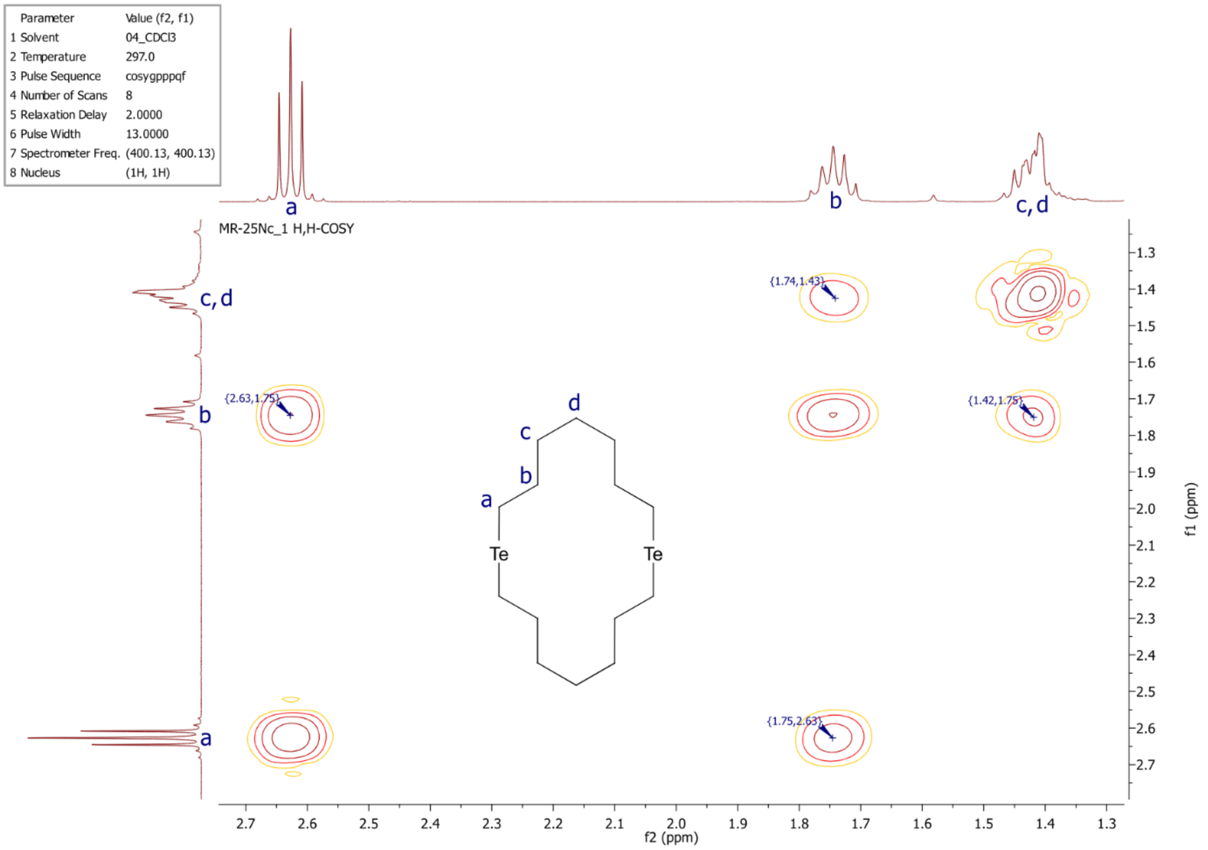

Figure S80. ^{1}H,^{1}H-COSY-NMR spectrum of 1,9-$Te_2(CH_2)_{14}$.

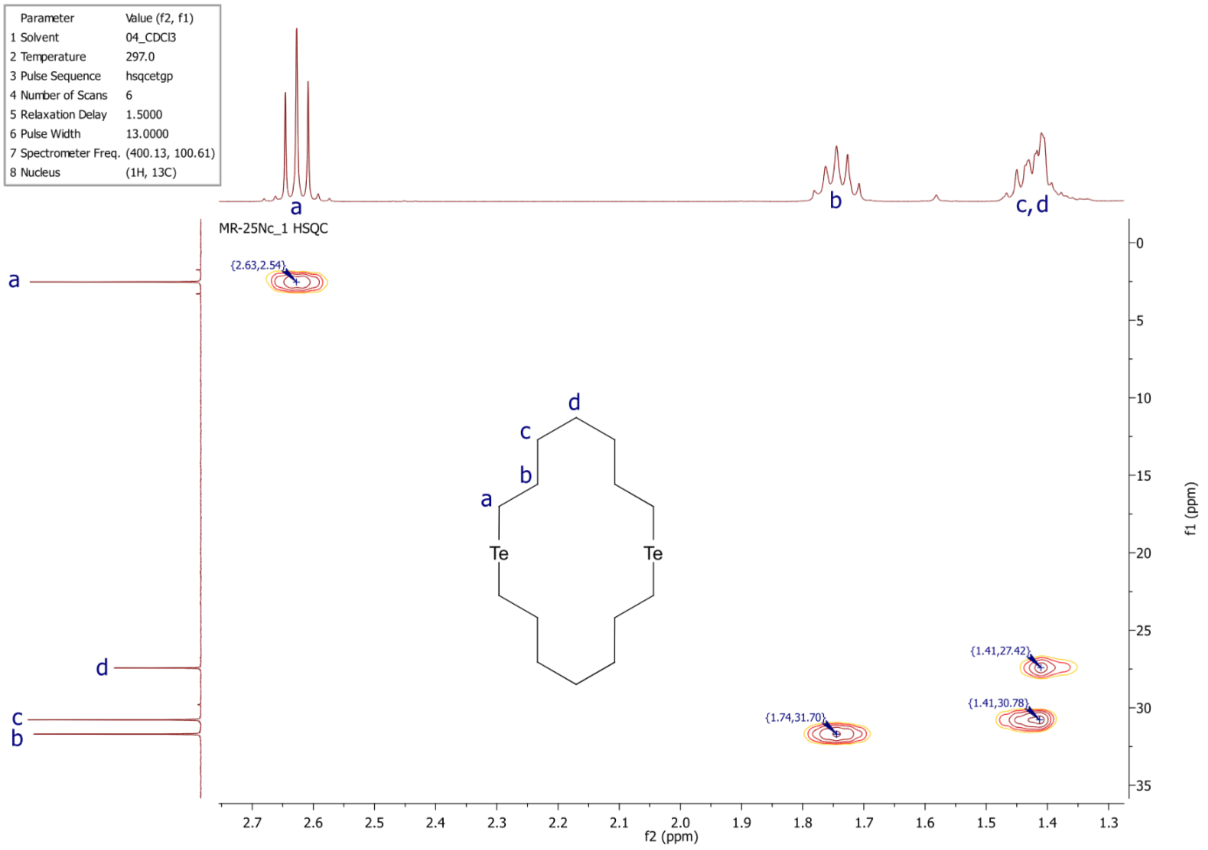

Figure S81. ^{1}H,^{13}C-HSQC-NMR spectrum of 1,9-$Te_2(CH_2)_{14}$.

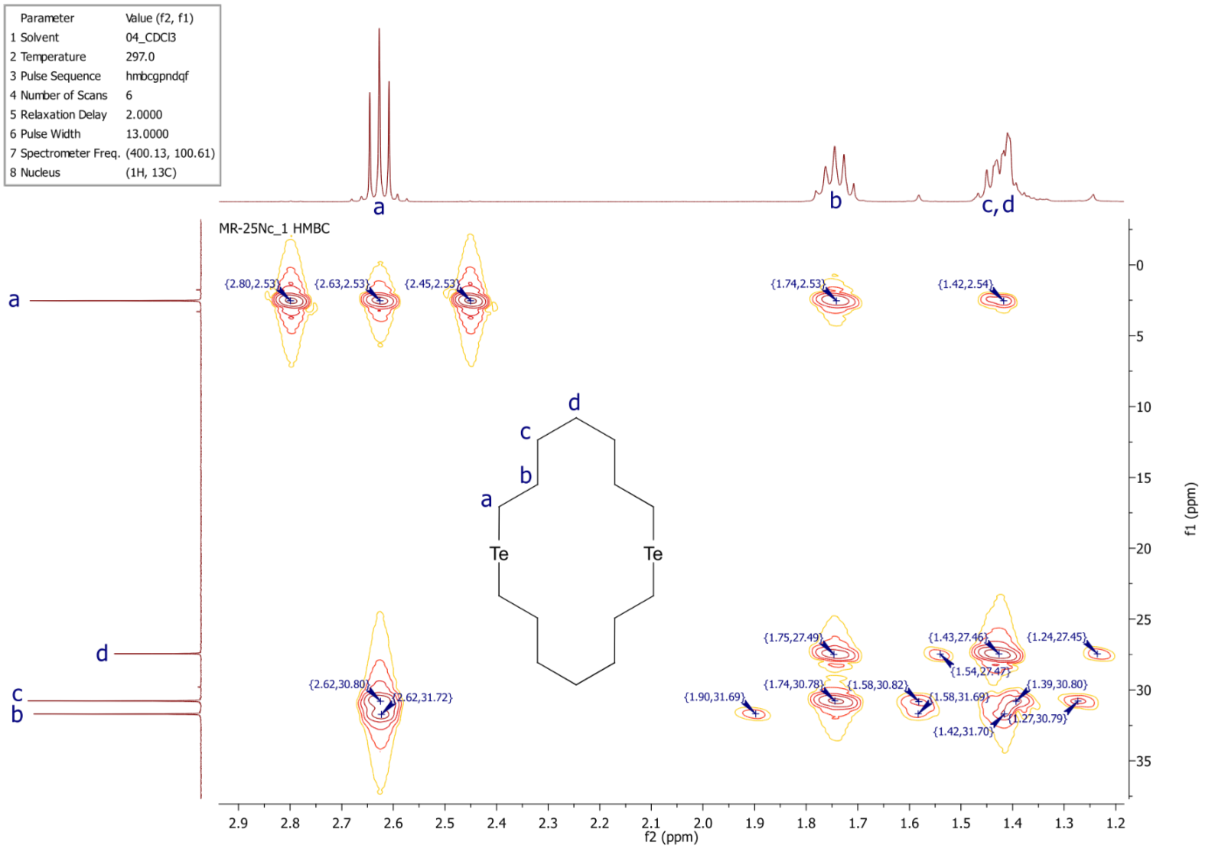

Figure S82. $^{1}H,^{13}C$-HMBC-NMR spectrum of 1,9-$Te_2(CH_2)_{14}$.

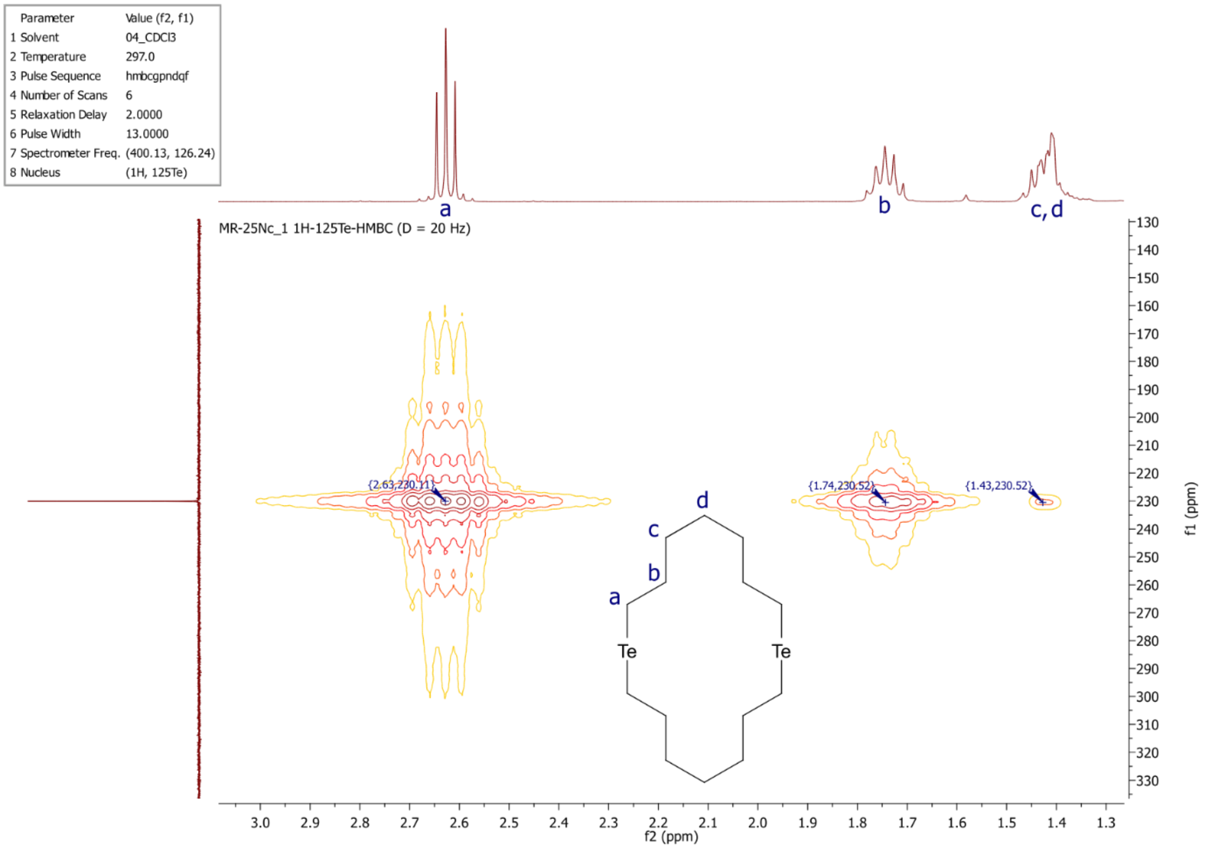

Figure S83. $^{1}H,^{125}Te$-HMBC-NMR spectrum of 1,9-$Te_2(CH_2)_{14}$.

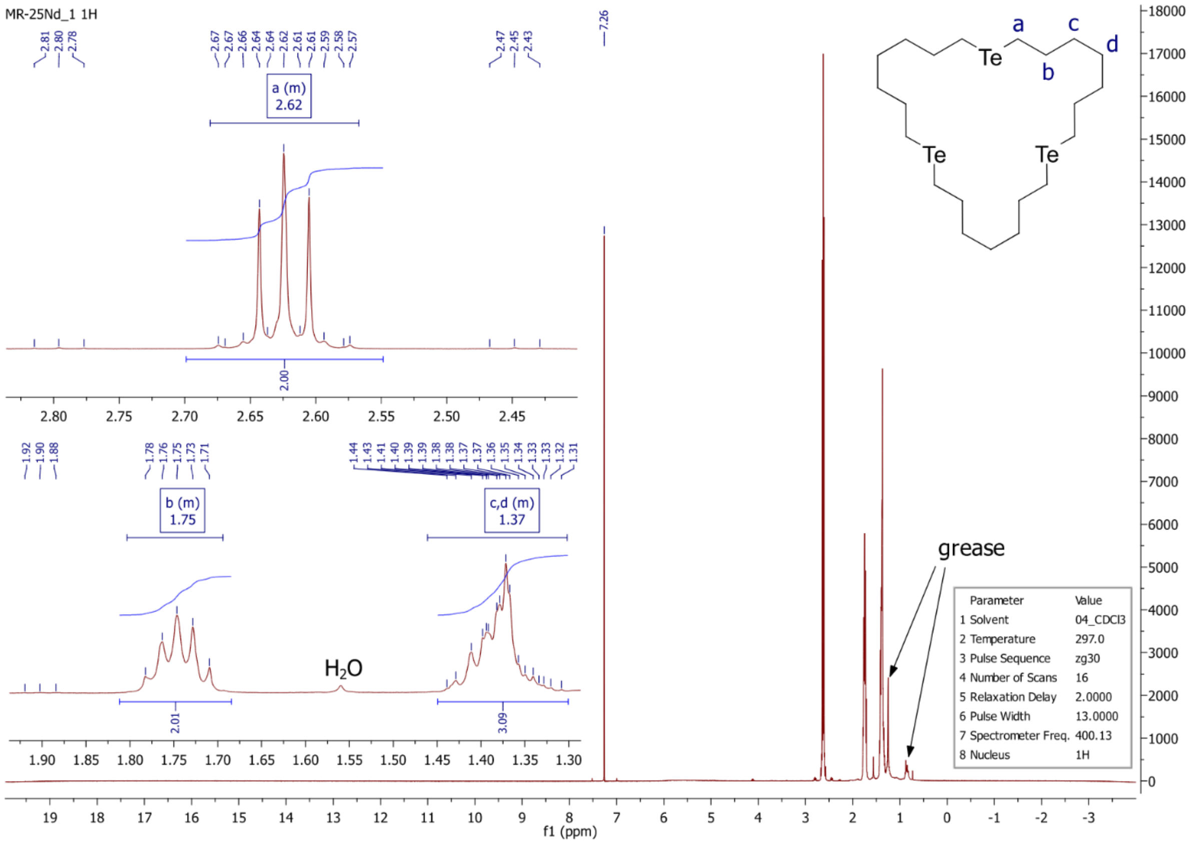

Figure S84. ^{1}H-NMR spectrum of 1,9,17-$Te_3(CH_2)_{21}$.

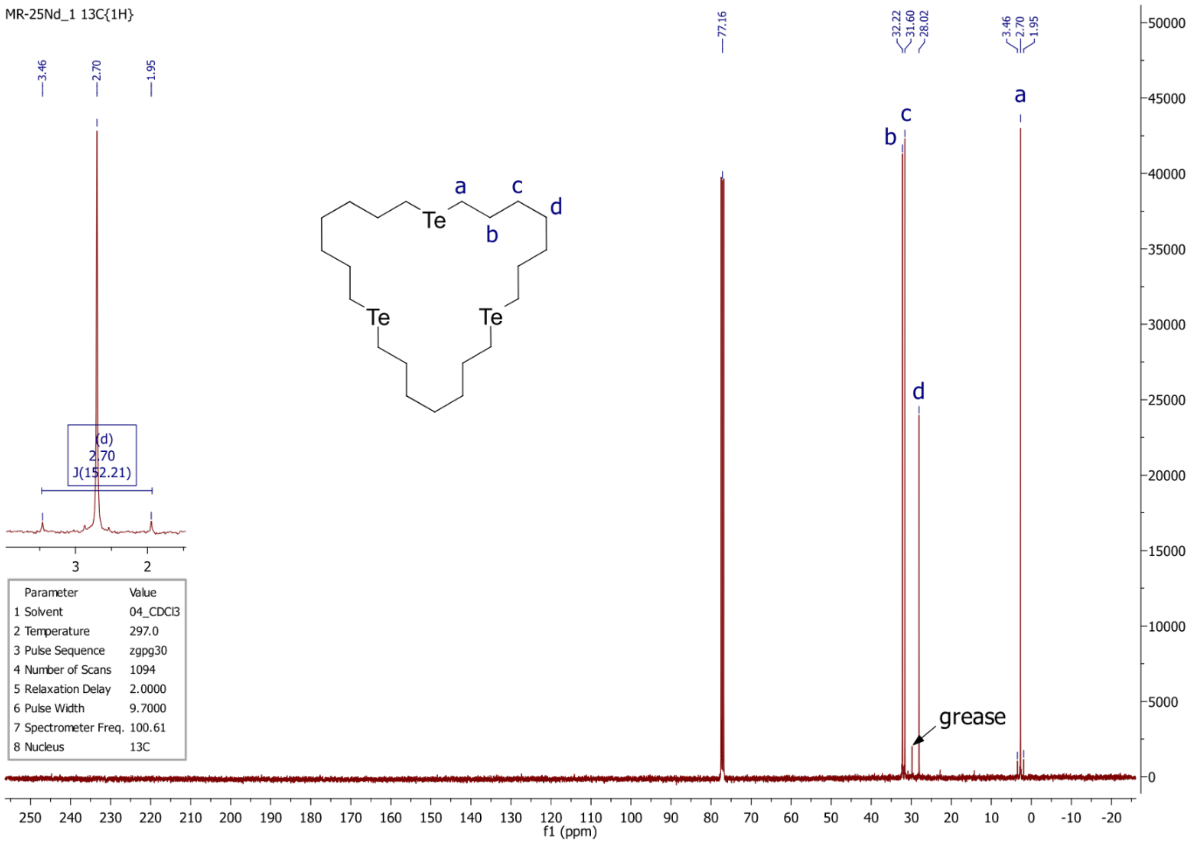

Figure S85. ^{13}C{^{1}H}-NMR spectrum of 1,9,17-$Te_3(CH_2)_{21}$.

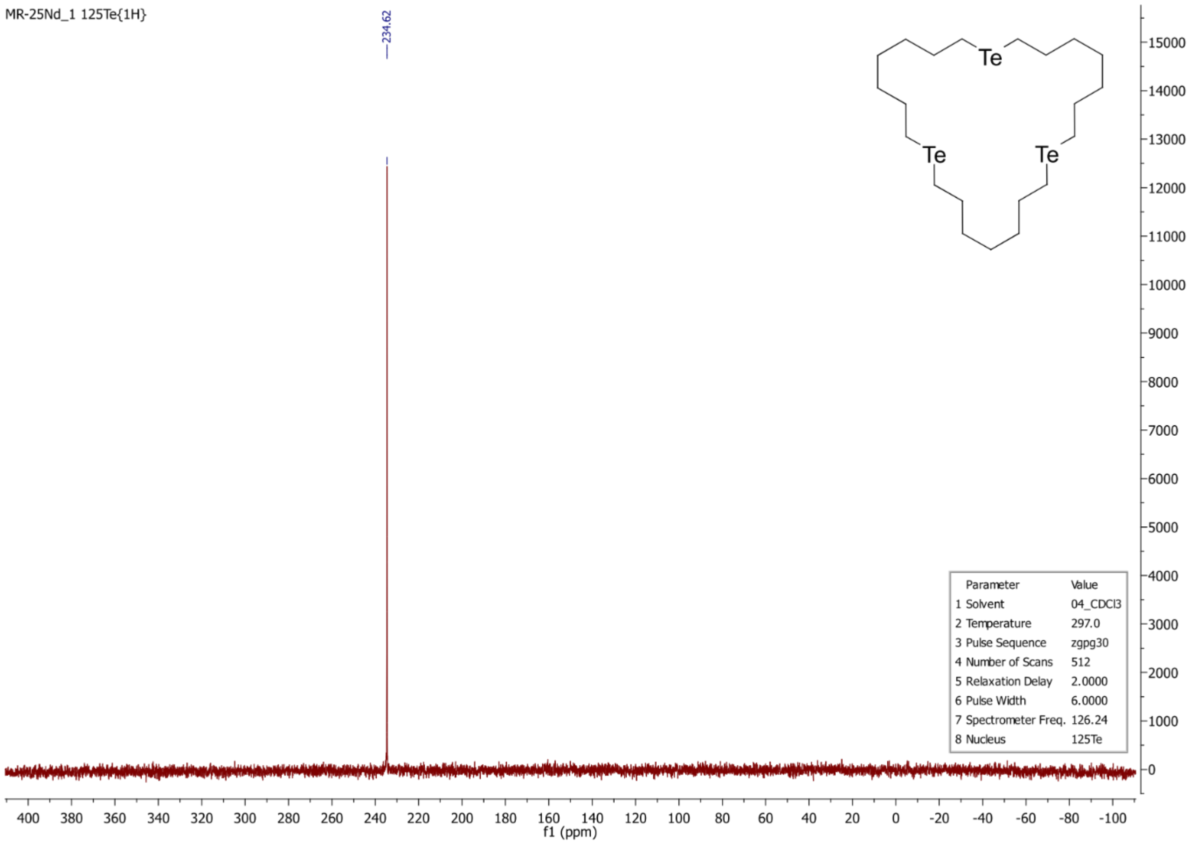

Figure S86. $^{125}Te\{^{1}H\}$-NMR spectrum of 1,9,17-$Te_3(CH_2)_{21}$.

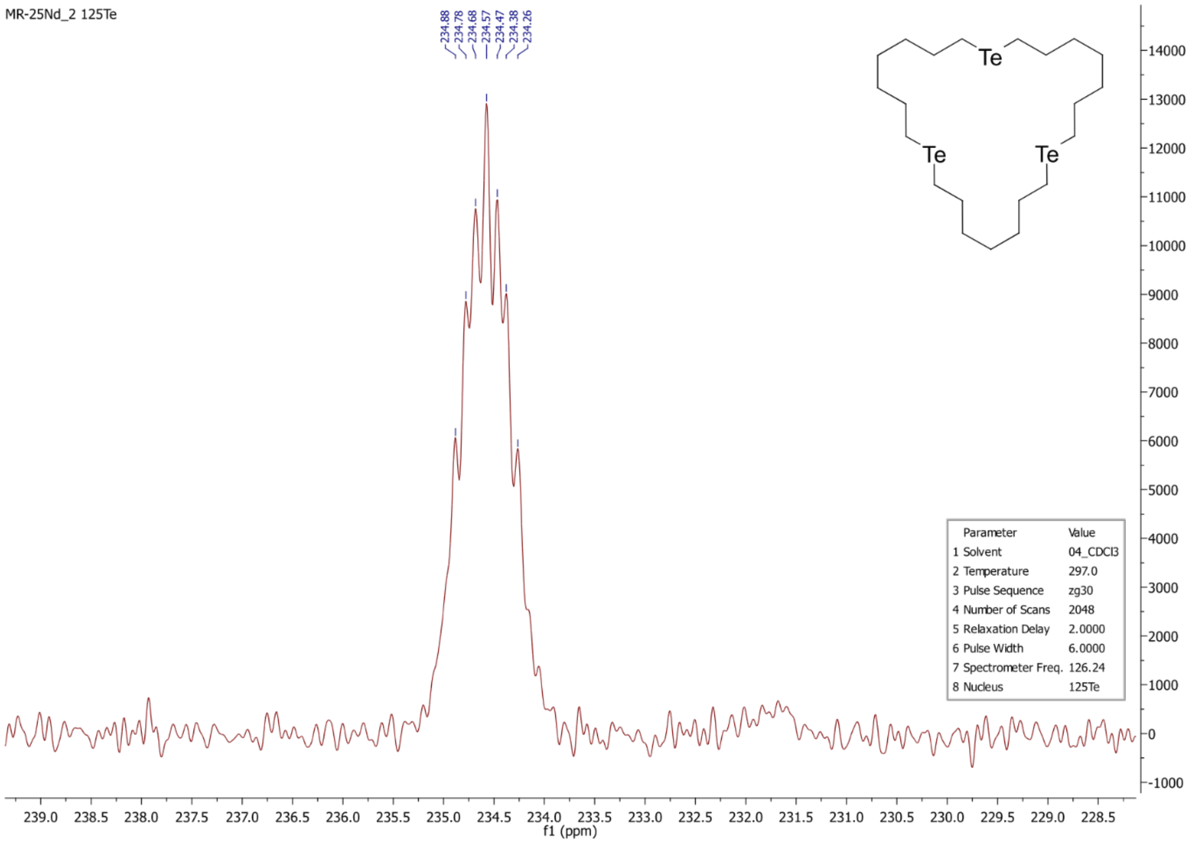

Figure S87. ^{125}Te-NMR spectrum of 1,9,17-$Te_3(CH_2)_{21}$.

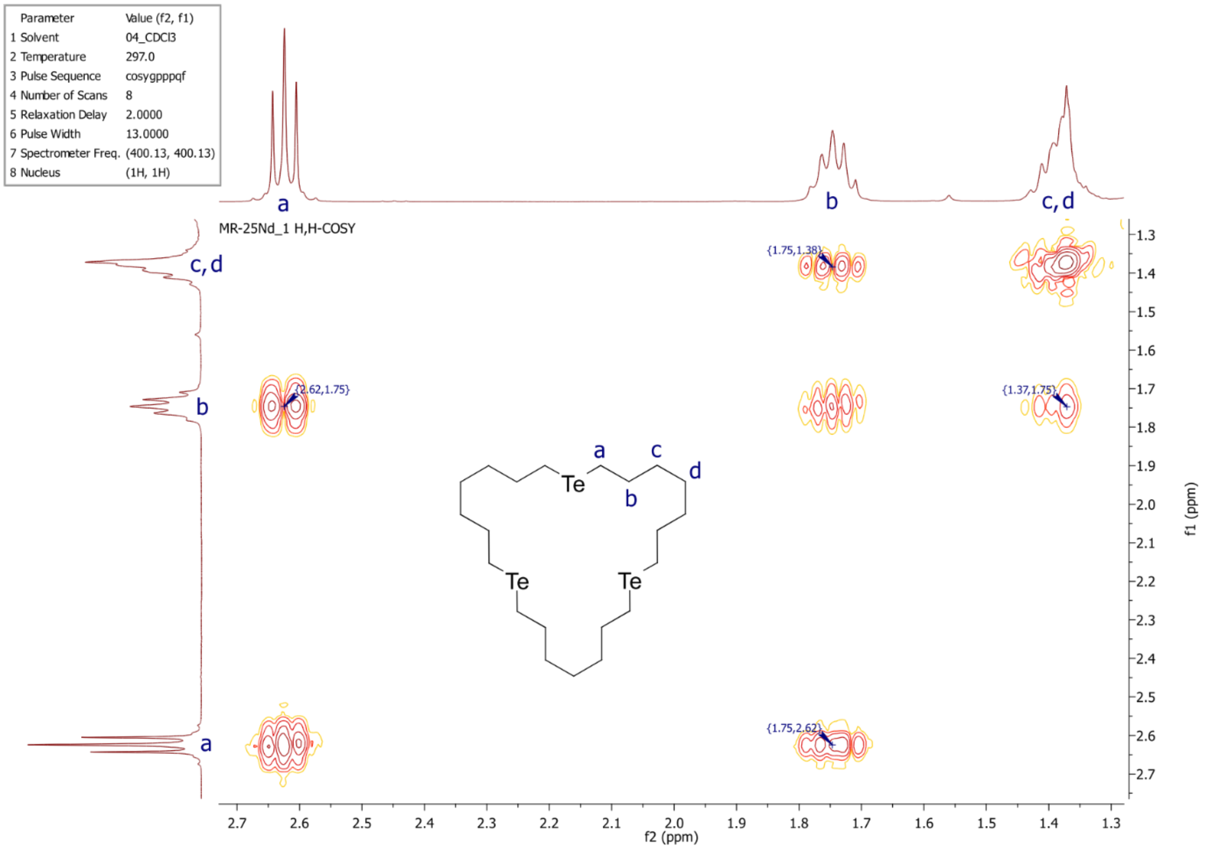

Figure S88. $^{1}H,^{1}H$-COSY-NMR spectrum of 1,9,17-$Te_3(CH_2)_{21}$.

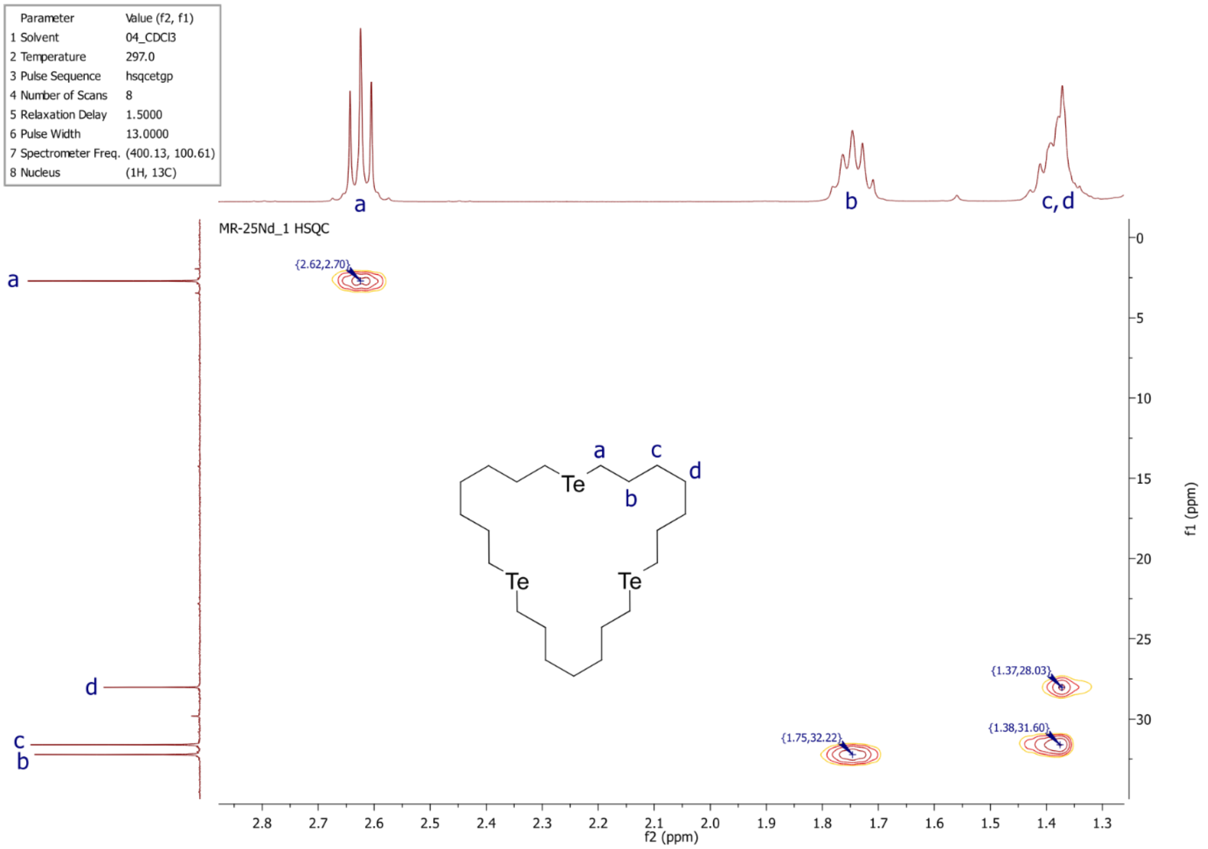

Figure S89. $^{1}H,^{13}C$-HSQC-NMR spectrum of 1,9,17-$Te_3(CH_2)_{21}$.

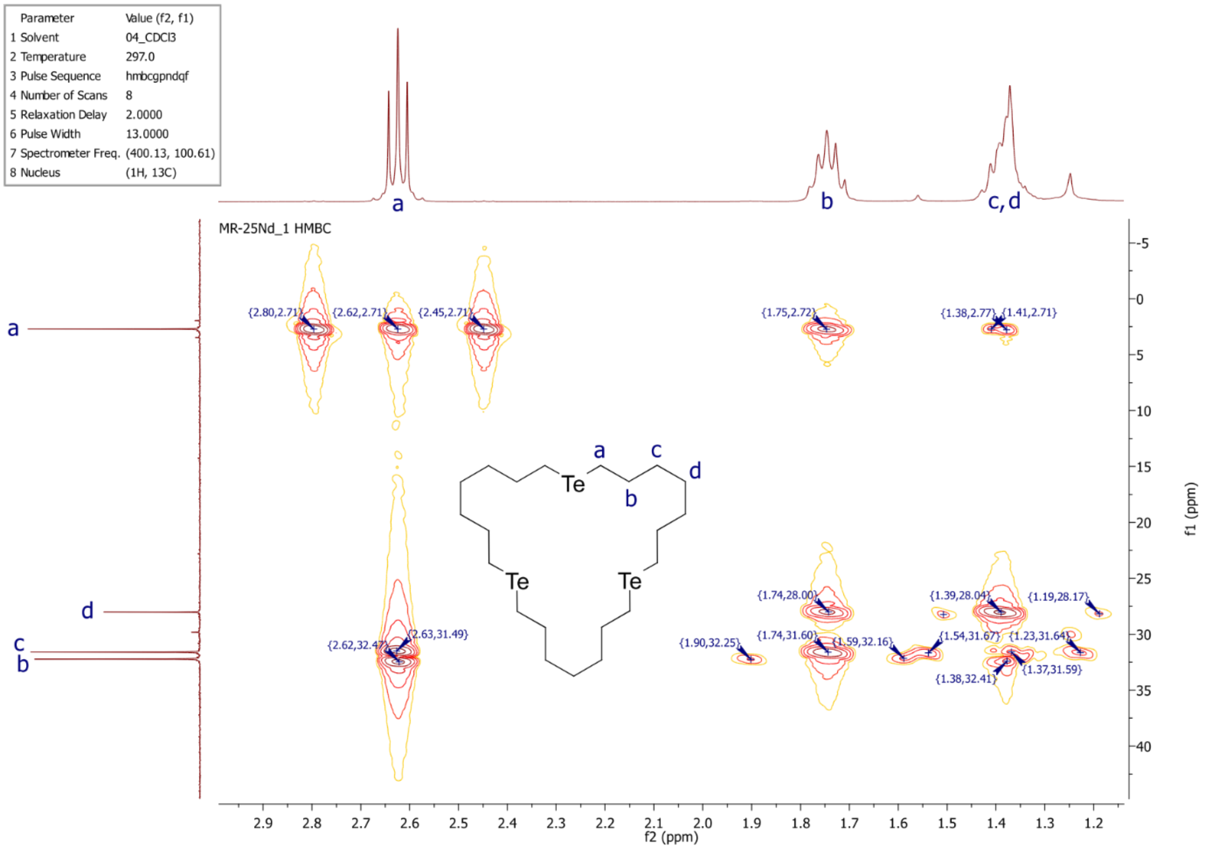

Figure S90. $^{1}H,^{13}C$-HMBC-NMR spectrum of 1,9,17-$Te_3(CH_2)_{21}$.

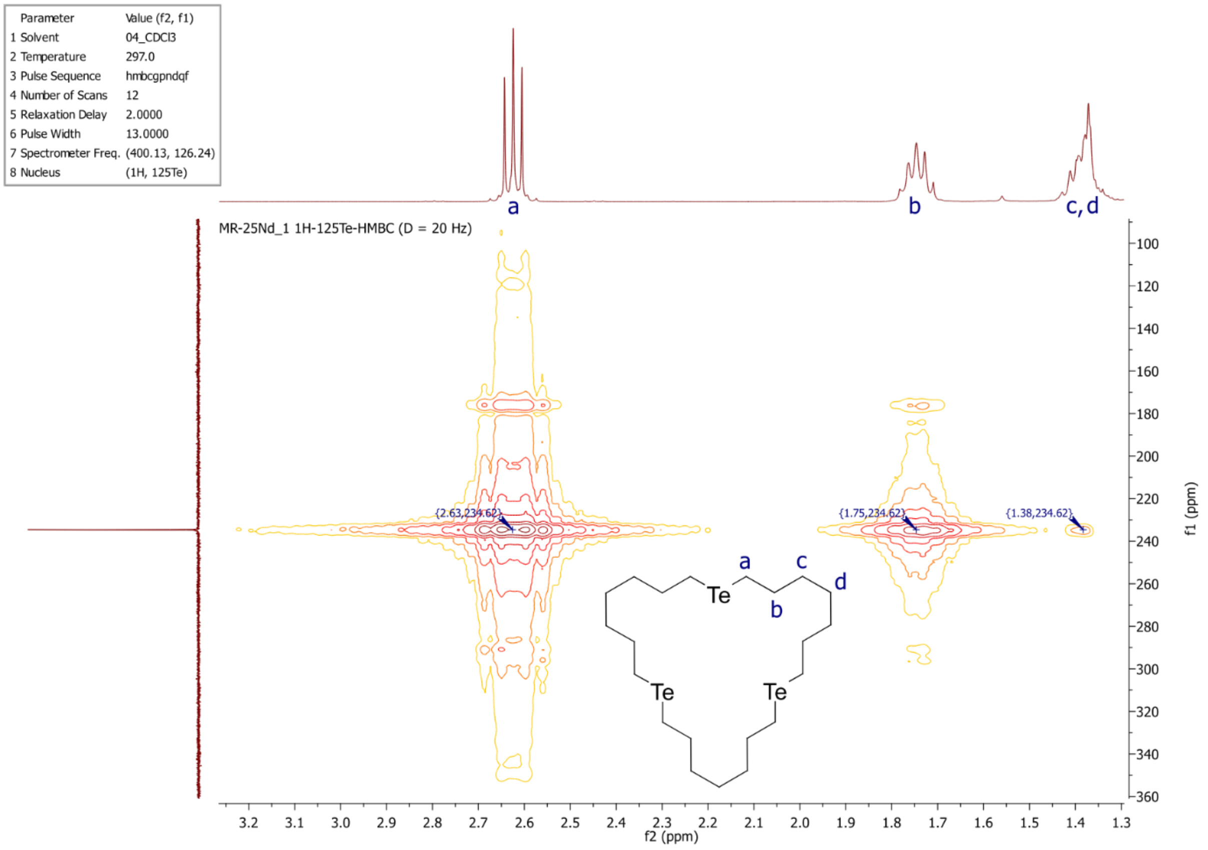

Figure S91. $^{1}H,^{125}Te$-HMBC-NMR spectrum of 1,9,17-$Te_3(CH_2)_{21}$.

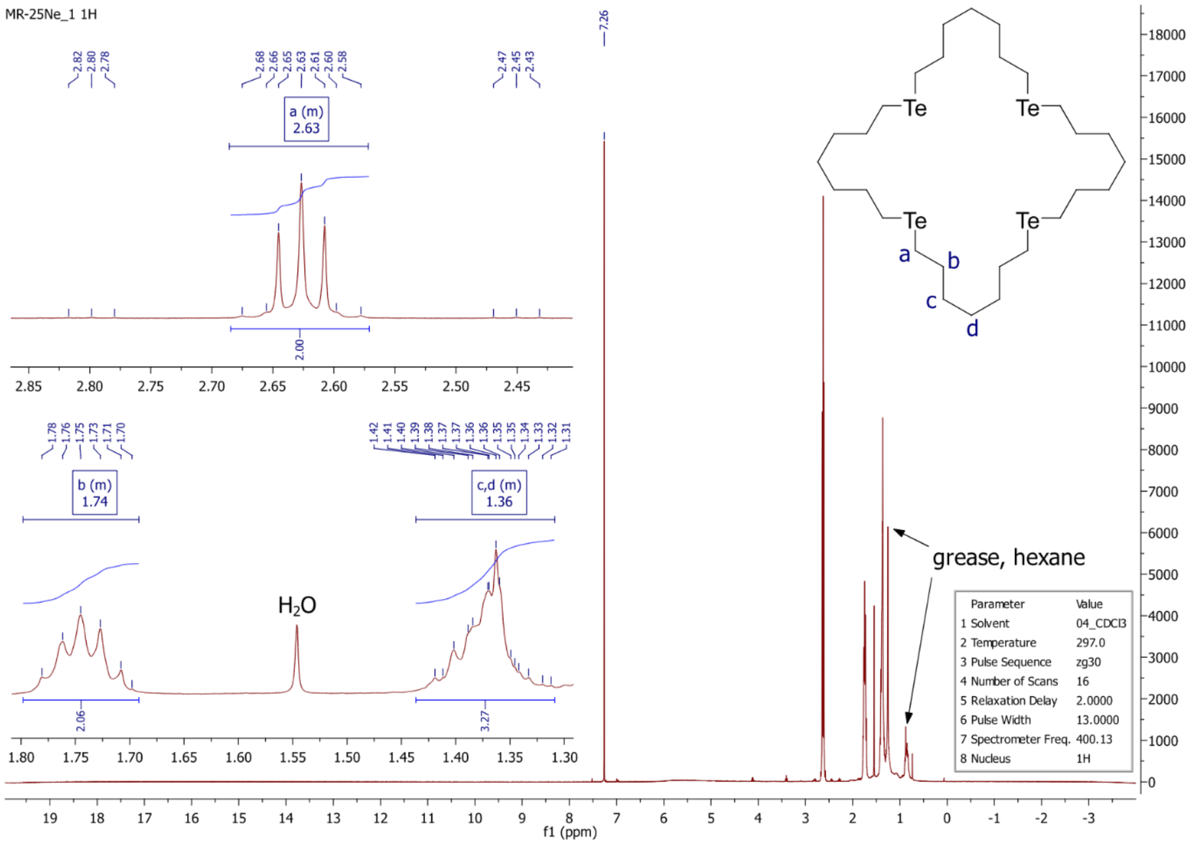

Figure S92. ^{1}H-NMR spectrum of 1,9,17,25-$Te_4(CH_2)_{28}$.

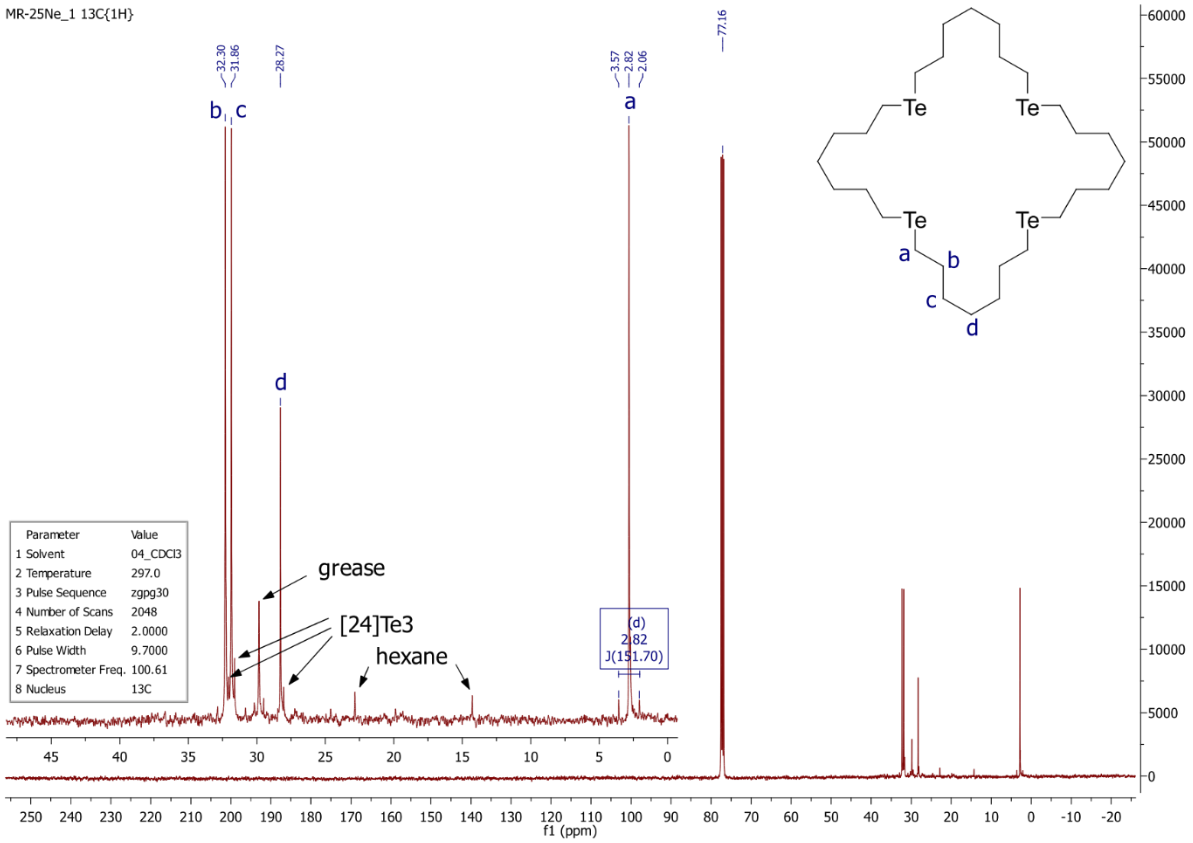

Figure S93. $^{13}C\{^1H\}$-NMR spectrum of 1,9,17,25-$Te_4(CH_2)_{28}$.

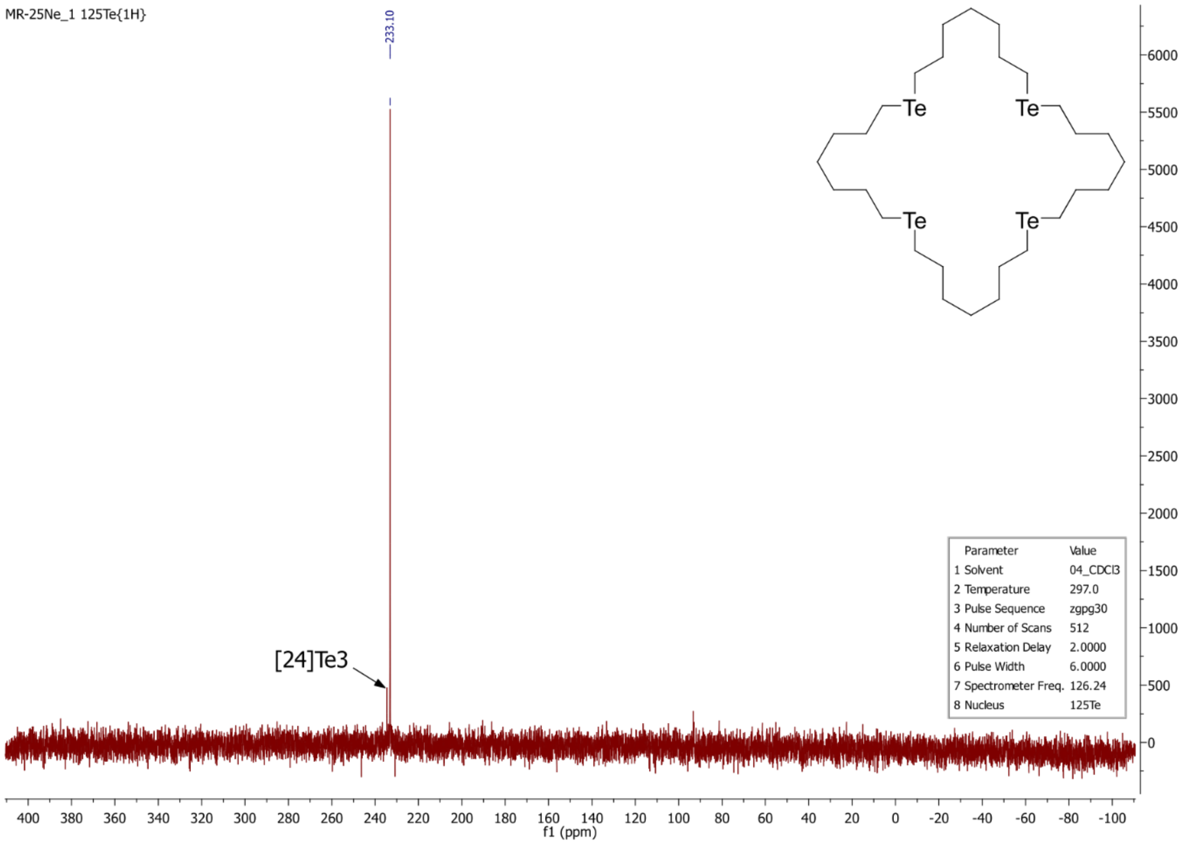

Figure S94. $^{125}Te\{^{1}H\}$-NMR spectrum of 1,9,17,25-$Te_4(CH_2)_{28}$.

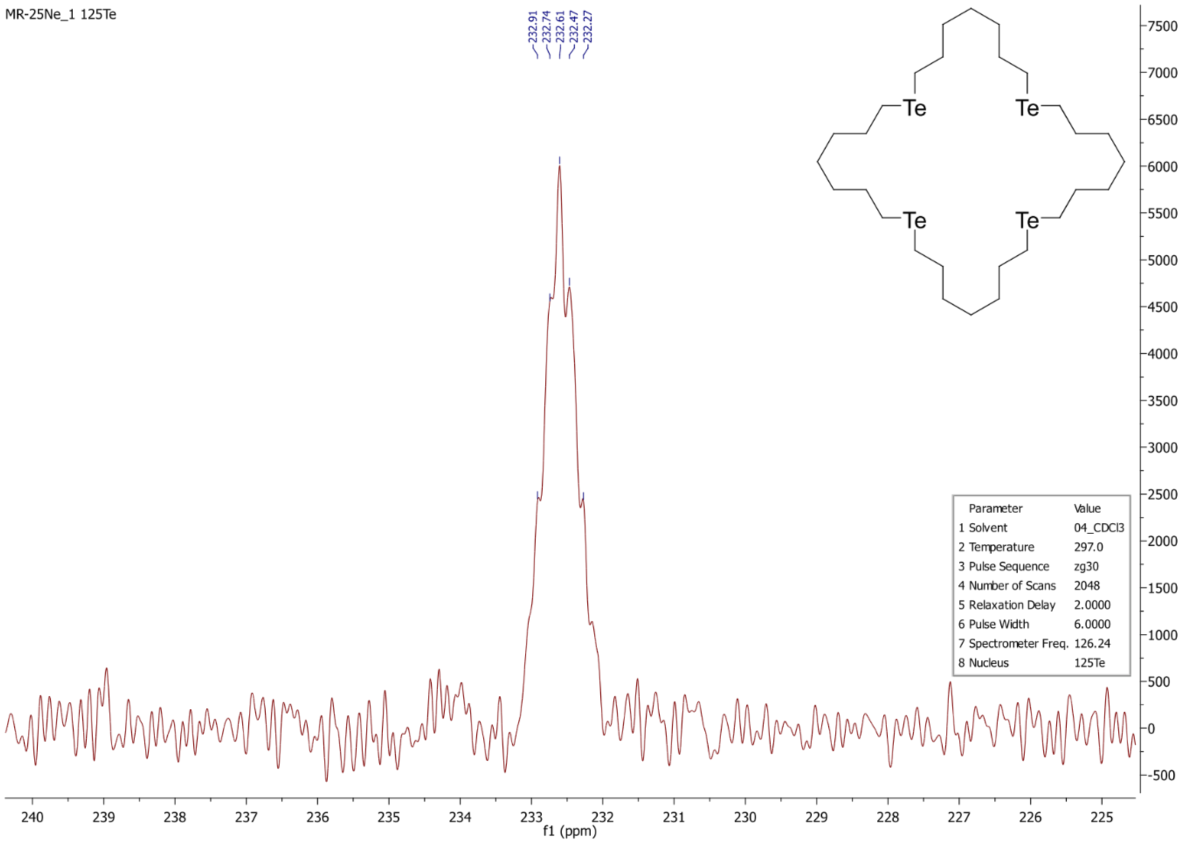

Figure S95. ^{125}Te-NMR spectrum of 1,9,17,25-$Te_4(CH_2)_{28}$.

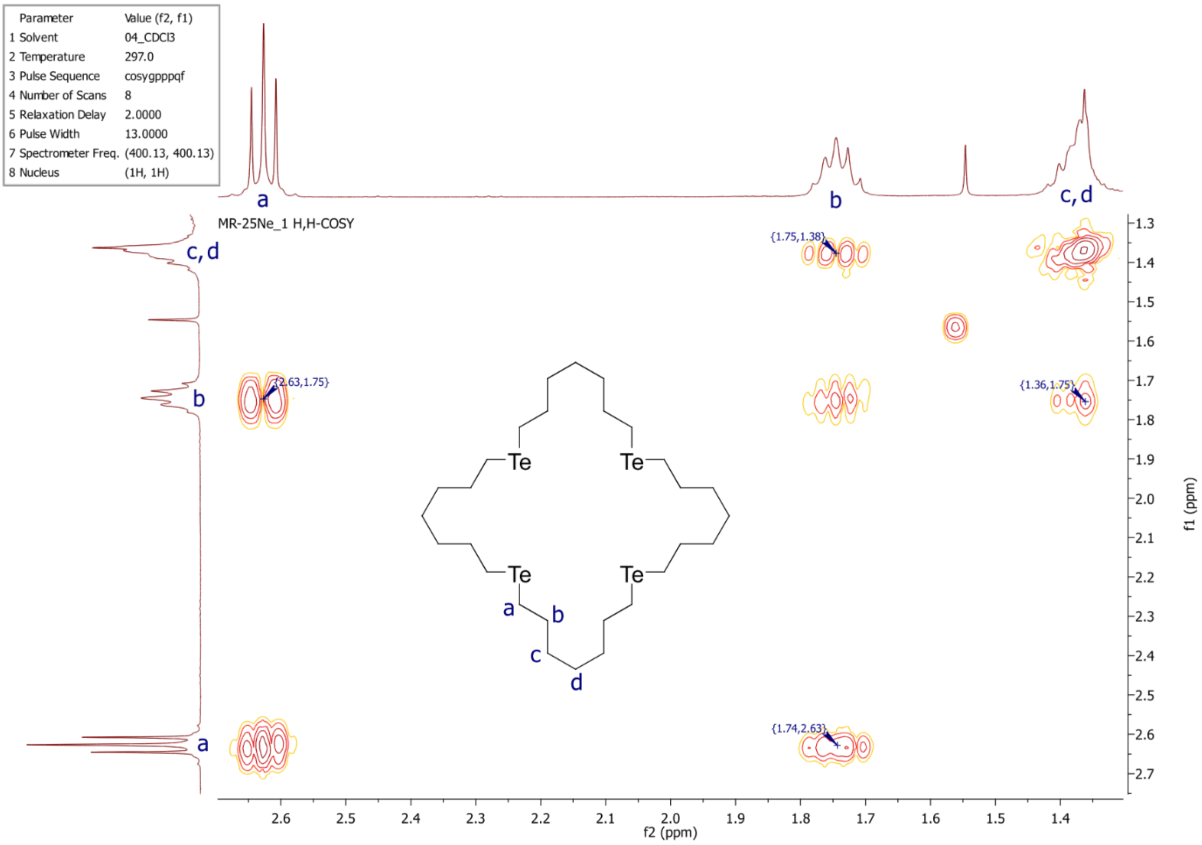

Figure S96. ^{1}H,^{1}H-COSY-NMR spectrum of 1,9,17,25-$Te_4(CH_2)_{28}$.

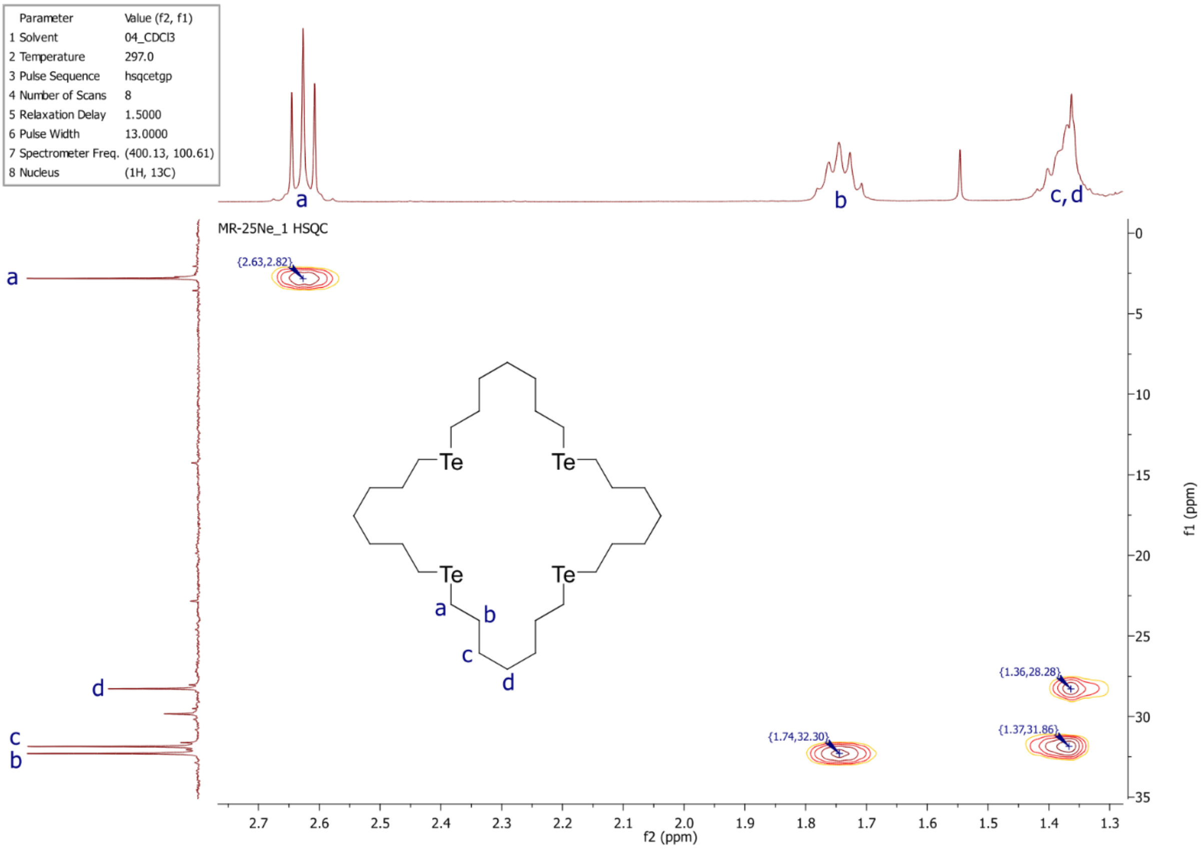

Figure S97. ^{1}H,^{13}C-HSQC-NMR spectrum of 1,9,17,25-$Te_4(CH_2)_{28}$.

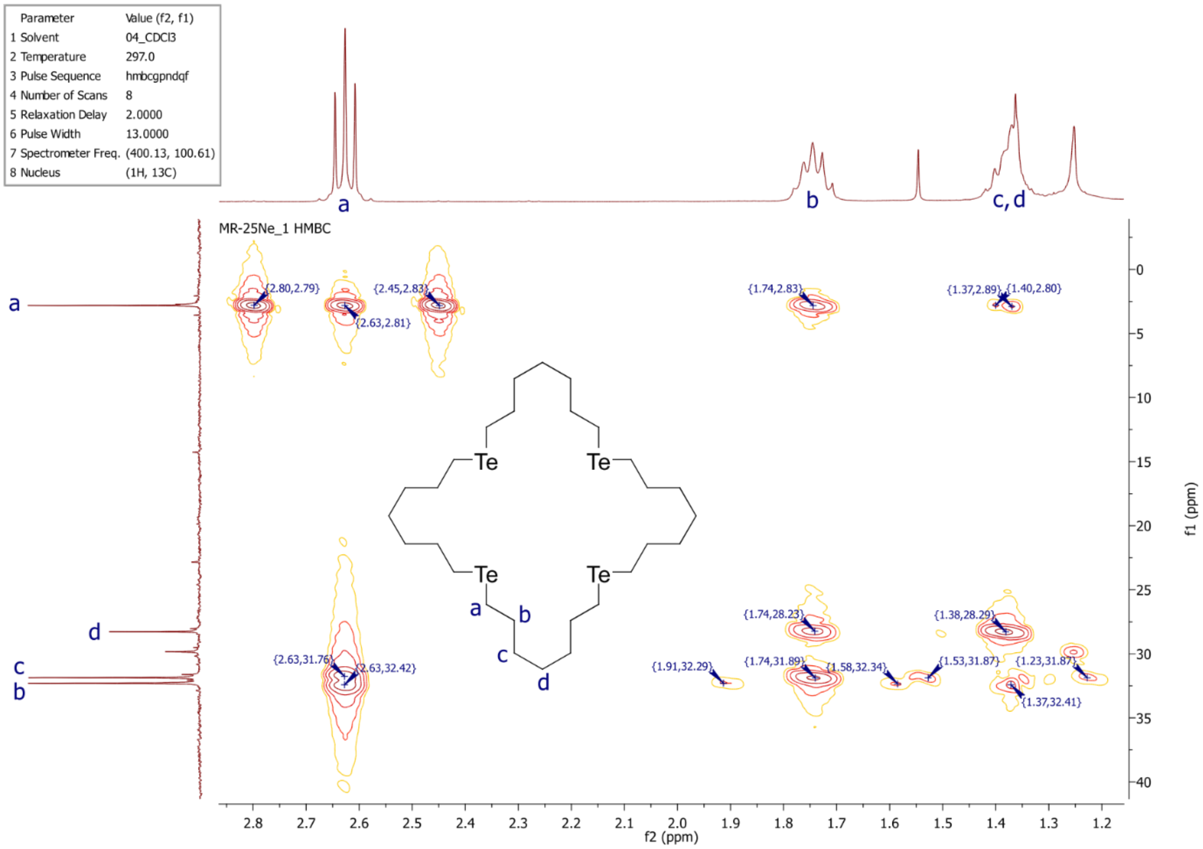

Figure S98. ^{1}H,^{13}C-HMBC-NMR spectrum of 1,9,17,25-$Te_4(CH_2)_{28}$.

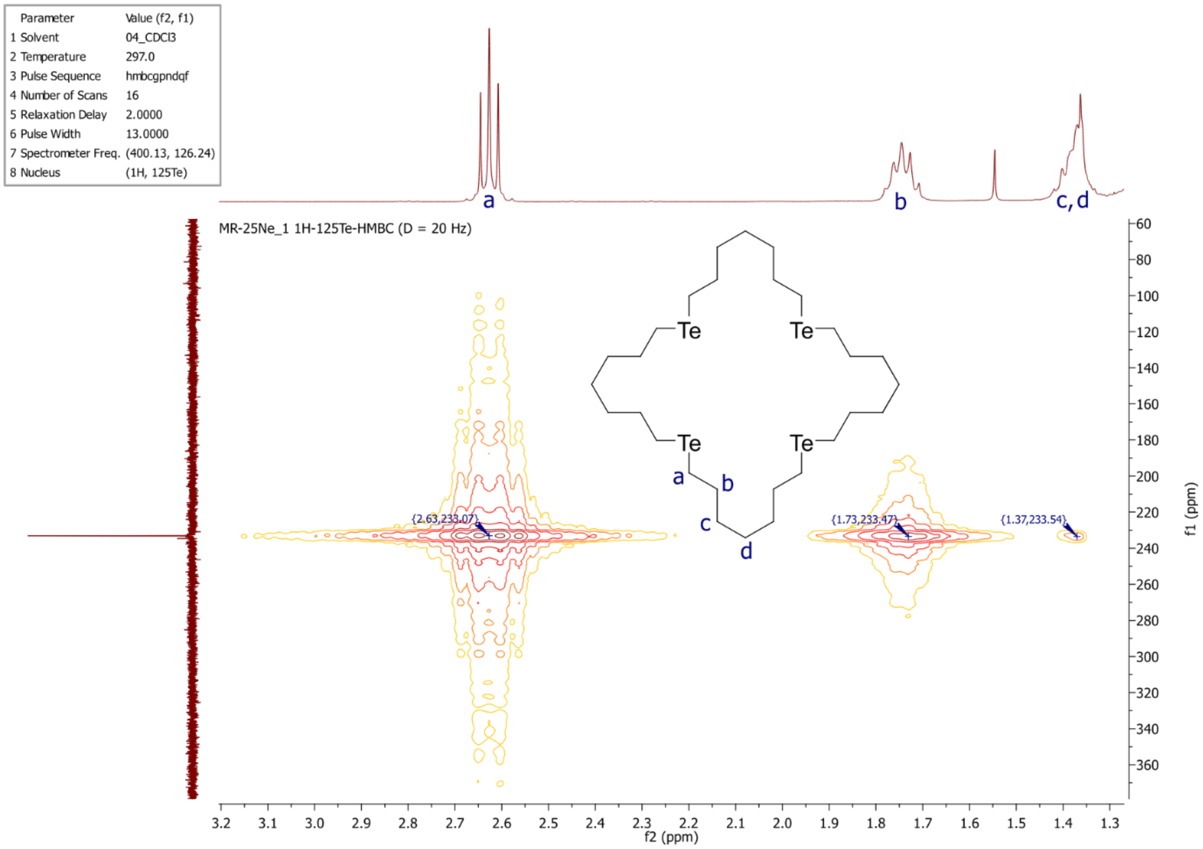

Figure S99. ^{1}H,^{125}Te-HMBC-NMR spectrum of 1,9,17,25-$Te_4(CH_2)_{28}$.

Computational Results

Table S6. Absolute energies (E), enthalpies (H), and Gibbs (G) energies (Hartree) from ORCA PBE0-D3/def2-TZVPP calculations using PCM model for solvent (EtOH).

Species	E	H	G(298 K)
$Te(CH_2)_5$	-464.438800	-464.290822	-464.330155
$Te_2(CH_2)_{10}$	-928.883108	-928.584660	-928.642016
$Te_3(CH_2)_{15}$	-1393.328278	-1392.879429	-1392.958955
$Te_4(CH_2)_{20}$	-1857.775873	-1857.177514	-1857.276320
$Te_5(CH_2)_{25}$	-2322.213743	-2321.466351	-2321.584702
$Br(CH_2)_5Br$	-5344.136343	-5343.983577	-5344.029769
Na_2Te	-592.499900	-592.493064	-592.527687
NaBr	-2736.124431	-2736.124431	-2736.124431

Table S7. Absolute energies (E), enthalpies (H), and Gibbs (G) energies (Hartree) from Crystal17 PBE0-D3/pob-TZVP calculations.

Species	E	H	G(298 K)
**Molecules**			
Na_2Te	-588.091291	-588.084413	-588.119865
NaBr	-2736.104916	-2736.100504	-2736.127167
$Te_2(CH_2)_{10}$	-920.029079		
$Te_2(CH_2)_{12}$	-998.584759		
$Te_3(CH_2)_9$	-1144.392476		
$Te_3(CH_2)_{18}$	-1497.881333		
$Te_4(CH_2)_{20}$	-1840.064283		
$Te_4(CH_2)_{24}$	-1997.168441		
$Te_4(CH_2)_{28}$	-2154.272888		
**Crystals**			
Na_2Te(*Fm*-3*m*, Z=4)	-2353.035100	-2353.002879	-2353.051716
NaBr(*Fm*-3*m*, Z=1)	-2736.210648	-2736.207639	-2736.210964
$Te_2(CH_2)_{10}$ (*P*-1, Z=2)	-1840.182750		
$Te_2(CH_2)_{10}$ (*P*1,Z=1)	-920.085362		
$Te_2(CH_2)_{12}$ ($P2_1/n$, Z=2)	-1997.302160		
$Te_3(CH_2)_9$ ($Pna2_1$, Z=4)	-4577.819295		
$Te_3(CH_2)_{18}$ (*P*-1, Z=4)	-5991.883299		
$Te_4(CH_2)_{20}$ (*I*-42*m*, Z=1)	-1840.182038		
$Te_4(CH_2)_{24}$ (*P*4/*nnc*, Z=2)	-3994.576339		
$Te_4(CH_2)_{28}$ (*I*-42*m*, Z=1)	-2154.413229		
